# Photo- and Metal-Mediated Deconstructive Approaches to Cyclic Aliphatic Amine Diversification

**DOI:** 10.1021/jacs.3c01318

**Published:** 2023-05-12

**Authors:** David M. Soro, Jose B. Roque, Jonas W. Rackl, Bohyun Park, Stefan Payer, Yuan Shi, J. Craig Ruble, Alexey L. Kaledin, Mu-Hyun Baik, Djamaladdin G. Musaev, Richmond Sarpong

**Affiliations:** † Department of Chemistry, University of California, Berkeley, California 94720, United States; ‡ Cherry L. Emerson Center for Scientific Computation, and Department of Chemistry, Emory University, 1515 Dickey Drive, Atlanta, Georgia 30322, United States; § Department of Chemistry, Korea Advanced Institute of Science and Technology (KAIST), Daejeon 34141, Korea; ∥ Center for Catalytic Hydrocarbon Functionalizations, Institute for Basic Science (IBS), Daejeon 34141, Korea; ⊥ Discovery Chemistry Research and Technologies, Eli Lilly and Company, Indianapolis, Indiana 46285, United States

## Abstract

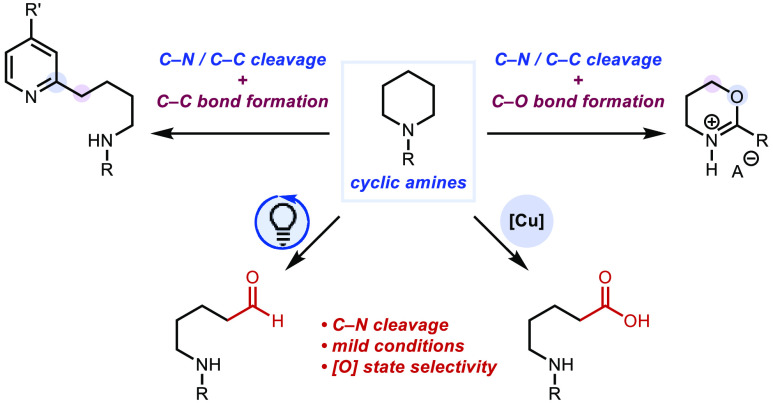

Described herein are studies toward the core modification of cyclic aliphatic amines using either a riboflavin/photo-irradiation approach or Cu(I) and Ag(I) to mediate the process. Structural remodeling of cyclic amines is explored through oxidative C–N and C–C bond cleavage using peroxydisulfate (persulfate) as an oxidant. Ring-opening reactions to access linear aldehydes or carboxylic acids with flavin-derived photocatalysis or Cu salts, respectively, are demonstrated. A complementary ring-opening process mediated by Ag(I) facilitates decarboxylative Csp^3^–Csp^2^ coupling in Minisci-type reactions through a key alkyl radical intermediate. Heterocycle interconversion is demonstrated through the transformation of N-acyl cyclic amines to oxazines using Cu(II) oxidation of the alkyl radical. These transformations are investigated by computation to inform the proposed mechanistic pathways. Computational studies indicate that persulfate mediates oxidation of cyclic amines with concomitant reduction of riboflavin. Persulfate is subsequently reduced by formal hydride transfer from the reduced riboflavin catalyst. Oxidation of the cyclic aliphatic amines with a Cu(I) salt is proposed to be initiated by homolysis of the peroxy bond of persulfate followed by α-HAT from the cyclic amine and radical recombination to form an α-sulfate adduct, which is hydrolyzed to the hemiaminal. Investigation of the pathway to form oxazines indicates a kinetic preference for cyclization over more typical elimination pathways to form olefins through Cu(II) oxidation of alkyl radicals.

## Introduction

**Figure 1:**
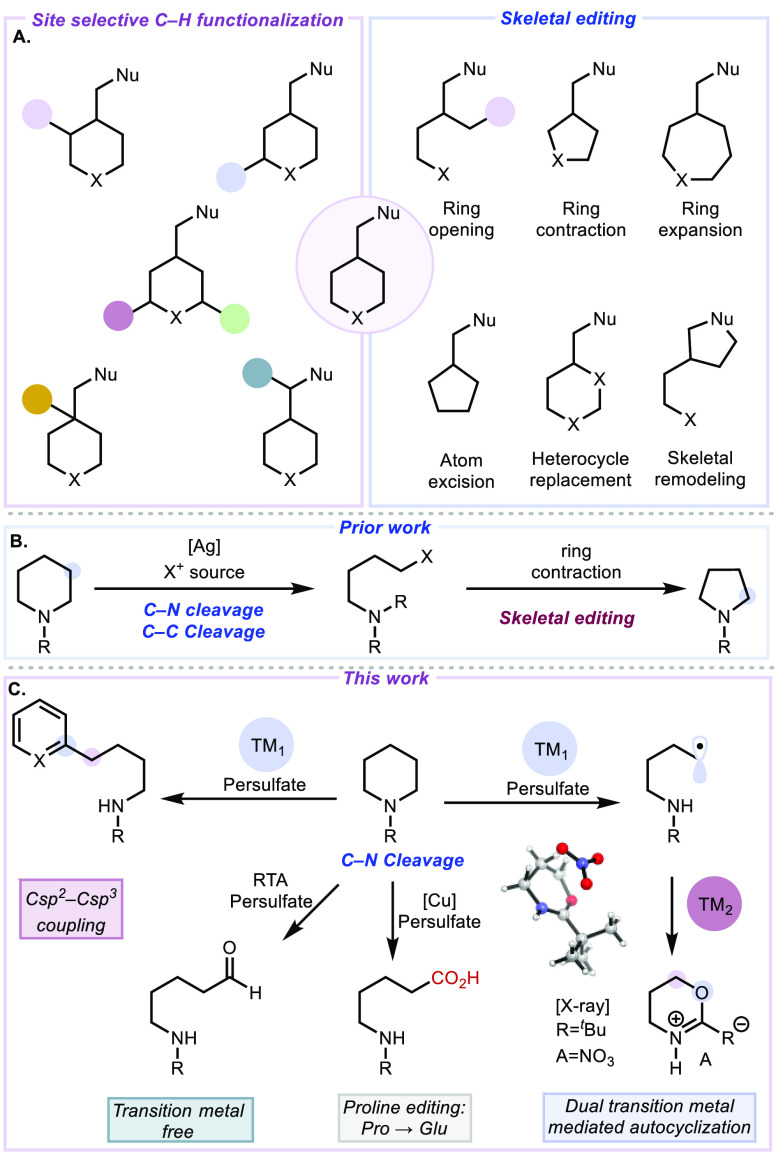
(A) Skeletal editing. (B) Prior work. (C) This work.

Modern drug discovery has benefited from advancements in chemical reaction development.^1^ For example, the development of selective C–H functionalization has changed how synthetic chemists approach the retrosynthetic analysis of bioactive compounds.^2^ Largely, disconnections of molecules to simpler precursors through a retrosynthesis exercise focus on removal of peripheral groups.^3,4^ As a result, in the forward sense, syntheses and late-stage diversification of molecules have mostly focused on peripheral modification. Alternatively, an emerging approach to access novel chemical space has been centered around making modifications to the core framework of molecules, contrasting traditional molecular structural diversification approaches.^5,6^ Recognizing a need for more methods to accomplish modification of the core framework of molecules (skeletal editing) to access unique chemical and functional space, we have initiated a program aimed at the deconstructive functionalization (i.e., breaking of traditionally strong bonds, such as C–C and C–N bonds, and functionalization of their constituent atoms) of saturated cyclic amines in order to access new chemical space. We have primarily focused on saturated azacycles, especially piperidines, given their prevalence in pharmaceuticals,^7^ as well as agrochemicals.^8^ Our previous studies have identified ring opening,^9^ ring contraction,^10^ as well as heterocycle replacement remodeling methods^11^ for achieving skeletal diversity (Figure 1A).

We have found oxidative pathways to be particularly effective for the deconstructive functionalization of cyclic aliphatic amines (saturated azacycles).^9,10^ In complementary approaches, others have demonstrated oxidative bond cleavage of cyclic amines under aerobic conditions^12−14^ as well as through the use of metal-oxo species.^15−17^ While these strategies provide effective ways to achieve diversity in cyclic amine transformations, metal-oxo catalysts tend to favor oxidative pathways leading to imide products;^18^ peripheral modification is favored over core modification for these catalysts. Our goal was to develop a straightforward, distinct approach that would achieve a reconfiguration of the cyclic amine skeleton through structural transformations beyond ring opening. Peroxydisulfate (persulfate) has been shown to be a versatile oxidant in various contexts. Given its high oxidation potential (redox potential = 2.01 V in aqueous solution),^19,20^ the redox reactivity of persulfate with transition metals or organic substrates can lead to different outcomes. In particular, persulfate has been used in the oxidative functionalization of amines, providing access to α-amino radicals and iminium intermediates.^21^ On this basis, our lab previously reported the deconstructive diversification of cyclic amines using a Ag(I)-persulfate combination (Figure 1B).^9,10^ With the goal of increasing the diversity of scaffolds that could be accessed, we envisioned that persulfate might serve a key role in the deconstructive functionalization of saturated cyclic amines by mediating the cleavage of the C–N bond to provide novel acyclic structures. By pairing the persulfate oxidant with various redox-active co-reagents, we anticipated tuning the deconstructive process in order to access a broad range of scaffolds.

Herein, we present complementary efforts that achieve saturated azacycle diversification. Specifically, we report (i) two mild oxidative ring-opening methods using riboflavin-persulfate and copper-persulfate combinations that generate aldehydes and acyclic alkyl carboxylic acids, respectively, (ii) direct Csp^2^–Csp^3^ bond formation through a ring-opening/Minisci process, and (iii) autocyclization by leveraging a dual transition metal system to convert between heterocycles (Figure 1C).

**Figure 2:**
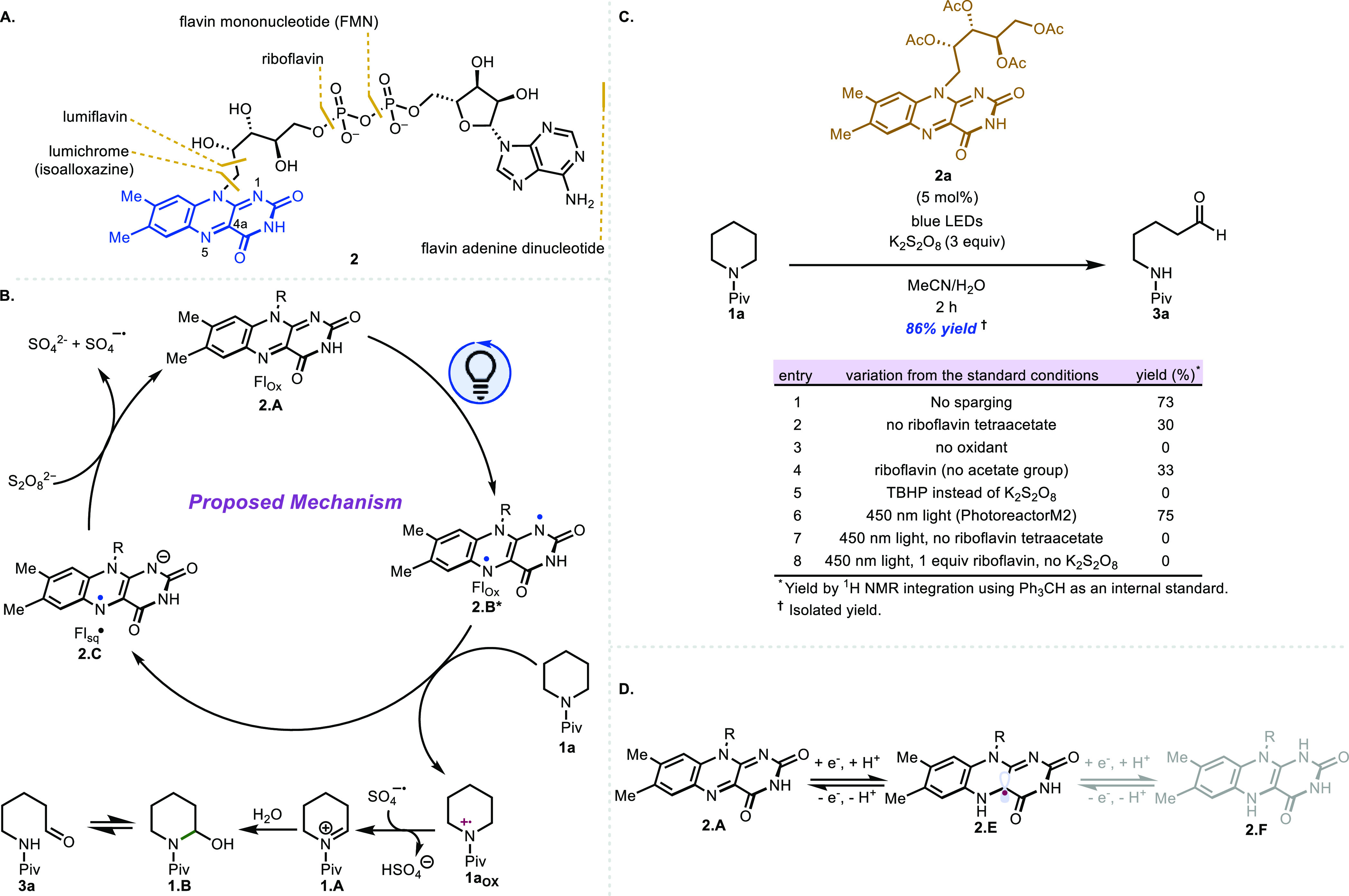
(A) Flavin derivatives. (B) Proposed reaction mechanism for the ring-opening oxidation of cyclic amines using riboflavin tetraacetate. (C) Selected reaction optimization experiments (see the Supporting Information for full details). (D) Typical redox states of flavin molecules.

Flavins, a set of molecules featuring an isoalloxazine core (Figure 2A), are key facilitators in a subset of biological electron transfer processes including the oxidation of amines by monoamine oxidases.^22^ They have also been used in nonbiological contexts for similar purposes.^23^ As such, we wondered whether they could effect nonbiological oxidative diversification of cyclic aliphatic amines. A general mechanism for our proposed transformation of saturated cyclic amines using the riboflavin-persulfate system is depicted in Figure 2B. Excitation of an isoalloxazine derivative (**2.A**) was expected to generate an excited state photocatalyst (2.B*). This excited state species should then oxidize cyclic amine **1a** via photoinduced electron transfer to produce an amidyl radical cation (**1a_ox_**) and reduced photocatalyst (**2.C**). Oxidation of the reduced photocatalyst by a persulfate ion would regenerate quinone **2.A** as well as a sulfate radical anion. The sulfate radical anion was anticipated to effect α-amino C–H abstraction of **1a_ox_** to generate iminium ion **1.A**. As previously proposed by us using the combination of Ag(I) and persulfate,^9,10^ the resulting iminium ion (**1.A**) would then be trapped by H_2_O to give hemiaminal **1.B**, which would suffer heterolytic C–N bond cleavage to furnish aldehyde **3a**. A pivaloyl group on the nitrogen atom was identified previously to be optimal in favoring the open-chain aminoaldehyde product (**3a**).^10^

## Results and Discussion

### Bio-Inspired Deconstructive Functionalization

We commenced our investigations of the oxidative C–N bond cleavage by evaluating a broad range of photoredox catalysts, oxidants, and solvent combinations (see the Supporting Information for details). After extensive optimization, we identified the conditions shown in Figure 2C that employ 5 mol % of riboflavin tetraacetate (**2a**), 3 equivalents of K_2_S_2_O_8_ in a 1:1 (v/v) mixture of MeCN/H_2_O, and irradiation with blue light-emitting diodes (Kessil brand A160WE Tuna Blue LED 40 W lamp).

**Scheme 1:**
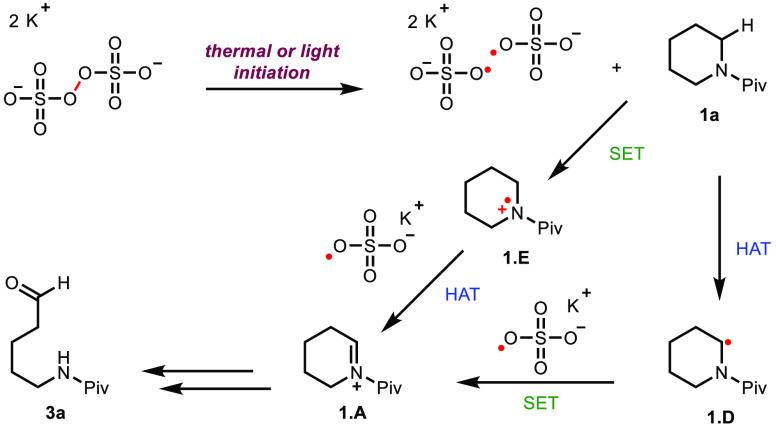
Light- or Thermal-Driven Homolysis of Persulfate

We considered whether the riboflavin photocatalyst might operate through the fully oxidized quinone state (see **2.A** in Figure 2D) as well as the one-electron-reduced semiquinone state (see **2.E** in Figure 2D) without reaching the fully reduced hydroquinone state (see **2.F** in Figure 2D) although that remained to be fully supported by additional studies. We hypothesized that upon generation of semiquinone **2.E**, oxidation by persulfate occurs to regenerate **2.A**, the corresponding sulfate dianion, and sulfate radical anion. Furthermore, the sulfate radical anion can also oxidize semiquinone **2.E**. Performing the reaction without sparging the reaction mixture (entry 1) led to a slightly diminished yield, presumably due to some catalyst deactivation by molecular oxygen. In the absence of riboflavin tetraacetate, only a 30% yield of product is obtained (entry 2). Likely, in this case, the product arises from light or thermal activation in which the homolytic O–O cleavage of the persulfate leads to two sulfate radical anions, which effect α-amino C–H abstraction followed by single-electron oxidation to generate **1.A** (Scheme 1). An alternative productive pathway, given the oxidizing strength of sulfate radical anions [+2.4 V vs saturated calomel electrode (SCE)], could proceed through an initial oxidation of the substrate prior to α-amino C–H abstraction.^24^ The light-driven homolysis of persulfate has been previously reported.^20^ Unfortunately, increased loadings of K_2_S_2_O_8_ along with increased temperature, and/or longer reaction times did not improve the yield, leading instead to undesired reactivity such as over-oxidation. The use of riboflavin instead of riboflavin tetraacetate also led to diminished yields (entry 4). Riboflavin has been reported to undergo rapid photodegradation upon irradiation,^25,26^ which might contribute to the decreased yields. The acetylation of the ribose side chain (i.e., riboflavin → riboflavin tetraacetate) presumably prevents competing intramolecular hydrogen atom transfer (HAT) from the T_1_ excited state.^27^

In our studies, persulfate emerged as the superior oxidant as other oxidants led to lower yields (entry 5). Control studies confirmed the importance of both the oxidant and light source as the desired product was not observed when either component was excluded from the reaction mixture. Irradiation with 450 nm blue light-emitting diodes (LEDs, Penn PhD Photoreactor M2) in the absence of riboflavin tetraacetate led to recovered starting material (entry 7). Presumably, O–O bond homolysis does not occur with irradiation at 450 nm,^28^ which is also supported by our calculated UV–Vis spectrum of K_2_S_2_O_8_ (see the Supporting Information). Additionally, the use of one equivalent of riboflavin tetraacetate as the sole oxidant in the reaction led only to recovery of the starting material without any observed product formation. However, upon the combination of 5 mol % of riboflavin tetraacetate with persulfate, a 75% yield of aldehyde **3a** was obtained (entry 6). These experiments as well as the low yields obtained using K_2_S_2_O_8_ alone suggest the major pathway for product formation is not light- or thermal-driven O–O bond homolysis followed by SET and is driven by the combined action of the photocatalyst and oxidant. Furthermore, no correlation was observed between the excited state triplet energies of the photocatalysts examined and starting material consumption (see the Supporting Information for details). These experiments imply that triplet energy transfer pathways are not operating in these oxidative ring-opening reactions.

**Figure 3:**
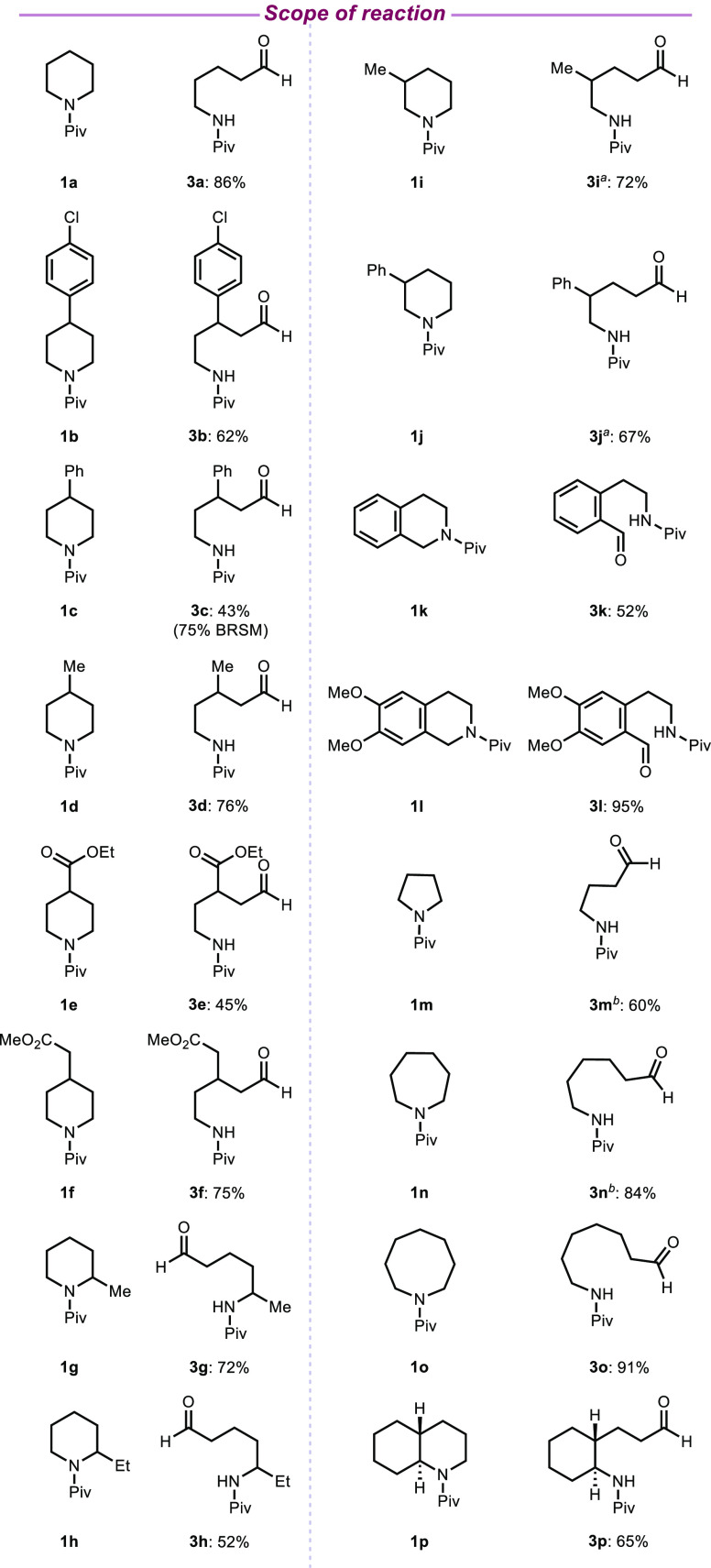
Cyclic amine scope. Only isolated yields are shown. Reaction conditions: cyclic amine (0.2 mmol), **2a** (5 mol %), K_2_S_2_O_8_ (3 equivalents), MeCN: H_2_O (1:1), and 2 h. All reactions were conducted using a Kessil lamp for irradiation. ^*a*^Major isolated constitutional isomer shown. ^*b*^Isolated yield of acid product: **3m**: 2.5%, **3n**: 13%).

Following our identification of an efficient set of conditions, we investigated the scope of the transition metal-free oxidative C–N cleavage protocol (Figure 3). Various substitution patterns on the piperidine ring were tolerated, providing access to the corresponding acyclic amines in moderate to good yields (43–95%). For example, 4-substituted piperidines led to β-substituted aliphatic aldehydes (**3b–3f**). Notably, piperidines containing benzylic sites that are susceptible to oxidation afforded the corresponding aldehydes in 62 and 43% yield, respectively. Piperidines bearing ester functional groups on the saturated azacycle backbone (e.g., **1e** and **1f**) also worked well. Complete positional selectivity was observed when 2-substituted piperidines (**1g**, **1h**) were subjected to the oxidative ring-opening protocol. Presumably, the selectivity that is observed is dictated by sterics in accordance with precedent.^9,10,29^ However, 3-substituted piperidines (**3i**, **3j**) gave a mixture of constitutional isomers.

Our transition metal-free oxidative protocol is not limited to piperidines. For example, skeletal diversification of the tetrahydroisoquinoline skeleton, which is present in a significant number of pharmaceuticals and natural products,^30,31^ is also possible. *N*-Pivaloyl-tetrahydroisoquinoline **1k** underwent oxidative ring opening to provide aldehyde **3k** in 52% yield. Notably, the C–N bond proximal to the arene ring was selectively cleaved. Furthermore, competing α-C–H abstraction was not observed despite the presence of two ether functional groups in **1l**; benzaldehyde **3l** was obtained in 95% yield. The α-arylation of ethers has been recently reported featuring open-shell alkyl radical intermediates, which add to heteroarenes (i.e., Minisci reaction).^32^ In these cases, the participating radicals were generated by hydrogen atom abstraction with persulfate.

Saturated aza-cycles of various ring sizes underwent oxidative ring opening to provide the corresponding aliphatic aldehydes in moderate to good yield (60–91%). However, in the case of **3m** and **3n**, the aldehyde groups underwent subsequent oxidation to the corresponding carboxylic acids, resulting in a mixture of products that were easily separated (see the Supporting Information for details).

In order to gain insight into the transition metal-free oxidative C–N cleavage protocol, we turned to density functional theory (DFT) and time-dependent DFT calculations. Following a series of computations to validate our models (see the Supporting Information), we selected riboflavin monoacetate (**2.A.m**, where “**m**” stands for monoacetate) as a model for riboflavin tetraacetate (**2.A**), which was used in our experiments. The calculations show that the formation of **(2.A.m)–[K_2_S_2_O_8_]** adduct is exergonic by ΔG of 5.9 kcal/mol. Since the coordination of **1a** to **(2.A.m)–[K_2_S_2_O_8_]** that leads to the **{(2.A.m)–[K_2_S_2_O_8_]}–(1a)** adduct is also exergonic (by 3.2 kcal/mol), we selected the {**(2.A.m)–[K_2_S_2_O_8_]}–(1a)** adduct as the photon-absorbing species, which aligns with precedent demonstrating prior coordination of persulfate to the photocatalyst.^33^ Here, we discuss only the energetically lowest conformers of every calculated structure and the corresponding Gibbs free energies unless otherwise stated—full computational details are described in the Supporting Information.

**Figure 4:**
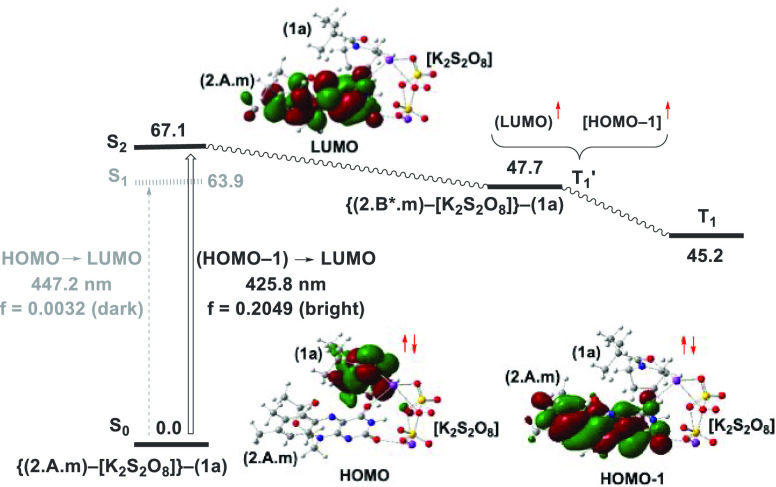
Schematic presentation of radiative generation of the S_2_ excited state of the riboflavin-persulfate-substrate adduct (**2.A.m**)–[K_2_S_2_O_8_]–**1a**. All energies are shown in kcal/mol.

**Figure 5:**
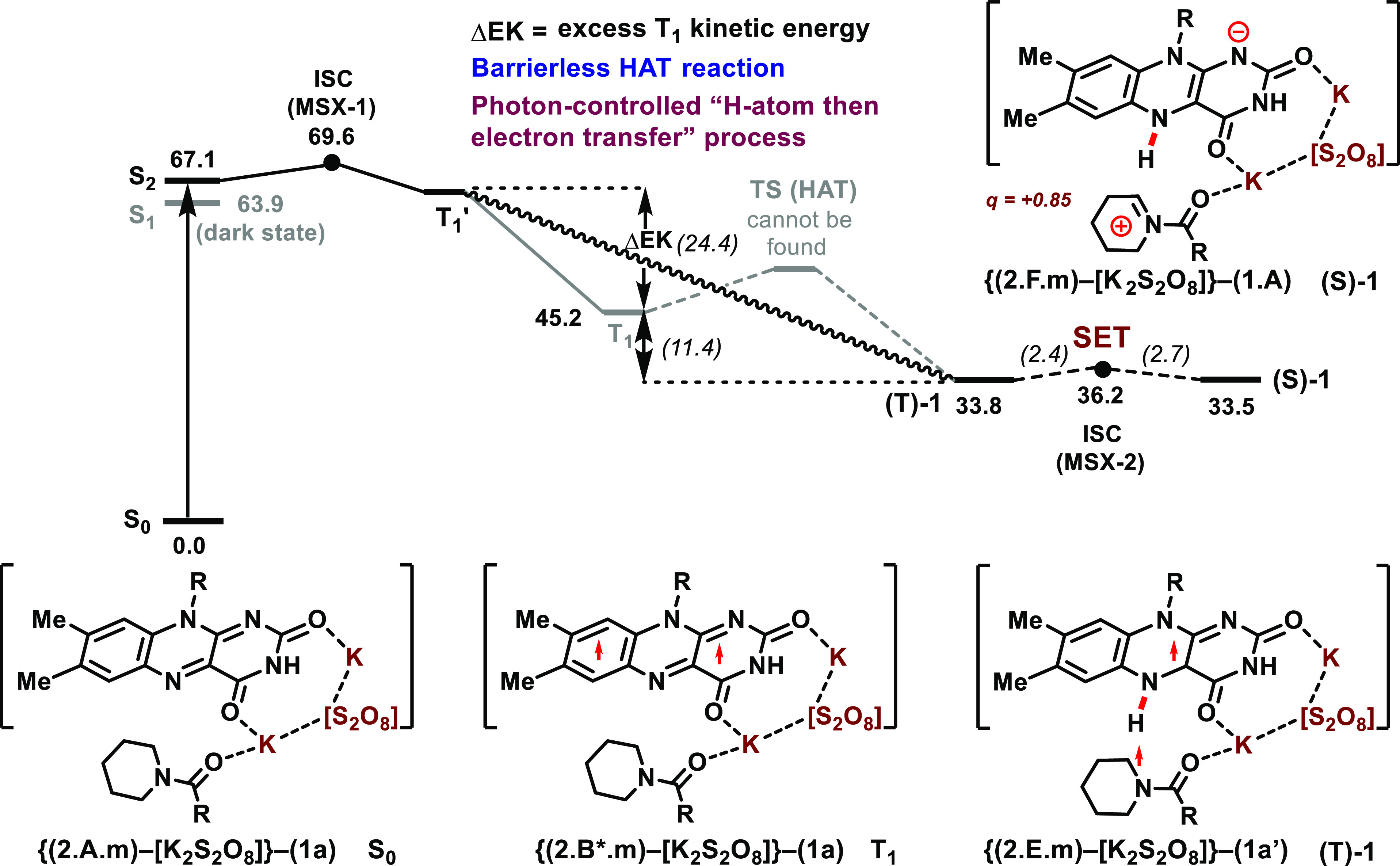
Computed reaction pathways for the oxidation of **1a** to **1.A**_._ All energies are Gibbs free energies listed in kcal/mol (energy differences are shown in parentheses).

**Figure 6:**
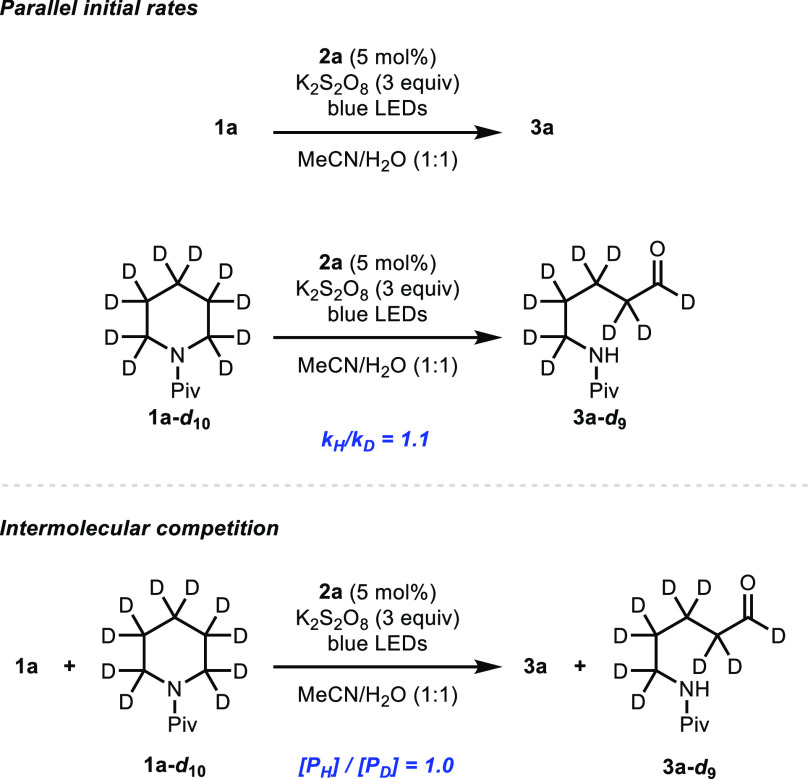
Initial rates comparison for **1a** and **1a-d_10_**, *k*_H_/*k*_D_ = 1.1. Intermolecular competition experiment (0.05 mmol **1a** + 0.05 mmol **1a-d_10_**), [*P*_H_]/[*P*_D_] = 1.0. See the Supporting Information for full details.

Our calculated reaction mechanism for the oxidation of **1a** is described in Figure 5. Irradiation of {**(2.A.m)–[K_2_S_2_O_8_]}–(1a)** with blue light (see the Supporting Information for the computed UV–Vis spectra of {**(2.A.m)–[K_2_S_2_O_8_]}–(1a)**, **(2.A.m)–[K_2_S_2_O_8_]**, and **2.A.m**) leads to excitation from the S_0_ state of **{(2.A.m)–[K_2_S_2_O_8_]}–(1a)** to its S_2_ bright state (see Figure 4). Its energetically lower-lying S_1_ state is a largely dark state that is unlikely to contribute to the reaction (see the Supporting Information). The bright S_0_/S_2_ transition is the (HOMO–1)–LUMO (i.e., π–π*) transition associated with the riboflavin fragment (see the Supporting Information). The excited S_2_ state of this system quenches to the optically dark triplet state T_1_ (through the T_1_′ state, see Figures 4 and 5) through the S_0_/T_1_ seam of crossing (MSX), which was determined to have a minimum point of 69.9 kcal/mol. Thus, the photoinitiated S_0_ → T_1_ transition is controlled by the minimum on the S_0_/T_1_ seam of crossing (MSX), which is energetically easily accessible at the wavelengths used in our experiments. The MSX serves as an interfacial “funnel”, which can also be described as intersystem crossing (ISC), from the optically bright singlets to the reactive T_1_ potential energy surface. The calculated 24.4 kcal/mol energy difference between the MSX point and the T_1_ minimum of complex **{(2.A.m)–[K_2_S_2_O_8_]}–(1a)** is internal energy that is available to complete the HAT from cyclic amine **1a** to the excited state riboflavin-persulfate complex. This process has no associated energy barrier and forms the triplet state intermediate **{(2.E.m)–[K_2_S_2_O_8_]}–(1a′)**, **(T)-1**. This conclusion is consistent with parallel reaction kinetic isotope effect (KIE) studies using Piv-protected piperidine-d_10_ (**1a-d_10_**). The results of the parallel experiments and intermolecular competition experiments (see the Supporting Information) do not support rate-limiting C–H bond cleavage (see Figure 6). Additionally, while we could not rule out radical chain pathways,^34^ a light on/off study revealed that any chain processes are short-lived, and irradiation is required for continued product formation (see the Supporting Information).

Calculations show that the formation of intermediate **(T)-1** is exergonic by 11.4 kcal/mol, relative to the optimized T_1_ state. Our computational analyses show that **(T)-1** possesses one less α-amino H-atom and has almost one full α-spin. Another unpaired α-spin is located in the riboflavin fragment. The subsequent single-electron transfer (SET) from the α-amino radical (i.e., **1a′**) to **{(2.E.m)–[K_2_S_2_O_8_]}** in **(T)-1** is almost thermoneutral. In the resulting singlet state intermediate **{(2.F.m)–[K_2_S_2_O_8_]}–(1.A)**, (i.e., **(S)-1**), the isoalloxazine is fully reduced and iminium ion **1.A** has formed. Extensive analyses show that the **(T)-1** → **(*S*)-1** transition occurs through the triplet-to-singlet seam of crossing (MSX), which has a minimum point located only ∼2.4 kcal/mol higher than **(T)-1**; therefore, the SET in **(T)-1** is a facile process and the **(T)-1**, **(*S*)-1** transition is exergonic by only 0.3 kcal/mol. Dissociation of iminium ion **1.A** from **(S)-1** requires only 10.4 kcal/mol and completes the formation of the iminium ion and intermediate **(S)-2** (see the Supporting Information for subsequent reactions from the **(S)-2** intermediate).

In summary, our calculations show that irradiation of a mixture of riboflavin tetraacetate, modeled as riboflavin monoacetate (**2.A.m**), potassium persulfate, and *N*-Piv-piperidine **1a** with blue light generates the triplet state intermediate {(2.B*.m)–[K_2_S_2_O_8_]}–(1a) (**T_1_**) through the S_0_/T_1_ seam of crossing (MSX), which is energetically accessible at the wavelengths used in our experiments. The iminium ion (**1.A**) generation from the T_1_ intermediate is a barrierless, photon-controlled process and occurs by a stepwise “H-atom *then* electron transfer” mechanism. This conclusion is the opposite of our initial hypothesis involving “electron *then* H-atom transfer” but is consistent with our experimental KIE studies.

### Copper-Mediated Deconstructive Functionalization

During our investigation of the photocatalytic oxidative ring opening of cyclic amines, we noted, in some cases, the subsequent oxidation of the generated aldehyde products to the corresponding carboxylic acids. Given the potential of these readily accessed carboxylic acids in subsequent derivatizations, we also explored complementary transition metal-mediated processes that would achieve oxidation of the aldehyde and provide access to alkyl carboxylic acids. Given the mild oxidation properties of copper salts (Cu(II)/Cu(I): −0.09 V vs SCE;^35^ Flavins: +1.67 V vs SCE;^36^*N*-acyl cyclic amines: +1.13 V vs SCE^9^), we envisaged a process in which a Cu(I) salt is oxidized by persulfate to Cu(II), forming a sulfate ion and a sulfate radical anion as byproducts. Under these conditions, oxidation of cyclic amine substrates through a HAT/SET process analogous to that depicted in Scheme 1 was expected. Upon formation of the open-chain aldehyde, a second oxidation to the carboxylic acid would be achieved through a Cu(II)/Cu(I) cycle.

**Figure 7:**
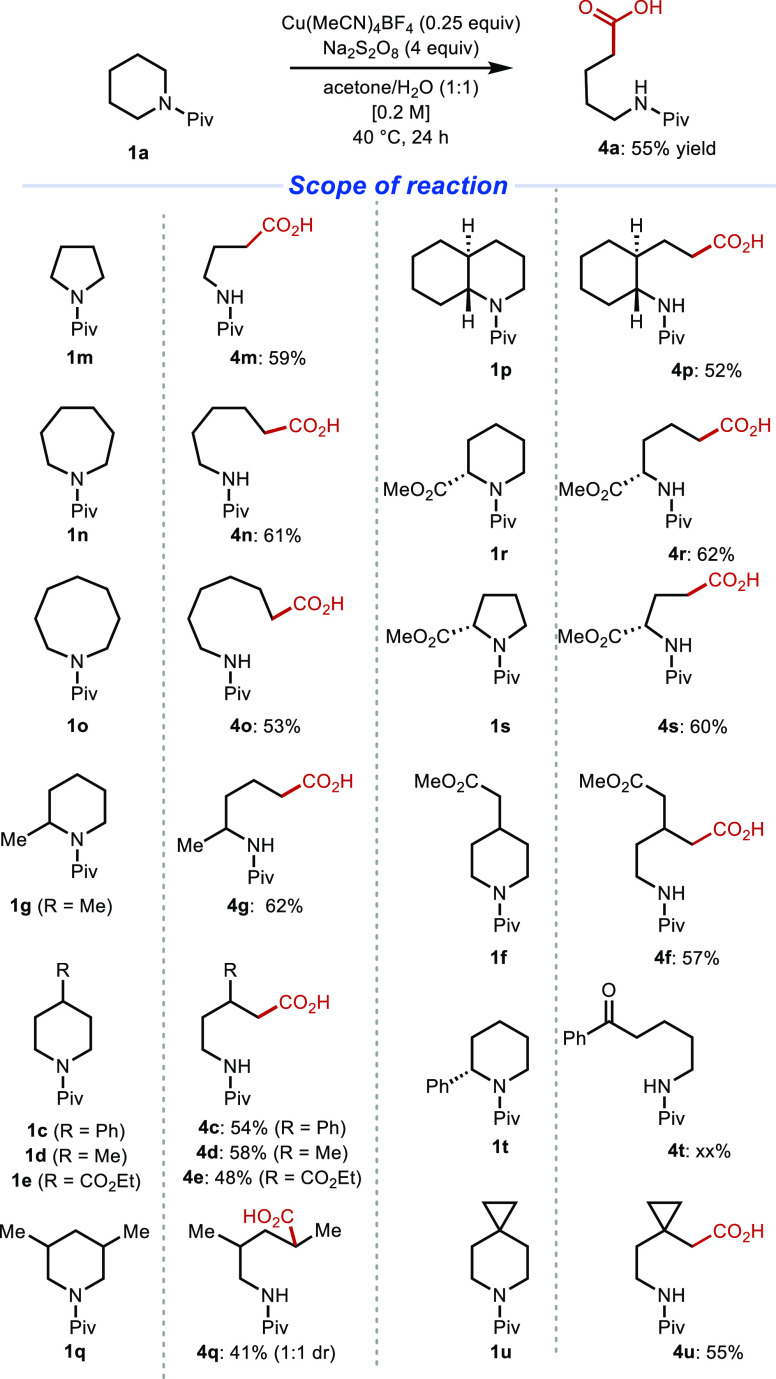
Copper-mediated oxidative ring opening of cyclic amines: reaction scope. Only isolated yields are shown. Reaction conditions: cyclic amine (0.2 mmol), Cu(MeCN)_4_BF_4_ (25 mol %), Na_2_S_2_O_8_ (4 equivalents), acetone/H_2_O (1:9), 24 h. ^*a*^Reaction performed in acetone/H_2_O (1:1). ^*b*^Reaction performed with 1 equivalent of Cu(MeCN)_4_BF_4_.

Following optimization of the reaction conditions (see the Supporting Information for details), *N*-Piv-piperidine was converted to carboxylic acid **4a** in 55% yield using 25 mol % Cu(MeCN)_4_BF_4_ and 4 equivalents of sodium persulfate in an acetone/water mixture. Cyclic amines of various ring sizes (**1m**–**10**, Figure 7) also underwent ring opening to form carboxylic acids with a range of chain lengths in moderate yield (53–61%). Piperidines bearing an α-methyl substituent (**1g**), analogous to our observations described above using riboflavin tetraacetate (Figure 3), exhibited selective ring opening on the less substituted side of the ring, giving the corresponding product in 49% yield. Other piperidine derivatives with various substitution patterns (**1c**–**1e**, **1q**) also participated in the ring opening. Esters and other substituents that possess activated benzylic hydrogens were tolerated and resulted in moderate yields (48–54%). The Cu(I)-mediated ring-opening reaction was also extended to other medicinally relevant saturated azacycles including perhydroquinoline (**1p**), pipecolic acid methyl ester (**1r**), and proline methyl ester (**1s**) in 52, 62, and 60% yield, respectively. Interestingly, a cyclic amine bearing an α-phenyl substituent resulted in the formation of a linear aryl ketone product (**4t**) along with a small amount (9%; 21%, based on recovered starting material; brsm) of a δ-keto acid product resulting from a second set of oxidations at the α-position of the amine (see the Supporting Information for details). Spiro-fused cyclopropyl piperidine **1u** was also converted to the corresponding carboxylic acid (**4u**; 55% yield) without competing opening of the cyclopropane, which may have occurred under more forcing conditions.

This mild oxidative ring-opening process can also be applied to the selective modification of amino acid sequences. Since polypeptides contain *N*-acylated cyclic amines in the form of proline residues, this method may provide a route for their modification.^37,38^ The copper-mediated oxidative ring-opening of a proline residue effectively transforms it to a glutamate residue through this direct core modification method. As is well recognized, the cyclic structure of proline imparts unique structural characteristics to peptide sequences because of its dihedral angle.^39−41^ As such, transforming a proline residue into a glutamate residue may not only impact the primary structure of a protein through a change in the amino acid sequence but could also have implications on larger-scale structures by influencing protein folding. While the transformation of a proline residue into glutamate has been reported previously,^12,17^ the existing methods require the use of catalysts that are either expensive or not commercially available, and therefore, these reaction conditions might be difficult to translate into industrial processes. Our method makes use of a cheap, commercially available catalyst that does not require difficult-to-access ligands.

**Figure 8:**
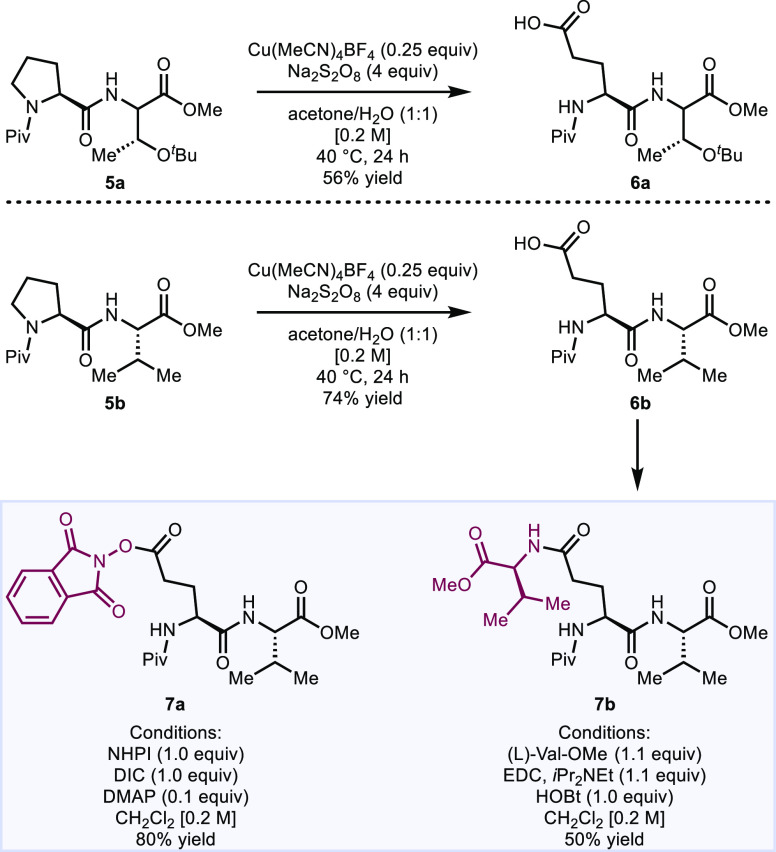
Peptide diversification. Only isolated yields are shown. See the Supporting Information for full experimental details.

We demonstrated the potential for modification of peptide sequences by performing a ring-opening oxidation on two dipeptides, Pro-Thr **5a** and Pro-Val **5b** (Figure 8). The resulting glutamate-bearing edited dipeptides were obtained in moderate yield (56 and 74%, respectively). Since the newly formed glutamate residue should serve as a reactive handle in esterification, amidation, and decarboxylation processes, we also briefly investigated subsequent transformations of the Glu-Val dipeptide. For example, esterification of the dipeptide with *N*-hydroxyphthalimide (NHPI) resulted in the corresponding NHPI ester (**7a**) in 80% yield. Given the emerging methods for engaging NHPI esters in cross-couplings,^42^**7a** may be used in a range of decarboxylative coupling reactions. The glutamic acid side chain could also be engaged in simple peptide couplings. For example, an amide coupling using valine led to branched peptide **7b** in 50% yield.

### Computational Study of the Cu(I)-Mediated Deconstructive C–H Functionalization of **1a** Using Sodium Persulfate (Na_2_S_2_O_8_) as an Oxidant

**Figure 9:**
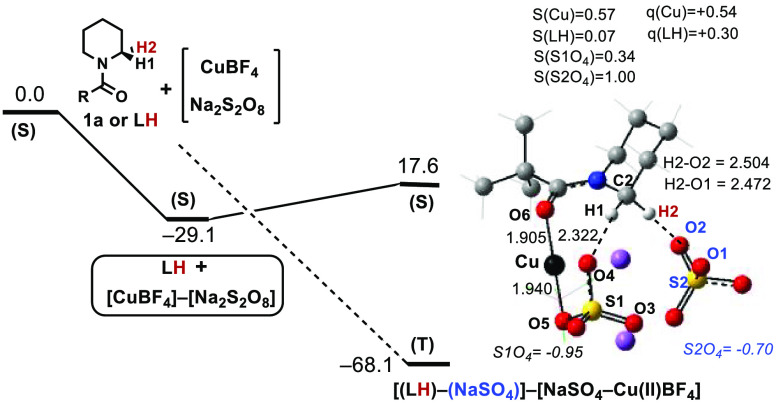
Computed reaction mechanism for the formation of intermediate [(**LH**)–(NaSO_4_)]–[NaSO_4_–Cu(II)BF_4_] upon reaction of CuBF_4_, **LH**, and sodium persulfate (Na_2_S_2_O_8_). Here, Gibbs free energies are in kcal/mol. Labels (S) and (T) represent the singlet and triplet electronic states, respectively. Spin densities, S(X), and Mulliken charges, q(X), are given in |e|. Geometries are in Å. BF_4_ anions are omitted for clarity.

In order to gain further insight into the Cu(I)-mediated oxidative opening of aliphatic amines derivatives (e.g., **1a**) to form ring-opened carboxylic acids, we launched DFT studies using CuBF_4_ to model Cu(I), *N*-Piv-piperidine **1a** as a substrate, and sodium persulfate (Na_2_S_2_O_8_) as an oxidant (see the Supporting Information for details). Similar to our previously reported Ag(I)-mediated C–C deconstructive fluorination of *N*-benzoylated cyclic amines using Selectfluor,^9,43^ one would expect either **1a** (represented as **LH** in Figure 9 to highlight and closely follow the anticipated H-atom transfer) or sodium persulfate coordination to the Cu(I)-center as a first step. Our calculations show that sodium persulfate coordination to CuBF_4_ is thermodynamically more favorable by 2.9 kcal/mol (26.2 vs 29.1 kcal/mol for the direct H-atom transfer) and leads to the singlet state adduct [CuBF_4_]–[Na_2_S_2_O_8_] (see the Supporting Information for more details).

**Figure 10:**
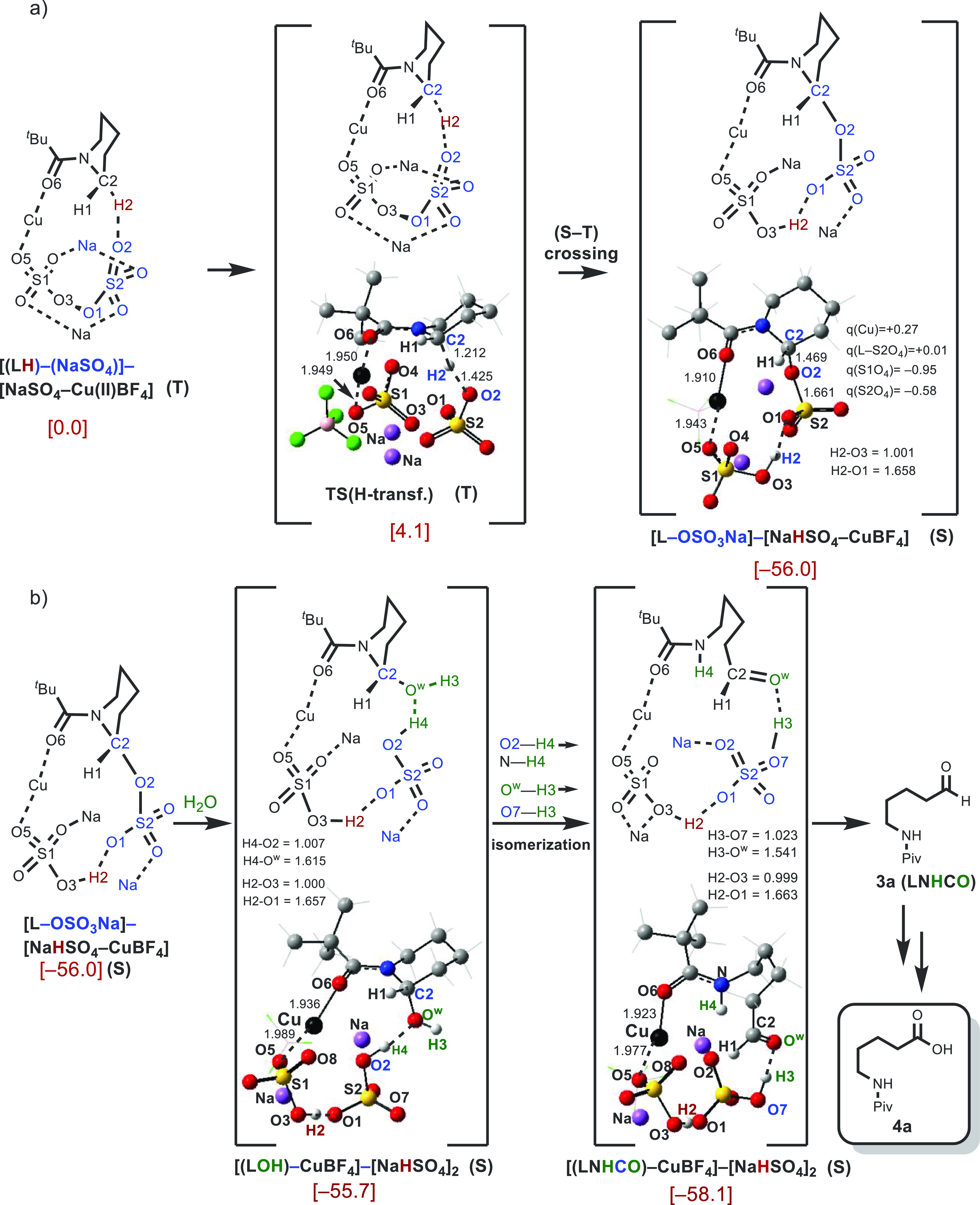
Computed ring-opening mechanism in intermediate [(**LH**)–(NaSO_4_)]–[NaSO_4_–Cu(II)BF_4_]. Gibbs free energies are reported in kcal/mol. Label (S) represents a singlet electronic state. Bond lengths are reported in Å. For simplicity, the BF_4_ fragment is omitted.

From singlet state adduct [CuBF_4_]–[Na_2_S_2_O_8_], SET could occur either prior to or after substrate coordination to the Cu(I)-center. In either case, SET from the Cu(I) center to sodium persulfate leads to the O–O bond cleavage and formation of sodium sulfate and a sulfate radical anion. Should the SET occur prior to substrate coordination to the Cu(I) center, the ring-opened aldehyde product (**3a** in Scheme 1) will form through a radical pathway initiated by H-atom abstraction by the sulfate radical anion (as shown in Scheme 1). However, our calculations show that the SET most likely occurs upon substrate coordination to the Cu(I) center since the formation of the triplet state Cu(II) intermediate [(**LH**)–(NaSO_4_)]–[NaSO_4_–Cu(II)BF_4_] is highly favored relative to the dissociation limit of (**LH**) + [CuBF_4_]–[Na_2_S_2_O_8_]. Interestingly, as can be seen in Figure 9, in the Cu(II) intermediate [(**LH**)–(NaSO_4_)]–[NaSO_4_–Cu(II)BF_4_], the S1O_4_ sulfate anion (left side of the structure, each SO_4_ unit here is denoted as S1O_4_ or S2O_4_ for distinction) is coordinated to the Cu(II)-center (Cu–O5 bond length = 1.940 Å). The second sulfate anion (S2O_4_) is weakly associated with the Cu-coordinated substrate (H2–O2 = 2.504 Å and H2–O1 = 2.472 Å). These sulfate anions are bridged by the two sodium cations. Since the SET is expected to be very fast and occur through the singlet-to-triplet seam of crossing for the [CuBF_4_]–[Na_2_S_2_O_8_] complex, it should not impact the rate of reaction or reaction outcome. Therefore, here, an in-depth analysis of this path was not conducted.^44,45^ The next step of the reaction, H-atom abstraction from the C2-position of the *N*-acylated piperidine substrate by the S2O_4_ sulfate anion, is illustrated in Figure 10a. This step is a two-state reactivity event (i.e., starts from a triplet state prereaction complex and results in a singlet state product) that has a small free energy barrier of 4.1 kcal/mol at the triplet state transition state **TS(H-transf.)**. The net process is highly exergonic (by 56.0 kcal/mol). Interestingly, intrinsic reaction coordinate (IRC) calculations show that while this process begins with abstraction of a H-atom from C2 by the O2-atom of the S2O_4_ sulfate anion at **TS(H-transf.)**, it culminates in a product where the abstracted H-atom has been transferred to the second sulfate anion (i.e., S1O_4_) and the O2-atom of the S2O_4_ sulfate anion is coordinated to the piperidine ring at the C2-position. Overall, this H-atom abstraction event leads to the formation of a singlet state product [L-OSO_3_Na]–[NaHSO_4_–CuBF_4_], which is a complex of the [L-OSO_3_Na] and [NaHSO_4_–CuBF_4_] fragments. Charge density analyses show that the [L-OSO_3_Na] unit possesses an overall +0.57 |e| charge.

Calculations show that in the presence of water molecules (see Figure 10b), intermediate [L-OSO_3_Na]–[NaHSO_4_–CuBF_4_] is in equilibrium with the hemiaminal (i.e., [(LOH)–CuBF_4_]–[NaHSO_4_]_2_). The hemiaminal is the product of H–O^w^H hydrolysis of the L–OSO_3_Na bond. Multiple proton-shuttles occur between the N-center of the substrate and HSO_4_^–^ unit, as well as between the LOH and HSO_4_^–^ units, which leads to formation of intermediate [(**LNHCO**)–CuBF_4_]–[NaHSO_4_]_2_. Calculations show that [(**LNHCO**)–CuBF_4_]–[NaHSO_4_]_2_ is only 2.4 kcal/mol lower in free energy than the [(LOH)–CuBF_4_]–[NaHSO_4_]_2_ complex. At this time, we cannot rule out the formation of [(**LNHCO**)–CuBF_4_]–[NaHSO_4_]_2_ through outer-sphere protonation–deprotonation by other molecules of water from the solvent.

Dissociation of **LNHCO** from [(**LNHCO**)–CuBF_4_]–[NaHSO_4_]_2_ leads to the final product. The computational data presented above shows that the overall reaction (**LH**) + CuBF_4_ + Na_2_S_2_O_8_ → (**LNHCO**) + [CuBF_4_]–[NaHSO_4_]_2_ is highly exergonic (by 126.2 kcal/mol) and will proceed spontaneously without a significant energy barrier. Under the oxidative reaction conditions, it is likely that the resulting aldehyde (**3a**) is oxidized to the corresponding carboxylic acid **4a** (see Figure 10B).^46^

### Ring-Opening Minisci Reaction

With access to various carboxylic acid derivatives through our oxidative ring-opening process, we envisioned engaging them in one-pot coupling reactions. To this end, we turned toward Csp^3^–Csp^2^ bond formation using a radical decarboxylation process and addition of the resulting alkyl radical to a heterocycle acceptor. We have previously demonstrated access to a proposed primary alkyl radical through a series of oxidations with a Ag(I) salt and persulfate oxidant.^10^ Therefore, we hypothesized that this radical might engage a coupling partner in carbon–carbon bond forming processes. This type of Csp^3^–Csp^2^ bond formation has recently been demonstrated in radical additions to quinones through a silver-mediated deconstructive process,^29^ as well as an analogous Minisci process.^100^ Since the conditions for the deconstructive process that lead to an alkyl radical are somewhat acidic (as a result of the formation of hydrogen sulfate ions and carbonic acid), we hypothesized that these conditions would be amenable to Minisci-type reactions,^20^ which proceed more favorably upon protonation of a heteroarene acceptor.

**Figure 11:**
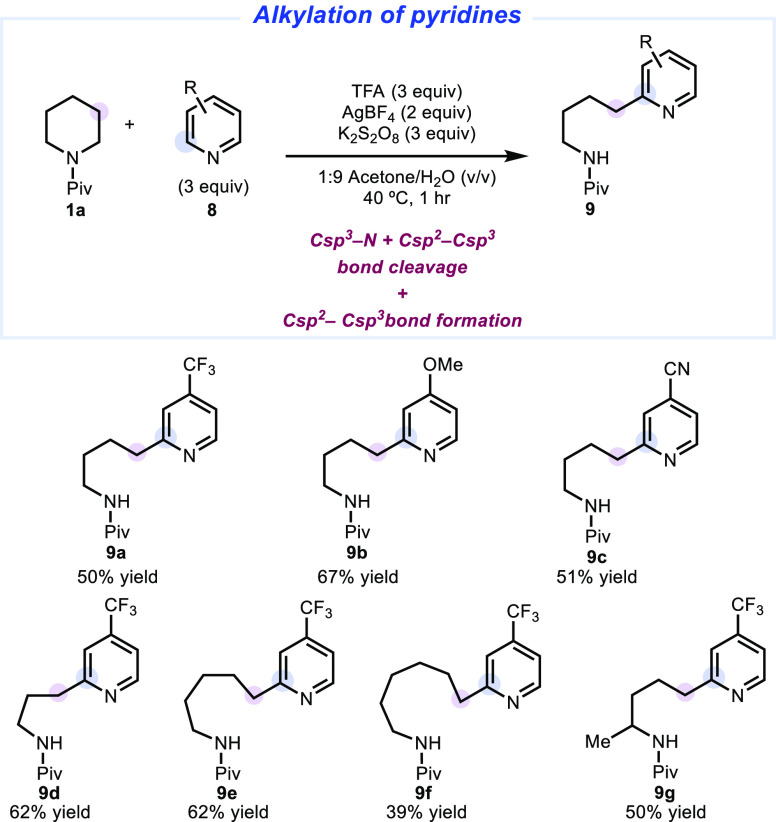
Deconstructive Minisci reaction scope. Only isolated yields are shown. See the Supporting Information for full experimental details.

Following optimization of the reaction conditions, treatment of Piv-protected piperidine **1a** with the Ag(I)/persulfate conditions and 4-trifluoromethylpyridine under acidic conditions (trifluoroacetic acid) afforded the corresponding amino-alkyl substituted pyridine in 50% yield (Figure 11). Pyridines bearing electron donating/withdrawing substituents were also competent in this coupling reaction, forming the desired products (**9a**–**9c**) in moderate yet useful yields (50–67%). We next subjected cyclic amines of various ring sizes (**9d**–**9f**) to the same conditions, again providing the alkylation products in moderate yield (39–62%). A 2-methyl substituted piperidine derivative also underwent the expected ring-opening selectively away from the substituted side of the ring (**9g**).

### Autocyclization Using Cu(II) Oxidation

**Figure 12:**
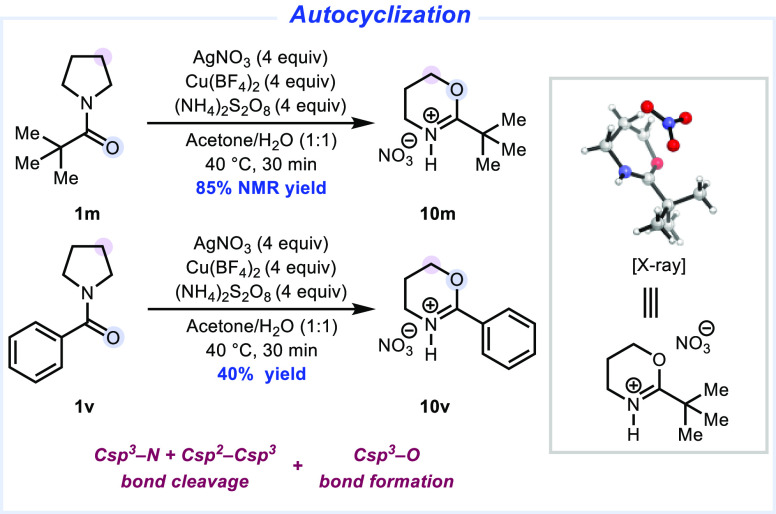
Autocyclization of cyclic amines. Isolated yields are shown unless otherwise noted. See the Supporting Information for full experimental details and crystallographic data.

Finally, we also investigated the interception of the proposed alkyl radical intermediate with other transition metals. Particularly, since De La Mare and coworkers^47,48^ previously reported the formation of alkenes from alkyl radicals through an oxidation by Cu(II) followed by an elimination, we wondered whether we could gain access to terminal olefins from the primary radical generated through the deconstructive process. However, upon the addition of a Cu(II) source to the Ag(I)/persulfate reagents for the deconstructive functionalization of Piv-protected pyrrolidine, we did not detect any of the expected alkene product. Instead, we observed the formation of an oxazine in 85% yield (Figure 12). This heterocycle may arise from oxidation of the alkyl radical by Cu(II) and intramolecular nucleophilic trapping by the amide carbonyl oxygen instead of elimination to generate an olefin. The structure of **10m** was unambiguously confirmed by X-ray crystallography. The formation of these oxazine heterocycles has previously been reported from the autocyclization of γ-bromo alkyl amides.^49^ We extended the transformation to the ring opening and subsequent cyclization of *N*-Bz-pyrrolidine (**1v**) to provide phenyl-substituted oxazine **10v** in 40% yield. The transformation of pyrrolidines to oxazines represents an effective strategy to access new chemical space in medicinal chemistry.

**Figure 13:**
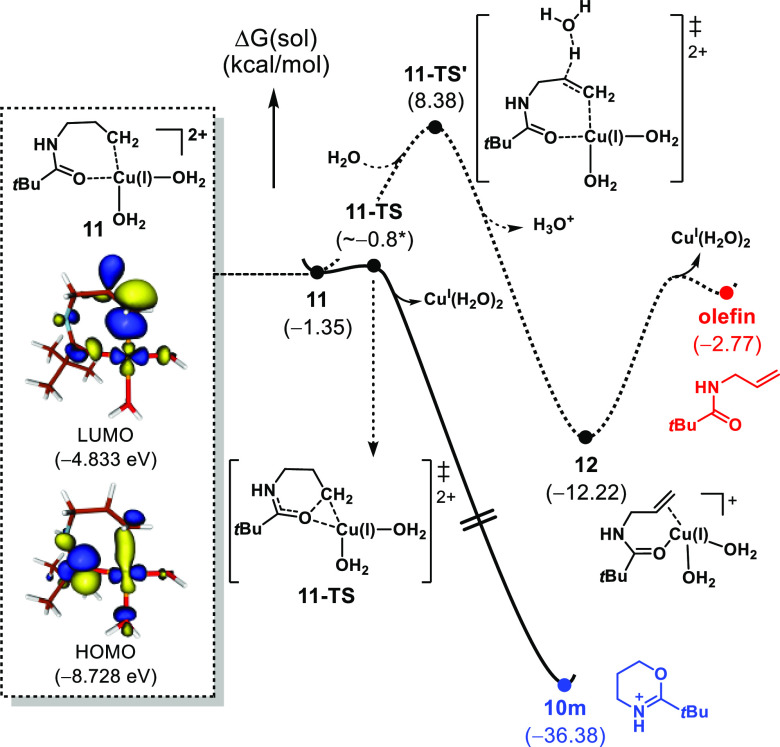
Free energy profile for the oxazine formation by Cu(II). See the Supporting Information for full computational details.

Since the formation of the oxazines did not align with our initial hypothesis, we embarked upon an examination of the mechanism for their formation using DFT calculations, as summarized in Figure 13. We have previously investigated a mechanism for the generation of an alkyl radical from a cyclic amine using Ag(I) and persulfate.^10,43^ Here, we propose that the alkyl radical is similarly formed prior to interacting with Cu(II) to form complex **11**, although the exact details continue to be investigated. While the combination of a carbon-centered radical with Cu(II) is often depicted as a Cu(III) intermediate,^50,51^ we recognize that these types of copper complexes may have an electronic structure that reflects a Cu(I) center, in which a formal reduction of Cu(II) occurs.^52^ Our calculations suggest that deprotonation to form the alkene has an associated barrier of 9.7 kcal/mol, while the competing intramolecular cyclization is essentially barrierless. Even though **11** is hydrated and the concentration of H_2_O is ∼500 times higher than the copper complex, calculations suggest that the intrinsic reaction rate of cyclization should be about 5 × 10^6^ times faster than deprotonation (see the Supporting Information for details). Therefore, the cyclization should be the dominant pathway. Indeed, a screen of various copper salts, which would otherwise lead to products such as olefination and halogenation of primary alkyl radicals,^48^ revealed that only the linear aldehyde **3m**, carboxylic acid **4m**, and cyclization product **10m** could be obtained (see the Supporting Information for details). The rapid cyclization can also be rationalized on the basis of the frontier molecular orbitals of **11**. While the orbitals of **11** are all interacting, there is a prominent interaction between the HOMO and LUMO orbitals that informs the observed cyclization to oxepine **10m**. As shown in Figure 13, the shape of the nucleophilic HOMO on the amidyl oxygen and the low energy LUMO on the primary alkyl in **11** are reasonably well matched for the favorable intramolecular HOMO–LUMO interaction.^53^

## Conclusions

In summary, we have developed a range of deconstructive functionalizations of cyclic aliphatic amines to achieve skeletal diversification that builds on our previous reports.^9−11^ We developed two methods for ring-opening transformations, achieving an organo-photocatalytic formation of linear aldehydes with riboflavin tetraacetate and a Cu(I)-mediated oxidation to access amine derivatives bearing carboxylic acid groups. We have also reported a combined organo-photocatalytic/transition metal-mediated oxidative ring-opening procedure for high-yielding formation of carboxylic acids (see the Supporting Information). Our calculations of the photoinduced oxidation of piperidines by riboflavin tetraacetate suggest that the oxidation is initiated by a photon-driven HAT.

We have also developed a ring-opening C–C bond formation process that takes advantage of a decarboxylative Csp^3^–Csp^2^ Minisci coupling reaction. While our lab previously reported the formation of alkyl halides through a similar transformation that could serve as precursors for C–C bond formation,^10^ the Minisci process reported here provides direct access to value-added C–C coupled products. A second study aimed at a one-pot, multimetallic, decarboxylative transformation led to the formation of oxazines. Additional investigations to expand the scope of oxazine formation from cyclic amines are ongoing in our lab.

In the studies presented here, persulfate emerged as a versatile oxidant that could be tuned by pairing it with various transition metals and organic photocatalysts to selectively oxidize various substrates. The multiple means by which the peroxy bond can be homolyzed, in combination with the oxidation potential and H-atom abstraction ability of the sulfate radical anion, allow for many different oxidative processes. The versatility of cyclic amines as substrates for late-stage diversification as reported here rests on their ability to serve as latent alkyl radicals. These studies, as well as previous reports from our laboratories and others, demonstrate high potential for skeletal modification of cyclic amines by the generation of an alkyl radical through oxidative ring opening. Other pathways for structural diversification of cyclic aliphatic amines are currently under investigation in our lab.

## Supporting Information Available

Experimental procedures, computational details, and compound characterization (PDF)
