# Supplementary material for: Photo- and Metal-Mediated Deconstructive Approaches to Cyclic Aliphatic Amine Diversification
Source: J Am Chem Soc. 2023 May 12;145(20):11245–57. doi: 10.1021/jacs.3c01318 (PMC10214453; doi:10.1021/jacs.3c01318)
Supplement: Supplementary file 1 — ja3c01318_si_001.pdf [file ja3c01318_si_001.pdf]

Photo- and Metal-Mediated Deconstructive Approaches to Cyclic Aliphatic Amine Diversification

David M. Soro,[||,◊] Jose B. Roque,[||,◊] Jonas W. Rackl,[||] Bohyun Park,[‡,⊥] Stefan Payer,[||] Yuan Shi,[§] J. Craig Ruble,[§] Alexey L. Kaledin,[†] Mu-Hyun Baik,[*,‡,⊥] Djamaladdin G. Musaev,[*,†] Richmond Sarpong[||*]

[||] Department of Chemistry, University of California, Berkeley, CA 94720, United States
[†]Cherry L. Emerson Center for Scientific Computation, and Department of Chemistry, Emory University, 1521 Dickey Drive, Atlanta, GA, 30322, United States
[‡]Department of Chemistry, Korea Advanced Institute of Science and Technology (KAIST), Daejeon 34141, Korea

[⊥]Center for Catalytic Hydrocarbon Functionalizations, Institute for Basic Science (IBS), Daejeon 34141, Korea

[§]Discovery Chemistry Research and Technologies, Eli Lilly and Company, Indianapolis, Indiana 46285, United States

Table of Contents

1. General Information....S4

1.1 Solvents and Reagents....S4

1.2 Experimental Procedures....S4

1.3 Analytical Instrumentation....S4

2. Experimental Procedures....S5

2.1 Preparation of N-Protected Cyclic Amines....S5

2.2 Preparation of Photocatalyst **2a** (Riboflavin Tetraacetate, RFTA) S11

2.3 Experimental Setup of Photoreactor....S12

2.4 Photochemical Method Optimization....S13

2.5 Experiments to Probe Triplet Energy Transfer Mechanism....S19

2.6 Kinetics Experiments....S20

2.7 Procedure for Riboflavin Tetraacetate-Mediated Ring Opening....S22

2.8 Procedure for Copper-Mediated Ring Opening....S30

2.9 Procedures for Peptide Diversification....S37

2.10 Procedure for Deconstructive Minisci Reaction....S40

2.11 Procedure for Autocyclization Reaction....S44

2.12 Counterion Effect for Autocyclization Reaction....S45

3. Computational Details....S46

3.1 General Computational Considerations for Photo- and Copper-Mediated Reactions....S46

3.2 Critical Interactions in **{(2.A.m)–[$K_2S_2O_8$]}** and **{(2.A.m)–[$K_2S_2O_8$]}–(1a)**....S47

3.3 Verification of Photocatalyst Model....S48

3.4 Computed Reaction Mechanism for Photo-Mediated Reaction....S52

3.5 Optimized Structure Energy Components for Photo-Mediated Reaction....S53

3.6 Optimized Structures and Energy Components for Copper-Mediated Reaction....S54

3.7 General Computational Considerations for Computations in Figure 13....S55

3.8 Reaction Rate Comparison Between **11-TS** and **11-TS'**....S56

3.9 Energy Components for Optimized Structures in Figure 13....S57

4. X-ray Crystallographic Data....S58

4.1 X-ray Analysis of 10m....S58

5. NMR Spectral Data....S63

6. Computational Coordinates and Vibrational Frequencies..S126

7. References..S148

1. General Considerations

1.1 Solvents and Reagents

Tetrahydrofuran (THF) and triethylamine (Et_3N) were sparged with argon and dried by passing through alumina columns using argon in a Glass Contour solvent purification system. Dichloromethane (CH_2Cl_2) was freshly distilled over calcium hydride under a N_2 atmosphere prior to each use. DMF was purchased in Aldrich Sure/Seal™ bottles. N-Boc-piperidine (**1a.a**) was obtained from Aldrich and used as received. Reagents were purchased from commercial vendors as follows: Riboflavin (RTA, 98%) was purchased from Alfa Aesar. Silver nitrate ($AgNO_3$, ≥99%) was purchased from Sigma-Aldrich. Ammonium persulfate ($(NH_4)_2S_2O_8$, ACS Reagent) was purchased from J. T. Baker Chemicals, potassium persulfate ($K_2S_2O_8$, ACS Reagent) was purchased from Fisher Scientific, and sodium persulfate ($Na_2S_2O_8$, 98+%) was purchased from Acros Organics. Acetonitrile (HPLC), acetone (HPLC) and water (HPLC) were purchased from Fisher Scientific.

1.2 Experimental Procedures

Unless otherwise noted in the experimental procedures, reactions were carried out in flame or oven-dried glassware under a positive pressure of N_2 in anhydrous solvents using standard Schlenk techniques. Reaction temperatures above room temperature (22–23 °C) were controlled by an IKA® temperature modulator and monitored using liquid-in-glass thermometers. Reaction progress was monitored using a combination of LC/MS analysis (using a Shimadzu LCMS-2020 (UFLC) equipped with the LC-20AD solvent delivery system, a SPD-20AV prominence UV/Vis detector (SPD-M20A Photo Diode Array), and a Thermo Scientific Hypersil GOLD HPLC column (5 µm particle size, 4.6 × 50 mm)), and thin-layer chromatography (TLC) on Macherey-Nagel (MN) silica gel plates (glass backed, 60 Å, 0.25 mm thickness, UV254 manganese-activated zinc silicate fluorescence indicator). Visualization of the developed plates was performed under UV-light (254 nm) irradiation, and then gently heated with *p*-anisaldehyde or cerium ammonium molybdate (CAM) stain. Flash column chromatography was performed with either glass columns using Silicycle silica gel (40–63 µm particle size) or using a Yamazen Smart Flash EPCLC W-Prep 2XY (dual channel) automated flash chromatography system on prefilled, premium, universal columns using ACS grade solvents. Preparative thin layer chromatography was performed on SiliCycle Siliaplates (glass backed, extra hard layer, 60 Å, 250 µm thickness, F254 indicator).

1.3 Analytical Instrumentation

^{1}H NMR and ^{13}C NMR data were recorded on Bruker AVQ-400, AVB-400, AV-600 and AV-700 spectrometers using $CDCl_3$ as solvent, typically at 20–23 °C. Chemical shifts (δ) are reported in ppm relative to the residual solvent signal (δ 7.26 for ^{1}H NMR, δ 77.16 for ^{13}C NMR in $CDCl_3$). ^{19}F NMR spectra were acquired on an AVQ-400 spectrometer and internally referenced to $CFCl_3$ (δ 0.00). Data for ^{1}H and ^{13}C spectroscopy are reported as follows; chemical shift (δ ppm), multiplicity (s = singlet, d = doublet, t = triplet, q = quartet, quint = quintet, hept = heptet, m = multiplet, br = broad), coupling constant (Hz), integration. Melting points were determined using a MEL-TEMP™ apparatus and are uncorrected. High-resolution mass spectra (HRMS) were analyzed as MeOH solutions (30–50 µM) using a Finnigan LTQ FT mass spectrometer (Thermo). Solutions were injected directly into the ion source via syringe pump with 5 uL/min flow rate. Xcalibur software (version 2.0.7, Thermo) was used for both spectra acquisition and data analysis.

2. Experimental Procedures

2.1 Preparation of *N*-Protected Cyclic Amines

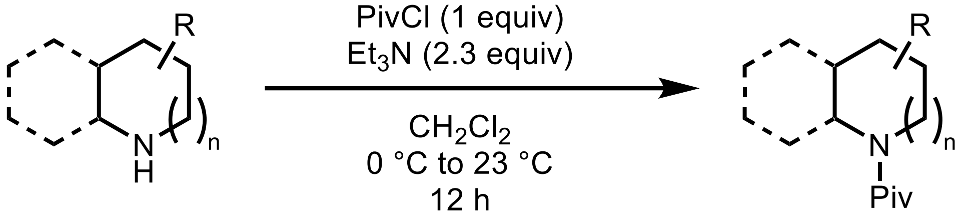

A 25 mL round-bottomed flask was charged with a solution of the cyclic amine (500 mg, 1.0 equiv) and Et_3N (2.3 equiv) in CH_2Cl_2 (20 mL) and cooled to 0 °C. Pivaloyl chloride (1.1 equiv) was added dropwise. The resulting mixture was allowed to warm to room temperature and stirred for 12 h, after which the reaction mixture was quenched with HCl (1M, 5 mL). The phases were separated, and the aqueous phase was extracted with CH_2Cl_2 (3 x 5–10 mL). The combined organic layers were washed with brine (5–10 mL), dried over Na_2SO_4, filtered, and concentrated under reduced pressure.

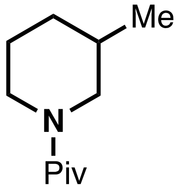

1i

2,2-Dimethyl-1-(3-methylpiperidin-1-yl)propan-1-one (**1i**): The title compound was prepared according to the general procedure using 3-methylpiperidine to give **1i** (644mg, 81%) as a colorless to slight yellowish liquid.
^{1}H NMR (400 MHz, $CDCl_3$): δ 4.28 (dd, *J* = 30.5, 13.2 Hz, 2H), 2.72 (t, *J* = 12.1 Hz, 1H), 2.41 (t, *J* = 12.0 Hz, 1H), 1.9 – 1.74 (m, 1H), 1.73 – 1.61 (m, 1H), 1.61 – 1.37 (m, 2H), 1.27 (s, 9H), 1.13 – 1.05 (m, 1H), 0.89 (d, *J* = 6.6 Hz, 3H);
^{13}C NMR (126 MHz, $CDCl_3$) δ 176.2, 38.9, 38.5, 33.44, 31.7, 28.6, 27.2, 25.8, 19.2;
HRMS (ESI): Calc'd for $C_{11}H_{22}NO$ $[M+H]^+$: 184.1696, found: 186.1695.

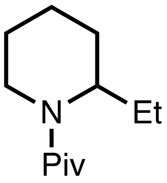

1h

1-(2-ethylpiperidin-1-yl)-2,2-dimethylpropan-1-one (**1h**): The title compound was prepared according to the general procedure using 2-ethylpiperidine to give **1h** (854 mg, 98%) as a slight yellowish liquid.
^{1}H NMR (400 MHz, $CDCl_3$): δ 4.67 (s, 1H), 4.01 (s, 1H), 2.98 (s, 1H), 1.70 – 1.51 (m, 6H), 1.49 – 1.31 (m, 2H), 1.25 (s, 9H), 0.83 (t, *J* = 7.3 Hz, 3H);
^{13}C NMR (126 MHz, $CDCl_3$) δ 176.6, 39.0, 28.7, 27.3, 26.3, 22.5, 19.2, 10.7

(*Two ^{13}C signals are missing due to peak broadening*);
HRMS (ESI): Calc'd for $C_{12}H_{24}NO$ $[M+H]^+$: 198.1852, found: 198.1852.

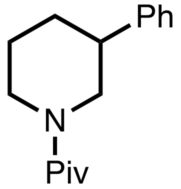

1j

2,2-dimethyl-1-(3-phenylpiperidin-1-yl)propan-1-one (**1j**): The title compound was prepared according to the general procedure using 3-phenylpiperidine (250 mg, 1.55 mmol, 1 equiv) to give **1j** (378 mg, 99%) as a slight yellowish liquid.
^{1}H NMR (400 MHz, $CDCl_3$): δ 7.37 – 7.29 (m, 2H), 7.27 – 7.20 (m, 3H), 4.52 (t, *J* = 15.3 Hz, 2H), 2.85 – 2.62 (m, 3H), 2.11 – 2.00 (m, 1H), 1.88 – 1.77 (m, 1H), 1.76 – 1.67 (m, 1H), 1.66 – 1.53 (m, 1H), 1.30 (s, 9H);
^{13}C NMR (126 MHz, $CDCl_3$) δ 176.4, 143.4, 128.8, 127.2, 126.9, 43.4, 39.00, 32.1, 28.6, 26.1 (*Two ^{13}C signals are missing due to peak broadening*);
HRMS (ESI): Calc'd for $C_{16}H_{24}NO$ $[M+H]^+$: 246.1852, found: 246.1852.

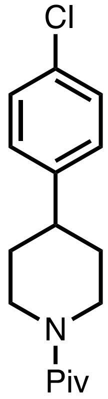

1b

1-(4-(4-chlorophenyl)piperidin-1-yl)-2,2-dimethylpropan-1-one (**1b**): The title compound was prepared according to the general procedure using 4-(4-chlorophenyl)piperidine (250 mg, 1.28 mmol, 1 equiv) to give **1b** (300 mg, 84%) as a slight brownish-yellowish liquid.
^{1}H NMR (400 MHz, $CDCl_3$): δ 7.32 – 7.24 (m, 3H), 7.17 – 7.09 (m, 2H), 4.57 (d, *J* = 13.3 Hz, 2H), 2.86 (t, *J* = 13.0 Hz, 2H), 2.74 (tt, *J* = 12.1, 3.8 Hz, 1H), 1.87 (d, *J* = 13.5 Hz, 2H), 1.66 – 1.51 (m, 2H), 1.31 (s, 9H);
^{13}C NMR (126 MHz, $CDCl_3$) δ 176.5, 149.6, 129.2, 125.5, 117.8, 49.7, 45.0, 28.5. (*Three ^{13}C signals are missing due to peak broadening*);
HRMS (ESI): Calc'd for $C_{16}H_{23}NOCl$ $[M+H]^+$: 280.1463, found: 280.1461.

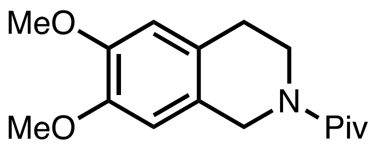

1l

1-(6,7-dimethoxy-3,4-dihydroisoquinolin-2(1*H*)-yl)-2,2-dimethylpropan-1-one (**1l**): The title compound was prepared according to the general procedure using 6,7-dimethoxy-1,2,3,4-tetrahydroisoquinoline (250 mg, 1.55 mmol, 1 equiv) to give **1l** (378 mg, 99%) as a slight yellowish liquid.
^{1}H NMR (500 MHz, $CDCl_3$) δ 6.61 (s, 1H), 6.59 (s, 1H), 4.67 (s, 1H), 4.09 – 3.68 (m, 8H), 2.80 (t, J = 6.1 Hz, 2H), 1.31 (s, 9H).
^{13}C NMR (126 MHz, $CDCl_3$) δ 176.8, 147.9, 147.8, 126.3, 125.5, 111.5, 109.2, 56.1, 56.1, 47.2, 43.6, 38.9, 28.6, 28.5.
HRMS (ESI): Calc'd for $C_{16}H_{24}NO_3$ $[M+H]^+$: 278.1751, found: 278.1750.

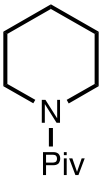

1a

2,2-dimethyl-1-(piperidin-1-yl)propan-1-one (**1b**) was prepared according to a published procedure.[1] Spectral data were in full agreement with the reported literature values.

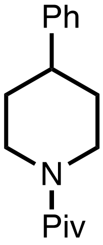

1c

2,2-dimethyl-1-(4-phenylpiperidin-1-yl)propan-1-one (**1e**) was prepared according to a published procedure.[2] Spectral data were in full agreement with the reported literature values.

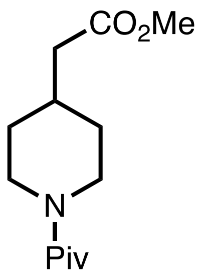

1f

Methyl 2-(1-pivaloylpiperidin-4-yl)acetate (**1f**) was prepared according to a published procedure. Spectral data were in full agreement with the reported literature values.[3]

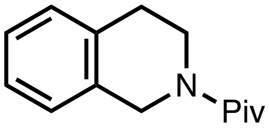

1k

1-(3,4-dihydroisoquinolin-2(1H)-yl)-2,2-dimethylpropan-1-one (**1k**) was prepared according to a published procedure.[4] Spectral data were in full agreement with the reported literature values.

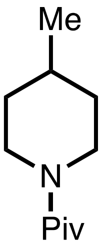

1d

2,2-dimethyl-1-(4-methylpiperidin-1-yl)propan-1-one (**1d**) was prepared according to a published procedure.[3] Spectral data were in full agreement with the reported literature values.

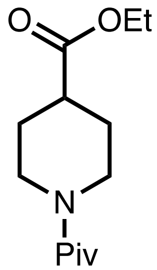

1e

Ethyl 1-pivaloylpiperidine-4-carboxylate (**1e**) was prepared according to a published procedure.[3] Spectral data were in full agreement with the reported literature values.

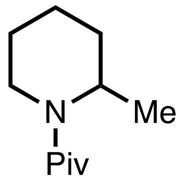

1g

2,2-dimethyl-1-(2-methylpiperidin-1-yl)propan-1-one (**1g**) was prepared according to a published procedure.[3] Spectral data were in full agreement with the reported literature values.

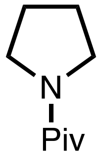

1m

2,2-dimethyl-1-(pyrrolidin-1-yl)propan-1-one (**1m**) was prepared according to a published procedure.[5] Spectral data were in full agreement with the reported literature values.

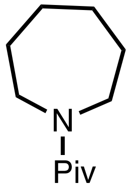

1n

1-(azepan-1-yl)-2,2-dimethylpropan-1-one (**1n**) was prepared according to a published procedure.[3] Spectral data were in full agreement with the reported literature values.

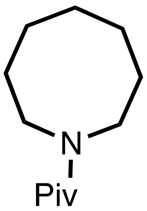

1o

1-(azocan-1-yl)-2,2-dimethylpropan-1-one (**1o**) was prepared according to a published procedure.[3] Spectral data were in full agreement with the reported literature values.

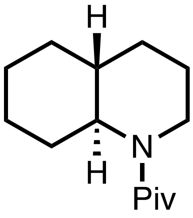

1p

2,2-dimethyl-1-((4a*R*,8a*S*)-octahydroquinolin-1(2*H*)-yl)propan-1-one (**1p**) was prepared according to a published procedure.[3] Spectral data were in full agreement with the reported literature values.

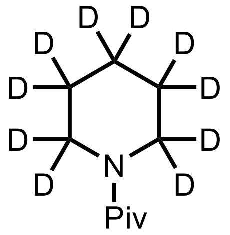

1a-*d*$_{10}$

2,2-dimethyl-1-(piperidin-1-yl-*d*$_{10}$)propan-1-one (**1a-*d*$_{10}$**): The title compound was prepared according to the general procedure using piperidine-*d*$_{11}$ to give **1a-*d*$_{10}$** (881 mg, 95%) as a colorless liquid.
^{1}H NMR (500 MHz, $CDCl_3$) δ 1.24 (s, 9H);
^{13}C NMR (126 MHz, $CDCl_3$) δ 176.2, 45.8 – 45.0 (m), 38.7, 28.5, 25.7 – 24.7 (m), 23.8 – 23.2 (m).
HRMS (ESI): Calc'd for $C_{16}H_{24}NO_2$ $[M+H]^+$: 180.2168, found: 180.2167.

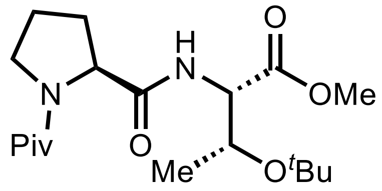

5a

Methyl O-(tert-butyl)-N-(pivaloyl-L-prolyl)-L-threoninate (**5a**) was prepared according to a published procedure.[3] Spectral data were in full agreement with the reported literature values.

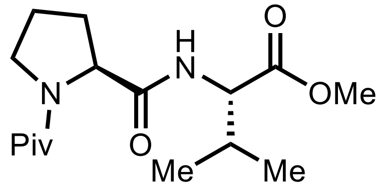

5b

Methyl pivaloyl-L-prolyl-L-valinate (**5b**) was prepared according to a published procedure.[3] Spectral data were in full agreement with the reported literature values.

2.2 Preparation of Photocatalyst 2a (Riboflavin Tetraacetate, RFTA)

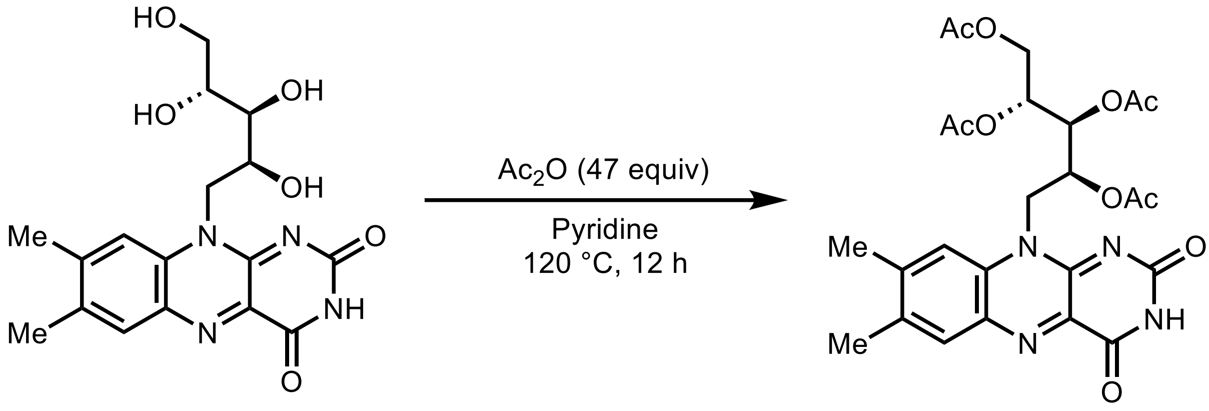

S1 **2a**

(2*R*,3*S*,4*S*)-5-(7,8-dimethyl-2,4-dioxo-3,4-dihydrobenzo[*g*]pteridin-10(2*H*)-yl)pentane-1,2,3,4-tetrayl tetraacetate (**2a**): (–)-Riboflavin (**S1** (10.0 g, 26.6 mmol, 1 equiv) was dissolved in pyridine (120 mL) and acetic anhydride (118 mL, 47 equiv) was added. The resulting reaction mixture was stirred at reflux (120 °C) over night (12 h) and then cooled to room temperature, diluted with CH_2Cl_2 (200 mL) and poured into ice cold HCl (aq., 1M, 200 mL). The aqueous and organic layer were separated, the aqueous phase extracted with CH_2Cl_2 (3 x 100 mL) and the combined organic layers washed with HCl (aq., 1 M, 100 mL) and water (100 mL), dried over Na_2SO_4 and concentrated in *vacuo*. Purification of the crude product via column chromatography (10% MeOH/CH_2Cl_2) afforded the title compound (**2a**) as yellow-brown solid (5.30 g, 37%).

Spectral data were in full agreement with the reported literature values.[6]

2.3 Experimental Setup of Photoreactor

Photochemical reactions were performed either in an air-cooled photobox lined with aluminum foil using a blue Kessil brand A160WE Tuna Blue LED 40 W lamp (Figure S1), or the commercially marketed *Penn PhD Photoreactor M2* (450 nm).

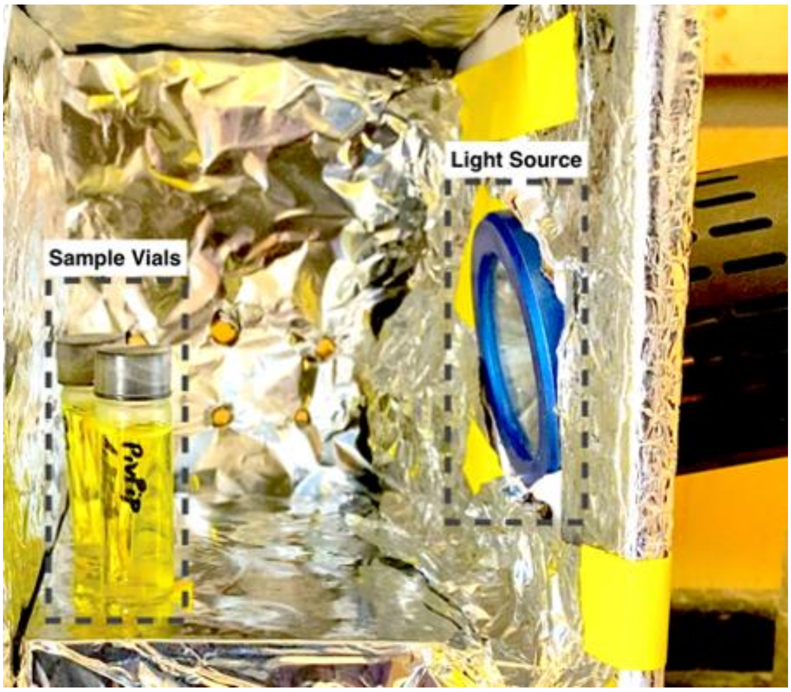

Figure S1. Photoreactor setup using a blue Kessil brand A160WE Tuna Blue LED 40 W lamp.

2.4 Photochemical Method Optimization

Representative Procedure for Photocatalyst Screening

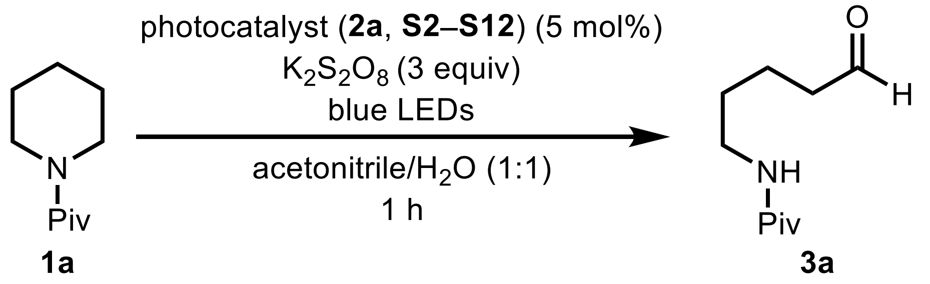

To a 2-dram vial was added sequentially piperidine **1a** (50.7 mg, 0.30 mmol, 1 equiv), potassium persulfate (243 mg, 0.60 mmol, 3 equiv), photocatalyst **(2a**, **S1** – **S11)** (5 mol%) and 15 ml of a 1:1 acetonitrile:H_2O solution. The resulting mixture was sparged with nitrogen for 10 min and subsequently transferred to the photoreactor, exposed to a blue Kessil brand A160WE Tuna Blue LED 40 W lamp and cooled with an airstream. After 1 h, the reaction mixture was partitioned with DCM (5 ml) and the phases were separated. The aqueous phase was extracted with DCM (4 X 5 ml) and the combined organic layers dried over Na_2SO_4 and concentrated under nitrogen. $(C_6H_5)_3CH$ (0.1 mmol, 24.4 mg) was added as NMR standard to the crude reaction product and the yield and conversion determined using ^{1}H-NMR (pg. S69–S70).

Table S1. Results of photocatalyst screening.

entry	catalyst	yield [%][#]	conv. [%][#]
1	**2a**	70 (4)	79
2	**S2**	9 (0)	28
3	**S3**	10 (1)	86
4	**S4**	7 (0)	23
5	**S5**	30 (9)	93
6	**S6**	5 (0)	48
7	**S7**	36 (9)	74
8	**S8**	10 (2)	35
9	**S9**	34 (17)	69
10	**S10**	29 (11)	91
11	**S11**	25 (3)	37
12	**S12**	38 (10)	71

[#] Yield and conversion by ^{1}H integration using Ph_3CH as internal standard. Yield of carboxylic acid shown in parentheses.

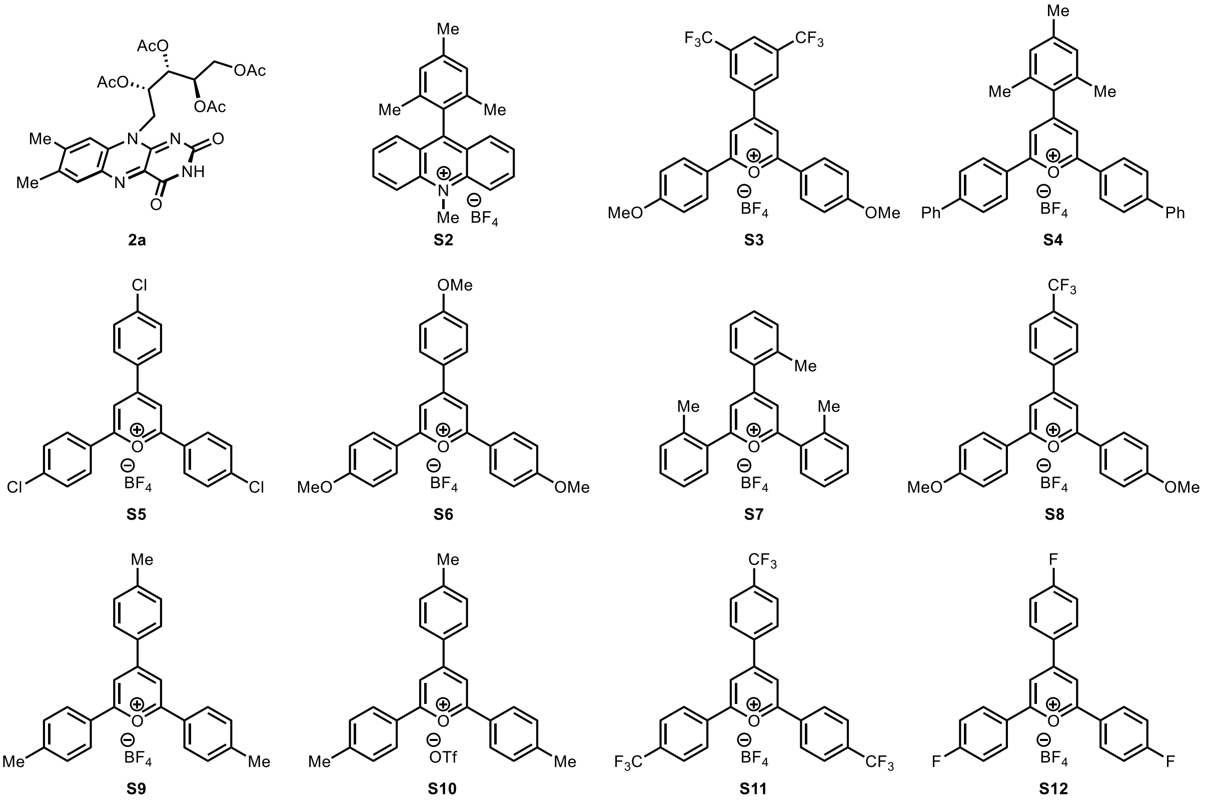

Figure S2. Photocatalysts used for optimization studies.

Photochemical Method – Optimization of Reaction Time

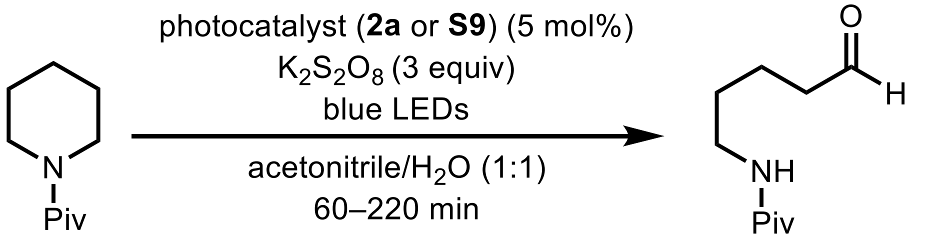

1a **3a**

To a 2-dram vial was added, sequentially, piperidine **1a** (50.7 mg, 0.30 mmol, 1 equiv), potassium persulfate (243 mg, 0.60 mmol, 3 equiv), photocatalyst **2a** or **S9** (5 mol%) and 15 ml of a 1:1 acetonitrile/H_2O solution. The resulting mixture was sparged with nitrogen for 10 min, placed in a photoreactor, and irradiated with a blue Kessil brand A160WE Tuna Blue LED 40 W lamp cooled with an airstream. After the set duration (see Table S2 below), the reaction mixture was partitioned with DCM (5 ml) and the phases were separated. The aqueous phase was extracted with DCM (4 X 5 ml) and the combined organic layers dried over Na_2SO_4 and concentrated under nitrogen. $(C_6H_5)_3CH$ (0.1 mmol, 24.4 mg) was added as NMR standard to the crude reaction product and the yield and conversion determined via ^{1}H-NMR (pg. S71).

Table S2. Optimization of reaction time for photochemical method.

entry	catalyst	time [min]	yield [%]#	conv. [%]#
1	**S9**	60	34 (17)	69
2	**S9**	120	11 (51)	>99
3	**2a**	60	70 (4)	79

4	**2a**	120	83 (13)	99
5	**2a**	220	59 (33)	>99

Yield and conversion by ^{1}H integration using Ph_3CH as internal standard. Yield of carboxylic acid shown in parentheses.

Photochemical Method – Optimization of Catalyst Loading

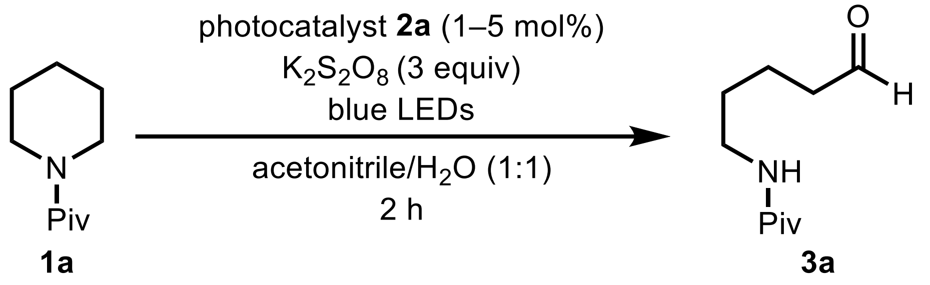

To a 2-dram vial was added, sequentially, piperidine **1a** (50.7 mg, 0.30 mmol, 1 equiv), potassium persulfate (243 mg, 0.60 mmol, 3 equiv), photocatalyst **2a** (1, 2.5, or 5 mol%) and 15 ml of a 1:1 acetonitrile:H_2O solution. The resulting mixture was sparged with nitrogen for 10 min and placed in a photoreactor, irradiated with a blue Kessil brand A160WE Tuna Blue LED 40 W lamp and cooled with an airstream. After 2 h, the reaction mixture was partitioned with DCM (5 ml) and the phases were separated. The aqueous phase was extracted with DCM (4 X 5 ml) and the combined organic layers dried over Na_2SO_4 and concentrated under nitrogen. $(C_6H_5)_3CH$ (0.1 mmol, 24.4 mg) was added as NMR standard to the crude reaction product and the yield as well as conversion determined by ^{1}H-NMR (pg. S72).

Table S3. Optimization of catalyst loading for RTA (**A**).

entry	cat. loading [mol%]	yield [%]#	conv. [%]#
1	1	37 (9)	97
2	2.5	63 (24)	96
3	5	83 (13)	99

Yield and conversion by ^{1}H integration using Ph_3CH as internal standard. Yield of carboxylic acid shown in parentheses.

Photochemical Method – Optimization of Oxidant and Solvent

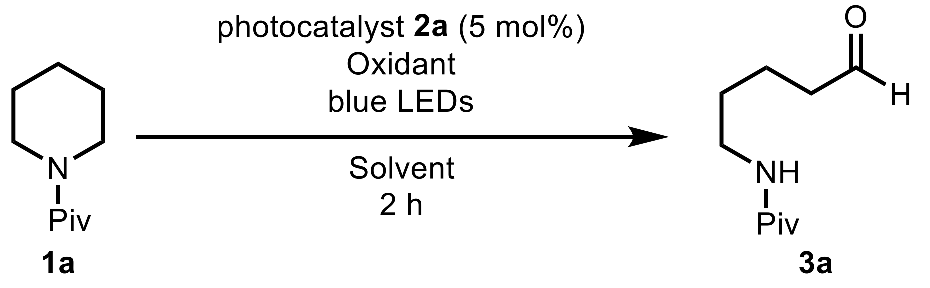

To a 2-dram vial was added, sequentially, piperidine **1a** (50.7 mg, 0.30 mmol, 1 equiv), oxidant (0.60 mmol, 3 equiv; or 1.20 mmol, 6 equiv), photocatalyst **2a** (5 mol%) and 15 ml of a 1:1 solvent solution. The resulting mixture was sparged with nitrogen for 10 min and placed in a photoreactor, exposed to a blue Kessil brand A160WE Tuna Blue LED 40 W lamp and cooled with an airstream. After 2 h, the reaction mixture was partitioned with DCM (5 ml) and the phases were separated. The aqueous phase was extracted with DCM (4 X 5 ml) and the combined organic layers dried over Na_2SO_4 and concentrated under nitrogen. $(C_6H_5)_3CH$ (0.1 mmol, 24.4 mg) was added as NMR standard to the crude reaction product and the yield and conversion determined using ^{1}H-NMR (pg. S72–S73).

Table S4. Optimization of oxidant for photochemical method.

entry	oxidant	equiv.	yield [%][#]	conv. [%][#]
1	$K_2S_2O_8$	3	83 (13)	99
2	$(NH_4)_2S_2O_8$	3	18 (1)	98
3	$Na_2S_2O_8$	3	70 (24)	>99
4	Oxone	3	47 (31)	87
5	H_2O_2 (30%)	3	0	35
6	TBHP	3	0	20

[#] Yield and conversion by ^{1}H integration using Ph_3CH as internal standard. Yield of carboxylic acid shown in parentheses.

Table S5. Optimization of solvent for photochemical method.

entry	solvent	M [mol/L]	yield [%][#]	conv. [%][#]
1	MeCN/H_2O (1:1)	0.02	83 (13)	99
2	Acetone/H_2O (1:1)	0.02	67 (18)	96
3	DCE/H_2O (1:1)	0.02	3 (0)	44

[#] Yield and conversion by ^{1}H integration using Ph_3CH as internal standard. Yield of carboxylic acid shown in parentheses.

Photochemical Method – Variation of Reaction Parameters

Any variation to the reaction conditions is listed as a change to the standard reaction parameters listed below.

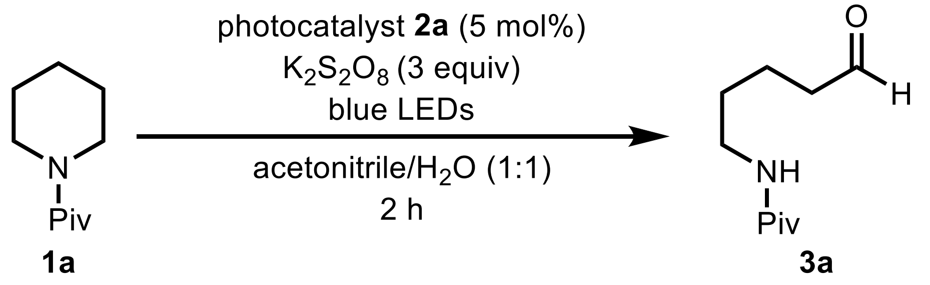

To a 2-dram vial was added, sequentially, piperidine **1a** (50.7 mg, 0.30 mmol, 1 equiv), potassium persulfate (243 mg, 0.60 mmol, 3 equiv), photocatalyst **2a** (8.1 mg, 5 mol%) and 15

ml of a 1:1 acetonitrile:H_2O solution. The resulting mixture was sparged with nitrogen for 10 min and placed in a photoreactor, exposed to a blue Kessil brand A160WE Tuna Blue LED 40 W lamp and cooled with an an airstream. After 2 h, the reaction mixture was partitioned with DCM (5 ml) and the phases were separated. The aqueous phase was extracted with DCM (4 X 5 ml) and the combined organic layers dried over Na_2SO_4 and concentrated under nitrogen. $(C_6H_5)_3CH$ (0.1 mmol, 24.4 mg) was added as an NMR standard to the crude reaction product and the yield and conversion determined using ^1H NMR (pg. S74).

Table S6. Variation of the reaction parameters with respect to the standard conditions.

entry	variation from the standard conditions	yield [%]
1	no sparging, open to air	73 (19)#
2	no riboflavin tetraacetate	29 (12)#
3	riboflavin (**S1**) (without acetate groups)	31 (4)#
4	no oxidant	0#
6	no light source	0#
7	450 nm	45 (34)#

Yield and conversion by ^1H integration using Ph_3CH as internal standard. Yield of carboxylic acid shown in parentheses.

Photochemical Method – N-Protecting Group Screening

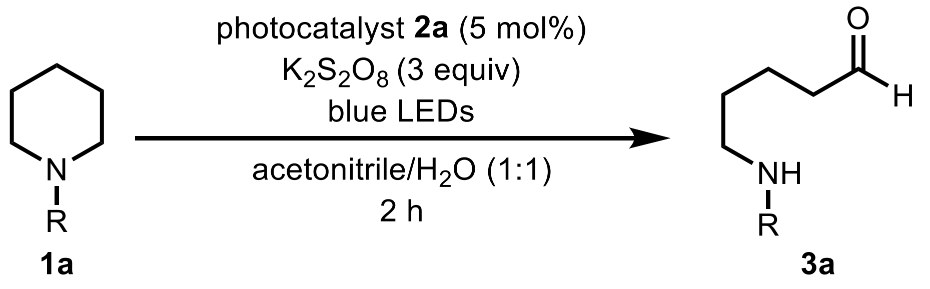

Piperidine derivatives bearing different substituents on nitrogen were subjected to the standard reaction conditions according to the representative procedure. The result obtained for N-Boc piperidine is representative of the observations made under the standard conditions.

For the quantification of the reaction outcome, $(C_6H_5)_3CH$ (0.1 mmol, 24.4 mg) was added as NMR standard to the crude reaction product and the yield and conversion determined using ^1H NMR integration (pg. S75).

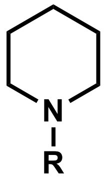

	R	yield#	conv.#
**1a**:	**Piv**	96%	99%
**1a.a**:	**Boc**	18%	86%
**1a.b**:	**Bz**	41%	54%

Yield by ^{1}H integration using Ph_3CH as internal standard.

Figure S3. Results for N-protecting group screening.

2.5 Experiments to Probe Triplet Energy Transfer Mechanism

Some recent reports[7] of photochemical transformations have implicated excited-state triplet energy transfer from the photocatalyst to the substrate or oxidant as a key process in the reaction mechanism. In order to determine the viability of such a reaction pathway in our photoinitiated riboflavin tetraacetate oxidation method, photocatalysts of varying triplet energies were explored in place of riboflavin tetraacetate (**2a**), and the conversion of substrate **1a** was assessed for a correlation between the photocatalyst triplet energy and substrate conversion (see Table S7 below). The excited state triplet energy of the photocatalyst does not appear to have an effect on substrate conversion, so it is unlikely that triplet energy transfer is an operable pathway for oxidation.

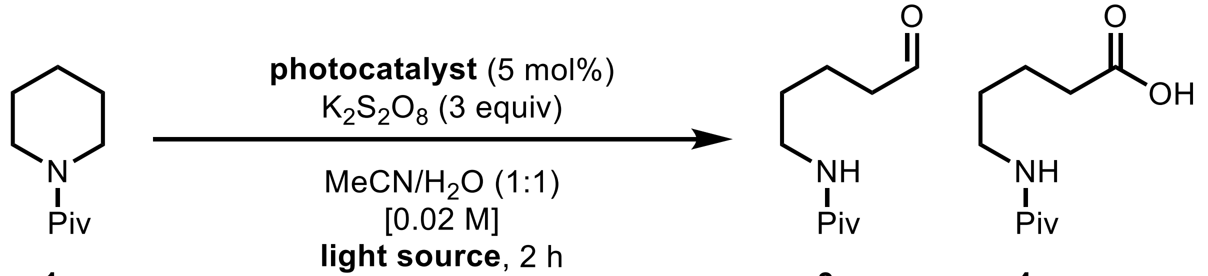

1a **3a** **4a**

Table S7. Results of triplet energy transfer mechanistic experiments.

catalyst	λ [nm]	triplet energy [kcal/mol]	conv. [%]#	3a yield [%]#	4a yield [%]#
(Ir[dF(CF$_3$)ppy]$_2$(dtbpy))PF$_6$	400	61	61	22	0
	450		63	21	0
9-fluorenone	400	53	34	2	0
	450		51	0	0
Ru(bpy)$_3$Cl · H$_2$O	400	47	99	0	46
	450		98	0	54

Yield and conversion by ^{1}H integration using Ph$_3$CH as internal standard.

2.6 Kinetics Experiments

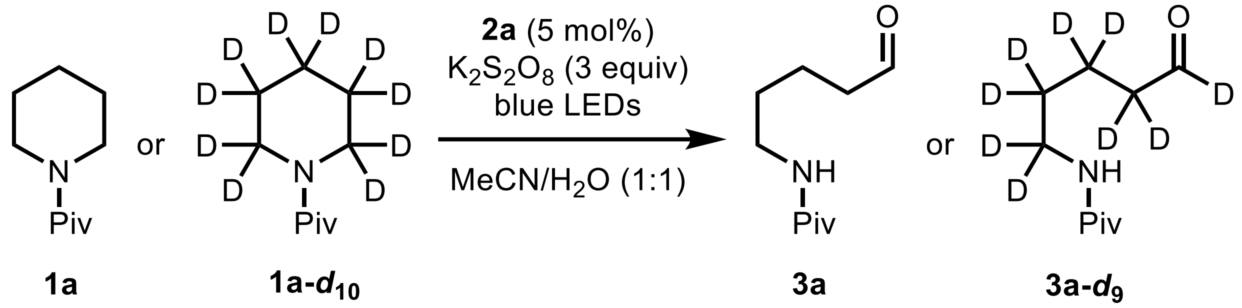

Kinetic Isotope Effect

Absolute rates: **1a** (169 mg, 1.00 mmol, 1 equiv) or **1a-*d*₁₀** (179 mg, 1.00 mmol, 1 equiv), potassium persulfate (811 mg, 3.00 mmol, 3 equiv), and **2a** (27.2 mg, 50.0 µmol, 0.05 equiv) were combined in a 30 mL vial with a magnetic stir bar, dissolved in 20 mL of 1:1 MeCN/H_2O, and the mixture was sparged with N_2. The mixture was allowed to stir under irradiation from a Kessil brand A160WE Tuna Blue LED 40 W lamp. Every 5 minutes, the lamp was quickly turned off, a 0.25 mL aliquot was taken from the mixture, the reaction vessel was placed back into the photobox, and irradiation was resumed. The aliquot was diluted with DI H_2O and extracted with 3 x 1 mL DCM. The combined organic layers were dried over Na_2SO_4, filtered, and concentrated. The resulting crude samples were analyzed by ^{1}H NMR using Ph_3CH as a standard to assess yield of **3a** or **3a-*d*₉**. k_H/k_D = 1.1 (average of three trials).

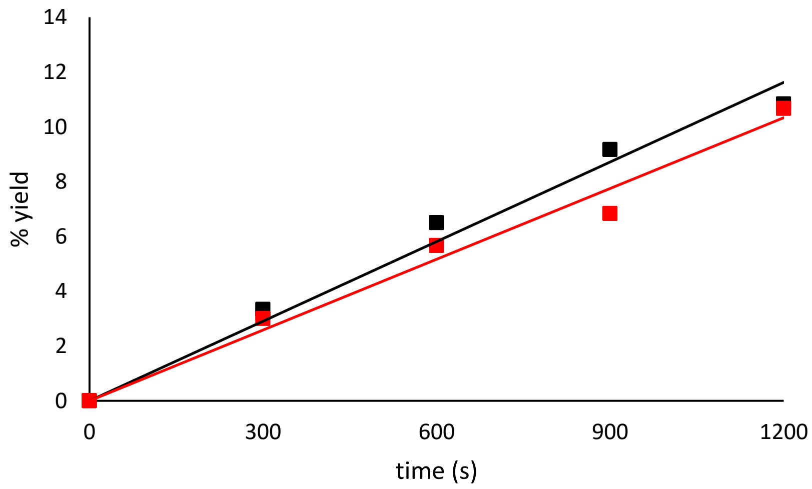

Figure S4. Initial rates for **1a** (black, 9.6 × 10^{-3} M/s) and **1a-*d*₁₀** (red, 8.2 × 10^{-3} M/s), k_H/k_D = 1.1.

Intermolecular competition: **1a** (8.5 mg, 50.0 µmol, 1 equiv) and **1a-*d*₁₀** (8.9 mg, 50.0 µmol, 1 equiv), potassium persulfate (81.1 mg, 0.300 mmol, 6 equiv), and **2a** (2.7 mg, 5.0 µmol, 0.1 equiv) were combined in a 2 dram vial with a magnetic stir bar, dissolved in 5 mL of 1:1 MeCN/H_2O, and the mixture was sparged with N_2. The mixture was allowed to stir under irradiation from a Kessil brand A160WE Tuna Blue LED 40 W lamp for 15 minutes. The mixture was extracted with 3 x 3 mL DCM. The combined organic layers were dried over Na_2SO_4, filtered, and concentrated. The aldehyde products were isolated by preparative thin-layer

chromatography (1:1 hexanes/EtOAc). The resulting mixture of **3a** and **3a-*d*$_9$** was analyzed by ^{1}H NMR using Ph_3CH as a standard to assess yields. $[P_H]/[P_D]$ = 1.0 (average of three trials)

Light On/Off Experiment

1a (169 mg, 1.00 mmol, 1 equiv), potassium persulfate (811 mg, 3.00 mmol, 3 equiv), and **2a** (27.2 mg, 50.0 µmol, 0.05 equiv) were combined in 30 mL vial with a magnetic stir bar, dissolved in 20 mL of 1:1 $MeCN/H_2O$, and the mixture was sparged with N_2. The reaction mixture was irradiated with a Kessil brand A160WE Tuna Blue LED 40 W lamp and kept in the dark for alternating 20 min periods. At the start/end of each period, a 0.25 mL aliquot was taken from the mixture. The aliquot was diluted with DI H_2O and extracted with 3 x 1 mL DCM. The combined organic layers were dried over Na_2SO_4, filtered, and concentrated. The resulting crude samples were analyzed by ^{1}H NMR using Ph_3CH as a standard to assess yield of **3a**.

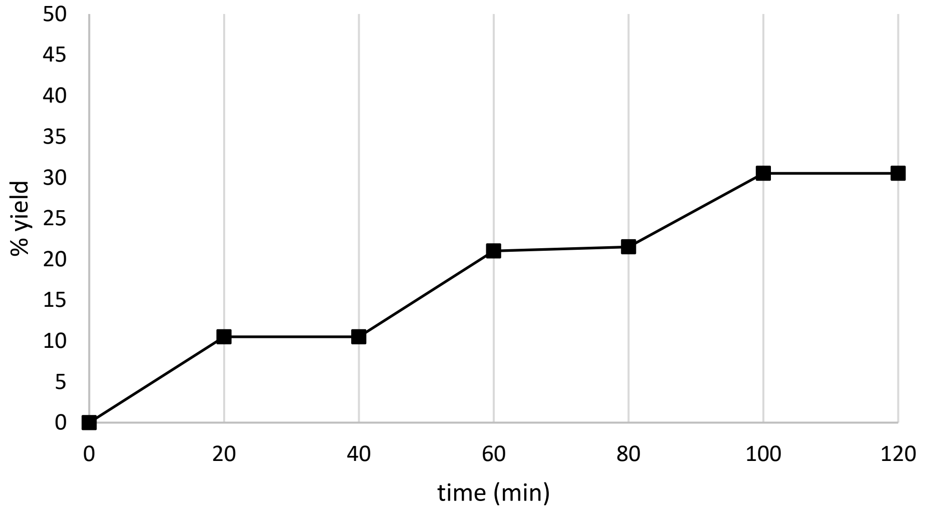

Figure S5. Light on/off experiment.

2.7 Procedure for Riboflavin Tetraacetate-mediated Ring Opening

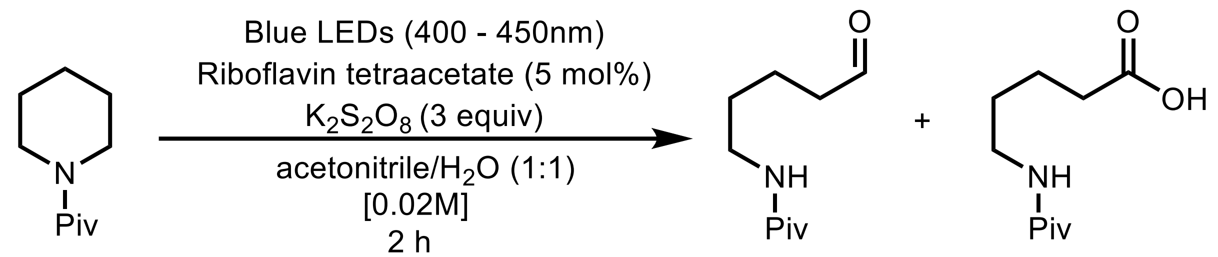

1a **3a** **4a**

To a 2-dram vial was added, sequentially, piperidine **1a** (50.7 mg, 0.30 mmol, 1 equiv), potassium persulfate (243 mg, 0.60 mmol, 3 equiv), photocatalyst riboflavin tetraacetate (8.1 mg, 5 mol%) and 15 ml of a 1:1 acetonitrile:H_2O solution. The resulting mixture was sparged with nitrogen for 10 min and placed in a photoreactor, exposed to a blue Kessil brand A160WE Tuna Blue LED 40 W lamp and cooled with an airstream. After 2 h, the reaction mixture was partitioned with DCM (5 ml) and the phases were separated. The aqueous phase was extracted with DCM (4 X 5 ml) and the combined organic layers dried over Na_2SO_4 and concentrated under nitrogen to afford a mixture of *N*-(5-oxopentyl)pivalamide (**3a**) and 5-pivalamidopentanoic acid (**4a**). The mixture was subjected to an acid-base extraction using sodium bicarbonate (sat. solution) and HCl (5N) to give the major aldehyde product (**3a**) (47.6 mg, 86%) as a dark yellow brownish oil and the minor acid product (**4a**) (1.9 mg, 3%) as a yellowish oil.

Aldehyde (**3a**):

^{1}H NMR (500 MHz, $CDCl_3$): δ 9.73 (t, *J* = 1.5 Hz, 1H), 5.83 (s, 1H), 3.20 (td, *J* = 7.0, 5.8 Hz, 2H), 2.46 (td, *J* = 7.0, 1.4 Hz, 2H), 1.65 – 1.55 (m, 2H), 1.54 – 1.45 (m, 2H), 1.15 (s, 9H);

^{13}C NMR (126 MHz, $CDCl_3$): δ 202.4, 178.6, 43.4, 39.0, 38.7, 29.1, 27.7, 19.1;

HRMS (ESI): Calc'd for $C_{10}H_{20}NO_2$ $[M+H]^+$: 186.1489, found: 186.1488.

Acid (**4a**):

^{1}H NMR (500 MHz, $CDCl_3$): δ 5.76 (s, 1H), 3.26 (qd, *J* = 7.1, 5.8 Hz, 2H), 2.40 (t, *J* = 7.1 Hz, 2H), 1.70 – 1.49 (m, 4H), 1.20 (d, *J* = 1.8 Hz, 9H);

^{13}C NMR (126 MHz, $CDCl_3$): δ 178.9, 177.0, 39.0, 38.8, 33.2, 29.1, 27.7, 21.9.

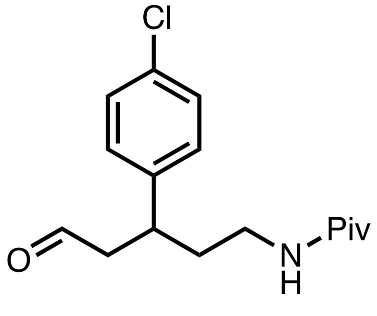

3b

***N*-(3-(4-chlorophenyl)-5-oxopentyl)pivalamide** (**3b**): The title compound was prepared according to the representative procedure using 1-(4-(4-chlorophenyl)piperidin-1-yl)-2,2-dimethylpropan-1-one to give **3b** (9.1 mg, 62%) as a colorless oil.

^{1}H NMR (500 MHz, CDCl$_3$): δ 9.67 (t, *J* = 1.5 Hz, 1H), 7.31 – 7.24 (m, 2H), 7.17 – 7.11 (m, 2H), 5.63 (s, 1H), 3.26 – 3.13 (m, 2H), 3.05 (ddt, *J* = 11.6, 7.5, 5.9 Hz, 1H), 2.84 – 2.69 (m, 2H), 1.92 – 1.73 (m, 2H), 1.14 (s, 9H);
^{13}C NMR (101 MHz, CDCl$_3$): δ 200.9, 178.6, 141.8, 132.8, 129.2, 128.9, 50.6, 38.7, 37.9, 37.1, 36.2, 27.6;
HRMS (ESI): Calc'd for $C_{16}H_{23}NO_2Cl$ $[M+H]^+$: 296.1412, found: 296.1411.

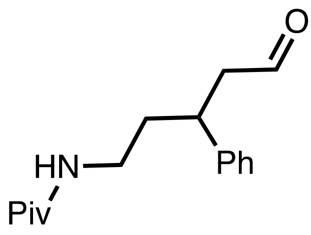

3c

***N*-(5-Oxo-3-phenylpentyl)pivalamide** (**3c**): The title compound was prepared according to the representative procedure using 2,2-dimethyl-1-(4-phenylpiperidin-1-yl)propan-1-one. Purification by preparative thin-layer chromatography (50% EtOAc/hexanes) provided (**3c**) (11.24 mg, 43%, 75% BRSM) as a colorless oil.
^{1}H NMR (600 MHz, CDCl$_3$) δ 9.68 (t, J = 1.7 Hz, 1H), 7.34 – 7.28 (m, 2H), 7.24 – 7.18 (m, 3H), 5.58 (s, 1H), 3.28 – 3.19 (m, 2H), 3.11 – 3.01 (m, 1H), 1.99 – 1.86 (m, 2H), 1.86 – 1.74 (m, 2H), 1.11 (s, 9H);
^{13}C NMR (151 MHz, CDCl$_3$) δ 201.4, 178.6, 143.3, 129.1, 127.5, 50.7, 38.7, 38.0, 36.2, 28.5, 27.6;
HRMS (ESI): Calc'd for $C_{16}H_{24}NO_2$ $[M+H]^+$: 262.1802, found: 262.1802.

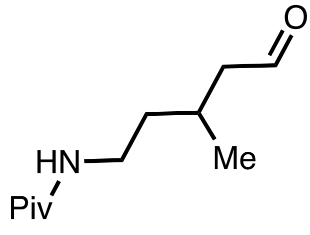

3d

***N*-(3-Methyl-5-oxopentyl)pivalamide** (**3e**): The title compound was prepared according to the representative procedure using 2,2-dimethyl-1-(4-methylpiperidin-1-yl)propan-1-one. Purification by preparative thin-layer chromatography (50% EtOAc/hexanes) provided (**3d**) (15.1 mg, 76%) as a colorless oil.

¹H NMR (400 MHz, $CDCl_3$): δ 9.75 (t, *J* = 1.7 Hz, 1H), 5.83 (s, 1H), 3.34 – 3.13 (m, 2H), 2.56 – 2.24 (m, 2H), 2.10 (dq, *J* = 13.5, 6.7 Hz, 1H), 1.55 – 1.38 (m, 2H), 1.19 (s, 9H), 0.99 (d, *J* = 6.8 Hz, 3H);
¹³C NMR (101 MHz, $CDCl_3$): δ 202.6, 178.7, 51.1, 38.8, 37.5, 36.6, 27.7, 25.5, 20.2;
HRMS (ESI): Calc'd for $C_{11}H_{21}NO_2Na$ $[M+Na]^+$: 222.1465, found: 222.1466.

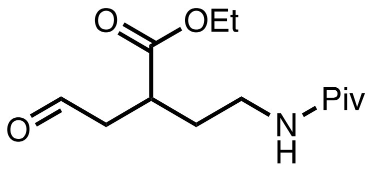

3e

Ethyl 4-oxo-2-(2-pivalamidoethyl)butanoate (**3e**): The title compound was prepared according to the representative procedure using ethyl 1-pivaloylpiperidine-4-carboxylate. Purification by preparative thin-layer chromatography (50% EtOAc/hexanes) provided (**3e**) (34.7 mg, 45%) as a colorless oil.
¹H NMR (500 MHz, $CDCl_3$): δ 9.74 (d, *J* = 1.0 Hz, 1H), 6.03 – 5.97 (m, 1H), 4.14 (q, *J* = 7.1 Hz, 2H), 3.37 – 3.15 (m, 2H), 2.96 – 2.84 (m, 2H), 2.70 – 2.60 (m, 1H), 1.90 – 1.66 (m, 2H), 1.24 (t, *J* = 7.1 Hz, 3H), 1.17 (s, 9H);
¹³C NMR (126 MHz, $CDCl_3$): δ 200.0, 178.8, 174.5, 61.2, 45.4, 38.8, 37.3, 36.7, 31.4, 31.3, 27.6, 14.3;
HRMS (ESI): Calc'd for $C_{13}H_{24}NO_4$ $[M+H]^+$: 258.1700, found: 257.1699.

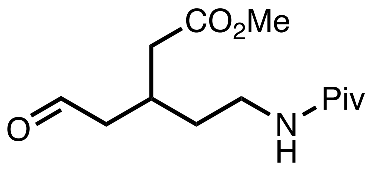

3f

Methyl 5-oxo-3-(2-pivalamidoethyl)pentanoate (**3f**): The title compound was prepared according to the representative procedure using methyl 2-(1-pivaloylpiperidin-4-yl)acetate. However, the catalyst loading was increased to 10 mol%. Purification by preparative thin-layer chromatography (70% EtOAc/hexanes) provided (**3f**) (9.62 mg, 75%) as a colorless oil.
¹H NMR (500 MHz, $CDCl_3$): δ 9.76 (d, *J* = 1.3 Hz, 1H), 6.04 (s, 1H), 3.67 (s, 3H), 3.25 (tdd, *J* = 6.8, 5.5, 1.6 Hz, 2H), 2.57 (dd, *J* = 6.3, 1.3 Hz, 2H), 2.52 – 2.43 (m, 1H), 2.42 – 2.31 (m, 2H), 1.55 (m, 2H), 1.20 (s, 9H);
¹³C NMR (126 MHz, $CDCl_3$): δ 201.4, 178.8, 172.9, 51.7, 48.2, 38.2, 36.9, 34.0, 28.3, 27.6, 26.7;
HRMS (ESI): Calc'd for $C_{13}H_{23}NO_4Na$ $[M+Na]^+$: 280.1519, found: 280.1519.

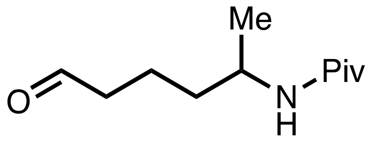

3g

***N*-(6-oxohexan-2-yl)pivalamide** (**3g**): The title compound was prepared according to the representative procedure on a 300 µmol scale using 2,2-dimethyl-1-(2-methylpiperidin-1-yl)propan-1-one. Purification by preparative thin-layer chromatography (50% EtOAc/hexanes) provided (**3g**) (43.3 mg, 72%) as a colorless oil.
^{1}H NMR (500 MHz, $CDCl_3$): δ 9.76 (t, *J* = 1.4 Hz, 1H), 5.41 (s, 1H), 4.04 – 3.92 (m, 1H), 2.56 – 2.40 (m, 2H), 1.62 – 1.38 (m, 4H), 1.19 (d, *J* = 3.6 Hz, 9H), 1.12 (d, *J* = 6.6 Hz, 3H);
^{13}C NMR (126 MHz, $CDCl_3$): δ 202.42, 178.1, 44.7, 43.6, 38.8, 36.4, 27.7, 21.1, 18.5;
HRMS (ESI): Calc'd for $C_{11}H_{22}NO_2$ $[M+H]^+$: 200.1645, found: 200.1646.

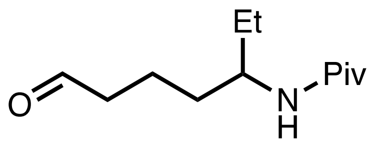

3h

***N*-(7-oxoheptan-3-yl)pivalamide** (**3h**): The title compound was prepared according to the representative procedure on a 300 µmol scale using 1-(2-ethylpiperidin-1-yl)-2,2-dimethylpropan-1-one. Purification by preparative thin-layer chromatography (50% EtOAc/hexanes) provided (**3h**) (33.4 mg, 52%) as a colorless oil.
^{1}H NMR (500 MHz, $CDCl_3$) δ 9.72 (t, *J* = 1.5 Hz, 1H), 5.35 (d, *J* = 9.0 Hz, 1H), 3.82 (dtd, *J* = 12.3, 8.7, 4.5 Hz, 1H), 2.54 – 2.34 (m, 2H), 1.68 – 1.41 (m, 4H), 1.34 (m, 2H), 1.17 (s, 9H), 0.85 (t, *J* = 7.5 Hz, 3H);
^{13}C NMR (126 MHz, $CDCl_3$) δ 202.4, 178.3, 49.9, 43.6, 34.3, 28.2, 27.8, 18.4, 10.3;
HRMS (ESI): Calc'd for $C_{12}H_{24}NO_2$ $[M+H]^+$: 214.1802, found: 214.1800.

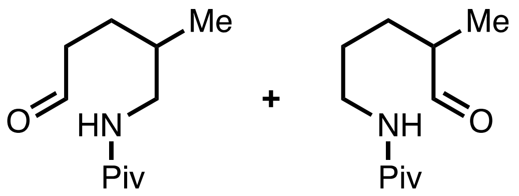

3i.a **3i.b**

***N*-(2-methyl-5-oxopentyl)pivalamide** (**3i.a**) and ***N*-(4-methyl-5-oxopentyl)pivalamide** (**3i.b**): The title compound was prepared according to the representative procedure using 2,2-dimethyl-1-(3-methylpiperidin-1-yl)propan-1-one. Purification by preparative thin-layer chromatography (50% EtOAc/hexanes) provided a mixture of **3i.a** and **3i.b** (14.4 mg, 72%; ratio **3i.a**:**3i.b** 1:0.78) as a colorless oil.
^{1}H NMR (400 MHz, $CDCl_3$): δ 9.76 (t, *J* = 1.3 Hz, 1H), 9.59 (d, *J* = 1.7 Hz, 1H), 5.89 (s, 1H), 5.78 (s, 1H), 3.29 – 3.00 (m, 4H), 2.60 – 2.42 (m, 2H), 2.36 (hd, *J* = 7.0, 1.7 Hz, 1H), 1.77 – 1.28 (m, 6H), 1.18 (d, *J* = 6.0 Hz, 18H), 1.09 (d, *J* = 7.1 Hz, 2H), 0.88 (d, *J* = 6.5 Hz, 3H);
^{13}C NMR (126 MHz, $CDCl_3$): δ 204.9, 202.5, 178.7, 178.6, 46.0, 44.7, 41.5, 39.4, 38.9, 38.8, 33.1, 27.7, 27.7, 27.5, 27.2, 25.9, 17.6, 13.5;
HRMS (ESI): Calc'd for $C_{11}H_{22}NO_2$ $[M+H]^+$: 200.1645, found: 200.1644.

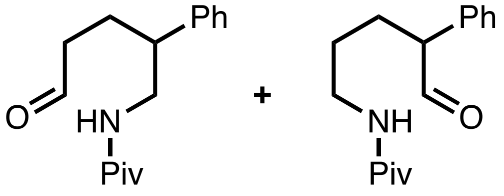

3j.a **3j.b**

***N*-(5-Oxo-2-phenylpentyl)pivalamide** (**3j.a**) and ***N*-(5-Oxo-4-phenylpentyl)pivalamide** (**3j.b**): The title compounds were prepared according to the representative procedure using 2,2-dimethyl-1-(3-phenylpiperidin-1-yl)propan-1-one. Purification by preparative thin-layer chromatography (50% EtOAc/hexanes) provided a mixture of **3j.a** and **3j.b** (52.40 mg, 67%; ratio **3j.a**:**3j.b** 1:0.5) as colorless oils.

^{1}H NMR (500 MHz, $CDCl_3$): δ 9.67 (t, *J* = 1.3 Hz, 1H), 9.66 (d, *J* = 1.6 Hz, 1H), 7.39 – 7.22 (m, 7H), 7.18 – 7.11 (m, 4H), 5.73 (s, 1H), 5.54 (s, 1H), 3.73 – 3.60 (m, 2H), 3.54 (ddd, *J* = 8.1, 6.4, 1.6 Hz, 1H), 3.28 – 3.16 (m, 3H), 2.86 – 2.76 (m, 2H), 2.44 – 2.27 (m, 3H), 2.14 – 1.98 (m, 3H), 1.91 – 1.79 (m, 2H), 1.76 – 1.66 (m, 1H), 1.53 – 1.39 (m, 1H), 1.17 (s, 6H), 1.06 (s, 14H);

^{13}C NMR (126 MHz, $CDCl_3$): δ 201.9, 200.6, 178.6, 178.5, 141.6, 136.1, 129.3, 129.0, 128.9, 127.9, 127.8, 127.3, 58.7, 45.1, 44.9, 41.8, 39.1, 38.7, 38.7, 27.7, 27.5, 27.3, 26.8, 25.5;

HRMS (ESI): Calc'd for $C_{16}H_{24}NO_2$ $[M+H]^+$: 262.1802, found: 262.1801.

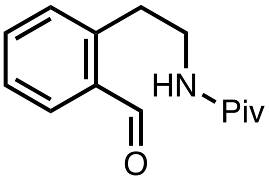

3k

***N*-(2-Formylphenethyl)pivalamide** (**3k**):The title compound was prepared according to the representative procedure using 1-(3,4-dihydroisoquinolin-2(1*H*)-yl)-2,2-dimethylpropan-1-one. Purification by preparative thin-layer chromatography (50% EtOAc/hexanes) provided the title compound (**3k**) (12.13 mg, 52%) as a yellow-brownish oil.

^{1}H NMR (500 MHz, $CDCl_3$) δ 10.17 (s, 1H), 7.80 (dd, *J* = 7.6, 1.6 Hz, 1H), 7.53 (td, *J* = 7.5, 1.5 Hz, 1H), 7.43 (td, *J* = 7.5, 1.2 Hz, 1H), 7.31 (dd, *J* = 7.7, 1.2 Hz, 1H), 5.99 (s, 1H), 3.51 (td, *J* = 6.9, 5.6 Hz, 2H), 3.25 (t, *J* = 6.9 Hz, 2H), 1.12 (s, 9H).

^{13}C NMR (126 MHz, $CDCl_3$) δ 193.8, 178.8, 141.7, 134.4, 134.1, 134.0, 132.1, 127.3, 41.2, 38.8, 32.4, 27.7;

HRMS (ESI): Calc'd for $C_{14}H_{20}NO_2$ $[M+H]^+$: 234.1489, found: 234.1489.

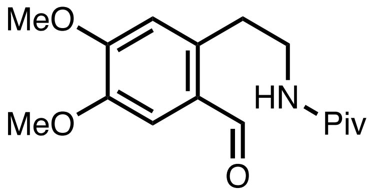

3l

***N*-(2-Formyl-4,5-dimethoxyphenethyl)pivalamide** (**3l**): The title compound was prepared according to the representative procedure using 1-(6,7-dimethoxy-3,4-dihydroisoquinolin-2(1*H*)-yl)-2,2-dimethylpropan-1-one. Purification by preparative thin-layer chromatography (50% EtOAc/hexanes) provided the title compound (**3l**) (27.9 mg, 95%) as a yellow-brownish oil.

^{1}H NMR (500 MHz, $CDCl_3$) δ 10.08 (s, 1H), 7.30 (s, 1H), 6.75 (s, 1H), 5.98 (t, J = 5.9 Hz, 1H), 3.93 (d, J = 9.4 Hz, 6H), 3.49 (td, J = 7.0, 5.8 Hz, 2H), 3.19 (t, J = 7.0 Hz, 2H), 1.13 (s, 9H).

^{13}C NMR (126 MHz, $CDCl_3$) δ 191.0, 178.9, 153.9, 148.0, 137.0, 127.3, 113.8, 113.6, 56.3, 56.2, 41.7, 38.8, 31.7, 27.7;

HRMS (ESI): Calc'd for $C_{16}H_{24}NO_4$ $[M+H]^+$: 294.1700, found: 294.1700.

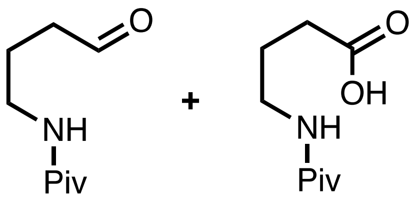

3m **4m**

***N*-(4-oxobutyl)pivalamide** (**3m**) and **4-pivalamidobutanoic acid** (**4m**): The title compounds were prepared according to the representative procedure using *N*-(4-oxobutyl)pivalamide--2,2-dimethyl-1-(pyrrolidin-1-yl)propan-1-one. Evaporation of the organic solvent afforded a mixture of the products (**3m**) and (**4m**). The mixture was subjected to an acid-base extraction using sodium bicarbonate (sat. solution) and HCl (5N) to give the major aldehyde product (**3m**) (30.9 mg, 60%) as a dark yellow brownish oil and the minor acid product (**4m**) (1.4 mg, 2.5%) as a yellowish oil.

Aldehyde (**3m**):

^{1}H NMR (500 MHz, $CDCl_3$): δ 9.77 (t, *J* = 1.2 Hz, 1H), 5.89 (s, 1H), 3.25 (td, *J* = 6.8, 5.6 Hz, 2H), 2.51 (td, *J* = 6.9, 1.2 Hz, 2H), 1.83 (p, *J* = 6.9 Hz, 2H), 1.16 (s, 9H);

^{13}C NMR (126 MHz, $CDCl_3$): δ 202.2, 178.8, 41.7, 39.1, 38.7, 27.6, 22.0;

HRMS (ESI): Calc'd for $C_9H_{18}NO_2$ $[M+H]^+$: 172.1332, found: 172.1334.

Acid (**4m**):

^{1}H NMR (700 MHz, $CDCl_3$) δ 6.05 (s, 1H), 3.35 – 3.29 (m, 2H), 2.39 (t, *J* = 6.9 Hz, 2H), 1.85 (p, *J* = 6.8 Hz, 2H), 1.19 (s, 9H);

^{13}C NMR (176 MHz, $CDCl_3$) δ 179.6, 177.5, 81.5, 39.2, 38.9, 31.7, 27.7, 24.8;

HRMS (ESI): Calc'd for $C_9H_{18}NO_3$ $[M+H]^+$: 188.1281, found: 188.1282.

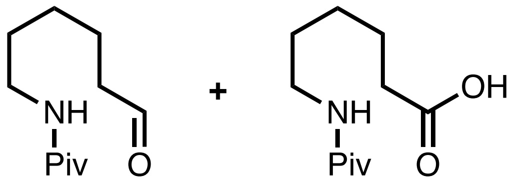

3n **4n**

***N*-(6-oxohexyl)pivalamide** (**3n**) and **6-pivalamidohexanoic acid** (**4n**): The title compounds were prepared according to the representative procedure using 1-(azepan-1-yl)-2,2-dimethylpropan-1-one. Evaporation of the organic solvent afforded a mixture of the products (**3n**) and (**4n**). The mixture was treated with an acid-base extraction using sodium bicarbonate (sat. solution) and HCl (5N) to give the major aldehyde product (**3n**) (16.8 mg, 84%) as a colorless oil and the minor acid product (**4n**) (2.8 mg, 13%) as a yellowish oil.

Aldehyde (**3n**):

^{1}H NMR (500 MHz, $CDCl_3$) δ 9.75 (t, *J* = 1.7 Hz, 1H), 5.68 (s, 1H), 3.23 (td, *J* = 7.2, 5.8 Hz, 2H), 2.44 (td, *J* = 7.2, 1.7 Hz, 2H), 1.64 (p, *J* = 7.3 Hz, 2H), 1.51 (ddd, *J* = 14.8, 7.8, 6.6 Hz, 2H), 1.38 – 1.30 (m, 2H), 1.18 (s, 9H);

^{13}C NMR (101 MHz, $CDCl_3$) δ 202.6, 178.7, 43.8, 39.3, 38.8, 29.5, 27.7, 26.4, 21.7;

HRMS (ESI): Calc'd for $C_{11}H_{22}NO_2$ $[M+H]^+$: 200.1645, found: 200.1646.

Acid (**4n**):

^{1}H NMR (500 MHz, $CDCl_3$) δ 5.67 (s, 1H), 3.25 (td, *J* = 7.2, 5.8 Hz, 2H), 2.36 (t, *J* = 7.4 Hz, 2H), 1.66 (p, *J* = 7.4 Hz, 2H), 1.57 – 1.47 (m, 2H), 1.42 – 1.32 (m, 2H), 1.19 (s, 9H);

^{13}C NMR (126 MHz, $CDCl_3$) δ 178.7, 177.7, 39.4, 38.8, 33.7, 29.4, 27.7, 26.3, 24.4;

HRMS (ESI): Calc'd for $C_{11}H_{22}NO_3$ $[M+H]^+$: 216.1594, found: 216.1595.

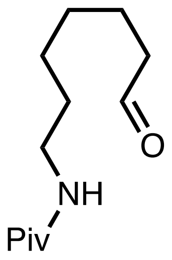

3o

***N*-(7-oxoheptyl)pivalamide** (**3o**): The title compound was prepared according to the representative procedure using 1-(azocan-1-yl)-2,2-dimethylpropan-1-one. Purification by preparative thin-layer chromatography (50% EtOAc/hexanes) provided (**3o**) (19.4 mg, 91%) as a colorless oil.

^{1}H NMR (500 MHz, $CDCl_3$): δ 9.75 (t, *J* = 1.7 Hz, 1H), 5.64 (s, 1H), 3.21 (td, *J* = 7.3, 5.7 Hz, 2H), 2.42 (td, *J* = 7.3, 1.8 Hz, 2H), 1.62 (p, *J* = 7.2 Hz, 2H), 1.49 (p, *J* = 7.2 Hz, 2H), 1.32 (qd, *J* = 6.8, 5.8, 3.6 Hz, 4H), 1.18 (s, 9H);

^{13}C NMR (126 MHz, $CDCl_3$): δ 202.8, 178.5, 43.9, 39.5, 38.8, 29.6, 28.9, 27.7, 26.7, 22.0;

HRMS (ESI): Calc'd for $C_{12}H_{24}NO_2$ $[M+H]^+$: 214.1802, found: 214.1803.

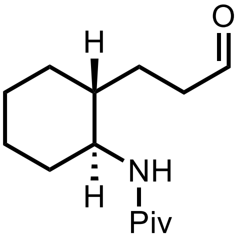

3p

***N*-((1*S*,2*R*)-2-(3-oxopropyl)cyclohexyl)pivalamide** (**3p**): The title compound was prepared according to the representative procedure using 2,2-dimethyl-1-((4a*R*,8a*S*)-octahydroquinolin-1(2*H*)-yl)propan-1-one. Purification by preparative thin-layer chromatography (80% EtOAc/hexanes) provided (**3p**) (15.5 mg, 65%) as a colorless oil.

^{1}H NMR (500 MHz, $CDCl_3$) δ 9.75 (d, *J* = 1.4 Hz, 1H), 5.62 (d, *J* = 8.7 Hz, 1H), 3.56 (tdd, *J* = 11.0, 8.8, 4.1 Hz, 1H), 2.60 – 2.50 (m, 1H), 2.49 – 2.39 (m, 1H), 2.04 – 1.93 (m, 1H), 1.89 – 1.83 (m, 1H), 1.83 – 1.75 (m, 1H), 1.74 – 1.64 (m, 2H), 1.38 – 1.27 (m, 3H), 1.21 (s, 9H), 1.15 – 0.95 (m, 3H);

^{13}C NMR (126 MHz, $CDCl_3$): δ 202.9, 178.1, 51.9, 42.7, 41.0, 38.9, 33.9, 31.1, 27.8, 25.8, 25.3, 24.4;

HRMS (ESI): Calc'd for $C_{14}H_{26}O_2N$ [M+H]$^+$: 240.1958, found: 240.1958;
Calc'd for $C_{14}H_{26}NO_2Na$ [M+Na]$^+$: 262.1778, found: 262.1778.

2.8 Procedure for Copper-Mediated Ring Opening

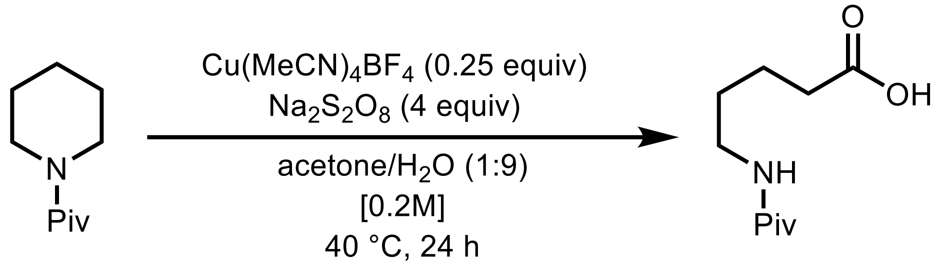

1a **4a**

A 1-dram vial was charged with piperidine **1a** (16.9 mg, 0.10 mmol) and 0.50 mL of a 1:9 acetone:H_2O solution. Sodium persulfate (95 mg, 0.40 mmol) and tetrakis(acetonitrile)copper(I) tetrafluoroborate (7.9 mg, 0.025 mmol) were added to the solution, and the resulting mixture was allowed to stir at 40 °C for 24 h. The reaction mixture was basified with sat. sodium bicarbonate to approx. pH 9, extracted with EtOAc (3 x 3 mL), subsequently acidified to ~pH 4 with 5N HCl and extracted with CH_2Cl_2 (3 x 3 mL). The combined CH_2Cl_2 extracts were dried over Na_2SO_4, filtered, and concentrated under reduced pressure to provide 5-pivalamidopentanoic acid **4a** (10.9 mg, 54%) as a colorless liquid.
^{1}H NMR and ^{13}C NMR spectral data were in full agreement with those for **4a** reported above.
HRMS (ESI): Calc'd for $C_{10}H_{19}NO_3$ $[M+H]^+$: 202.1438, found: 202.1439.

NOTE: Substrates typically show full conversion after a 24 h reaction period. Some over-oxidation can be observed, leading to reduced yields of the desired carboxylic acid.

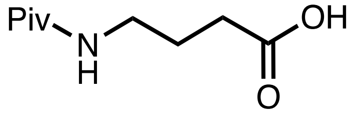

4m

4-Pivalamidobutanoic acid (4m): The title compound was prepared according to the representative procedure on a 200 µmol scale using 2,2-dimethyl-1-(pyrrolidin-1-yl)propan-1-one. Purification by acid-base extraction provided the title compound (**4m**) (22.1 mg, 59%) as a colorless oil.
^{1}H NMR (700 MHz, $CDCl_3$) δ 6.05 (broad s, 1H), 3.32 (td, J = 6.7, 5.8 Hz, 2H), 2.39 (t, J = 6.9 Hz, 2H), 1.85 (apparent p, J = 6.9 Hz, 2H), 1.19 (s, 9H).
^{13}C NMR (176 MHz, $CDCl_3$) δ 179.6, 177.5, 39.2, 38.8, 31.7, 27.6, 24.8.
HRMS (ESI): Calc'd for $C_9H_{18}NO_3$ $[M+H]^+$: 188.1281, found: 188.1282.

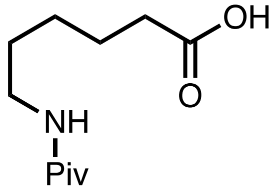

4n

6-Pivalamidohexanoic acid (4n): The title compound was prepared according to the representative procedure on a 200 µmol scale using 1-(azepan-1-yl)-2,2-dimethylpropan-1-one. Purification via acid-base extraction provided the title compound (**4n**) (26.3 mg, 61%) as a colorless oil.
^{1}H NMR (700 MHz, $CDCl_3$) δ 5.74 (broad s, 1H), 3.23 (td, J = 7.2, 5.7 Hz, 2H), 2.34 (t, J = 7.4 Hz, 2H), 1.64 (apparent p, J = 7.4 Hz, 2H), 1.51 (apparent p, J = 7.6 Hz, 2H), 1.44 – 1.33 (m, 2H), 1.18 (s, 9H).
^{13}C NMR (176 MHz, $CDCl_3$) δ 178.9, 178.4, 39.4, 38.8, 33.9, 29.3, 27.7, 26.3, 24.4.
HRMS (ESI): Calc'd for $C_{11}H_{22}NO_3$ $[M+H]^+$: 216.1594, found: 216.1595.

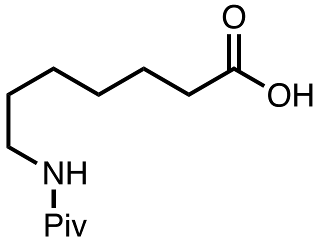

4o

7-Pivalamidoheptanoic acid (4o): The title compound was prepared according to the representative procedure on a 200 µmol scale using 1-(azocan-1-yl)-2,2-dimethylpropan-1-one. Purification using acid-base extraction provided the title compound (**4o**) (24.3 mg, 53%) as a colorless oil.
^{1}H NMR (500 MHz, $CDCl_3$) δ 5.68 (s, 1H), 3.22 (td, J = 7.2, 5.7 Hz, 2H), 2.34 (t, J = 7.4 Hz, 2H), 1.63 (p, J = 7.4 Hz, 2H), 1.50 (p, J = 7.3 Hz, 2H), 1.35 (qtd, J = 16.2, 5.8, 2.8 Hz, 4H), 1.18 (s, 9H);
^{13}C NMR (126 MHz, $CDCl_3$) δ 178.9, 178.8, 39.6, 38.8, 34.0, 29.5, 28.7, 27.7, 26.6, 24.7;
HRMS (ESI): Calc'd for $C_{12}H_{24}NO_3$ $[M+H]^+$: 230.1751, found: 230.1752.

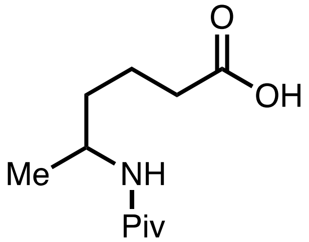

4g

5-Pivalamidohexanoic acid (**4g**): The title compound was prepared according to the representative procedure on a 200 µmol scale using 2,2-dimethyl-1-(2-methylpiperidin-1-yl)propan-1-one. Purification using acid-base extraction provided the title compound (**4g**) (26.7 mg, 62%) as a colorless oil.
^{1}H NMR (500 MHz, $CDCl_3$) δ 5.52 (d, *J* = 8.3 Hz, 1H), 3.97 (ddd, *J* = 14.2, 7.9, 6.1 Hz, 1H), 2.43 – 2.28 (m, 2H), 1.69 – 1.54 (m, 2H), 1.54 – 1.39 (m, 2H), 1.17 (s, 9H), 1.11 (d, 3H);
^{13}C NMR (126 MHz, $CDCl_3$) δ 178.4, 178.3, 44.9, 38.7, 38.6, 36.2, 33.7, 27.7, 21.2, 21.0;
HRMS (ESI): Calc'd for $C_{11}H_{22}NO_3$ $[M+H]^+$: 216.1594, found: 216.1595.

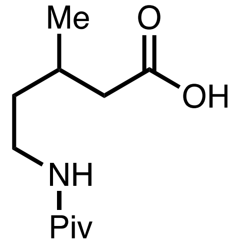

4d

5-Pivalamidoheptanoic acid (4d): The title compound was prepared according to the representative procedure using 1-(2-ethylpiperidin-1-yl)-2,2-dimethylpropan-1-one. Purification using acid-base extraction provided the title compound (**4d**) (25.0 mg, 59%) as a yellow oil.

^{1}H NMR (500 MHz, $CDCl_3$) δ 5.95 (d, J = 5.3 Hz, 1H), 3.27 (td, J = 7.0, 5.4 Hz, 2H), 2.34 (dd, J = 15.6, 7.2 Hz, 1H), 2.23 (dd, J = 15.6, 6.6 Hz, 1H), 1.99 (dq, J = 13.8, 7.0 Hz, 1H), 1.65 – 1.50 (m, 1H), 1.43 (dq, J = 14.2, 7.1 Hz, 1H), 1.17 (s, 9H), 0.99 (d, J = 6.9 Hz, 3H);

^{13}C NMR (126 MHz, $CDCl_3$) δ 179.2, 177.8, 41.3, 38.8, 37.6, 36.2, 27.8, 27.6, 20.1;

HRMS (ESI): Calc'd for $C_{11}H_{22}NO_3$ $[M+H]^+$: 216.1594, found: 216.1595.

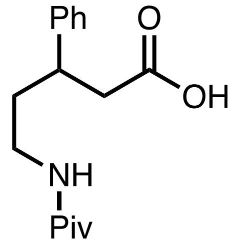

4c

3-phenyl-5-pivalamidopentanoic acid (**4c**): The title compound was prepared according to the representative procedure on a 200 µmol scale using 2,2-dimethyl-1-(4-phenylpiperidin-1-yl)propan-1-one and 1:1 acetone/H_2O solvent. Purification using acid-base extraction provided the title compound (**4c**) (30.2 mg, 54%) as a yellowish oil.

^{1}H NMR (500 MHz, $CDCl_3$) δ 7.33 – 7.27 (m, 2H), 7.24 – 7.16 (m, 3H), 5.64 (t, *J* = 5.6 Hz, 1H), 3.27 – 3.17 (m, 1H), 3.16 – 2.98 (m, 2H), 2.73 – 2.58 (m, 2H), 2.00 – 1.88 (m, 1H), 1.85 - 1.72 (m, 1H), 1.08 (s, 9H);

^{13}C NMR (126 MHz, $CDCl_3$) δ 178.9, 176.7, 143.3, 129.0, 127.5, 127.1, 41.4, 40.1, 38.7, 38.2, 35.7, 27.5;

HRMS (ESI): Calc'd for $C_{11}H_{24}NO_3$ $[M+H]^+$: 278.1751, found: 278.1751.

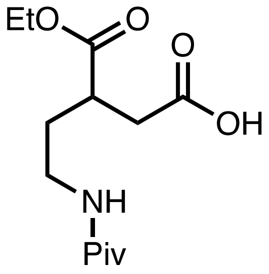

4e

3-(Ethoxycarbonyl)-5-pivalamidopentanoic acid (4e): The title compound was prepared according to the representative procedure on a 200 µmol scale using ethyl 1-pivaloylpiperidine-4-carboxylate. Purification using acid-base extraction provided the title compound (**4e**) (24.9 mg, 46%) as a yellow-orange oil.
^{1}H NMR (500 MHz, $CDCl_3$) δ 6.2 (t, J = 5.8 Hz, 1H), 4.2 (q, J = 7.1 Hz, 2H), 3.5 – 3.1 (m, 2H), 2.9 – 2.8 (m, 2H), 2.5 (dd, J = 16.5, 5.6 Hz, 1H), 1.9 – 1.8 (m, 1H), 1.8 (dtd, J = 14.2, 7.1, 5.3 Hz, 1H), 1.3 (t, J = 7.1 Hz, 3H), 1.2 (s, 9H);
^{13}C NMR (126 MHz, $CDCl_3$) δ 179.4, 175.8, 174.7, 61.2, 39.0, 38.8, 37.6, 35.8, 31.2, 27.6, 14.2;
HRMS (ESI): Calc'd for $C_{13}H_{24}NO_5$ $[M+H]^+$: 274.1649, found: 274.1649.

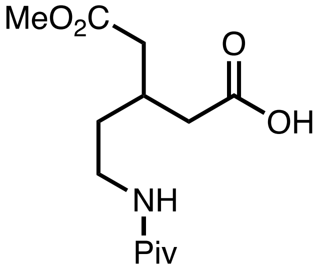

4f

5-methoxy-5-oxo-3-(2-pivalamidoethyl)pentanoic acid (4f): The title compound were prepared according to the representative procedure on a 200 µmol using methyl 2-(1-pivaloylpiperidin-4-yl)acetate. Purification via acid-base extraction provided the title compound (**4f**) (31.2 mg, 57%) as a colorless oil.
^{1}H NMR (500 MHz, $CDCl_3$) δ 6.17 (s, 1H), 3.68 (s, 3H), 3.39 – 3.28 (m, 1H), 3.23 (ddd, J = 14.0, 6.9, 5.5 Hz, 1H), 2.53 – 2.28 (m, 5H), 1.59 (pd, J = 5.8, 2.6 Hz, 2H), 1.20 (s, 9H);
^{13}C NMR (126 MHz, $CDCl_3$) δ 179.6, 176.3, 173.2, 51.9, 38.9, 38.4, 37.2, 33.9, 29.2, 27.63, 27.61;
HRMS (ESI): Calc'd for $C_{13}H_{24}NO_5$ $[M+H]^+$: 274.1649, found: 274.1649.

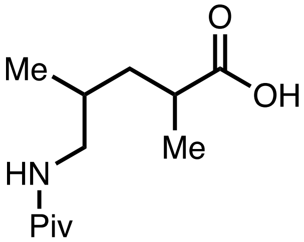

4q

2,4-Dimethyl-5-pivalamidopentanoic acid (4q): The title compound was prepared according to the representative procedure on a 200 µmol scale using 1-(3,5-dimethylpiperidin-1-yl)-2,2-dimethylpropan-1-one. Purification using acid-base extraction provided the title compound (**4q**) (18.8 mg, 41%, mixture of diastereomers 1.0:0.5) as a slight yellowish oil.
^{1}H NMR See ^{1}H NMR spectrum (pg. S101).
^{13}C NMR (126 MHz, $CDCl_3$) δ 182.1, 181.8, 179.3, 179.0, 45.2, 45.1, 38.9 (corresponding peak for minor diastereomer overlapping), 38.3, 38.2, 37.5, 36.9, 32.0, 31.3, 27.70, 27.68, 18.3, 18.0, 17.9, 17.3;

HRMS (ESI): Calc'd for $C_{12}H_{24}NO_3$ $[M+H]^+$: 230.1751, found: 230.1751.

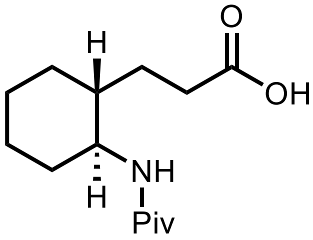

4p

3-(2-Pivalamidocyclohexyl)propanoic acid (4p): The title compound was prepared according to the representative procedure on a 200 µmol scale using 2,2-dimethyl-1-(octahydroquinolin-1(2*H*)-yl)propan-1-one and one equivalent of tetrakis(acetonitrile)copper(I) tetrafluoroborate (63.0 mg, 0.20 mmol). Purification using acid-base extraction provided the title compound (**4p**) (26.5 mg, 52%) as a light orange amorphous solid.
^{1}H NMR (500 MHz, $CDCl_3$) δ 9.74 (br s, 1H), 6.32 (d, J = 7.8 Hz, 1H), 4.59 (td, J = 7.5, 5.0 Hz, 1H), 3.74 (s, 3H), 2.44 – 2.25 (m, 2H), 1.92 – 1.86 (m, 1H), 1.75 – 1.55 (m, 3H), 1.21 (s, 9H);
^{13}C NMR (126 MHz, $CDCl_3$) δ 178.8, 178.6, 51.99, 51.98, 42.6, 38.9, 33.8, 31.0, 27.7, 27.2, 25.7, 25.3;
HRMS (ESI): Calc'd for $C_{14}H_{26}NO_3$ $[M+H]^+$: 256.1907, found: 256.1907.

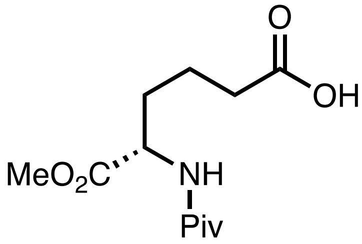

4r

3-(2-Pivalamidocyclohexyl)propanoic acid (4r): The title compound was prepared according to the representative procedure using 2,2-dimethyl-1-(octahydroquinolin-1(2*H*)-yl)propan-1-one. Purification using acid-base extraction provided the title compound (**4r**) (32.0 mg, 62%) as a yellow oil.
^{1}H NMR (500 MHz, $CDCl_3$) δ 9.73 (br s, 2H), 6.32 (d, J = 7.6 Hz, 1H), 4.59 (apparent td, J = 7.5, 5.1 Hz, 1H), 3.74 (s, 3H), 2.70 – 2.23 (m, 2H), 1.91 (apparent ddt, J = 12.3, 9.5, 5.1 Hz, 1H), 1.78 – 1.52 (m, 3H), 1.21 (s, 9H);
^{13}C NMR (126 MHz, $CDCl_3$) δ 178.8, 178.0, 173.2, 52.6, 51.8, 38.9, 33.3, 31.8, 27.5, 20.5;
HRMS (ESI): Calc'd for $C_{12}H_{22}NO_5$ $[M+H]^+$: 260.1492, found: 260.1493.

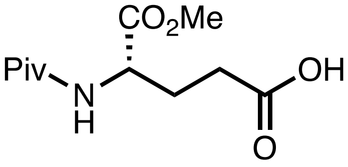

4s

(*S*)-5-Methoxy-5-oxo-4-pivalamidopentanoic acid (4s): The title compound was prepared according to the representative procedure using methyl pivaloyl-*L*-prolinate. Purification using acid-base extraction provided the title compound (**4s**) (29.3 mg, 60%) as a slight yellowish oil.
^{1}H NMR (700 MHz, $CDCl_3$) δ 6.46 (d, J = 7.5 Hz, 1H), 4.62 (td, J = 8.2, 5.1 Hz, 1H), 3.76 (s, 3H), 2.48 – 2.38 (m, 2H), 2.31 – 2.15 (m, 1H), 1.98 (apparent ddt, J = 13.8, 8.5, 6.9 Hz, 1H);
^{13}C NMR (176 MHz, $CDCl_3$) δ 179.2, 176.9, 172.7, 52.8, 51.8, 38.9, 30.3, 27.5, 27.5;
HRMS (ESI): Calc'd for $C_{11}H_{20}NO_5$ $[M+H]^+$: 246.1336, found: 246.1337.

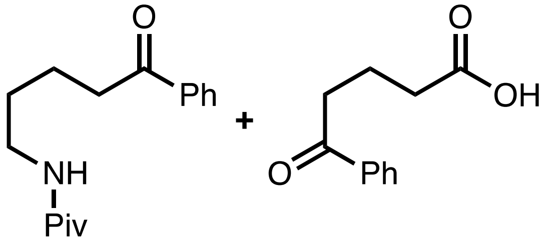

4t **4t.a**

***N*-(5-Oxo-5-phenylpentyl)pivalamide (4t) and 5-oxo-5-phenylpentanoic acid (4t.a):** The title compounds were prepared according to the representative procedure using (*S*)-2,2-dimethyl-1-(2-phenylpiperidin-1-yl)propan-1-one. Purification using acid-base extraction provided the title compounds **4t** (8.9 mg, 17%, 41% brsm) as a colorless oil and **4t.a** (3.5 mg, 9%, 17% brsm) as a white amorphous solid.

4t:
^{1}H NMR (500 MHz, $CDCl_3$) δ 8.14 – 7.86 (m, 2H), 7.64 – 7.51 (m, 1H), 7.46 (dd, J = 8.3, 7.1 Hz, 2H), 5.85 (s, 1H), 3.27 (td, J = 6.9, 5.6 Hz, 2H), 3.02 (t, J = 7.0 Hz, 2H), 1.92 – 1.73 (m, 2H), 1.65 – 1.53 (m, 2H), 1.20 (s, 9H);
^{13}C NMR (126 MHz, $CDCl_3$) δ 200.3, 178.7, 137.0, 133.2, 128.2, 39.2, 38.8, 38.0, 29.2, 27.8;
HRMS (ESI): Calc'd for $C_{16}H_{23}NO_2Na$ $[M+Na]^+$: 284.1621, found: 284.1623.

4t.a:
^{1}H NMR (500 MHz, $CDCl_3$) δ 8.16 – 7.88 (m, 2H), 7.75 – 7.52 (m, 1H), 7.46 (apparent t, J = 7.7 Hz, 2H), 3.09 (t, J = 7.1 Hz, 2H), 2.51 (t, J = 7.1 Hz, 2H), 2.09 (apparent p, J = 7.1 Hz, 2H).
^{13}C NMR (126 MHz, $CDCl_3$) δ 199.5, 178.1, 136.9, 133.3, 128.8, 128.2, 37.4, 33.0, 19.2;
HRMS (ESI): Calc'd for $C_{11}H_{12}O_3Na$ $[M+Na]^+$: 216.0679, found: 216.0677

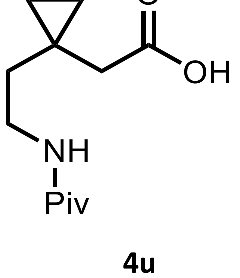

4u

2-(1-(2-pivalamidoethyl)cyclopropyl)acetic acid (4u): The title compound was prepared according to the representative procedure on a 200 µmol scale using 2,2-dimethyl-1-(6-azaspiro[2.5]octan-6-yl)propan-1-one. Purification using acid-base extraction provided the title compound (**4u**) (25.0 mg, 55%) as a yellowish oil.

^{1}H NMR (500 MHz, $CDCl_3$) δ 6.30 (br s, 1H), 3.34 (td, J = 6.8, 5.5 Hz, 2H), 2.31 (s, 2H), 1.57 (t, J = 6.8 Hz, 2H), 1.18 (s, 9H), 0.51 – 0.46 (m, 2H), 0.46 – 0.41 (m, 2H).

^{13}C NMR (126 MHz, $CDCl_3$) δ 179.0, 177.1, 41.0, 38.7, 37.7, 36.3, 27.6, 15.2, 12.3.

HRMS (ESI): Calc'd for $C_{12}H_{22}NO_3$ $[M+H]^+$: 228.1595, found: 228.1595.

2.9 Procedures for Peptide Diversification

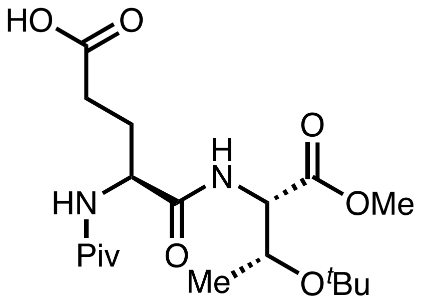

6a

(*S*)-5-(((2*S*,3*R*)-3-(*tert*-Butoxy)-1-methoxy-1-oxobutan-2-yl)amino)-5-oxo-4-pivalamido-pentanoic acid (6a): The title compound was prepared according to the representative procedure using methyl (3*R*)-3-(*tert*-butoxy)-2-((*S*)-1-pivaloylpyrrolidine-2-carboxamido)butanoate (**5a**) with 1:1 acetone/H2O as solvent. Purification using acid-base extraction provided the title compound **6a** (22.5 mg, 56%, mixture of rotamers 1:0.8) as a slightly yellow oil.

^{1}H NMR (500 MHz, $CDCl_3$) δ 7.06 (t, J = 9.3 Hz), 6.92 (d, J = 7.3 Hz), 6.77 (d, J = 7.7 Hz), 4.68 – 4.58 (m), 4.45 (ddd, J = 9.2, 4.4, 1.8 Hz), 4.23 (dtd, J = 12.6, 6.2, 1.8 Hz), 3.70 (d, J = 5.0 Hz), 2.60 – 2.41 (m), 2.26 – 2.14 (m), 2.09 – 1.95 (m), 1.21 (d, J = 9.0 Hz), 1.16 (dd, J = 6.3, 4.9 Hz), 1.10 (d, J = 7.3 Hz);
^{13}C NMR 13C NMR (126 MHz, $CDCl_3$) δ 179.5, 179.3, 176.9, 176.6, 172.24, 172.20, 171.3, 171.1, 74.5, 74.4, 67.4, 67.3, 58.3, 58.1, 52.9, 52.5, 52.3, 39.0, 38.9, 30.5, 30.2, 28.4, 28.2, 27.6, 27.5, 21.09, 21.08;
HRMS (ESI): Calc'd for $C_{18}H_{29}N_9O_2$ $[M+H]^+$: 403.2439, found: 403.2439.

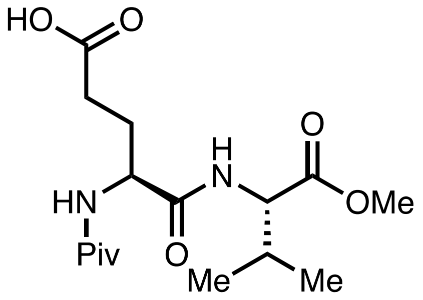

6b

(*S*)-5-(((*S*)-1-Methoxy-3-methyl-1-oxobutan-2-yl)amino)-5-oxo-4-pivalamidopentanoic acid (6b): The title compound was prepared according to the representative procedure using methyl pivaloyl-*L*-prolyl-*L*-valinate (**5b**). Purification using acid-base extraction provided the title compound **6b** (25.5 mg, 74%, mixture of rotamers 1:0.5) as a slightly yellow oil.

^{1}H NMR (500 MHz, $CDCl_3$) δ 7.50 (t, J = 8.2 Hz), 6.84 (d, J = 8.0 Hz), 4.74 – 4.65 (m), 4.45 (dd, J = 8.7, 5.1 Hz), 4.42 (dd, J = 8.3, 5.2 Hz), 3.72 (s), 3.70 (s), 2.48 (qd, J = 5.7, 2.3 Hz), 2.24 – 2.05 (m), 2.01 – 1.88 (m), 1.29 – 1.22 (m), 1.19 (s), 1.18 (s), 0.95 – 0.88 (m);
^{13}C NMR (126 MHz, $CDCl_3$) δ 179.8, 179.7, 176.3, 176.2, 172.2, 172.1, 172.0, 57.9, 57.7, 52.29, 52.28, 52.2, 52.1, 38.94, 38.91, 38.6, 31.0, 30.7, 30.3, 30.1, 27.4, 27.2, 19.2, 19.1, 17.84, 17.83;
HRMS (ESI): Calc'd for $C_{15}H_{23}N_9O$ $[M+H]^+$: 345.2020, found: 345.2019.

Procedures for Peptide Diversification from Dipeptide 6b

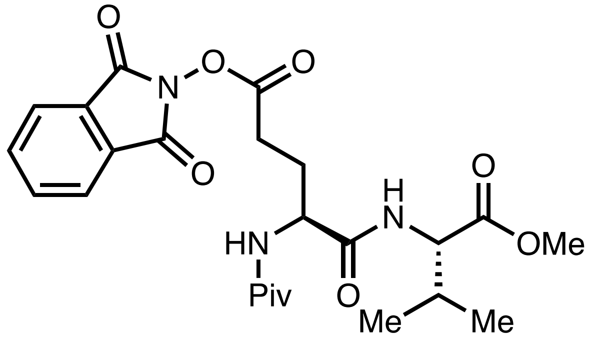

7a

1,3-dioxoisoindolin-2-yl (*S*)-5-(((*S*)-1-methoxy-3-methyl-1-oxobutan-2-yl)amino)-5-oxo-4-pivalamidopentanoate (7a): A 2-dram vial was charged with carboxylic acid **5b** (207 mg, 0.6 mmol), *N*-hydroxyphthalimide (NHPI; 97.9 mg, 0.6 mmol), *N*,*N'*-dicyclohexylcarbodiimide (DCC; 123.8 mg, 0.6 mmol), and dry CH_2Cl_2 (3.0 mL), and the reaction mixture was allowed to stir at room temperature overnight. The mixture was concentrated under reduced pressure, and the resulting crude material was purified by column chromatography (1:2 Hexanes/EtOAc) to provide the title compound **7a** (237 mg, 80%, mixture of rotamers 1:1) as an off-white solid.
^{1}H NMR (500 MHz, $CDCl_3$) δ 7.88 (ddd, J = 5.5, 2.5, 0.9 Hz), 7.79 (ddd, J = 5.5, 3.1, 0.9 Hz), 6.97 (s), 6.44 (d, J = 8.3 Hz), 4.78 – 4.57 (m), 4.47 (ddd, J = 8.5, 4.9, 1.4 Hz), 3.73 (d, J = 0.9 Hz), 3.72 (d, J = 1.1 Hz), 3.04 – 2.83 (m), 2.82 – 2.60 (m), 2.48 – 2.25 (m), 2.18 (dqd, J = 15.0, 6.7, 3.6 Hz), 1.23 (d, J = 0.8 Hz), 1.21 (d, J = 0.7 Hz), 0.94 (dd, J = 10.4, 7.0 Hz), 0.91 (dd, J = 6.9, 1.5 Hz);
^{13}C NMR (126 MHz, $CDCl_3$) δ 179.5, 179.1, 171.9, 171.2, 171.1, 169.69, 169.67, 169.6, 162.0, 161.9, 135.0, 129.0, 124.2, 57.7, 57.6, 52.34, 52.30, 52.2, 52.1, 39.01, 38.95, 31.1, 30.9, 27.8, 27.7, 27.57, 27.55, 27.3, 27.1, 19.2, 19.1, 17.8;
HRMS (ESI): Calc'd for $C_{24}H_{32}NO_8$ $[M+H]^+$: 490.2184, found: 490.2184.

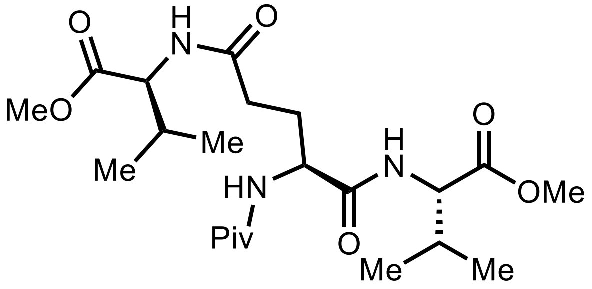

7b

Dimethyl 2,2'-(((S)-2-pivalamidopentanedioyl)bis(azanediyl))(2S,2'S)-bis(3-methylbutanoate) (7b): A 1-dram vial was charged with carboxylic acid **5b** (34.4 mg, 0.1 mmol), *L*-valine methyl ester hydrochloride (18.4 mg, 0.11 mmol), hydroxybenzotriazole (HOBt; 13.5 mg, 0.1 mmol), and dry CH_2Cl_2 (1.5 mL) and cooled to 0 °C in an ice bath. iPr_2Net (23 µL, 0.13 mmol) was added to the mixture dropwise over 5 min, and the resulting mixture was stirred at 0 °C for 10 min. To this mixture was added 1-(3-dimethylaminopropyl)-3-ethylcarbodiimide hydrochloride (EDC; 21.1 mg, 0.11 mmol), and the mixture was allowed to stir and warm to room temperature over 16 h. The reaction mixture was cooled to 0 °C and quenched with 1 M HCl (1 mL). The phases were separated, and the aqueous phase was extracted with CH_2Cl_2 (3 mL × 3). The combined organic layers were washed with brine (1 mL), dried over Na_2SO_4, filtered, and concentrated under reduced pressure. The resulting crude material was purified by column chromatography (1:2 Hexanes/EtOAc) to provide the title compound **7b** (22.9 mg, 50%, mixture of rotamers) as a white amorphous solid.

^{1}H NMR (500 MHz, $CDCl_3$) δ 8.06 (d, J = 8.3 Hz), 7.81 (d, J = 8.8 Hz), 7.30 (d, J = 8.5 Hz), 7.11 (d, J = 9.0 Hz), 6.86 (d, J = 6.8 Hz), 6.46 (d, J = 7.1 Hz), 4.82 (dddd, J = 8.1, 6.9, 2.5, 1.3 Hz), 4.55 (dddd, J = 8.8, 6.4, 5.3, 1.1 Hz), 4.48 – 4.40 (m), 4.28 (ddd, J = 9.3, 7.1, 5.6 Hz), 3.74 (d, J = 0.9 Hz), 3.73 (d, J = 0.8 Hz), 3.73 (d, J = 0.8 Hz), 3.71 (d, J = 0.9 Hz), 2.46 (dd, J = 6.1, 4.3 Hz), 2.43 (dd, J = 6.0, 4.3 Hz), 2.40 – 1.87 (m), 1.18 (d, J = 0.9 Hz), 1.15 (d, J = 0.9 Hz), 1.07 – 0.86 (m);

^{13}C NMR (126 MHz, $CDCl_3$) δ 179.6, 178.3, 174.7, 173.9, 173.8, 173.3, 173.0, 172.4, 172.1, 58.6, 57.7, 57.63, 57.58, 52.7, 52.6, 52.42, 52.41, 52.24, 52.22, 38.8, 38.7, 32.6, 32.4, 30.8, 30.5, 30.4, 29.7, 28.9, 27.53, 27.52, 19.5, 19.3, 19.2, 19.1, 18.1, 18.0, 17.93, 17.90.

HRMS (ESI): Calc'd for $C_{24}H_{32}NO_8$ $[M+H]^+$: 458.2861, found: 458.2864.

2.10 Procedure for Deconstructive Minisci Reaction

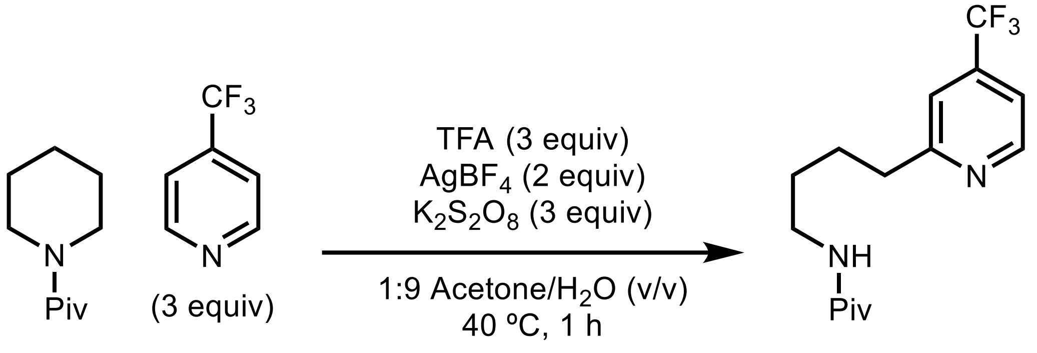

1a **8a** **9a**

A 1-dram vial was charged with **1a** (33.9 mg, 0.20 mmol), **8a** (88.3 mg, 0.60 mmol), and 2.0 mL of a 1:9 acetone:H_2O solution by volume. Trifluoroacetic acid (46 µL, 0.60 mmol) was added to the mixture, and the contents were allowed to stir for 5 min. Potassium persulfate (162 mg, 0.60 mmol) and silver tetrafluoroborate (78 mg, 0.40 mmol) were added and the mixture was allowed to stir at 40 °C. After 1 h, CH_2Cl_2 (2.0 mL) was added and the phases were separated. The aqueous layer was extracted with CH_2Cl_2 (2.0 mL × 3). The combined organic layers were dried over Na_2SO_4, filtered, and concentrated under reduced pressure. The resulting crude mixture was purified by preparative thin-layer chromatography (50% EtOAc/hexanes) to provide *N*-(4-(4-(trifluoromethyl)pyridin-2-yl)butyl)pivalamide (**9a**) (29 mg, 48%) as a yellow oil.

^{1}H NMR (700 MHz, $CDCl_3$) δ 8.71 (d, *J* = 5.2 Hz, 1H), 7.40 (s, 1H), 7.37 (d, *J* = 5.2 Hz, 1H), 5.84 (s, 1H), 3.28 (td, *J* = 7.0, 5.7 Hz, 2H), 2.92 (t, *J* = 7.7 Hz, 2H), 1.84 – 1.75 (m, 2H), 1.57 (p, *J* = 7.2 Hz, 2H), 1.17 (s, 9H).

^{13}C NMR (176 MHz, $CDCl_3$) δ 178.7, 163.4, 149.8, 139.3 (q, *J* = 34.2 Hz), 122.9 (q, *J* = 273.3 Hz), 118.9 (q, *J* = 3.7 Hz), 117.2 (q, *J* = 3.4 Hz), 39.3, 38.8, 37.4, 29.2, 27.7, 26.9.

^{19}F NMR (376 MHz, $CDCl_3$) δ -64.07 (s, 3F).

HRMS (ESI): Calc'd for $C_{15}H_{22}F_3N_2O$ $[M+H]^+$: 303.1679, found: 303.1687.

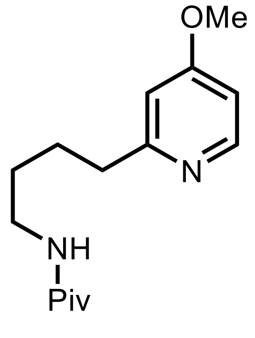

9b

N-(4-(4-Methoxypyridin-2-yl)butyl)pivalamide (**9b**): The title compound was prepared according to the representative procedure using **1a** and **8b**. Purification by preparative thin-layer chromatography (8% MeOH/CH_2Cl_2) provided the title compound (35.7 mg, 67%) as a yellow oil.

^{1}H NMR (700 MHz, $CDCl_3$) δ 8.4 (d, *J* = 5.6 Hz, 1H), 6.7 (dt, *J* = 8.1, 2.4 Hz, 2H), 5.9 (s, 1H), 3.9 (s, 3H), 3.3 (td, *J* = 6.9, 5.5 Hz, 2H), 2.8 (t, *J* = 7.6 Hz, 2H), 1.8 – 1.8 (m, 2H), 1.6 (p, *J* = 7.2 Hz, 2H), 1.2 (s, 9H);
^{13}C NMR (126 MHz, $CDCl_3$) δ 178.6, 166.3, 163.5, 150.3, 108.9, 107.7, 55.2, 39.5, 38.8, 37.8, 29.0, 27.8, 27.1;
HRMS (ESI): Calc'd for $C_{15}H_{25}N_2O_2$ $[M+H]^+$: 265.1911, found: 265.1910.

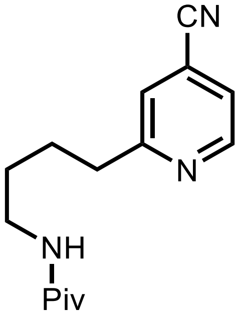

9c

N-(4-(4-cyanopyridin-2-yl)butyl)pivalamide (**9c**): The title compound was prepared according to the representative procedure using **1a** and **8c**. Purification by preparative thin-layer chromatography (5% MeOH/CH_2Cl_2) provided the title compound (26.7 mg, 51%) as a colorless oil.
^{1}H NMR (400 MHz, $CDCl_3$) δ 8.73 (dd, *J* = 5.1, 0.9 Hz, 1H), 7.43 (s, 1H), 7.39 (dd, *J* = 5.0, 1.5 Hz, 1H), 5.88 (s, 1H), 3.30 (td, *J* = 6.9, 5.6 Hz, 2H), 2.91 (t, *J* = 7.6 Hz, 2H), 1.79 (tt, *J* = 8.9, 6.9 Hz, 2H), 1.64 – 1.39 (m, 2H), 1.21 (s, 9H).
^{13}C NMR (101 MHz, $CDCl_3$) δ 178.8, 163.6, 150.1, 124.7, 122.7, 121.0, 116.7, 39.2, 38.7, 37.5, 29.1, 27.7, 26.7.
HRMS (ESI): Calc'd for $C_{15}H_{22}N_3O$ $[M+H]^+$: 260.1758, found: 260.1758.

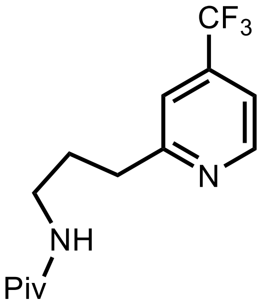

9d

N-(3-(4-(trifluoromethyl)pyridin-2-yl)propyl)pivalamide (**9d**): The title compound was prepared according to the representative procedure using **1m** and **8a**. Purification by preparative thin-layer chromatography (50% EtOAc/hexanes) provided the title compound (35.8 mg, 62%) as a yellow oil.
^{1}H NMR (500 MHz, $CDCl_3$): δ 8.69 (d, *J* = 5.2 Hz, 1H), 7.37 (s, 1H), 7.34 (d, *J* = 5.2 Hz, 1H), 6.11 (s, 1H), 3.31 (q, *J* = 6.6 Hz, 2H), 2.92 (t, *J* = 7.4 Hz, 2H), 1.99 (p, *J* = 7.1 Hz, 2H), 1.17 (s, 9H);
^{13}C NMR (126 MHz, $CDCl_3$) δ 178.7, 163.1, 150.3, 138.9 (q, *J* = 33.9 Hz), 122.9 (q, *J* = 273.1 Hz), 118.7 (q, *J* = 3.6 Hz), 117.0 (q, *J* = 3.5 Hz), 39.2, 38.8, 35.7, 28.9, 27.7.

^{19}F NMR (470 MHz, $CDCl_3$) δ -64.85 (s, 3F);
HRMS (ESI): Calc'd for $C_{14}H_{20}F_3N_2O$ $[M+H]^+$: 289.1522, found: 289.1531.

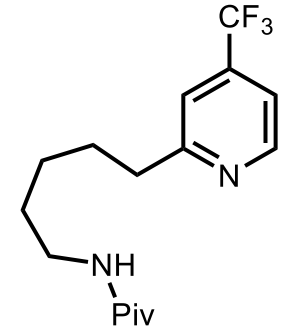

9e

N-(5-(4-(Trifluoromethyl)pyridin-2-yl)pentyl)pivalamide (**9e**): The title compound was prepared according to the representative procedure using **1n** and **8a**. Purification by preparative thin-layer chromatography (50% EtOAc/hexanes) provided the title compound (32.8 mg, 52%) as a yellow oil.
^{1}H NMR (500 MHz, $CDCl_3$) δ 8.68 (d, *J* = 5.1 Hz, 1H), 7.34 (s, 1H), 7.32 (dd, *J* = 5.1, 1.6 Hz, 1H), 5.62 (s, 1H), 3.23 (td, *J* = 7.2, 5.7 Hz, 2H), 2.92 – 2.79 (m, 2H), 1.85 – 1.73 (m, 2H), 1.54 (p, *J* = 7.3 Hz, 2H), 1.43 – 1.33 (m, 2H), 1.16 (s, 9H).
^{13}C NMR (126 MHz, $CDCl_3$) δ 178.5, 163.8, 150.3, 138.7 (q, *J* = 33.7 Hz), 123.0 (q, *J* = 273.1 Hz), 118.4 (q, *J* = 3.6 Hz), 116.8 (q, *J* = 3.6 Hz), 39.4, 38.8, 38.3, 29.6, 29.3, 27.7, 26.6.
^{19}F NMR (470 MHz, $CDCl_3$) δ -64.82 (s, 3F).
HRMS (ESI): Calc'd for $C_{16}H_{24}F_3N_2O$ $[M+H]^+$: 317.1836, found: 317.1835.

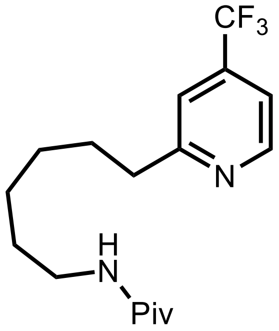

9f

N-(6-(4-(Trifluoromethyl)pyridin-2-yl)hexyl)pivalamide (**9f**): The title compound was prepared according to the representative procedure using **1o** and **8a**. Purification by preparative thin-layer chromatography (50% EtOAc/hexanes) provided the title compound (26.1 mg, 39%) as a yellow oil.
^{1}H NMR (500 MHz, $CDCl_3$) δ 8.71 (d, *J* = 5.2 Hz, 1H), 7.37 (s, 1H), 7.34 (d, *J* = 4.9 Hz, 1H), 5.62 (s, 1H), 3.22 (td, *J* = 7.3, 5.7 Hz, 2H), 2.90 – 2.84 (m, 2H), 1.76 (p, *J* = 7.3 Hz, 2H), 1.51 (p, *J* = 7.3 Hz, 2H), 1.38 (qd, *J* = 9.8, 9.3, 4.1 Hz, 4H), 1.19 (s, 9H).
^{13}C NMR (151 MHz, $CDCl_3$) δ 178.5, 164.0, 150.2, 138.8, 126.2 – 119.8 (m), 118.5 (q, *J* = 4.0, 3.5 Hz), 116.9 (q, *J* = 3.4 Hz), 39.6, 38.8, 38.3, 31.1, 29.6, 29.0, 27.8, 26.7.
^{19}F NMR (376 MHz, $CDCl_3$) δ -64.01 (s, 3F).

HRMS (ESI): Calc'd for $C_{17}H_{26}F_3N_2O$ $[M+H]^+$: 331.1992, found: 331.1991.

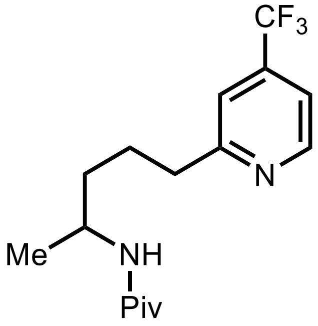

9g

N-(5-(4-(Trifluoromethyl)pyridin-2-yl)pentan-2-yl)pivalamide (**9g**): The title compound was prepared according to the representative procedure using **1g** and **8a**. Purification by preparative thin-layer chromatography (50% EtOAc/hexanes) provided the title compound (31.8 mg, 50%) as a yellow oil.

^{1}H NMR (500 MHz, $CDCl_3$) δ 8.61 (d, *J* = 5.2 Hz, 1H), 7.27 (s, 1H), 7.24 (dd, *J* = 5.2, 1.6 Hz, 1H), 5.42 – 5.32 (m, 1H), 3.95 (dq, *J* = 8.4, 6.6 Hz, 2H), 2.80 (qdd, *J* = 13.9, 8.7, 6.6 Hz, 2H), 1.68 (dddt, *J* = 18.3, 13.9, 8.9, 6.6 Hz, 2H), 1.47 – 1.35 (m, 2H), 1.09 (s, 9H), 1.03 (d, *J* = 6.6 Hz, 3H).

^{13}C NMR (126 MHz, $CDCl_3$) δ 177.9, 163.6, 150.2, 138.8 (q, *J* = 33.6 Hz), 123.0 (q, *J* = 273.1 Hz), 118.6 (q, *J* = 3.5 Hz), 116.9 (q, *J* = 3.5 Hz), 44.8, 38.7, 38.0, 36.6, 27.7, 26.2, 21.1.

^{19}F NMR (470 MHz, $CDCl_3$) δ -64.84 (s, 3F).

HRMS (ESI): Calc'd for $C_{16}H_{24}F_3N_2O$ $[M+H]^+$: 317.1835, found: 317.1836.

2.11 Procedure for Autocyclization Reaction

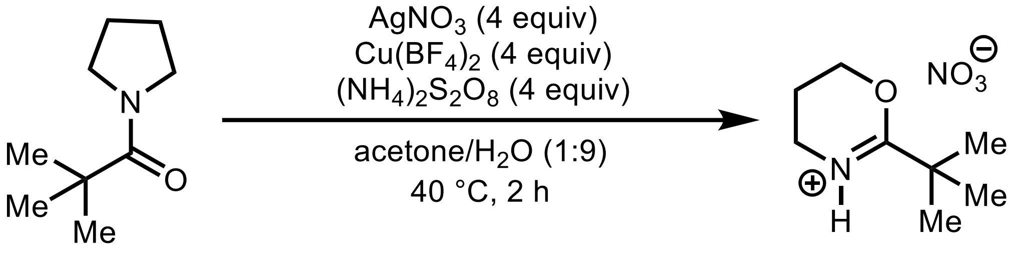

1m **10m**

2-(*tert*-Butyl)-5,6-dihydro-4*H*-1,3-oxazine (10m): To a 1-dram vial was added, sequentially, cyclic amine **1m** (31.1 mg, 0.20 mmol, 1 equiv), silver nitrate (136 mg, 0.80 mmol, 4 equiv), copper(II) tetrafluoroborate (190 mg, 0.80 mmol, 4 equiv), ammonium persulfate (183 mg, 0.80 mmol, 4 equiv) and 1 ml of a 1:1 acetone:H_2O solution. The resulting mixture was then stirred at 40 °C for 2 h. The reaction mixture was diluted with water (0.5 mL) and extracted with 3 x 3 mL of diethylether and the organic layers were discarded. The aqueous layer was then extracted with DCM (3 x 3 mL) and the combined DCM layers were dried over Na_2SO_4, filtered, and concentrated under reduced pressure, affording the title compound as a white solid (26.5 mg, 65%).

^{1}H NMR (500 MHz, $CDCl_3$) δ 10.1 (s, 1H), 4.7 (t, J = 5.5 Hz, 2H), 3.7 (d, J = 5.9 Hz, 2H), 2.2 (p, J = 5.7 Hz, 2H), 1.3 (d, J = 1.3 Hz, 9H).
^{13}C NMR (126 MHz, $CDCl_3$) δ 182.0, 70.0, 39.4, 39.1, 26.3, 18.7.
HRMS (ESI): Calc'd for $C_8H_{16}NO$ $[M+H]^+$: 142.1226, found: 142.1227.

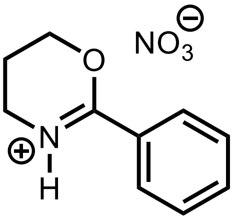

10v

2-phenyl-5,6-dihydro-4*H*-1,3-oxazine (10v): The title compound was prepared according to the representative procedure using phenyl(piperidin-1-yl)methanone. Preparative TLC (1:1 Et_2O/DCM) afforded the title compound **10v** (18.0 mg, 40%) as a white solid.

^{1}H NMR (500 MHz, $CDCl_3$) δ 11.54 (s, 1H), 8.01 – 7.93 (m, 2H), 7.67 (td, J = 7.4, 1.2 Hz, 1H), 7.51 (t, J = 8.0 Hz, 2H), 4.81 (t, J = 5.4 Hz, 2H), 3.83 (t, J = 6.0 Hz, 2H), 2.30 (p, J = 5.8 Hz, 2H).
^{13}C NMR (126 MHz, $CDCl_3$) δ 168.9, 135.7, 129.5, 128.5, 125.4, 70.2, 39.6, 19.1.
HRMS (ESI): Calc'd for $C_{10}H_{12}NO$ $[M+H]^+$: 162.0913, found: 162.0914.

2.12 Counterion Effect for Autocyclization Reaction

The autocyclization reaction was conducted according to the representative procedure above but instead using copper salts with different counter-anions to determine whether other products, such as the expected olefin or ligand-transfer products[8], might be formed. The only materials observed were the open chain aldehyde, carboxylic acid, cyclization product, and unreacted substrate.

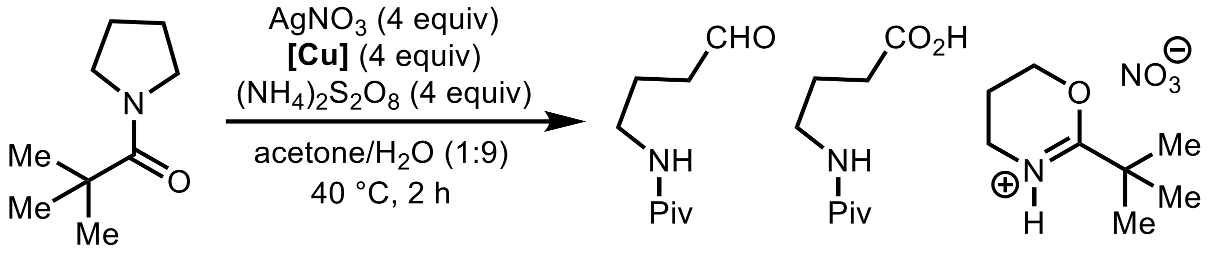

1m 3m 4m 10m

Table S8. Results of autocyclization reaction with various copper salts.

[Cu]	recovered 1m [%]#	3m yield [%]#	4m yield [%]#	10m yield [%]#
$CuCl_2$	33	3	5	—
$CuBr_2$	69	3	3	—
$Cu(OAc)_2 \cdot H_2O$	16	9	15	3
$CuSO_4 \cdot 5H_2O$	—	—	13	12
$Cu(MeCN)_4BF_4$	—	—	46	6

Yield and conversion by ^1H integration using Ph_3CH as internal standard.

3. Computational Details

3.1 General Computational Considerations for Photo- and Copper-Mediated Reactions

Optimization of all reported structures and frequency calculations for the reactions of (a) the riboflavin tetraacetate (**2a**), potassium persulfate, and N-Piv-piperidine (**1a**), as well as the reaction of (b) $CuBF_4$, sodium-persulfate, and N-Piv-piperidine (**1a**) were performed using the Gaussian-16 suite of programs[9] at the B3LYP-D3(BJ)/[6-31G(d,p) + Lanl2dz (Cu)] level of theory with the corresponding Hay-Wadt effective core potential[10–12] for Cu. Therefore, in the calculations described here, we used the B3LYP density functional[13–15] with Grimme's empirical dispersion-correction (D3)[16] and Becke-Johnson (BJ) damping-correction.[17–19] Frequency analyses were used to characterize each minimum and transition state (TS) with zero and one imaginary frequency, respectively. Intrinsic reaction coordinate (IRC) calculations were performed for all TSs to ensure their true nature. Bulk solvent effects were incorporated for all calculations (including geometry optimizations and frequency calculations) using the self-consistent reaction field polarizable continuum model (IEF-PCM).[20,21] Water was chosen as solvent. The reported thermodynamic data were computed at a temperature of 298.15 K and at 1 atm of pressure. Various spin states (including the open-shell singlet states, where that is appropriate) were considered for all key species. All UV-Vis calculations were performed at the TD-DFT [14] level of theory.[22] Previously, we[23,24] and others[25,26] have demonstrated that the B3LYP-D3(BJ) and B3LYP are a reasonable choice for geometry optimization, frequency calculations, as well as for calculation of the relative energies. The role of intersystem crossing (ISC) was examined by locating low energy points on the singlet-triplet crossing surfaces, or seams. These points, which we call minima on the seam of crossing (MSX), were searched using the MECPRO optimize [see: Hamill LA, Snyder JD, Ess DH (**2016**) MECPro Version 1.0.3: Minimum Energy Crossing Program] by starting from each of the triplet and singlet minima along the reaction pathway. We used the xTB's Conformer-Rotamer Ensemble Sampling Tool (CREST) software to identify possible stable conformers of **{(2.A.m)–[$K_2S_2O_8$]}** and **{(2.A.m)–[$K_2S_2O_8$]}–(1a)**. In these calculations, conformers were sampled using the MF-MD-GC workflow. Later, we utilized the outcomes of these conformational analyses in our DFT calculations, and always have validated the related potential conformers. However, in this Article, we discussed only lowest energy structures.

3.2 Critical Interactions in {(2.A.m)–[$K_2S_2O_8$]} and {(2.A.m)–[$K_2S_2O_8$]}–(1a)

We wish to emphasize that our initial conformational analyses of **{(2.A.m) – [$K_2S_2O_8$]}** and **{(2.A.m)–[$K_2S_2O_8$]}–(1a)**, performed using the xTB's Conformer-Rotamer Ensemble Sampling Tool (CREST) software, have enabled us to identify several critically important interactions both between (**2.A.m**) and $K_2S_2O_8$ as well as between the **{(2.A.m)– [$K_2S_2O_8$]}** unit and substrate **1a** (See Figure S6). Over the course of this study, we always have utilized these interactions for design of our DFT calculations. Thus, we always validated the related potential conformers, and we discuss only the lowest energy structures in this Article.

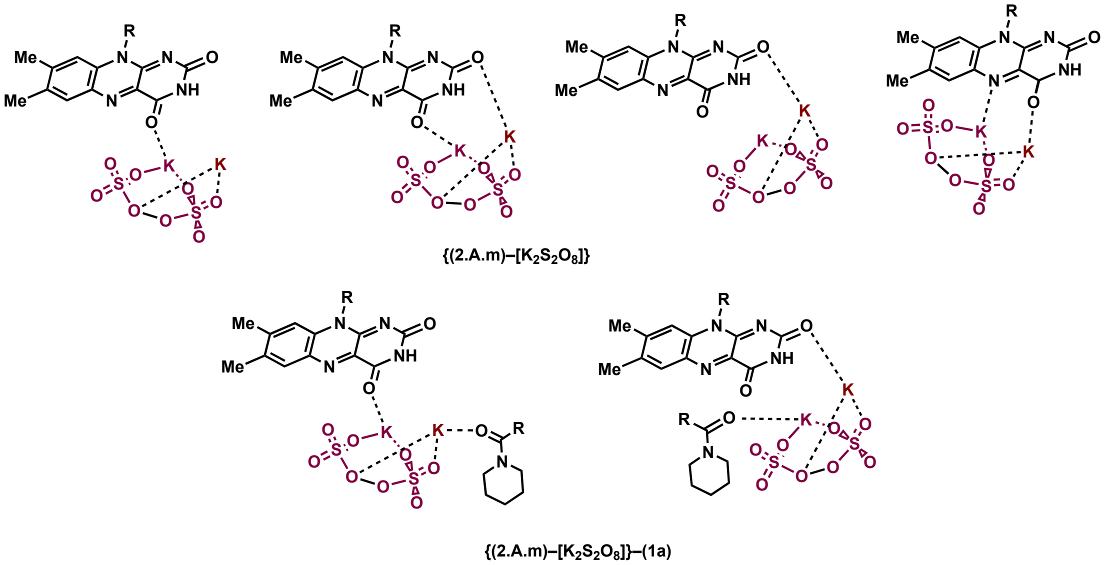

Figure S6. The validated conformers of the **{(2.A.m)–[$K_2S_2O_8$]}** and **{(2.A.m)–[$K_2S_2O_8$]}–(1a)** structures.

3.3 Verification of Photocatalyst Model

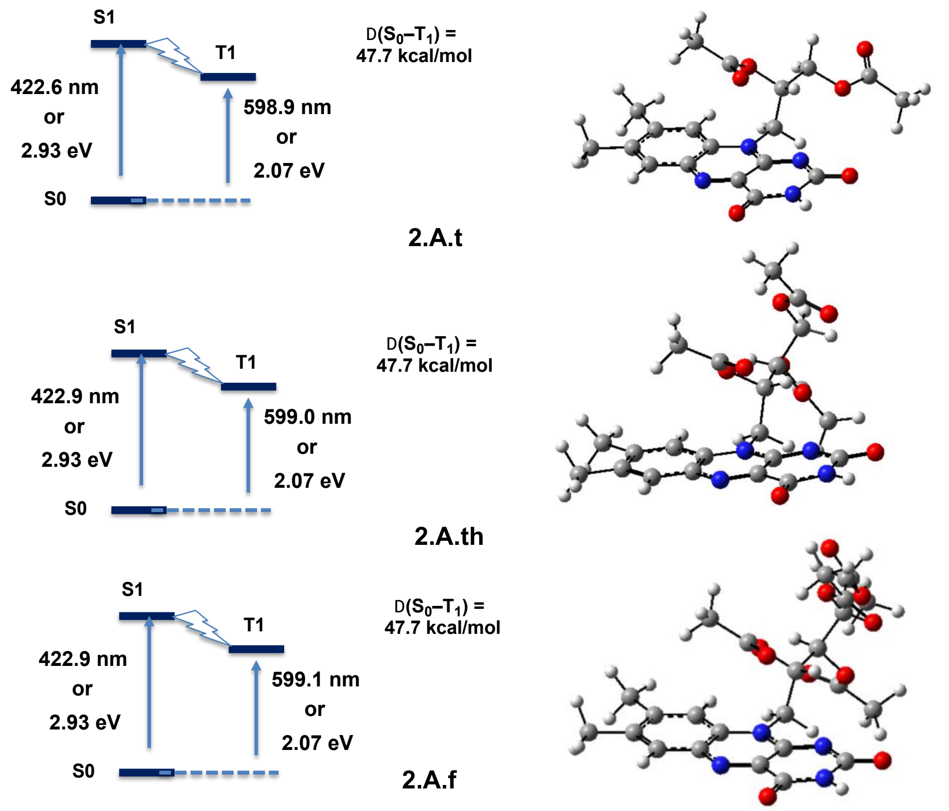

Figure S7. Validation for the use of the isoalloxazine monoacetate as a model for isoalloxazine tetraacetate.

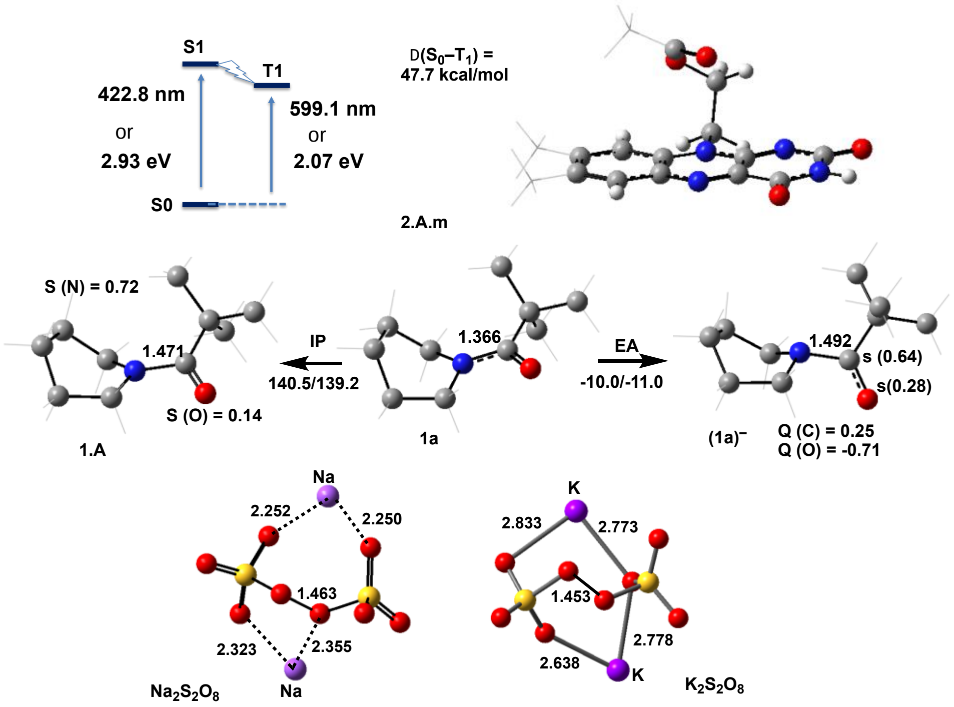

Figure S8. The calculated geometry parameters and relative energies of isoalloxazine monoacetate, (**2.A.m**), substrate **1a**, iminium ion **1.A**, $Na_2S_2O_8$, and $K_2S_2O_8$.

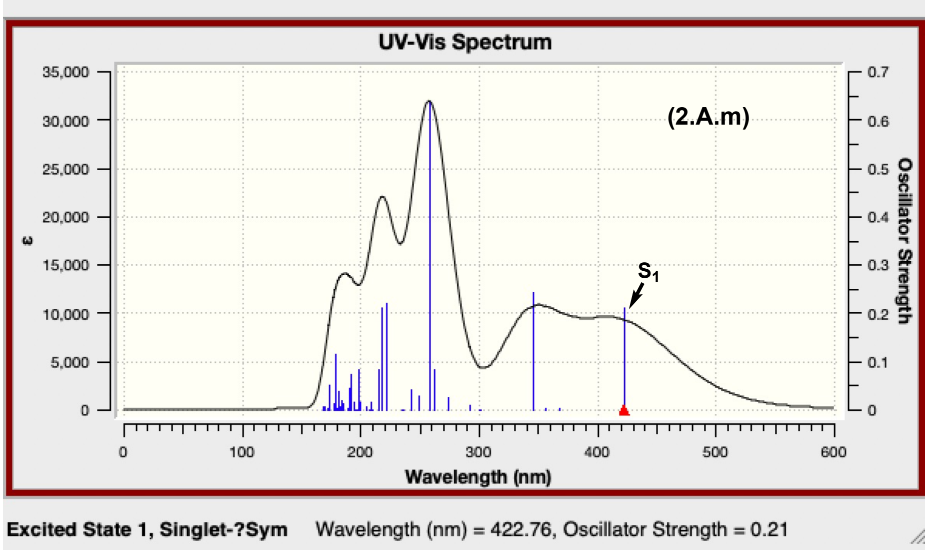

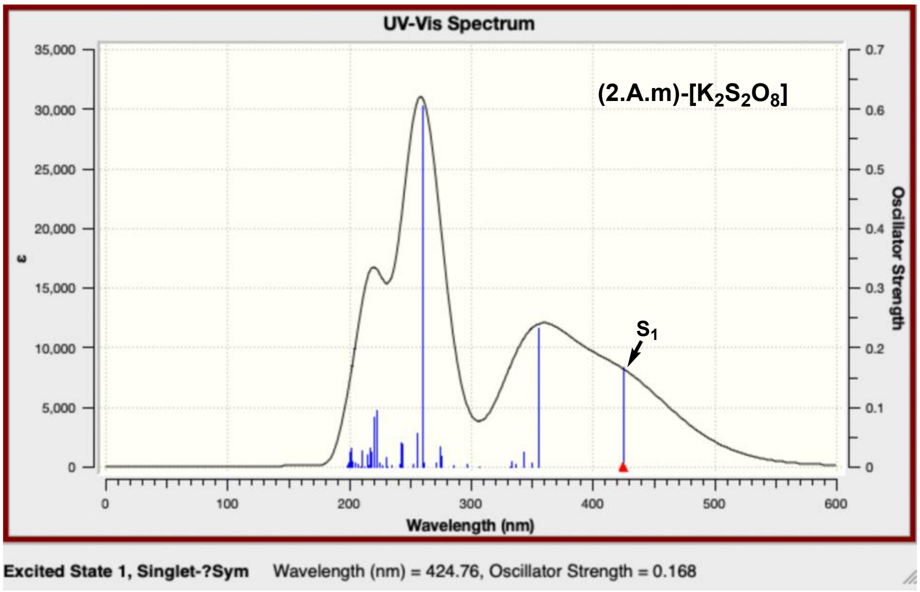

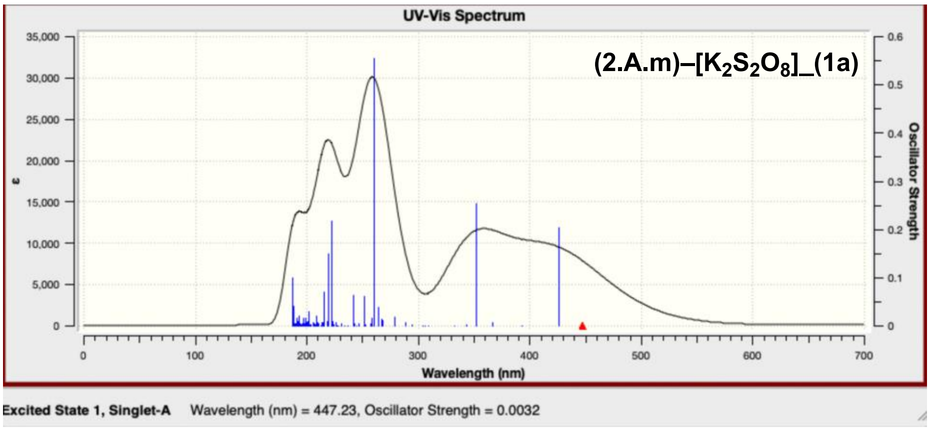

Figure S9. The calculated UV-vis spectra of isoalloxazine monoacetate, (**2.A.m**, isoalloxazine - persulfate **(2.A.m)–[$K_2S_2O_8$]**, and **{(2.A.m)–[$K_2S_2O_8$]–(1a)}** systems.

Table S9. Excitation energies (in eV and nm), oscillator strengths (f) for the isoalloxazine monoacetate-persulfate adduct, **{(2.A.m)–[$K_2S_2O_8$]–(1a)}**, and associated frontier molecular orbitals.

Excited State 1: Singlet-A 2.7723 eV 447.23 nm f=0.0032 (dark)
HOMO -> LUMO 0.70640

Excited State 2: Singlet-A 2.9117 eV 425.81 nm f=0.2049 (bright)
HOMO-3 -> LUMO 0.13122
HOMO-1 -> LUMO 0.68961

Excited State 3: Singlet-A 3.1542 eV 393.08 nm f=0.0002 (dark)
HOMO-2 -> LUMO 0.70468

Excited State 4: Singlet-A 3.3778 eV 367.06 nm f=0.0065 (dark)
HOMO-8 -> LUMO 0.45939
HOMO-6 -> LUMO -0.23666
HOMO-5 -> LUMO 0.45320

Excited State 5: Singlet-A 3.5251 eV 351.72 nm f=0.2539 (bright)
HOMO-3 -> LUMO 0.66806
HOMO-1 -> LUMO -0.11662
HOMO-1 -> LUMO -0.12354

-----------------------------------MOs---------------------------------
LUMO+1 energy = –0.045754 a.u.

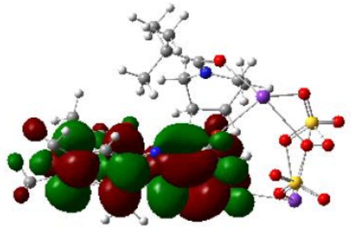

LUMO –0.112529 a.u.

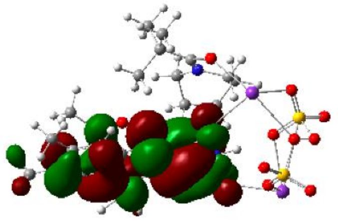

HOMO –0.234451 a.u.

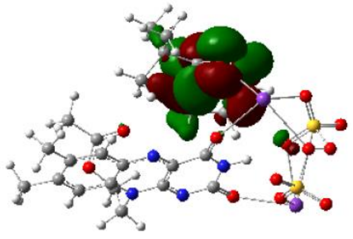

HOMO–1 –0.235775 au.u

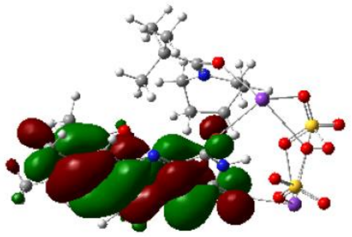

HOMO–2 –0.248124

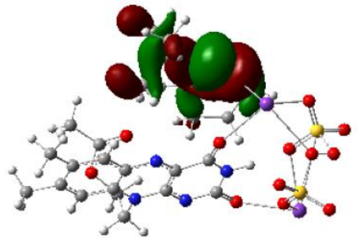

HOMO–3 –0.261656

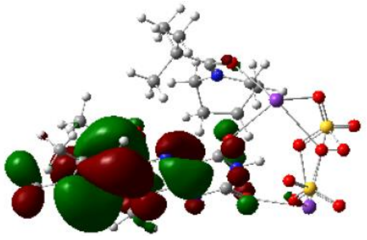

To validate that $K_2S_2O_8$ is not the photocatalyst under blue light irradiation, we have calculated the several lower-lying excited states of $K_2S_2O_8$ at the TD-DFT level. These calculations (see Figure S10) show that the first feasible excited transition of its UV-Vis spectra is the S_0/S_4 transition at 201.1 nm with a transition dipole of f = 0.0102. This is the HOMO–3 $\rightarrow$ LUMO transition, where the LUMO is the antibonding orbital of the peroxy O–O bond. Indeed, at 450 nm irradiation, flavin catalysis should lead to substrate oxidation rather than the persulfate homolysis.

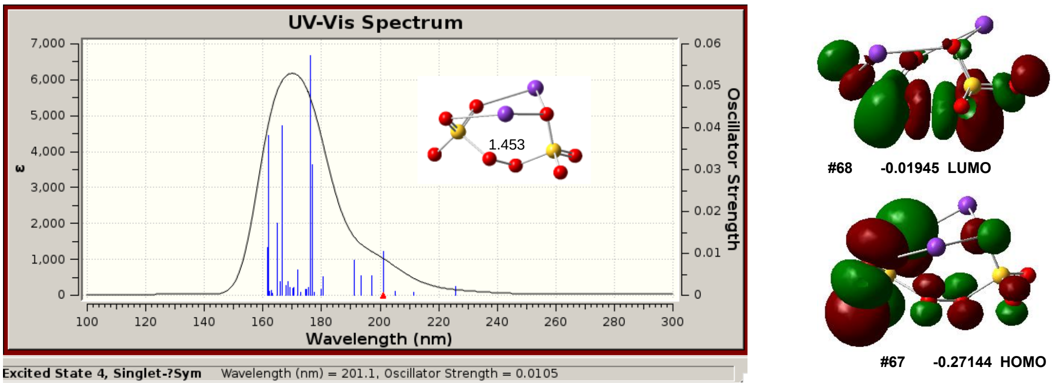


```
Excited State   1:   5.4885 eV  225.90 nm  f=0.0020
    65 -> 68        0.51235
    66 -> 68        0.35060
    67 -> 68       -0.22669

Excited State   2:   5.8605 eV  211.56 nm  f=0.0008
    65 -> 68        0.16933
    66 -> 68        0.16572
    67 -> 68        0.64189

Excited State   3:   6.0389 eV  205.31 nm  f=0.0009
    65 -> 68       -0.39161
    66 -> 68        0.55695

Excited State   4:   6.1654 eV  201.10 nm  f=0.0105
    60 -> 68        0.11249
    64 -> 68        0.65696
```

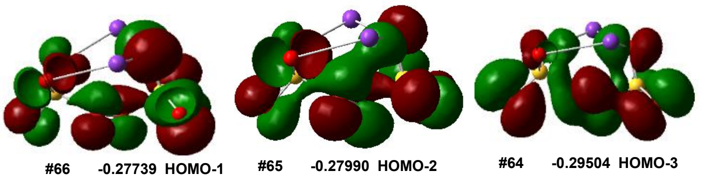

Figure S10. The calculated UV-Vis spectrum of the $K_2S_2O_8$ oxidant and associated frontier molecular orbitals with their energies (in a.u.). Here, we also show the character of the first four transitions as well as their energies and dipole moments (f).

3.4 Computed Reaction Mechanism for Photo-Mediated Reaction

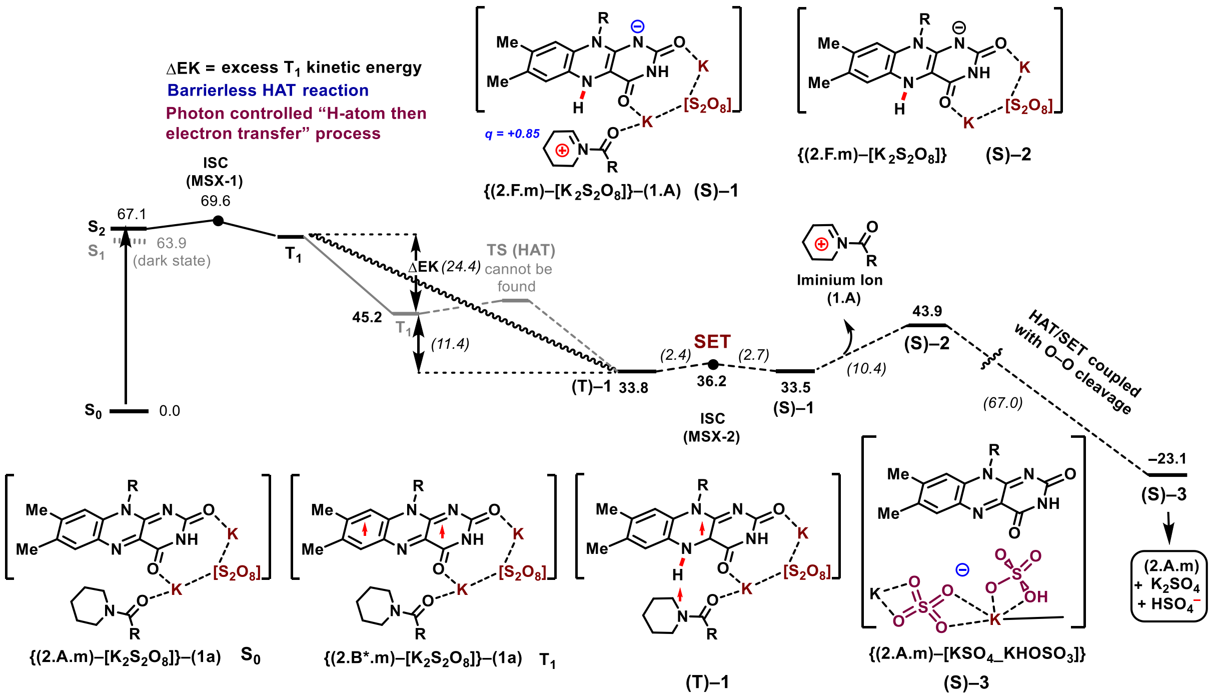

Figure S11. Computed reaction mechanism for the photocatalytic transformation of *N*-Piv-piperidine (**1a**) to iminium ion **1.A** by the reaction of riboflavin monoacetate (**2.A.m**), **1a**, and $K_2S_2O_8$. All energies are listed in kcal/mol, and energy differences are shown in parentheses. Here, the energies given for the reaction $\mathbf{T_1} \rightarrow$ **[(S)-3]** are Gibbs free energies (calculated relative to the $\mathbf{T_1}$ state), except the energy of the **MSX-2** which is an electronic energy (E) calculated relative to that of the **(T)–1** intermediate. The presented energies for the $\mathbf{S_0} \rightarrow \mathbf{T_1}$ are electronic energies, calculated relative to the $\mathbf{S_0}$ state.

The processes leading to the formation of iminium ion and **(S)–2** intermediate were presented in main text. From the byproduct **(S)–2** (i.e.,{(**2.F.m**)–[$K_2S_2O_8$]}**)**, the reaction undergoes *SET, HAT, and O–O homolysis* to form the singlet state complex {(**2.A.m**)–[K_2SO_4]–[$HOSO_3^-$], **(S)-3**. This process may proceed in a stepwise or concerted fashion. The conversion **(S)–2** $\rightarrow$ **(S)–3** is calculated to be highly exergonic (by 77.4 kcal/mol). Because of the multi-component nature of this reaction, we failed to locate the associated transition state(s). However, we have established that the first step of this multi-step process is a SET from the fully reduced isoalloxazine-acetate, (**2.F.m**), to the potassium-persulfate.

3.5 Optimized Structure Energy Components for Photo-Mediated Reaction

Table S10. Calculated total electronic (E_{tot}), E_{tot} with zero-point energy corrections (E_{tot} + ZPEC), enthalpy (H), and Gibbs free energies (in Hartrees; all values are negative) of all reported structures for the reaction of the isoalloxazine-acetate, potassium persulfate, and N-Piv-piperidine (**1a**)

Str	$-E_{tot}$	$-E_{tot}$ + ZPEC	–H	–G
(**2.A.m**)_sing	1139.423323	1139.110825	1139.088549	1139.16196
(**2.A.m**)_trip	1139.351508	1139.042038	1139.019378	1139.094658
(**2.A.m**)_1min	1139.540237	1139.230171	1139.20776	1139.281876
(**2.A.m**)_1plus	1139.200118	1138.88866	1138.866161	1138.940735
$K_2S_2O_8$	2598.037921	2598.003612	2597.988744	2598.048963
K_2SO_4	1899.058273	1899.041357	1899.032089	1899.078054
(**2.A.t**), sing	1406.636299	1406.253586	1406.225075	1406.31546
(**2.A.th**), sing	1673.850366	1673.396492	1673.362321	1673.465634
(**2.A.f**), sing	1941.066384	1940.541517	1940.501644	1940.618886
**1a**, singlet	522.575576	522.29325	522.279314	522.331989
**1a**, triplet	522.4478571	522.169834	522.155278	522.211105
**1a**_1plus	522.3498876	522.069779	522.055457	522.110236
**1a**_1minus	522.5861193	522.309523	522.295276	522.349493
**1.A**	521.7908303	521.52002	521.506158	521.558656
(**2.A.m**)_ $K_2S_2O_8$ S_0'	3737.498766	3737.150155	3737.112509	3737.2236
(**2.B*.m**)_ $K_2S_2O_8$, $\mathbf{T_1}$**'**	3737.426034	3737.080619	3737.042464	3737.156538
(**2.A.m**)_ $K_2S_2O_8$_**1a**, $\mathbf{T_1}$	4260.038301	4259.408437	4259.355687	4259.49964
sing	4260.100542	4259.467873	4259.415338	4259.558948
[**2.C.m**]_ $K_2S_2O_8$_1min, doub	3737.618466	3737.272547	3737.234512	3737.348769
quartet	3737.489247	3737.148696	3737.109624	3737.224997
**(T)–1**	4260.055298	4259.42552	4259.372532	4259.517803
**(S)–1**	4260.060305	4259.428837	4259.376186	4259.518369
[**2.F.m**]_ $K_2S_2O_8$_1min				
Singlet	3738.225214	3737.867193	3737.828621	3737.943193
Triplet	3738.150189	3737.795498	3737.756296	3737.873933
[**2.A.m**]_$HOSO_3$_K_2SO_4_1min				
	3738.352924	3737.993242	3737.955312	3738.066452

3.6 Optimized Structures and Energy Components for Copper-Mediated Reaction

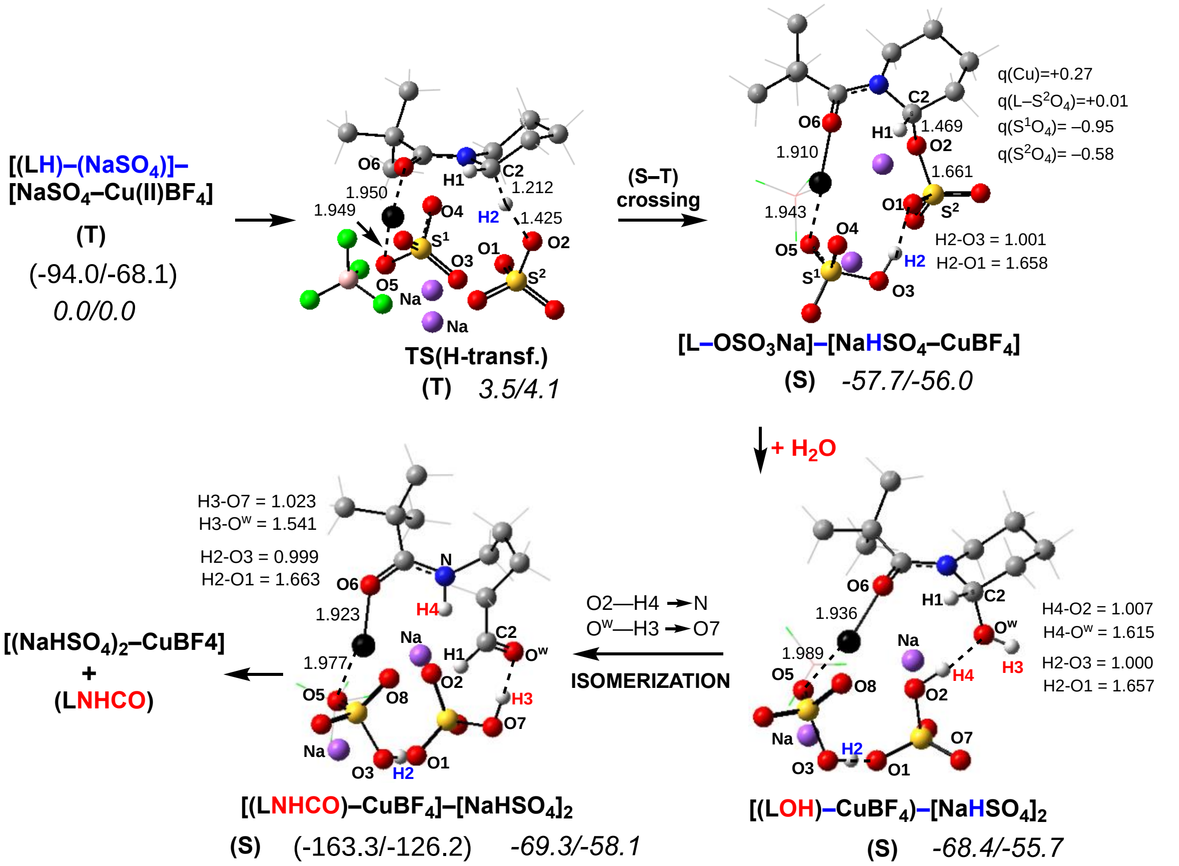

Figure S12. Computed relevant intermediates along with their geometry parameters (in Å) and relative energies (in kcal/mol) for the reaction of $CuBF_4$, N-Piv-piperidine (**1a** or **LH**) and sodium persulfate ($Na_2S_2O_8$).

Table S11. Calculated total electronic (E_{tot}), E_{tot} with zero-point energy corrections (E_{tot} + ZPEC), enthalpy (H), and Gibbs free energies (in Hartrees; all values are negative) of all reported structures for the reaction of $CuBF_4$, sodium-persulfate, and N-Piv-piperidine **1a**.

Str	$-E_{tot}$	$-(E_{tot}$ + ZPEC)	–H	–G
SO_4_min	699.1222694	699.108177	699.102801	699.13656
$HOSO_3$, 1min	699.7899769	699.76346	699.757571	699.791896
H_2O	76.4257436	76.40442	76.400641	76.422735
H_2SO_4	700.228991	700.190672	700.184452	700.219072
Na_2SO4	1023.859253	1023.84169	1023.832966	1023.875261
LH, chair	522.575576	522.29325	522.279314	522.331989
CuBF4	620.6138319	620.598672	620.591333	620.630662
$Na_2S_2O_8$	1722.821866	1722.786712	1722.772502	1722.828513
LH_$CuBF_4$	1143.251625	1142.951678	1142.92987	1143.004439
$Na_2S_2O_8$_$CuBF_4$	2343.502843	2343.451628	2343.429223	2343.505589
[(LH)–($NaSO_4$)]–[$NaSO_4$–$CuBF_4$]				

triplet	2866.165074	2865.829466	2865.792933	2865.899653
singlet	2866.134761	2865.798742	2865.761898	2865.871906
TS(H-transf)	2866.152814	2865.823397	2865.787345	2865.893068
[L–OSO_3Na]–[$NaHSO_4$–$CuBF_4$]				
singlet	2866.259207	2865.920586	2865.884829	2865.988907
[(LOH)–$CuBF_4$]–$NaHSO_4$]$_2$				
	2942.705094	2942.340241	2942.302495	2942.411233
[(LNHCO)–$CuBF_4$]–[$NaHSO_4$]$_2$				
singlet	2942.703819	2942.342931	2942.303953	2942.415055
LNHCO	597.8048282	597.521264	597.50508	597.563274
[($NaHSO_4$)$_2$–$CuBF_4$]				
	2344.846166	2344.771362	2344.748954	2344.824479

3.7 General Computational Considerations for Computations in Figure 13

All calculations were conducted using DFT[27] implemented in the Gaussian 09 suite[28] of ab initio quantum chemistry programs with Becke's three-parameter exchange functional B3LYP including Grimme's D3 dispersion correction with Becke-Johnson damping levels of theory.[13,14,16,29–32] Geometry optimizations were conducted using Pople's 6-31G(d,p) basis set for main group elements.[33–38] Copper was modeled using the Los Alamos ECP plus DZ basis (LANL2DZ) that includes relativistic effective core potentials.[10] The energies of the optimized structures were reevaluated by additional single-point calculations on each optimized geometry using the same functional and Pople's 6-311++G(d,p) basis set.[39–42] Copper was remodeled for single-point calculations using Stuttgart group effective core potential SDD.[43] Solvation effects were adopted at the same level of single-point calculations and at the self-consistent reaction field polarizable continuum model (IEF-PCM) with a dielectric constant of 78.3553 for water.[44] The Gibbs free energies were computed with the following equations.

$$G(sol) = \varepsilon(sol) + G(corr) \quad (1)$$
$$G(corr) = H(corr) - TS(tot) \quad (2)$$
$$H(corr) = E(tot) + k_BT \quad (3)$$
$$E(tot) = E(t)+E(r)+E(v)+E(e) \quad (4)$$
$$S(tot) = S(t)+S(r)+S(v)+S(e) \quad (5)$$
$$\Delta E(SCF) = \Sigma E(SCF) \text{ for products} - \Sigma E(SCF) \text{ for reactants} \quad (6)$$
$$\Delta G(sol) = \Sigma G(sol) \text{ for products} - \Sigma G(sol) \text{ for reactants} \quad (7)$$

E(sol) is the electronic energy in solution phase computed from the SCF (self-consisted field) procedure with the IEF-PCM calculations; G(sol) is the free energy in solution phase; G(corr) is the thermal correction to the free energy; T is the temperature (298.15 K); S(tot) is the entropy; E(tot) is the total internal thermal energy; E(t), E(r), E(v), and E(e) are the internal thermal energies from translation, rotation, vibration, and electronic motions, respectively; S(t), S(r), S(v), and S(e) are the entropies from translation, rotation, vibration, and electronic motions, respectively; The entropy we refer to is specifically of the solute(s), and the entropy of the solvent is implicitly comprised in the continuum solvation model.

3.8 Reaction Rate Comparison Between 11-TS and 11-TS’.

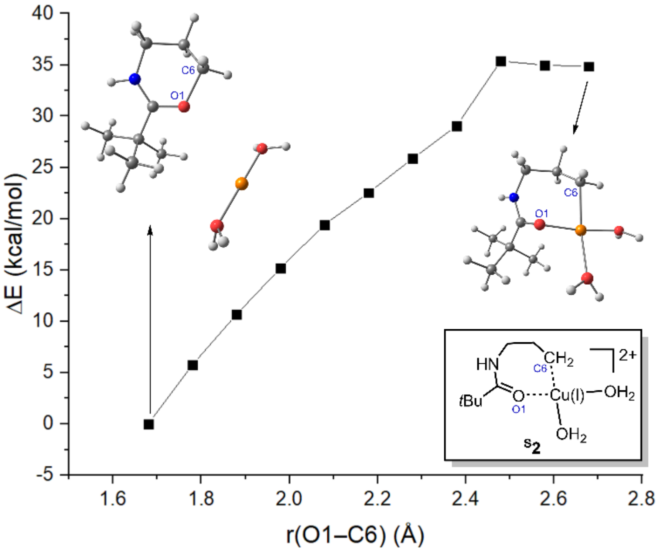

Figure S13. Electronic energy surface plot for the cyclization of **11** toward dihydro-oxazine (**10m**) with respect to the O1–C6 distance.

To investigate the reaction rate for the cyclization of **11** toward the cyclized product, dihydro-oxazine (**10m**), we have conducted a potential energy surface scan from **11** to **10m** with respect to the O1–C6 bond length as shown in Figure S13. However, despite our efforts with various methods, the transition state calculations for the cyclization did not converge. Instead, we assumed that the transition might require free energy of about 0.5 kcal/mol, which is the electronic energy difference between two points at r(O1–C6) = 2.68 Å (**11**) and at r(O1–C6) = 2.48 Å (**11-TS** in Figure S13). Because of this uncertainty, we denoted the ΔG(sol) for **11-TS** as ~–0.8 in Figure S13.

More significant is the relative reaction rate for the cyclization as compared to the rate of deprotonation. On the basis of transition state theory, the equilibrium constant for the quasi-equilibrium, $K^‡$, can be written as,

$$K^‡ = \exp(-\Delta G^‡/RT)$$

The ratio [**11-TS**]/[**11**] is then estimated to be $\exp(-(0.5\times4182\ \text{J})/(8.3145\ \text{J}\cdot\text{K}^{-1}\times298.15\ \text{K}))≒0.43$. Whereas, for the deprotonation, the ratio [**11-TS’**]/[**11**] is calculated to be $[H_2O]\times\exp(-(9.73\times4182\ \text{J})/(8.3145\ \text{J}\cdot\text{K}^{-1}\times298.15\ \text{K}))≒4.13\times10^{-6}$, where $[H_2O]$ = 55.6. The rate constant $k^‡$ for the bimolecular reaction is known to be generally much lower than that of the unimolecular reaction, we posit that the cyclization of **11** is at least 10^5 times faster than the deprotonation.

3.9 Energy Components for Optimized Structures in Figure 13

Table S12. Computed energy components for optimized structures.

	**E(sol)(SCF)/(Hartree)**	**Thermal Correction to G/(Hartree)**
	B3LYP-D3<br>/6-311++G(d,p)/SDD	B3LYP-D3<br>/6-31G(d,p)/LANL2DZ
**$Cu^{I}(H_2O)_2$**	-350.17225	0.01968
**H_2O**	-76.26704	0.00371
**H_3O^+**	-76.85586	0.01528
**CO**	-113.35032	-0.01411
**11**	-794.62020	0.23612
**11-TS'**	-871.08488	0.25297
**12**	-794.25191	0.22776
**olefin**	-444.04010	0.18358
**10m**	-444.48969	0.20236

4. X-ray Crystallographic Data

4.1 X-ray Analysis of 10m

A colorless block 0.12 x 0.08 x 0.05 mm in size was mounted on a Cryoloop with Paratone oil. Data were collected in a nitrogen gas stream at 100(2) K using omega scans. Crystal-to-detector distance was 30.23 mm and exposure time was 0.50 seconds per frame at low and 2.00 seconds at high angles, using a scan width of 0.5°. Data collection was 100% complete to 74.000° in θ. A total of 61459 reflections were collected covering the indices -21<=h<=21, -21<=k<=21, -8<=l<=8. 1204 reflections were found to be symmetry independent, with an R_{int} of 0.0652. Indexing and unit cell refinement indicated a primitive, tetragonal lattice. The space group was found to be P 42/m b c (No. 135). The data were integrated using the CrysAlis^{Pro} 1.171.40.68a software program and scaled using the SCALE3 ABSPACK scaling algorithm. Solution by intrinsic phasing (SHELXT-2015) produced a heavy-atom phasing model consistent with the proposed structure. All non-hydrogen atoms were refined anisotropically by full-matrix least-squares (SHELXL-2014). All hydrogen atoms were placed using a riding model. Their positions were constrained relative to their parent atom using the appropriate HFIX command in SHELXL-2014.

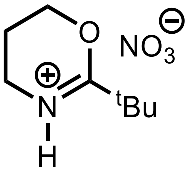

10m

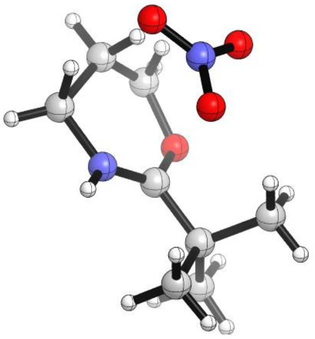

This crystal structure has been deposited at the Cambridge Crystallographic Data Center under CCDC 2128702.

Table S13. Crystal data and structure refinement for JRoque_David.

Identification code	JRoque_David	
Empirical formula	C8 H16 N2 O4	
Formula weight	204.23	
Temperature	150(2) K	
Wavelength	1.54184 Å	
Crystal system	Tetragonal	
Space group	P 42/m b c	
Unit cell dimensions	a = 17.20020(10) Å	a= 90°.
	b = 17.20020(10) Å	b= 90°.
	c = 6.94210(10) Å	g = 90°.
Volume	2053.80(4) Å^3	
Z	8	
Density (calculated)	1.321 Mg/m^3	
Absorption coefficient	0.893 mm^{-1}	
F(000)	880	
Crystal size	0.120 x 0.080 x 0.050 mm^3	
Theta range for data collection	3.634 to 79.180°.	
Index ranges	-21<=h<=21, -21<=k<=21, -8<=l<=8	
Reflections collected	61459	

Independent reflections	1204 [R(int) = 0.0652]
Completeness to theta = 74.000°	99.8 %
Absorption correction	Semi-empirical from equivalents
Max. and min. transmission	1.00000 and 0.55325
Refinement method	Full-matrix least-squares on F^2
Data / restraints / parameters	1204 / 0 / 99
Goodness-of-fit on F^2	1.175
Final R indices [I>2sigma(I)]	R1 = 0.0598, wR2 = 0.1451
R indices (all data)	R1 = 0.0608, wR2 = 0.1457
Extinction coefficient	n/a
Largest diff. peak and hole	0.278 and -0.359 e.Å^{-3}

Table S14. Atomic coordinates (x 10^4) and equivalent isotropic displacement parameters (Å^{2}x 10^3) for jroque_david. U(eq) is defined as one third of the trace of the orthogonalized U^{ij} tensor.

	x	y	z	U(eq)
O(1)	7080(1)	5330(1)	5000	34(1)
N(1)	8120(1)	4507(1)	5000	33(1)
N(2)	8130(2)	4606(1)	0	43(1)
O(4)	8791(1)	4837(1)	0	73(1)
O(2)	8016(1)	3891(1)	0	87(1)
C(4)	7382(1)	4630(1)	5000	28(1)
O(3)	7588(2)	5080(1)	0	83(1)
C(7)	8711(2)	5137(2)	5000	41(1)
C(2)	6768(1)	3996(2)	5000	34(1)
C(1)	7127(2)	3191(2)	5000	47(1)
C(3)	6268(2)	4101(1)	3207(5)	67(1)
C(6)	8324(2)	5882(2)	4215(6)	37(1)
C(5)	7600(2)	5995(2)	5361(10)	36(2)

Table S15. Bond lengths [Å] and angles [°] for jroque_david.

O(1)-C(4)	1.310(3)
O(1)-C(5)	1.474(4)
N(1)-C(4)	1.286(3)
N(1)-C(7)	1.485(3)
N(1)-H(1)	0.88(4)
N(2)-O(4)	1.205(3)
N(2)-O(3)	1.239(3)
N(2)-O(2)	1.246(3)
C(4)-C(2)	1.518(3)
C(7)-C(6)	1.544(4)
C(7)-H(7A)	0.9900
C(7)-H(7B)	0.9900
C(2)-C(1)	1.517(4)
C(2)-C(3)	1.523(3)
C(2)-C(3)#1	1.523(3)
C(1)-H(1A)	0.99(3)
C(1)-H(1B)	1.02(3)
C(3)-H(3A)	0.9800
C(3)-H(3B)	0.9800
C(3)-H(3C)	0.9800
C(6)-C(5)	1.490(6)

C(6)-H(6A)	0.9900
C(6)-H(6B)	0.9900
C(5)-H(5A)	0.9900
C(5)-H(5B)	0.9900
C(4)-O(1)-C(5)	118.2(2)
C(4)-N(1)-C(7)	123.7(2)
C(4)-N(1)-H(1)	124(2)
C(7)-N(1)-H(1)	112(2)
O(4)-N(2)-O(3)	119.6(3)
O(4)-N(2)-O(2)	118.3(3)
O(3)-N(2)-O(2)	122.1(3)
N(1)-C(4)-O(1)	122.8(2)
N(1)-C(4)-C(2)	124.6(2)
O(1)-C(4)-C(2)	112.5(2)
N(1)-C(7)-C(6)	108.1(2)
N(1)-C(7)-H(7A)	110.1
C(6)-C(7)-H(7A)	110.1
N(1)-C(7)-H(7B)	110.1
C(6)-C(7)-H(7B)	110.1
H(7A)-C(7)-H(7B)	108.4
C(1)-C(2)-C(4)	111.9(2)
C(1)-C(2)-C(3)	109.71(16)
C(4)-C(2)-C(3)	107.95(15)
C(1)-C(2)-C(3)#1	109.71(16)
C(4)-C(2)-C(3)#1	107.95(15)
C(3)-C(2)-C(3)#1	109.6(3)
C(2)-C(1)-H(1A)	107.1(19)
C(2)-C(1)-H(1B)	110.9(13)
H(1A)-C(1)-H(1B)	109.4(16)
C(2)-C(3)-H(3A)	109.5
C(2)-C(3)-H(3B)	109.5
H(3A)-C(3)-H(3B)	109.5
C(2)-C(3)-H(3C)	109.5
H(3A)-C(3)-H(3C)	109.5
H(3B)-C(3)-H(3C)	109.5
C(5)-C(6)-C(7)	106.3(3)
C(5)-C(6)-H(6A)	110.5
C(7)-C(6)-H(6A)	110.5
C(5)-C(6)-H(6B)	110.5
C(7)-C(6)-H(6B)	110.5
H(6A)-C(6)-H(6B)	108.7
O(1)-C(5)-C(6)	108.3(3)
O(1)-C(5)-H(5A)	110.0
C(6)-C(5)-H(5A)	110.0
O(1)-C(5)-H(5B)	110.0
C(6)-C(5)-H(5B)	110.0
H(5A)-C(5)-H(5B)	108.4

Symmetry transformations used to generate equivalent atoms:
#1 x,y,-z+1

Table S16. Anisotropic displacement parameters (Å^{2}x 10^3)for jroque_david. The anisotropic displacement factor exponent takes the form: -2p^2[h^{2}a*^{2}U^{11} + ... + 2 h k a* b* U^{12}]

	U^{11}	U^{22}	U^{33}	U^{23}	U^{13}	U^{12}
O(1)	26(1)	21(1)	56(1)	0	0	-2(1)
N(1)	25(1)	23(1)	51(1)	0	0	-1(1)
N(2)	41(1)	26(1)	61(2)	0	0	6(1)
O(4)	39(1)	39(1)	143(3)	0	0	-3(1)
O(2)	44(1)	26(1)	191(4)	0	0	4(1)
C(4)	26(1)	22(1)	37(1)	0	0	-2(1)
O(3)	51(2)	36(1)	162(3)	0	0	18(1)
C(7)	24(1)	28(1)	71(2)	0	0	-6(1)
C(2)	23(1)	23(1)	54(2)	0	0	-4(1)
C(1)	31(1)	23(1)	87(3)	0	0	-3(1)
C(3)	62(2)	42(1)	96(2)	11(1)	-41(2)	-18(1)
C(6)	35(2)	26(2)	49(2)	3(2)	-2(2)	-6(1)
C(5)	35(2)	21(1)	53(6)	-3(2)	0(2)	-7(1)

Table S17. Hydrogen coordinates (x 10^4) and isotropic displacement parameters (Å^{2}x 10^3) for jroque_david.

	x	y	z	U(eq)
H(7A)	9157	4989	4176	49
H(7B)	8904	5226	6325	49
H(3A)	6001	4604	3268	100
H(3B)	5883	3682	3142	100
H(3C)	6599	4084	2059	100
H(6A)	8675	6334	4370	44
H(6B)	8198	5822	2831	44
H(5A)	7727	6027	6749	44
H(5B)	7341	6485	4975	44
H(1A)	6692(19)	2813(19)	5000	40(9)
H(1B)	7459(15)	3108(13)	3810(40)	58(8)
H(1)	8330(20)	4050(20)	5000	48(10)

Table S18. Torsion angles [°] for jroque_david.

C(7)-N(1)-C(4)-O(1)	0.0
C(7)-N(1)-C(4)-C(2)	180.0
C(5)-O(1)-C(4)-N(1)	-11.1(3)
C(5)-O(1)-C(4)-C(2)	168.9(3)
C(4)-N(1)-C(7)-C(6)	-21.79(18)
N(1)-C(4)-C(2)-C(1)	0.0
O(1)-C(4)-C(2)-C(1)	180.0
N(1)-C(4)-C(2)-C(3)	-120.80(18)
O(1)-C(4)-C(2)-C(3)	59.20(18)
N(1)-C(4)-C(2)-C(3)#1	120.80(18)
O(1)-C(4)-C(2)-C(3)#1	-59.20(18)
N(1)-C(7)-C(6)-C(5)	51.9(3)
C(4)-O(1)-C(5)-C(6)	44.2(5)
C(7)-C(6)-C(5)-O(1)	-63.1(4)

Symmetry transformations used to generate equivalent atoms:
#1 x,y,-z+1

5. NMR Spectral Data

Pivaloyl Protected Cyclic Amines

2,2-dimethyl-1-(3-methylpiperidin-1-yl)propan-1-one

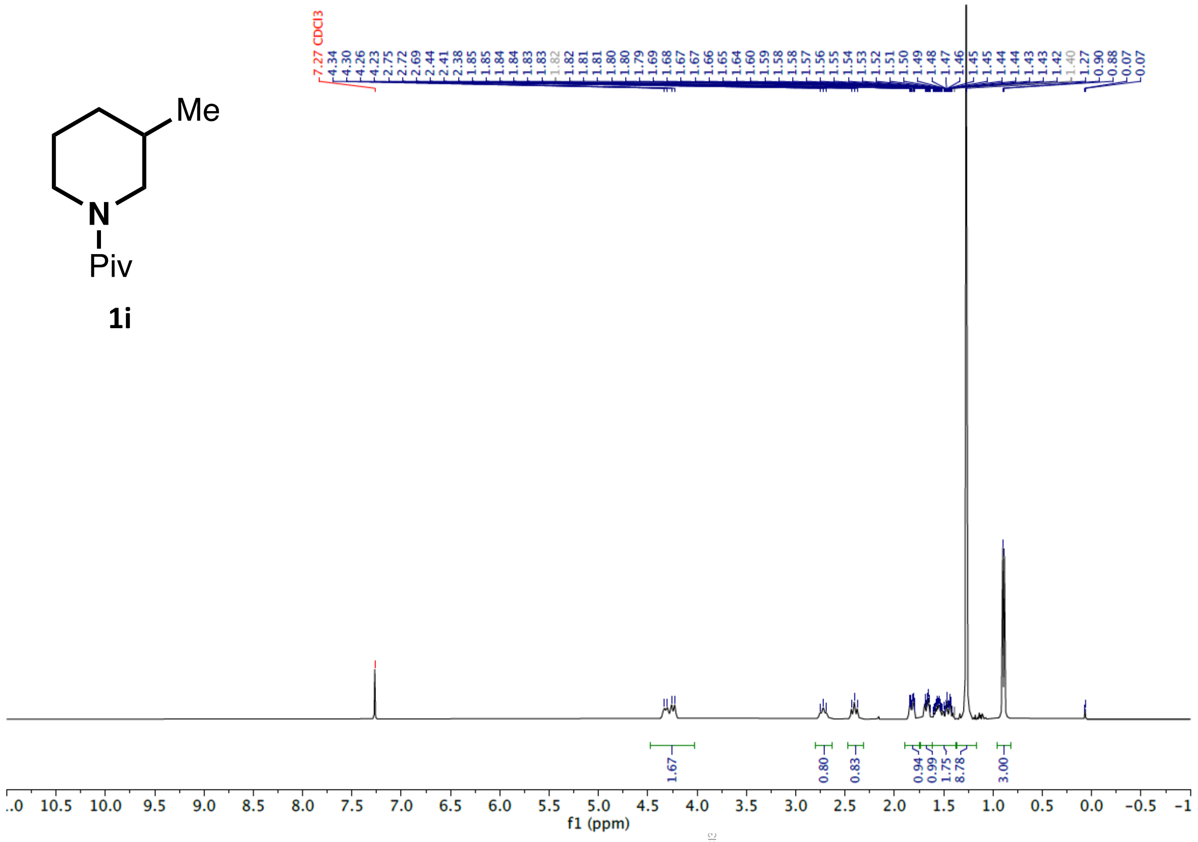

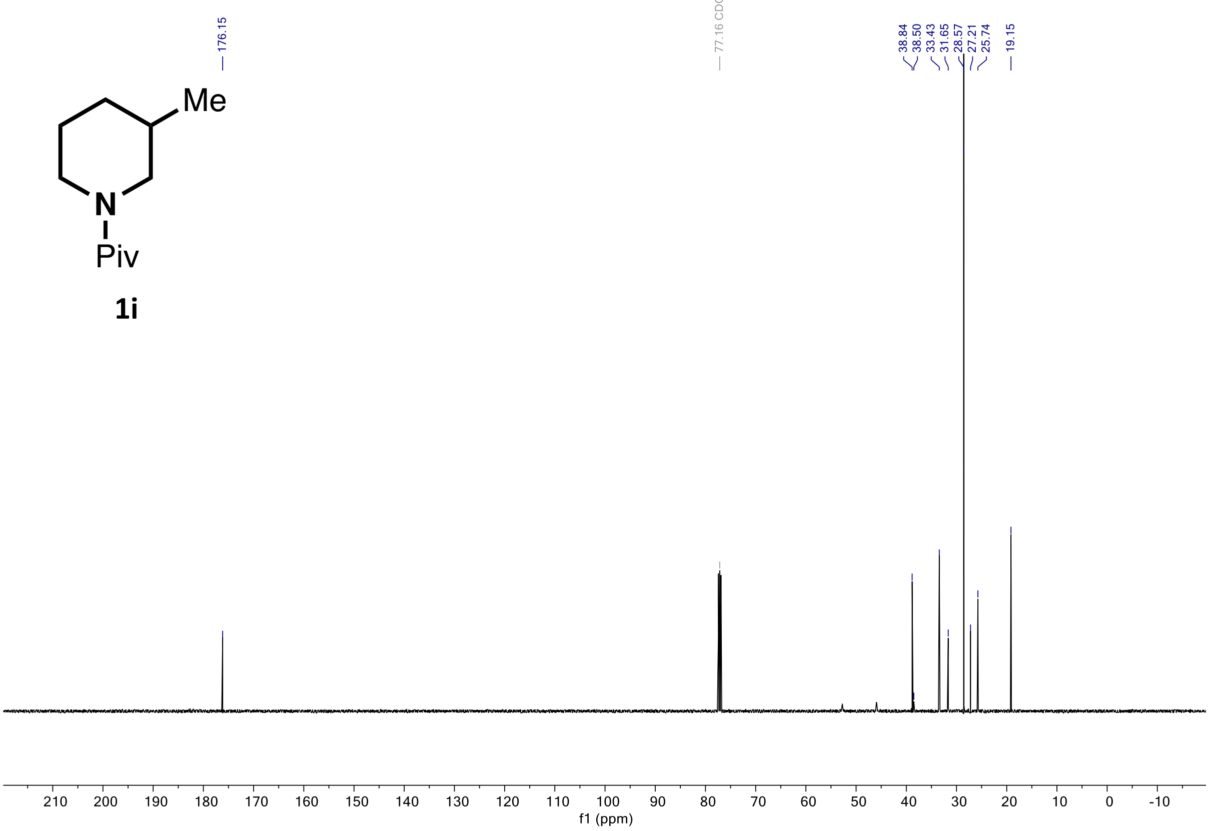

1-(2-ethylpiperidin-1-yl)-2,2-dimethylpropan-1-one

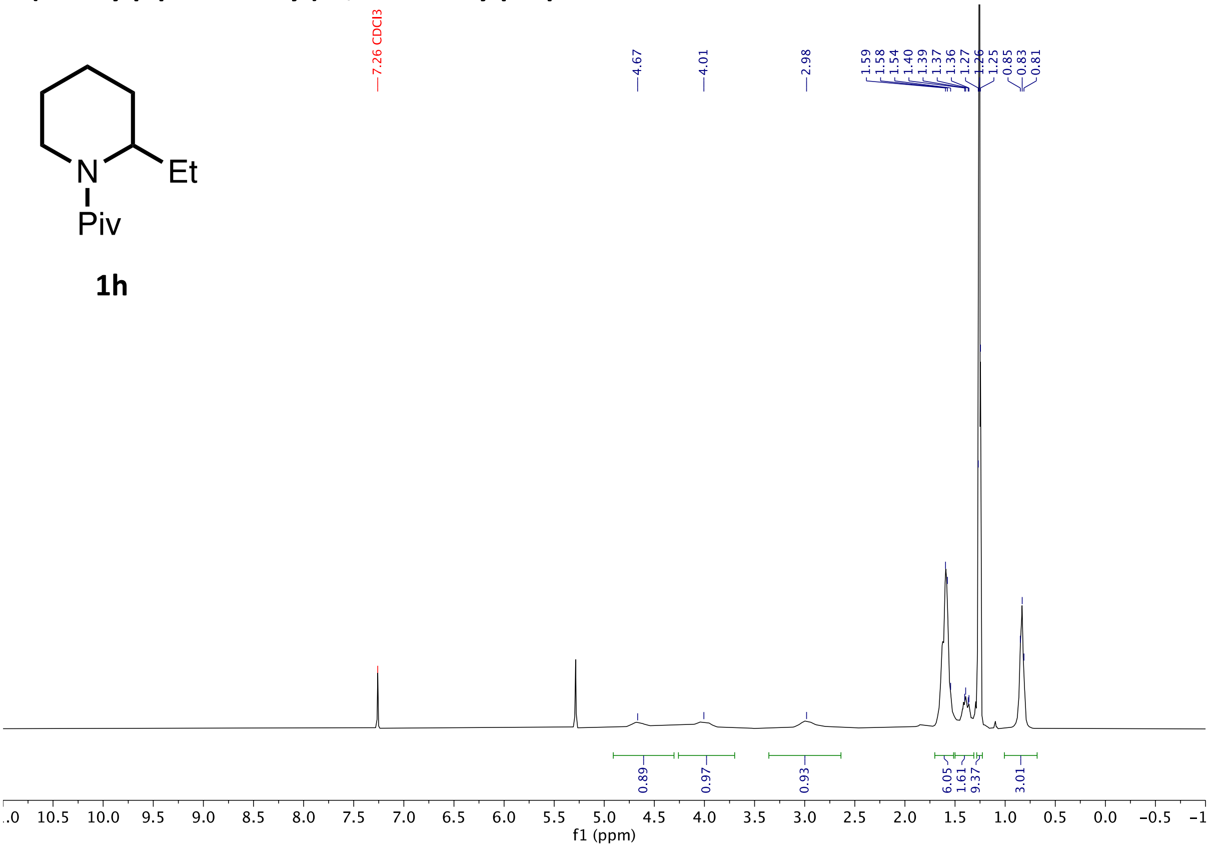

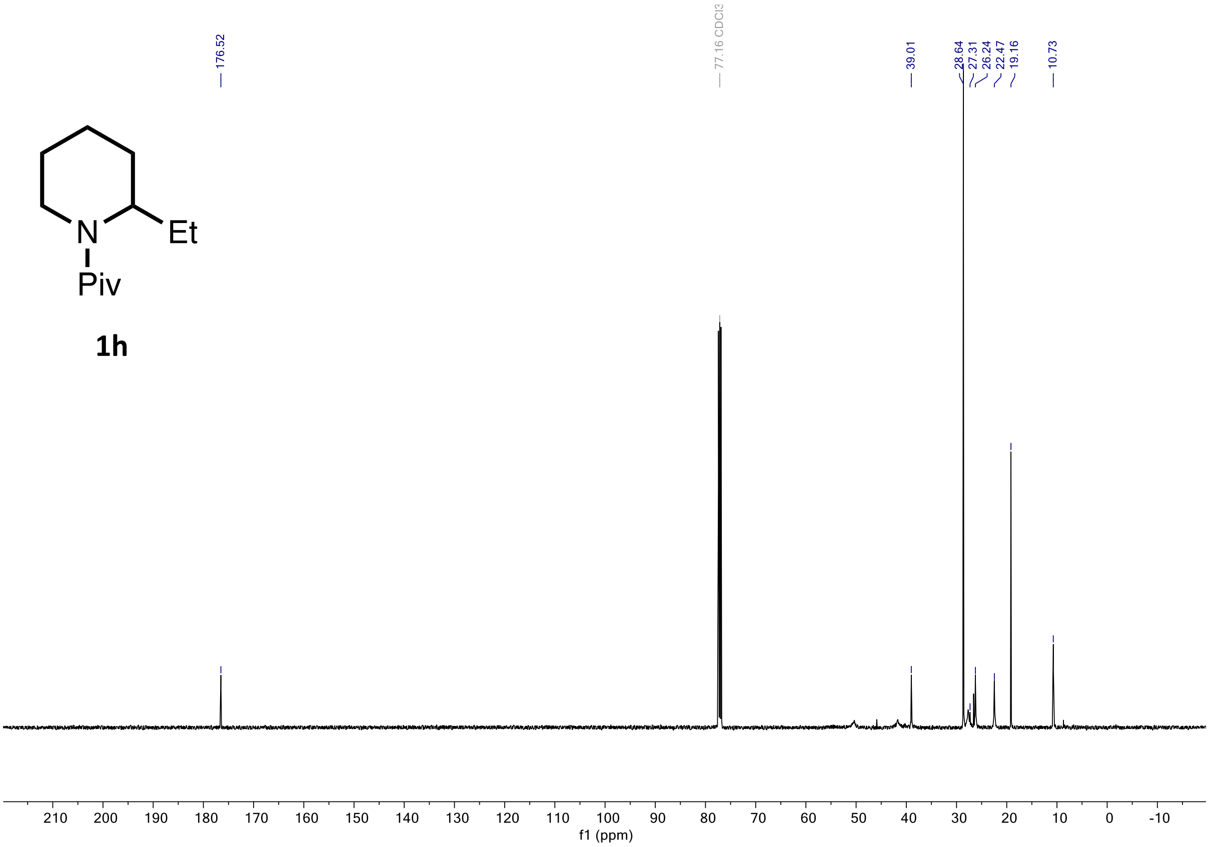

2,2-dimethyl-1-(3-phenylpiperidin-1-yl)propan-1-one

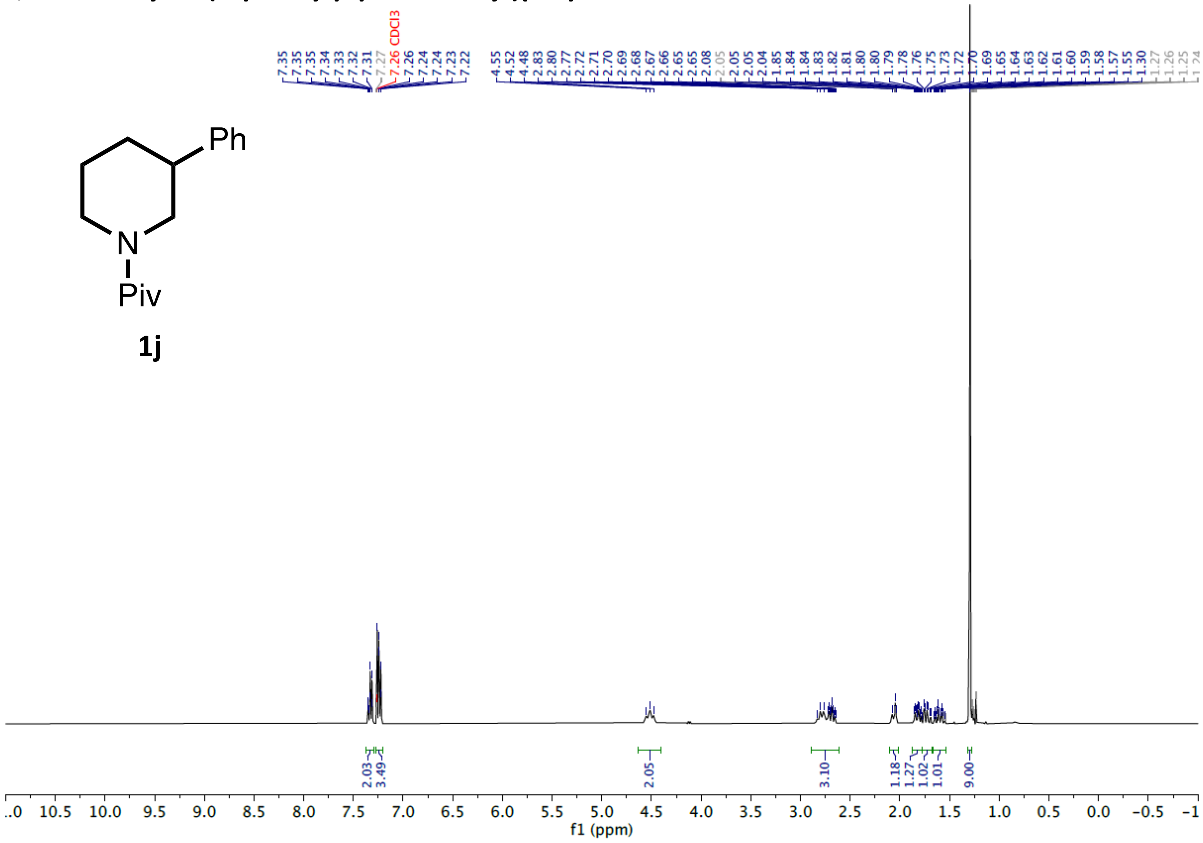

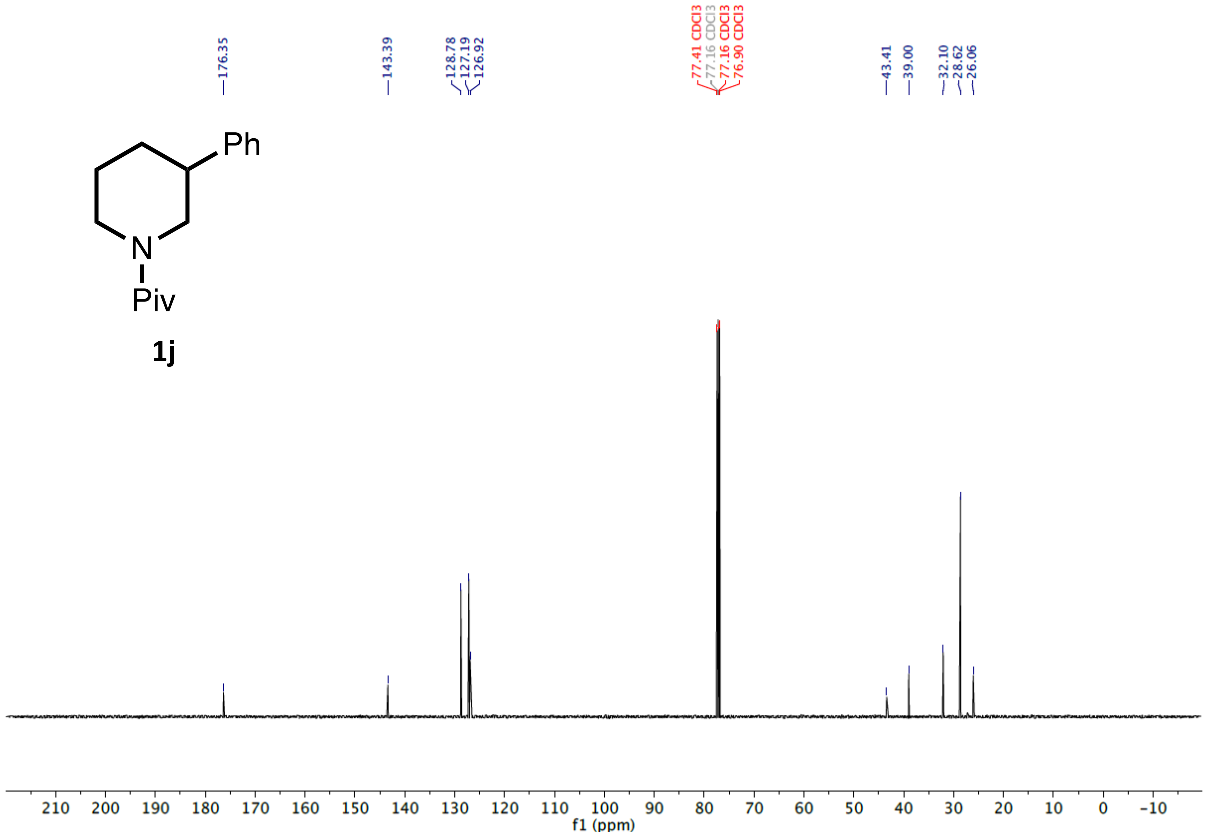

1-(4-(4-chlorophenyl)piperidin-1-yl)-2,2-dimethylpropan-1-one

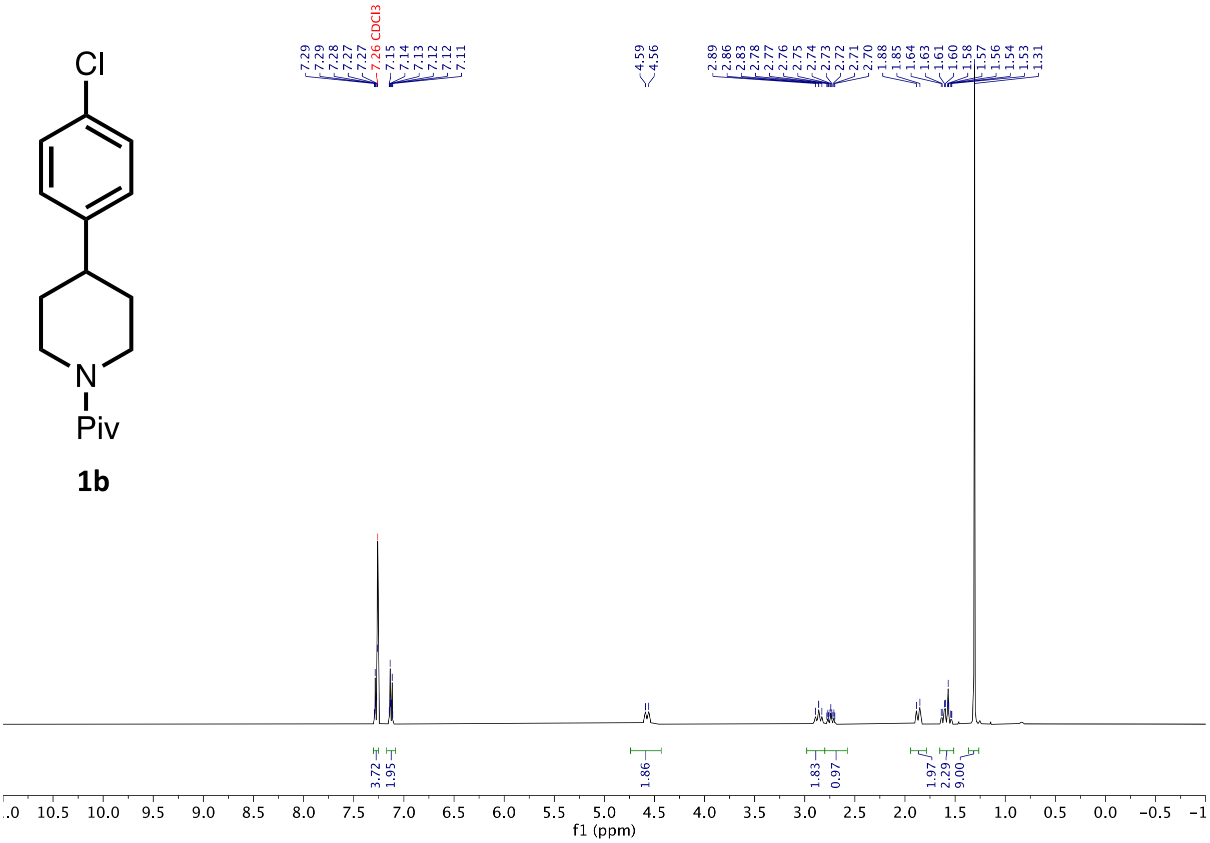

1b

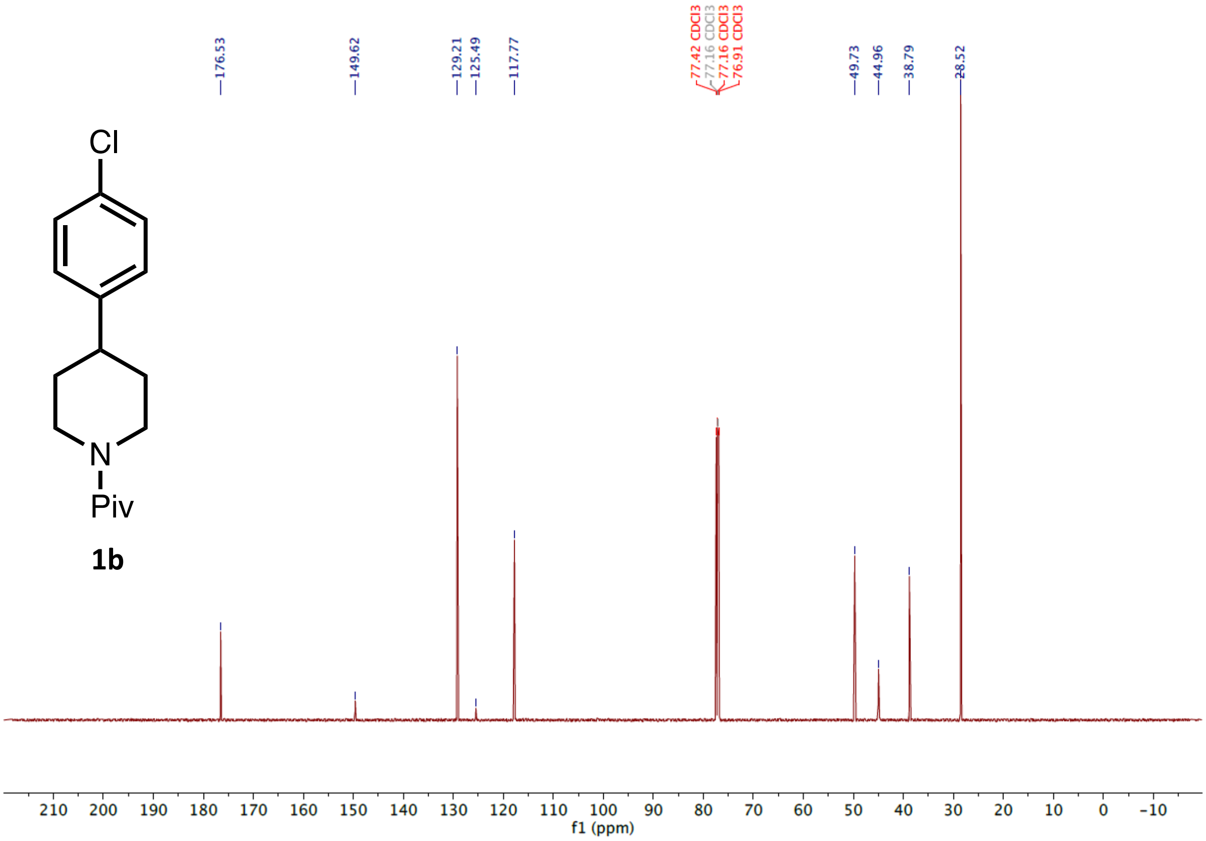

1b

1-(6,7-dimethoxy-3,4-dihydroisoquinolin-2(1*H*)-yl)-2,2-dimethylpropan-1-one

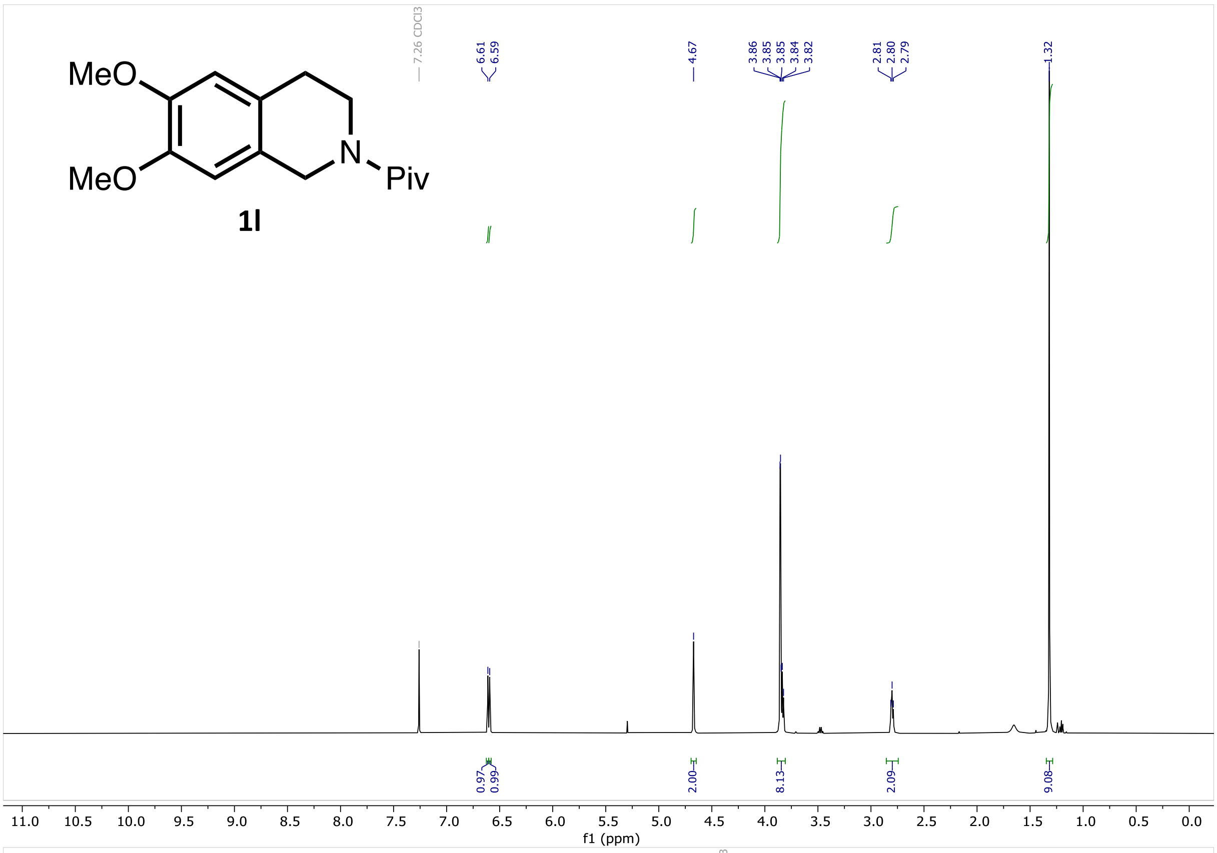

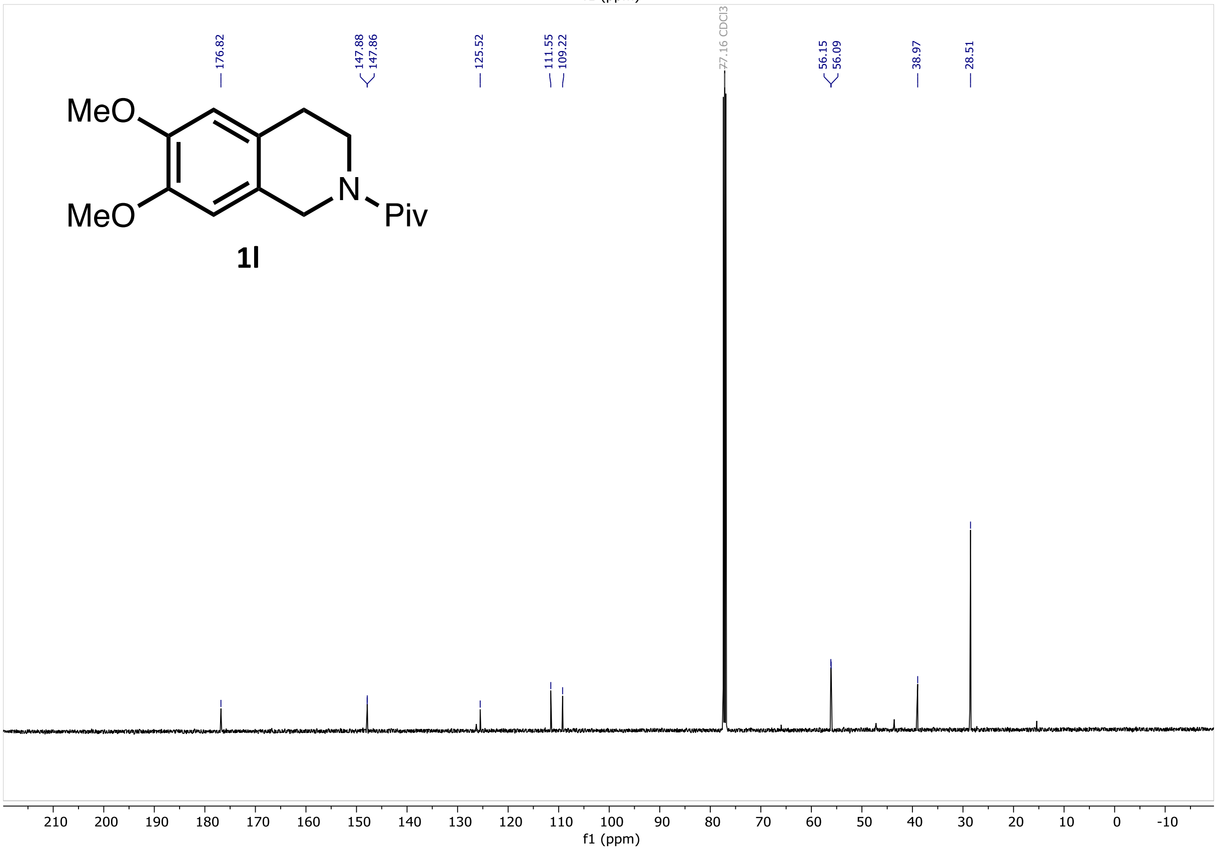

2,2-dimethyl-1-(piperidin-1-yl-d_{10})propan-1-one

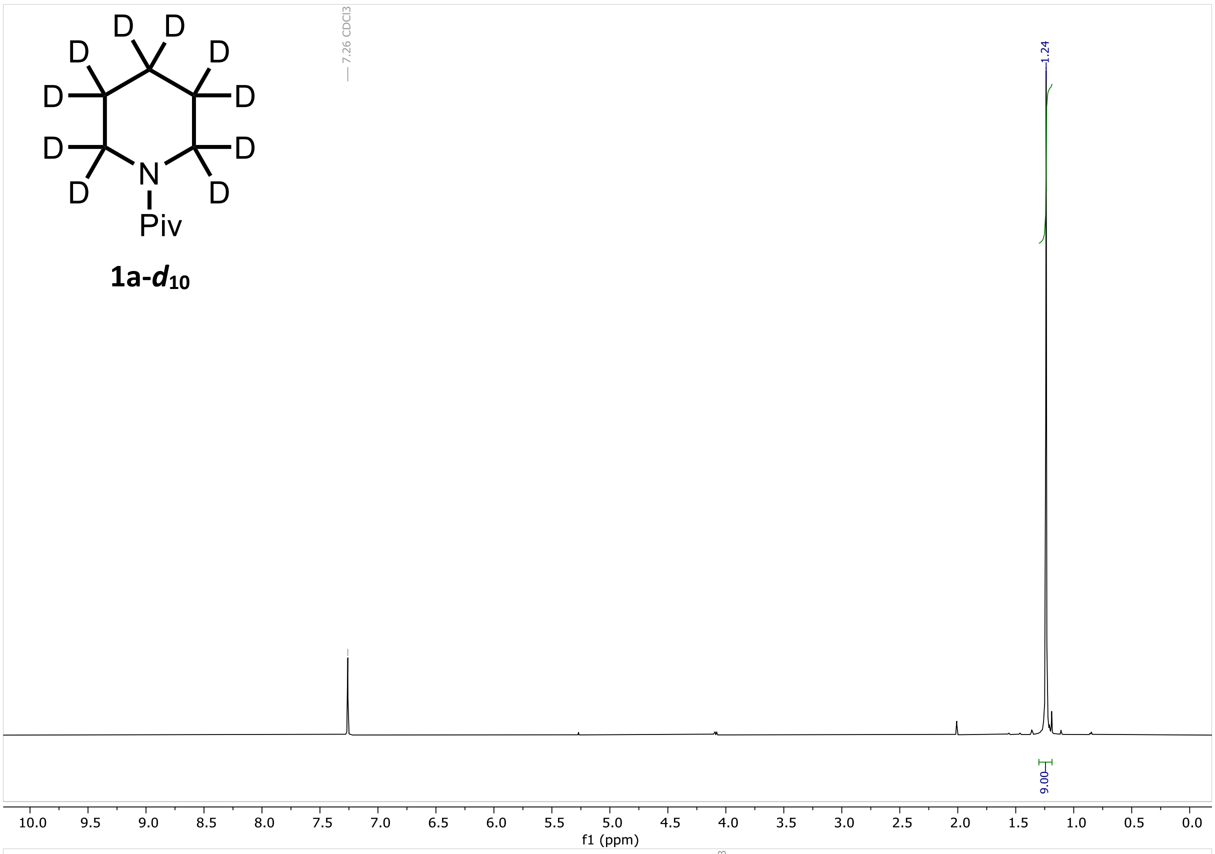

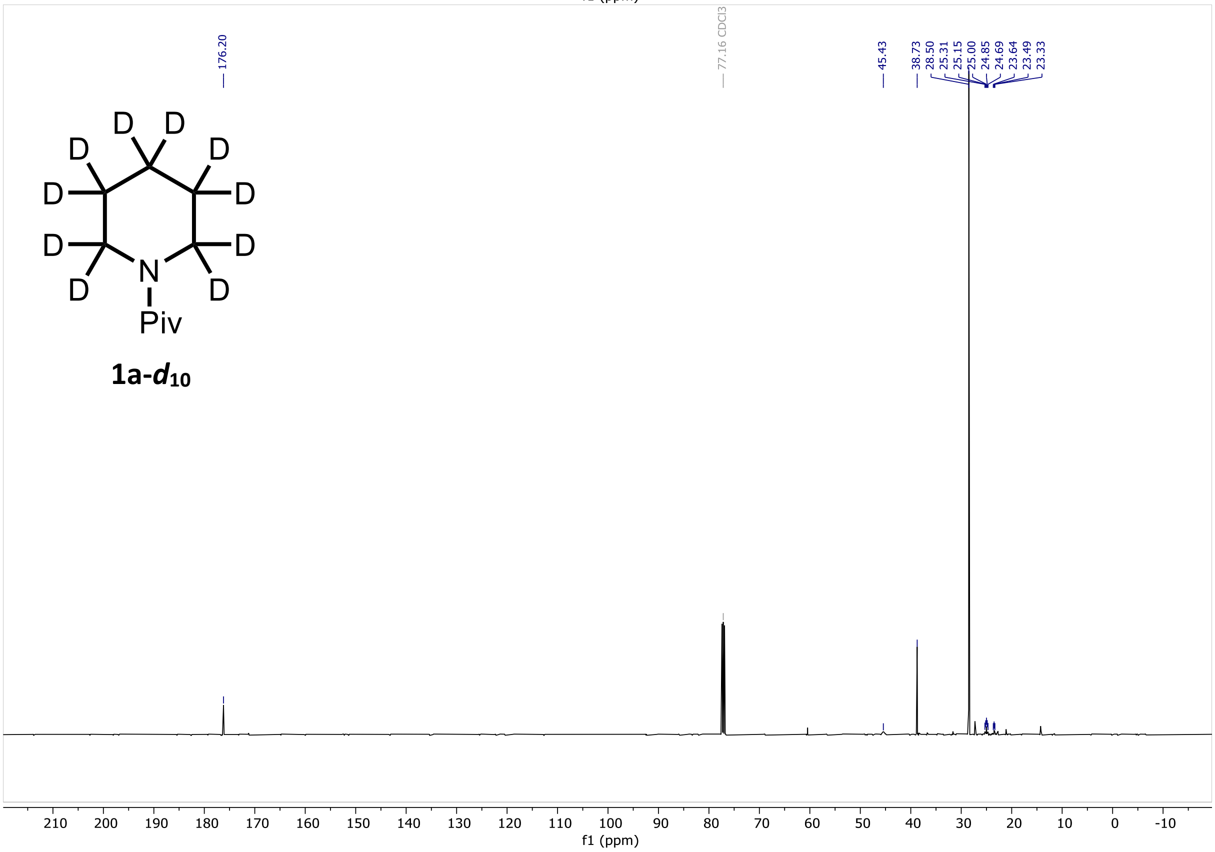

NMR Spectra – Photocatalyst Screening

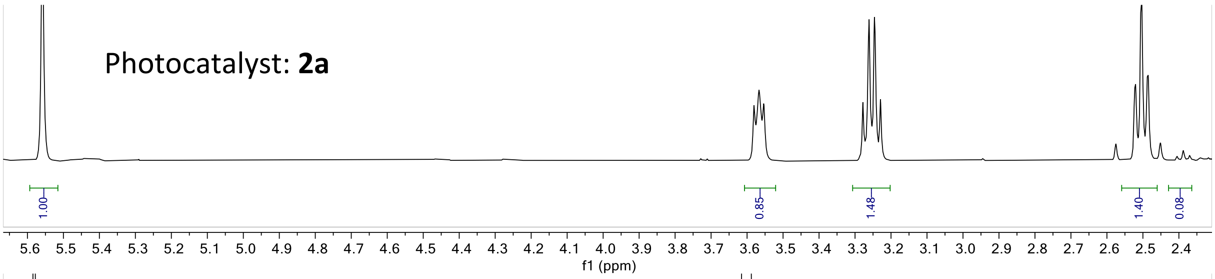

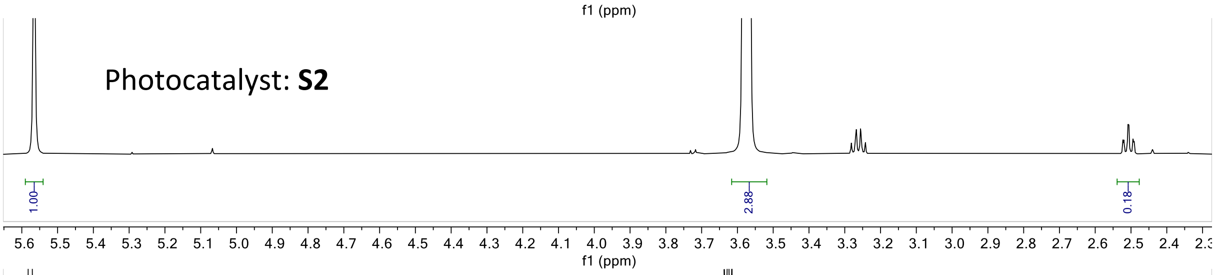

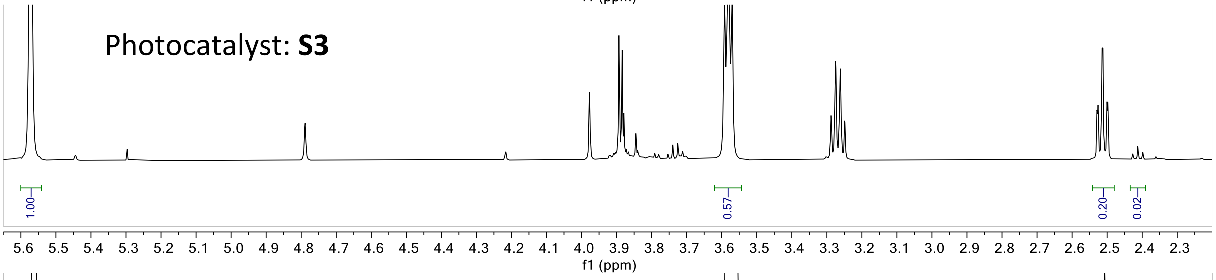

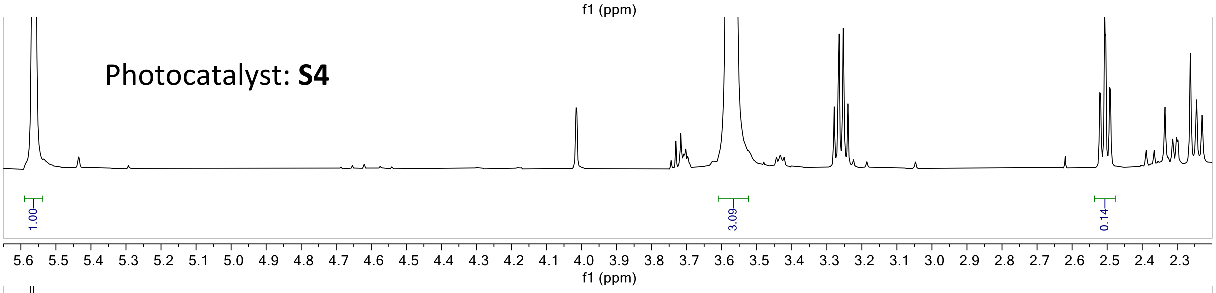

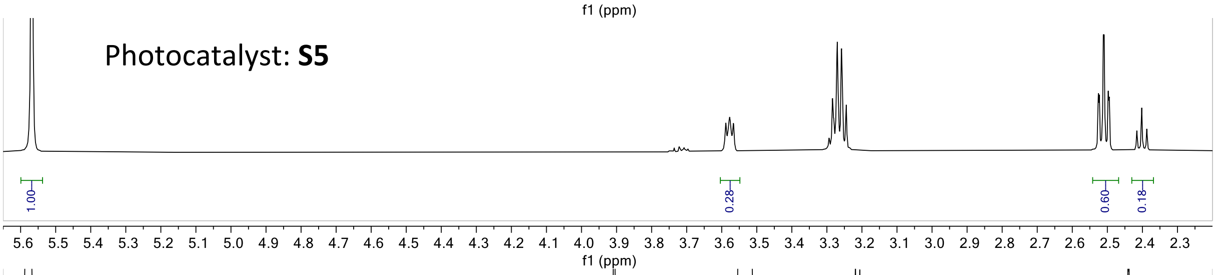

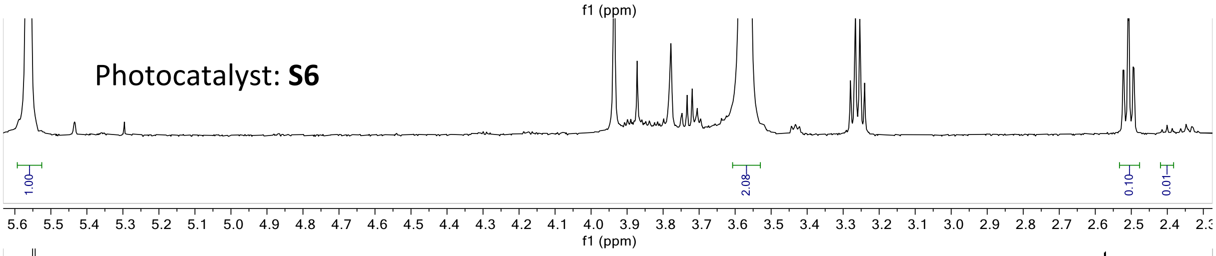

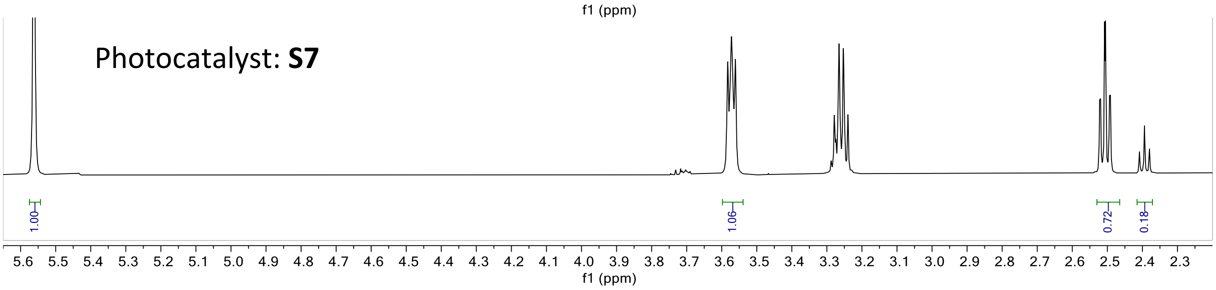

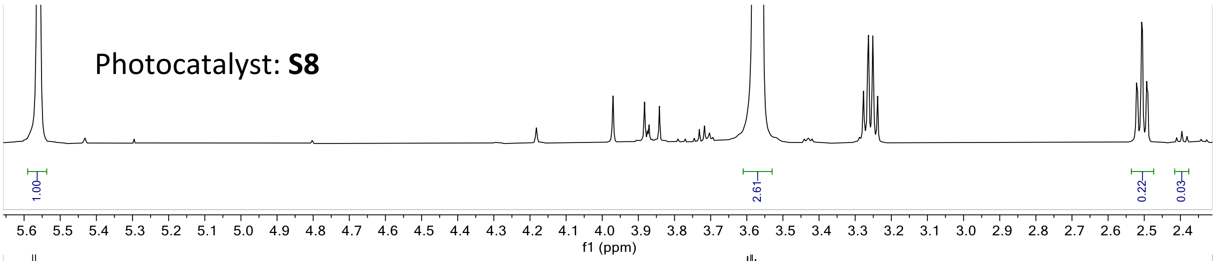
Photocatalyst: S8
1.00
2.61
0.22
0.03
f1 (ppm)

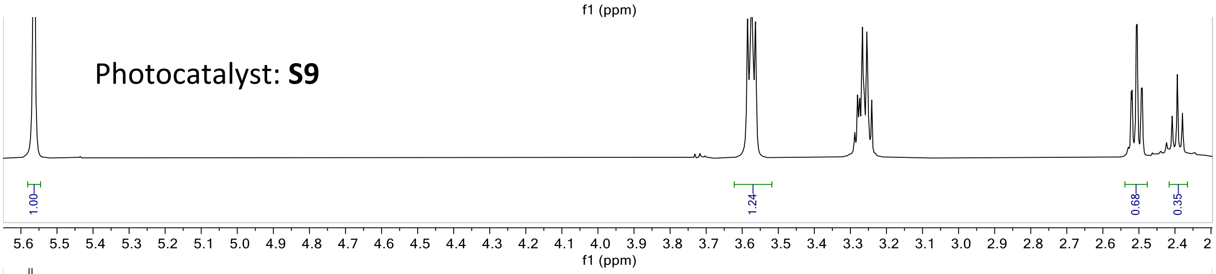
Photocatalyst: S9
1.00
1.24
0.68
0.35
f1 (ppm)

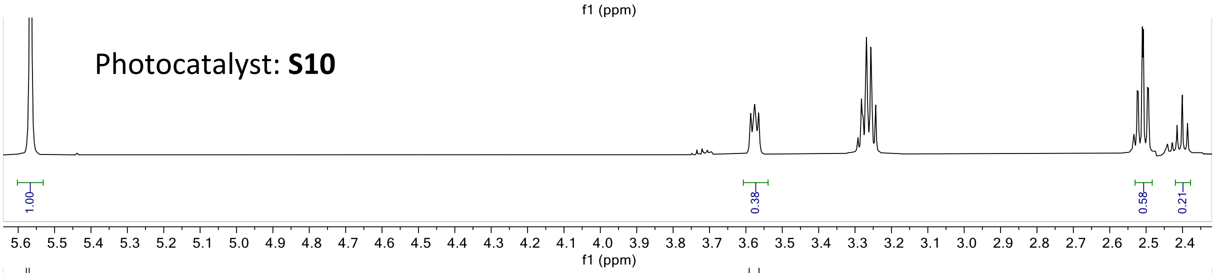
Photocatalyst: S10
1.00
0.38
0.58
0.21
f1 (ppm)

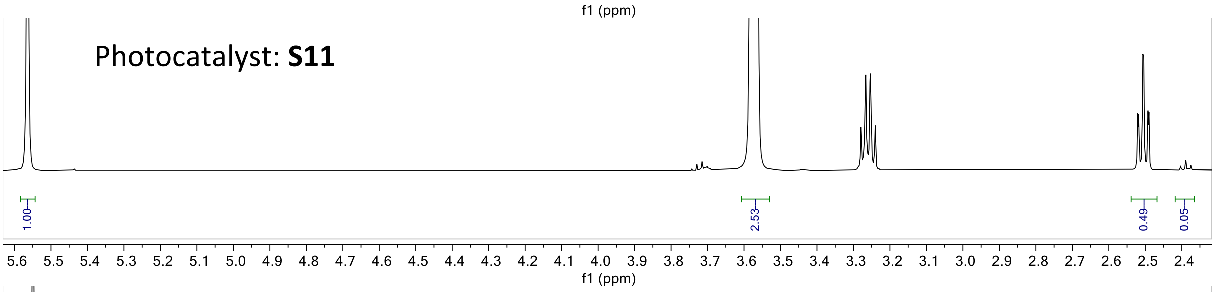
Photocatalyst: S11
1.00
2.53
0.49
0.05
f1 (ppm)

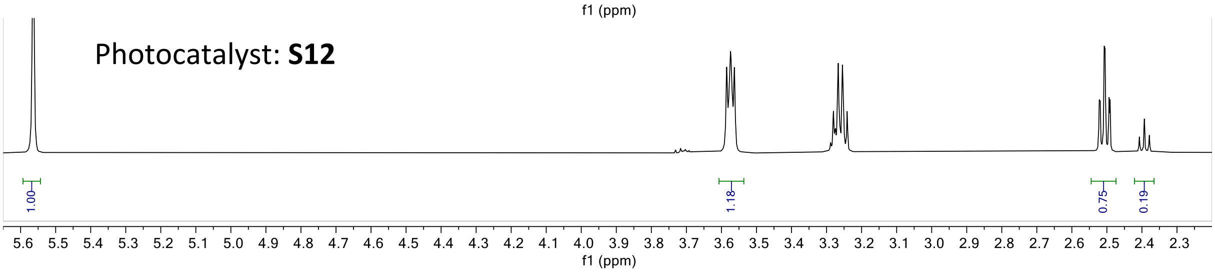
Photocatalyst: S12
1.00
1.18
0.75
0.19
f1 (ppm)

NMR Spectra – Reaction Optimization

Optimization of Reaction Time

For spectra of catalyst **S9** and **2a** for 60 min reaction time, see pg. S69.

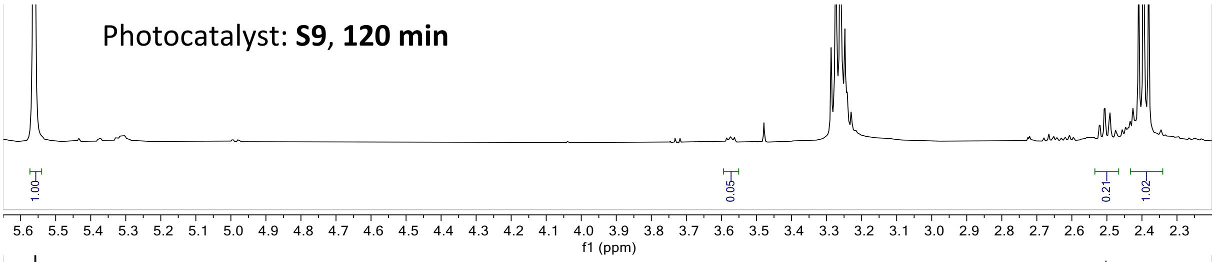

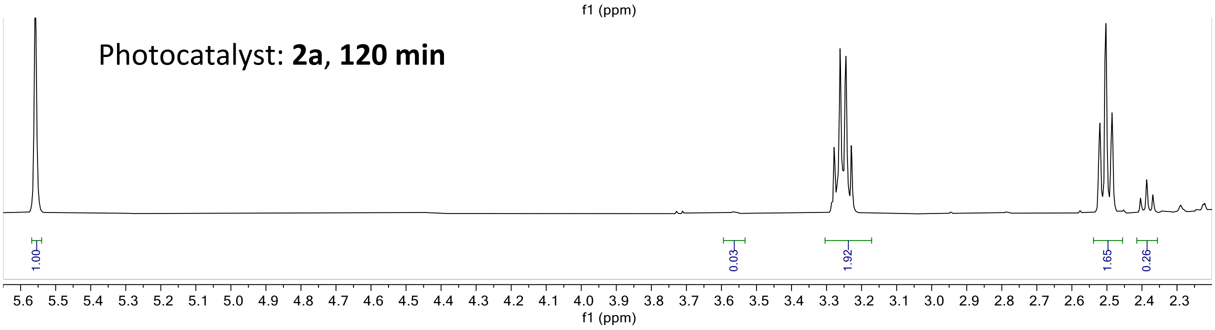

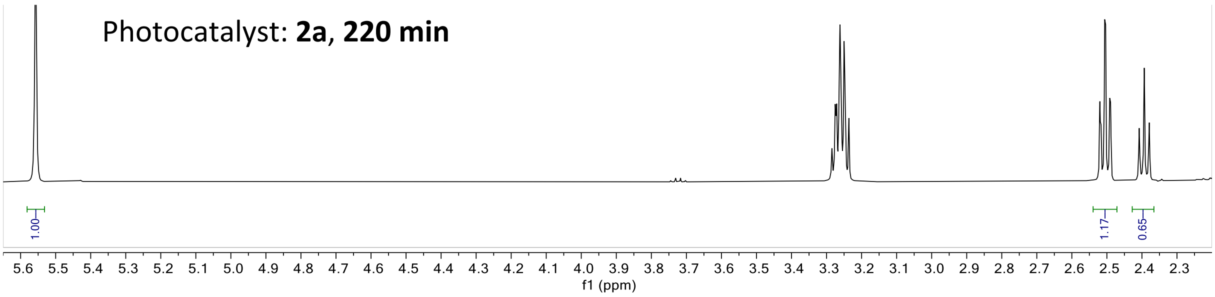

Optimization of Catalyst Loading

For spectrum of catalyst **2a** for 5 mol% catalyst loading, see pg. S71.

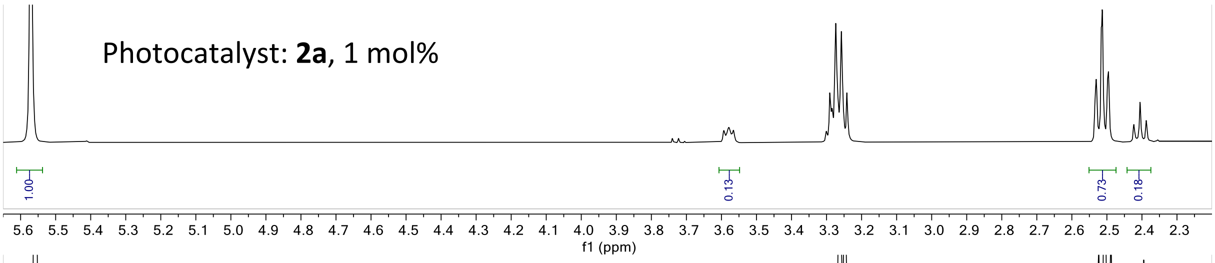

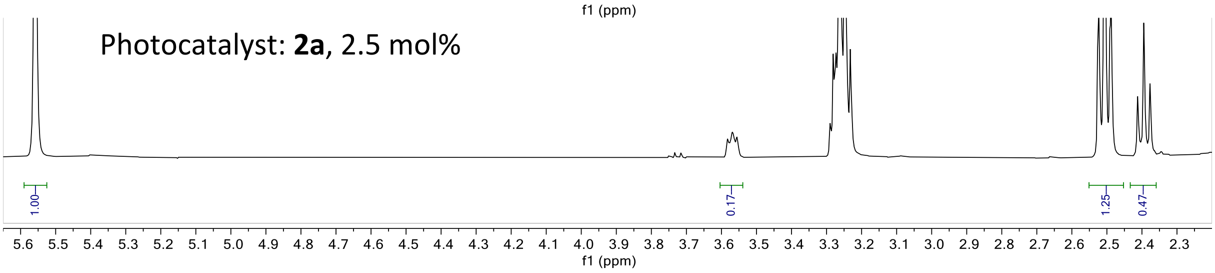

Optimization of Oxidant and Solvent

For spectrum of catalyst **2a** and 3 equiv. $K_2S_2O_8$, see pg. S71.

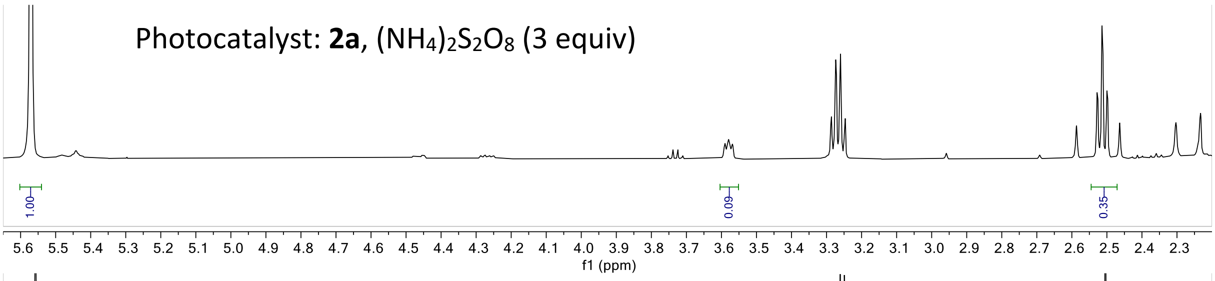

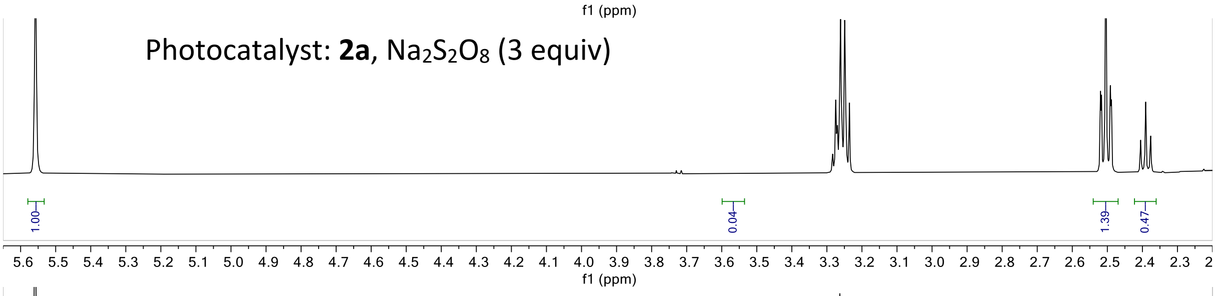

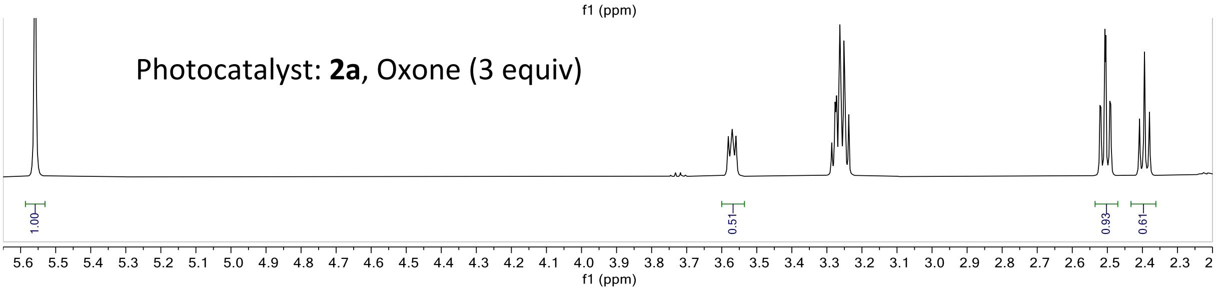

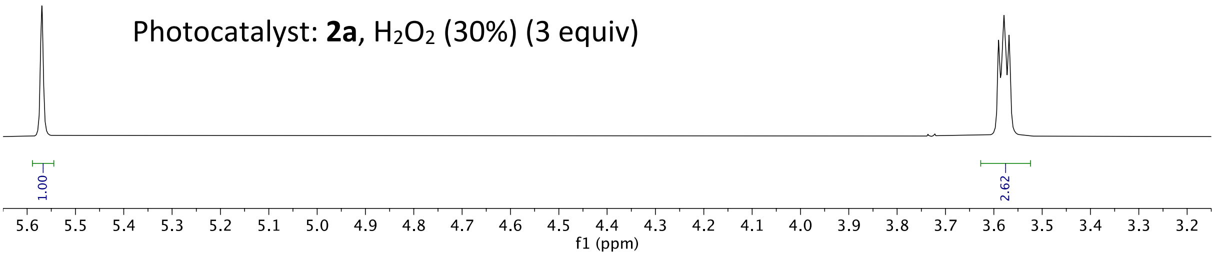

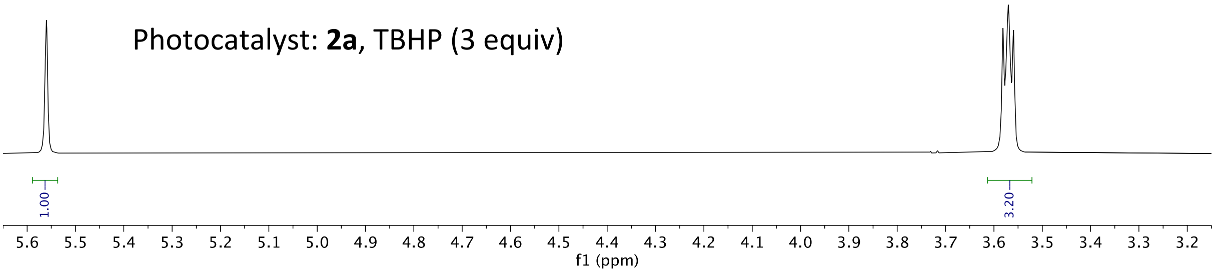

For spectrum of catalyst **2a** in 1:1 acetonitrile/water, see pg. S71.

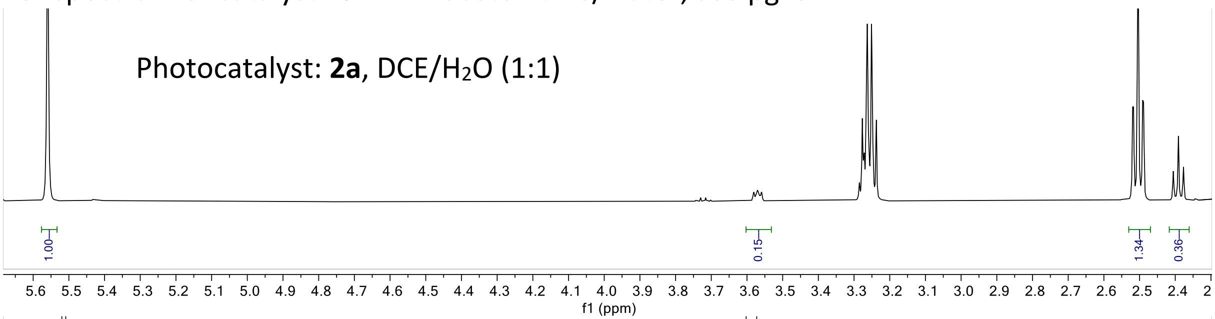

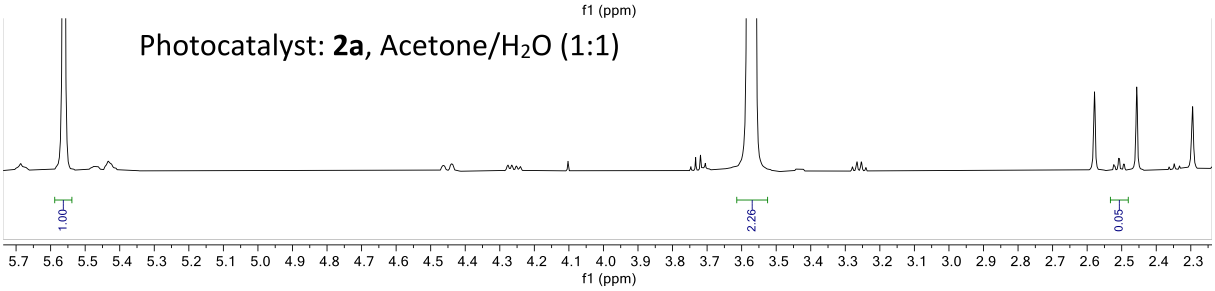

NMR Spectra – Variation of Reaction Parameters

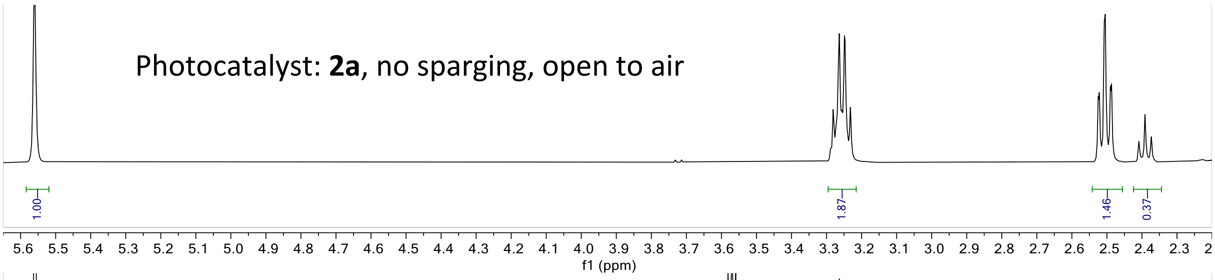

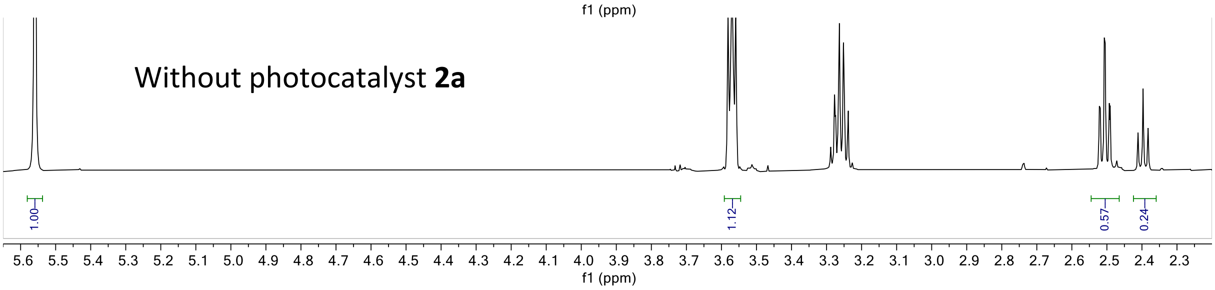

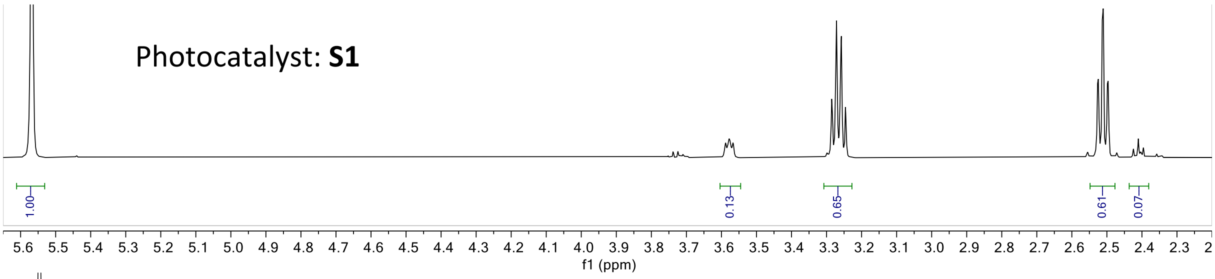

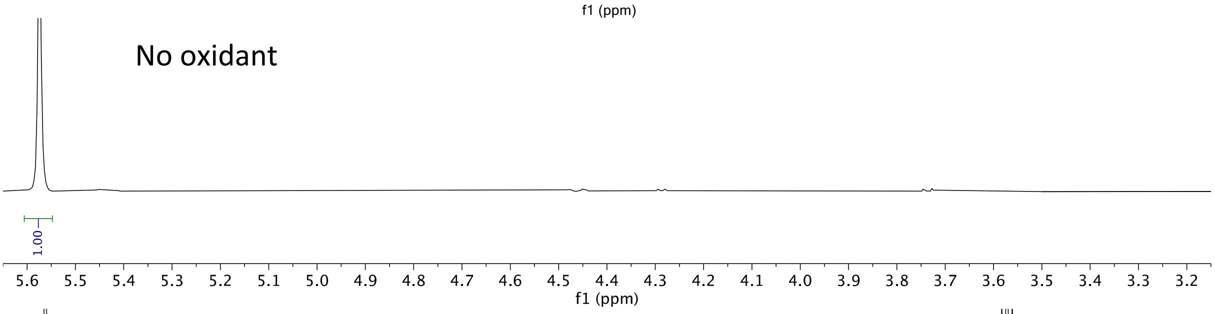

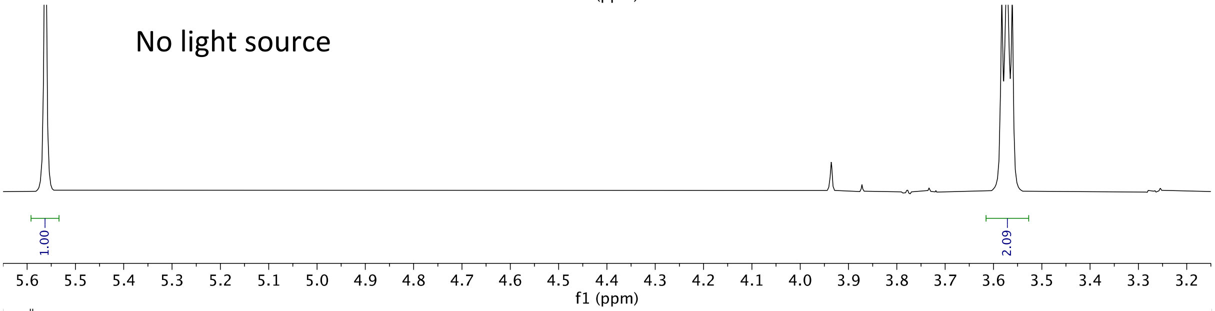

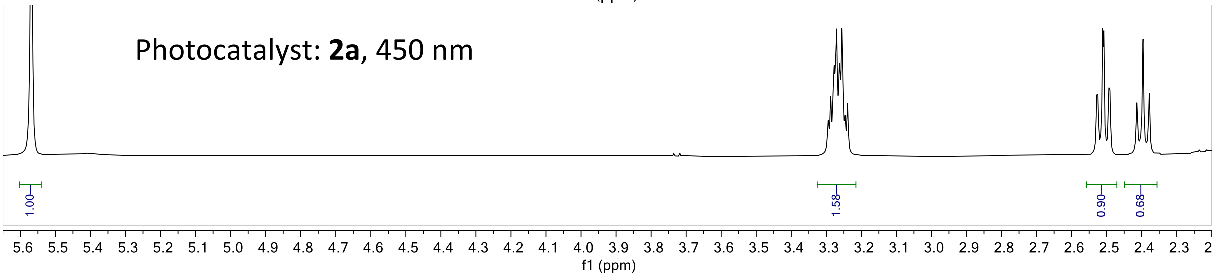

NMR Spectra – N-Protecting Groups

For spectrum of N-Piv piperidine, see pg. S71.

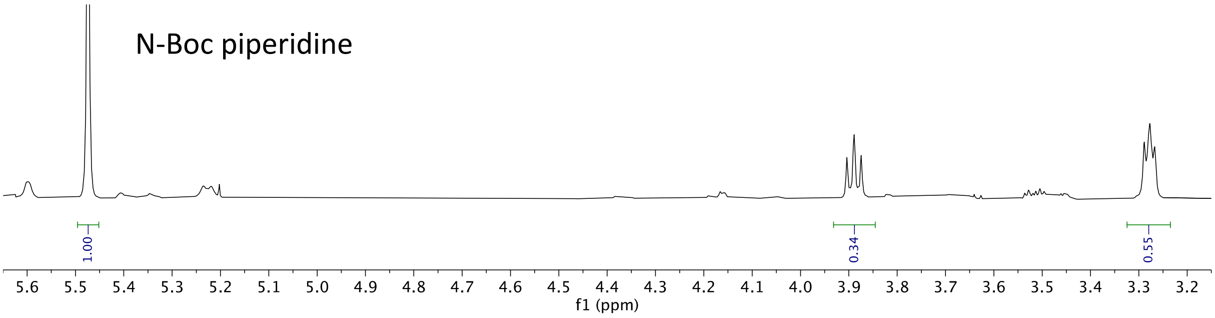

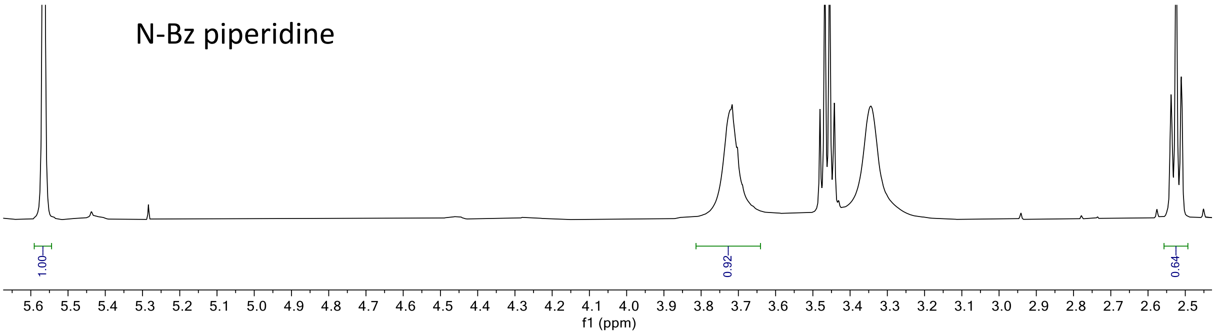

NMR Spectra – Aldehyde Products

N-(5-oxopentyl)pivalamide

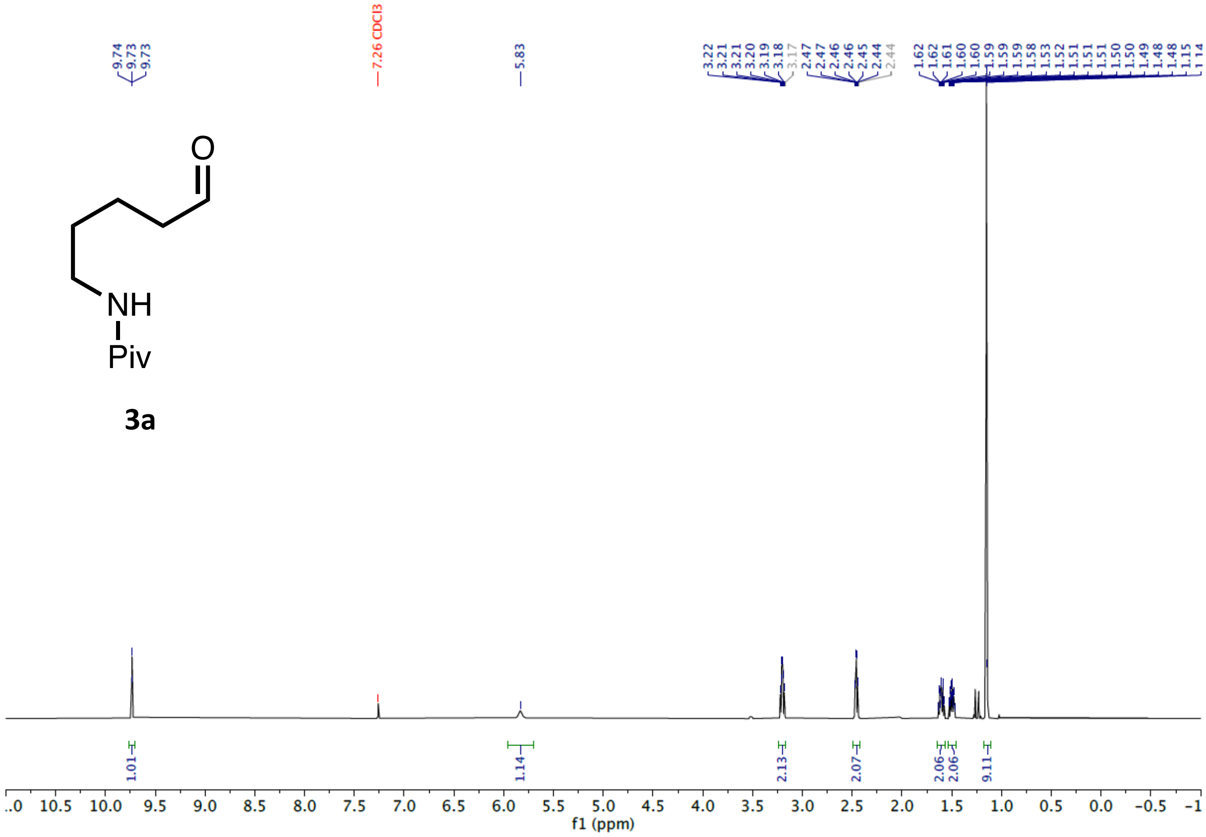

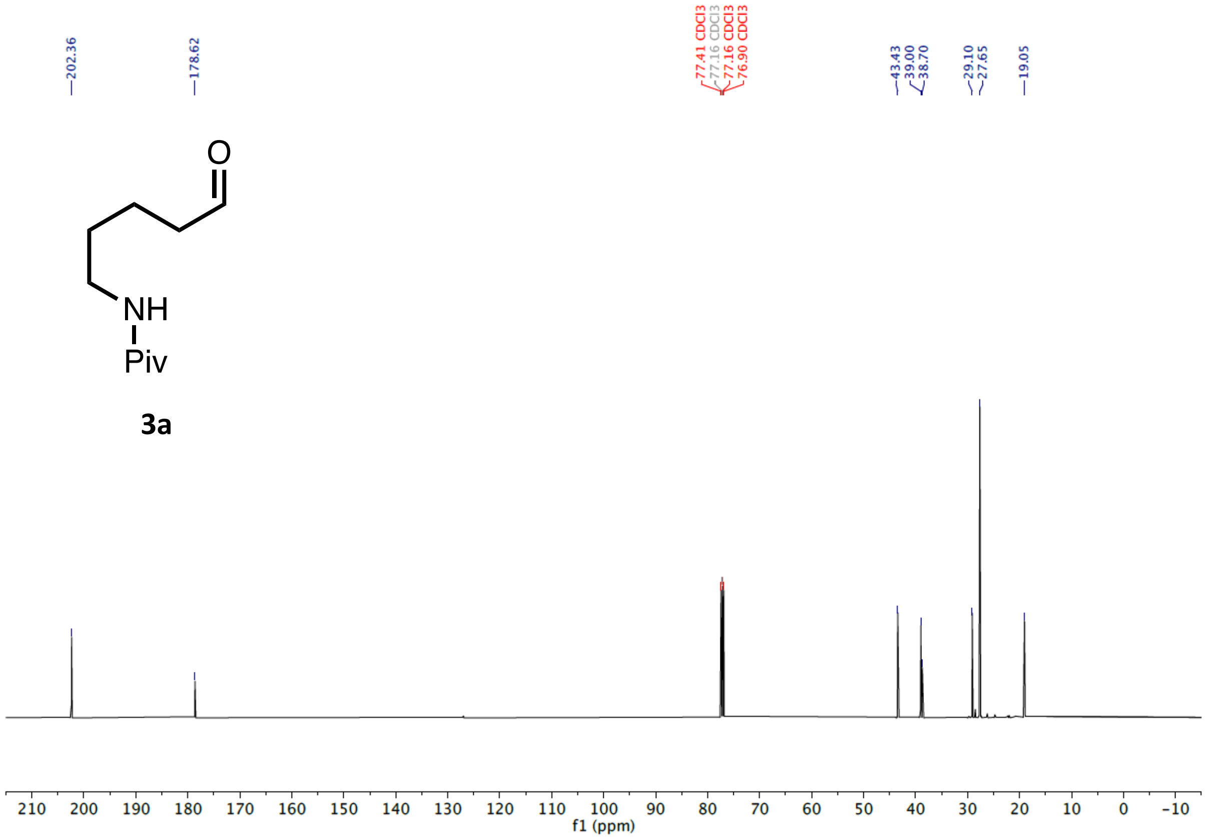

5-pivalamidopentanoic acid

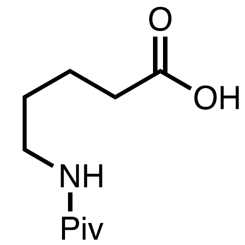

4a

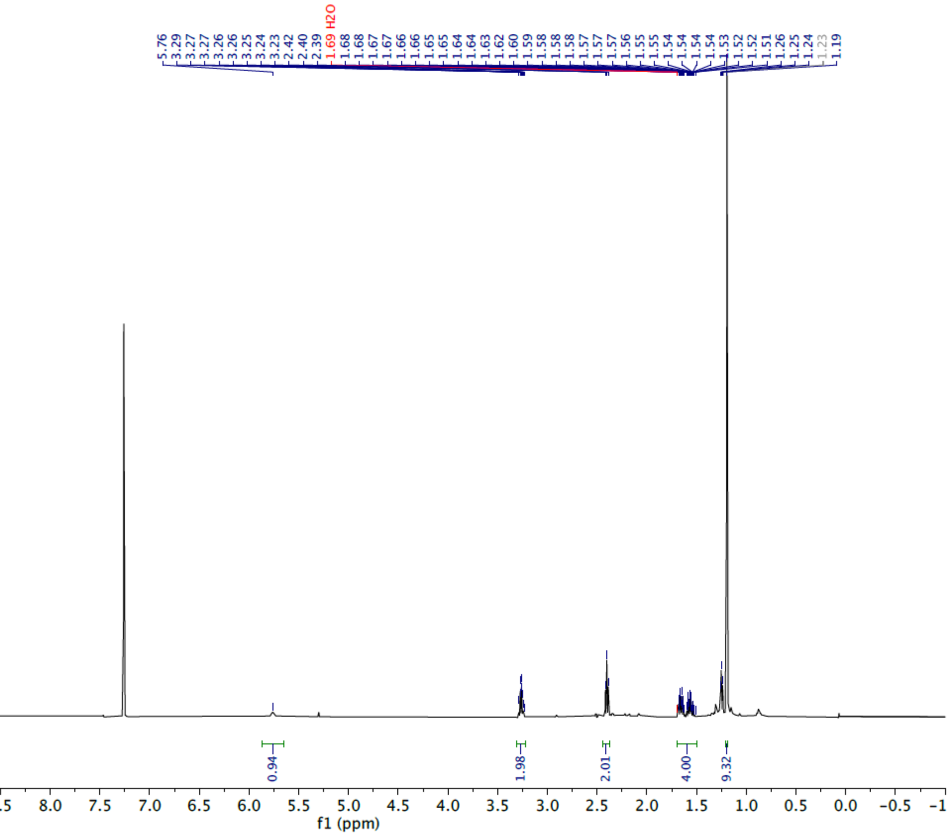

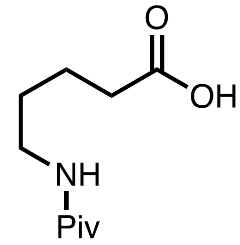

4a

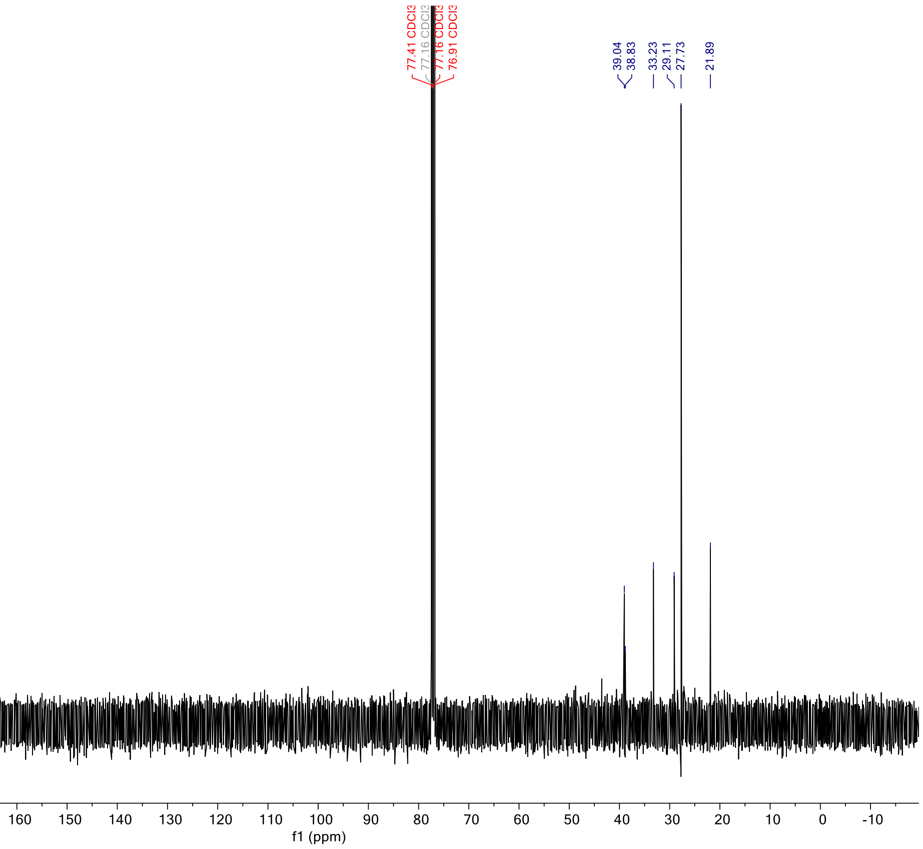

***N*-(4-oxobutyl)pivalamide**

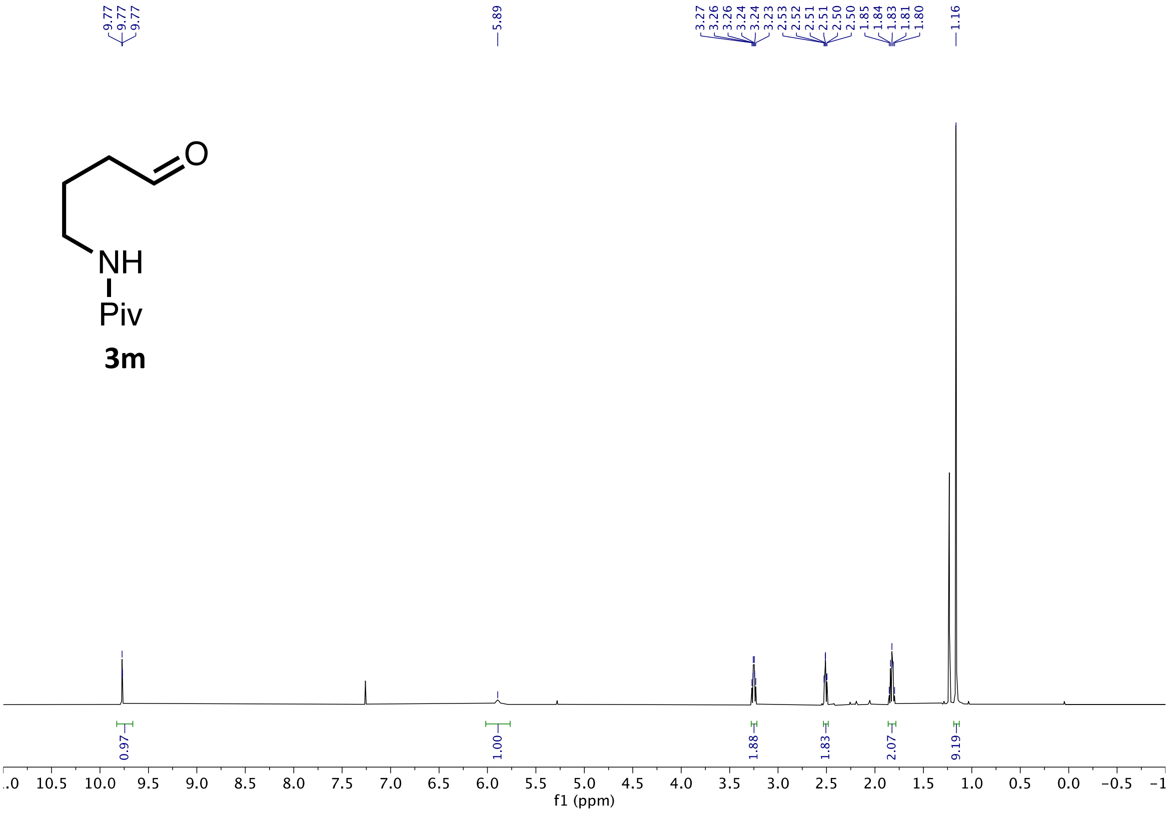

3m

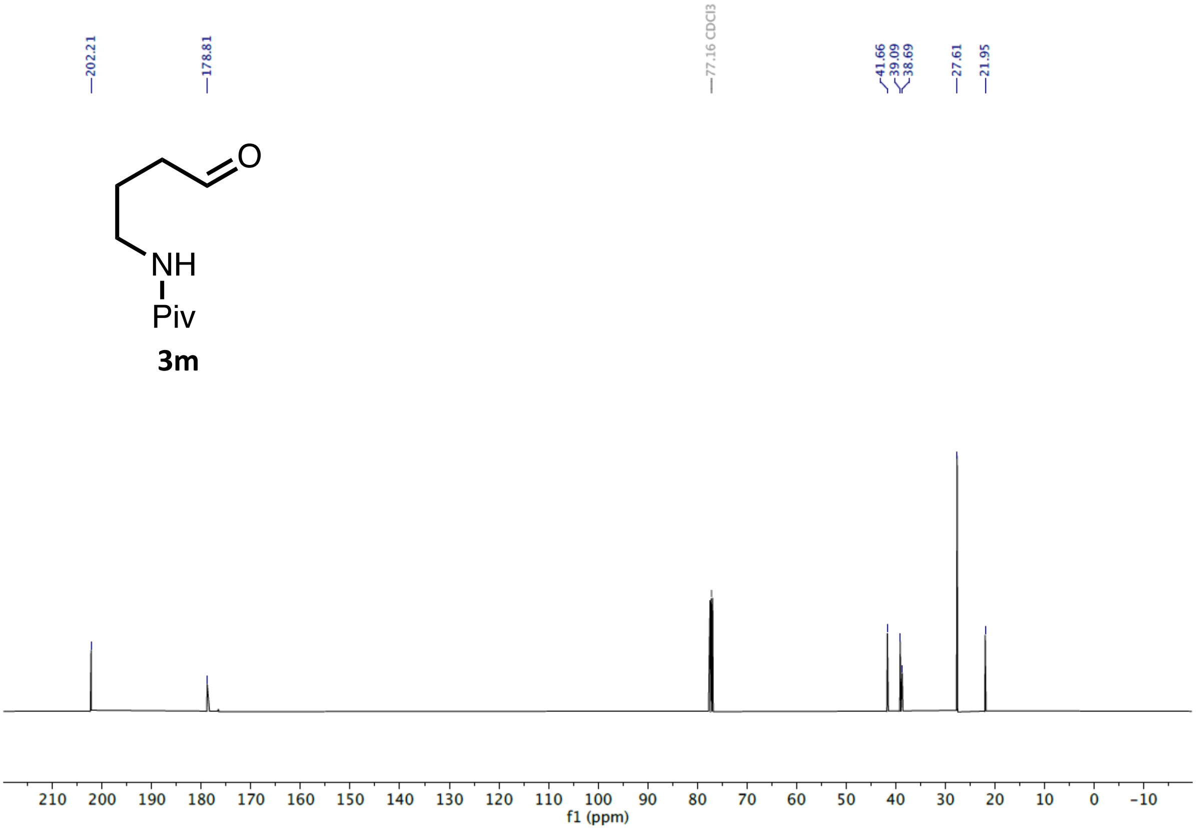

3m

4-pivalamidobutanoic acid

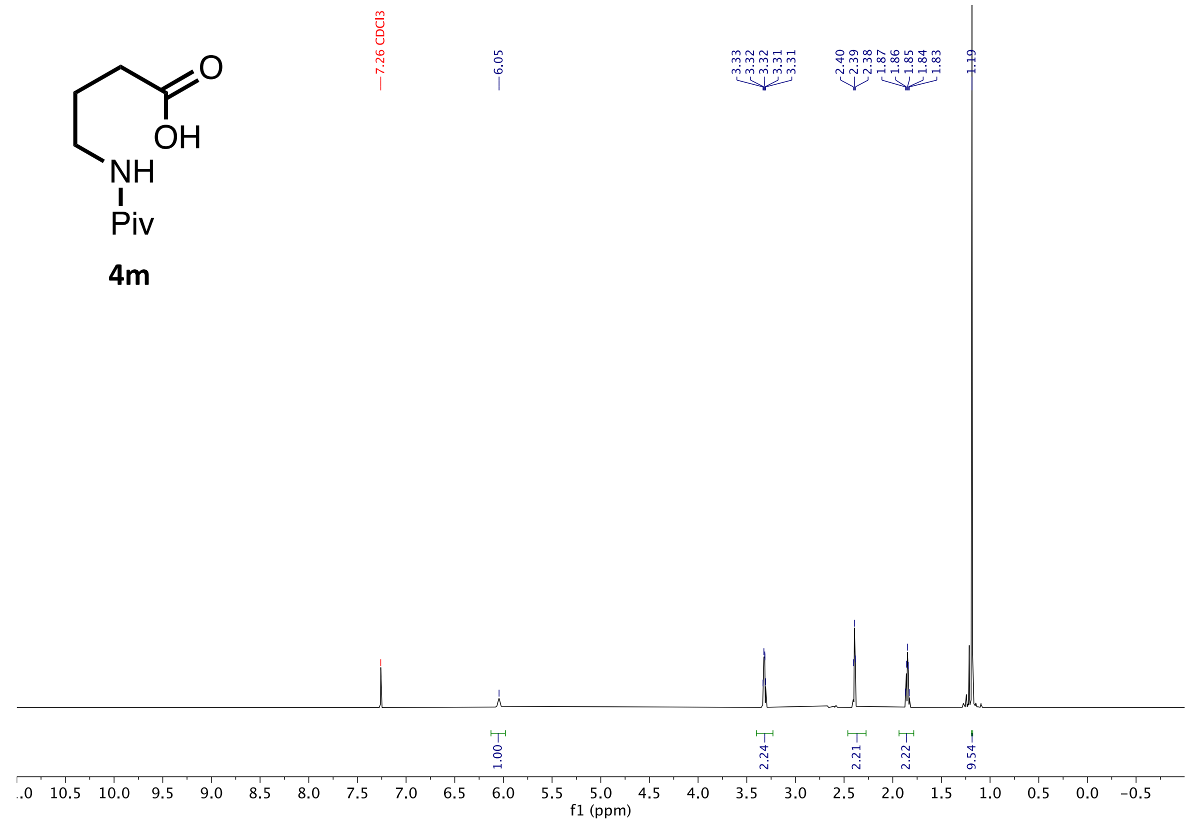

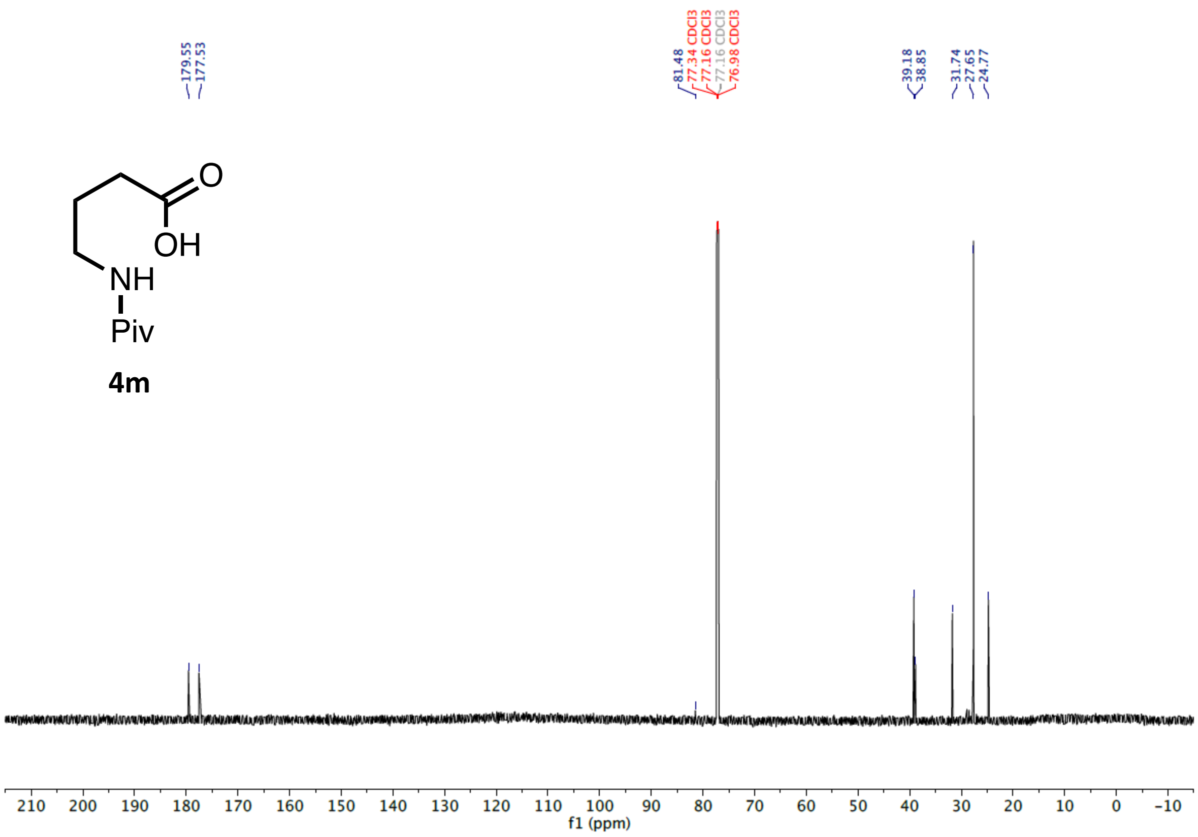

***N*-(6-oxohexyl)pivalamide**

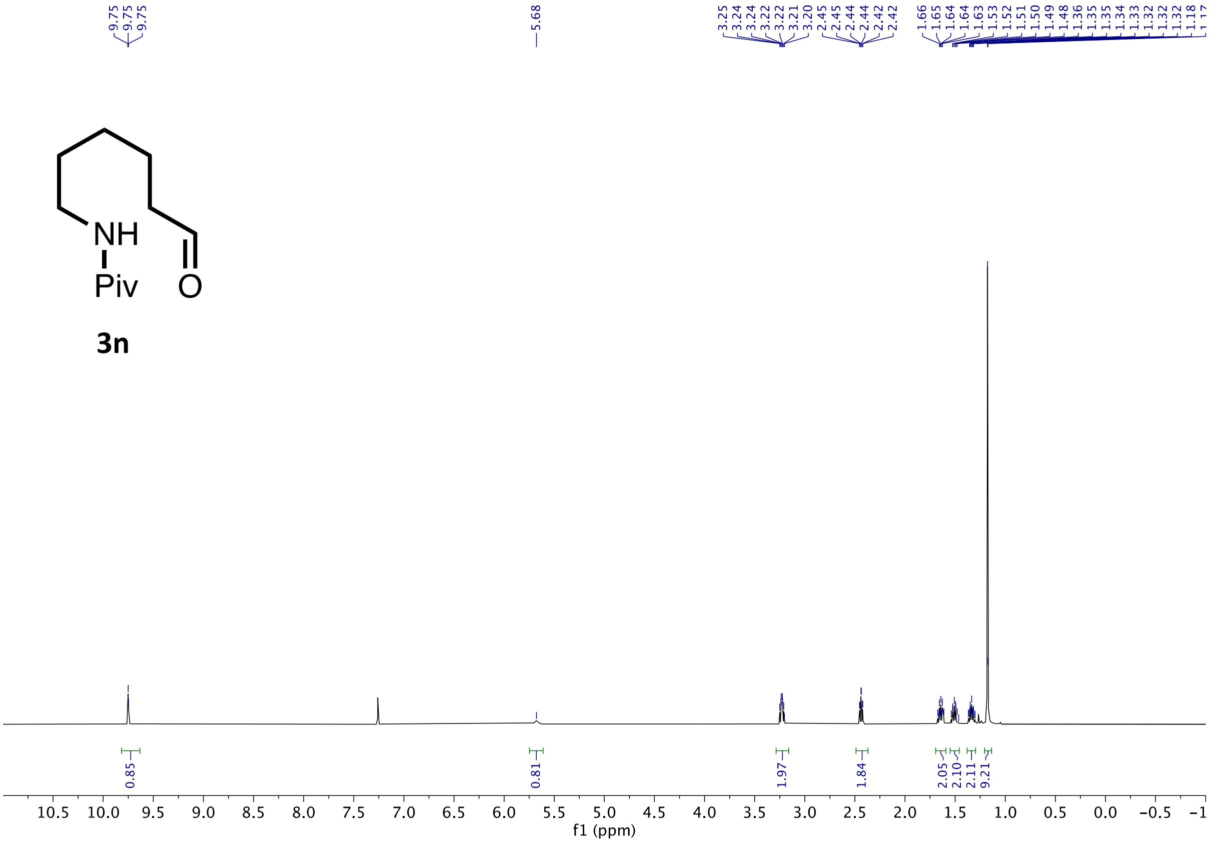

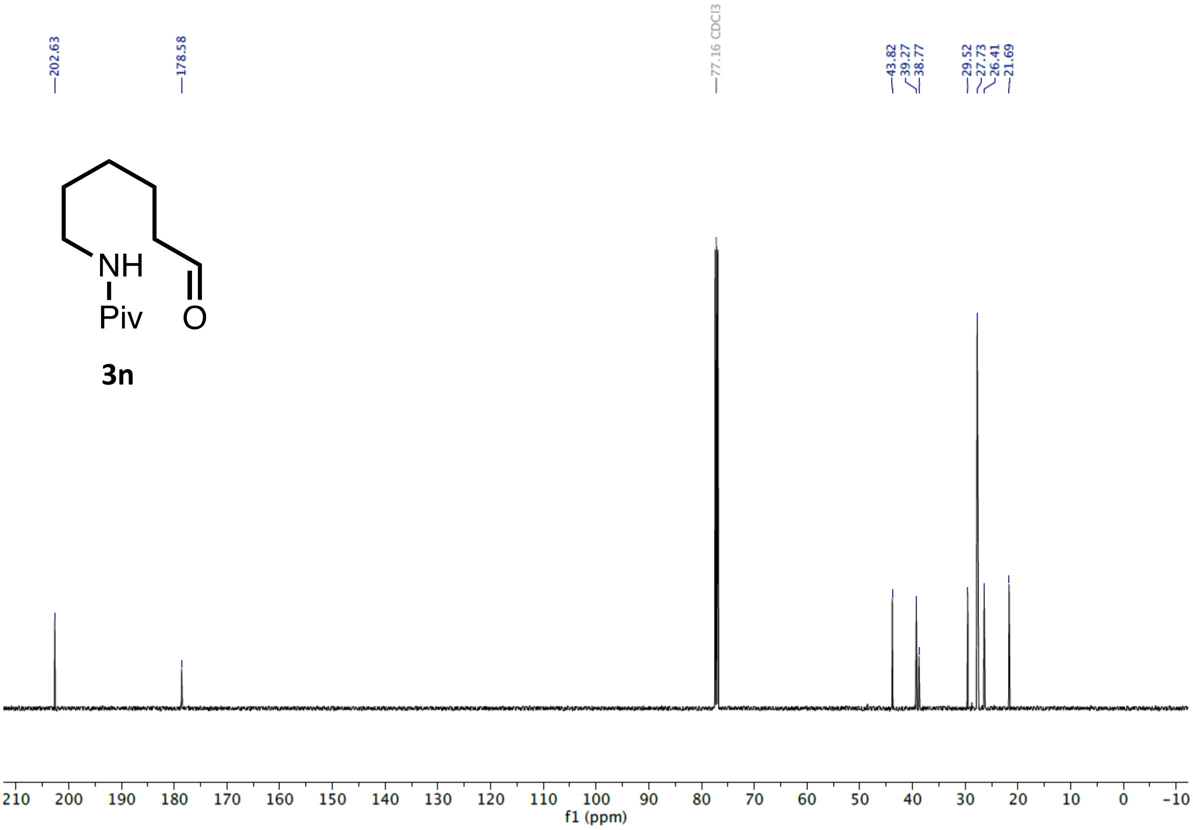

6-pivalamidohexanoic acid

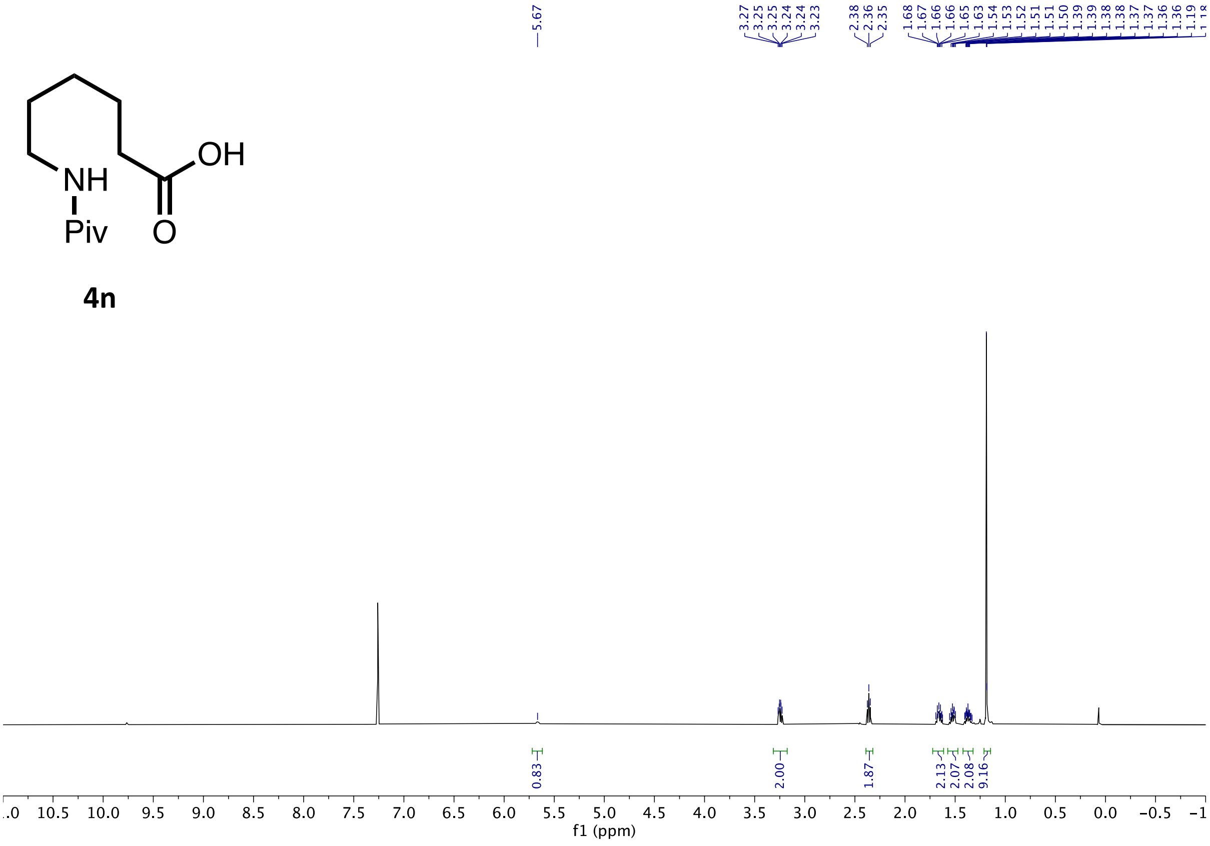

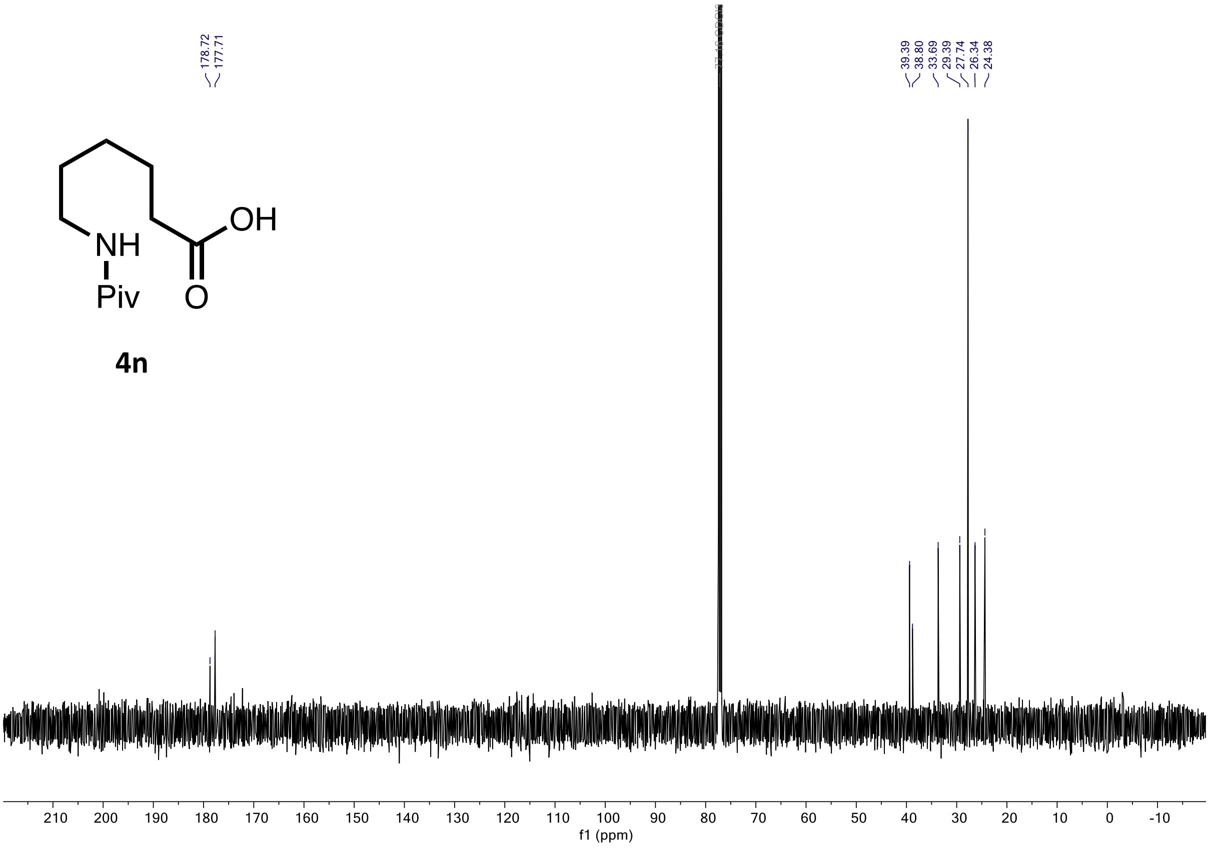

N-(7-oxoheptyl)pivalamide

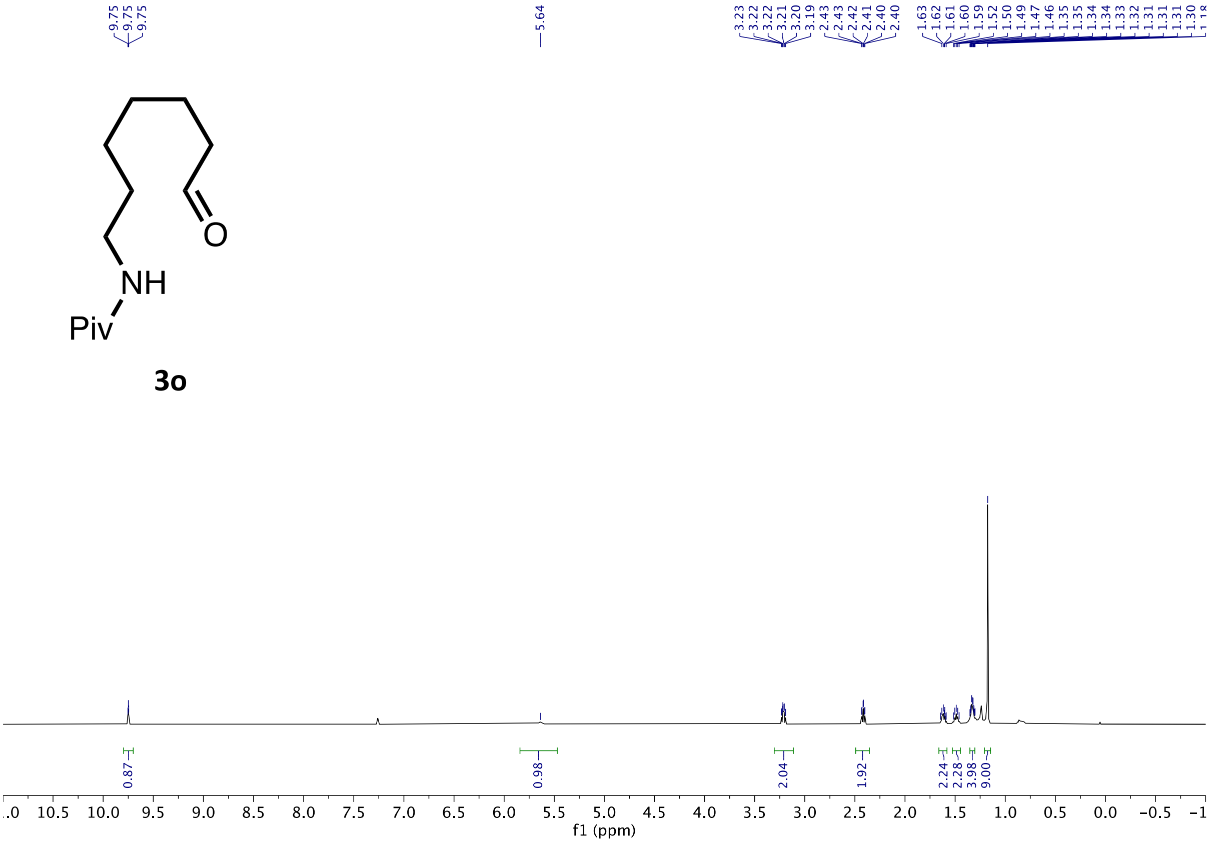

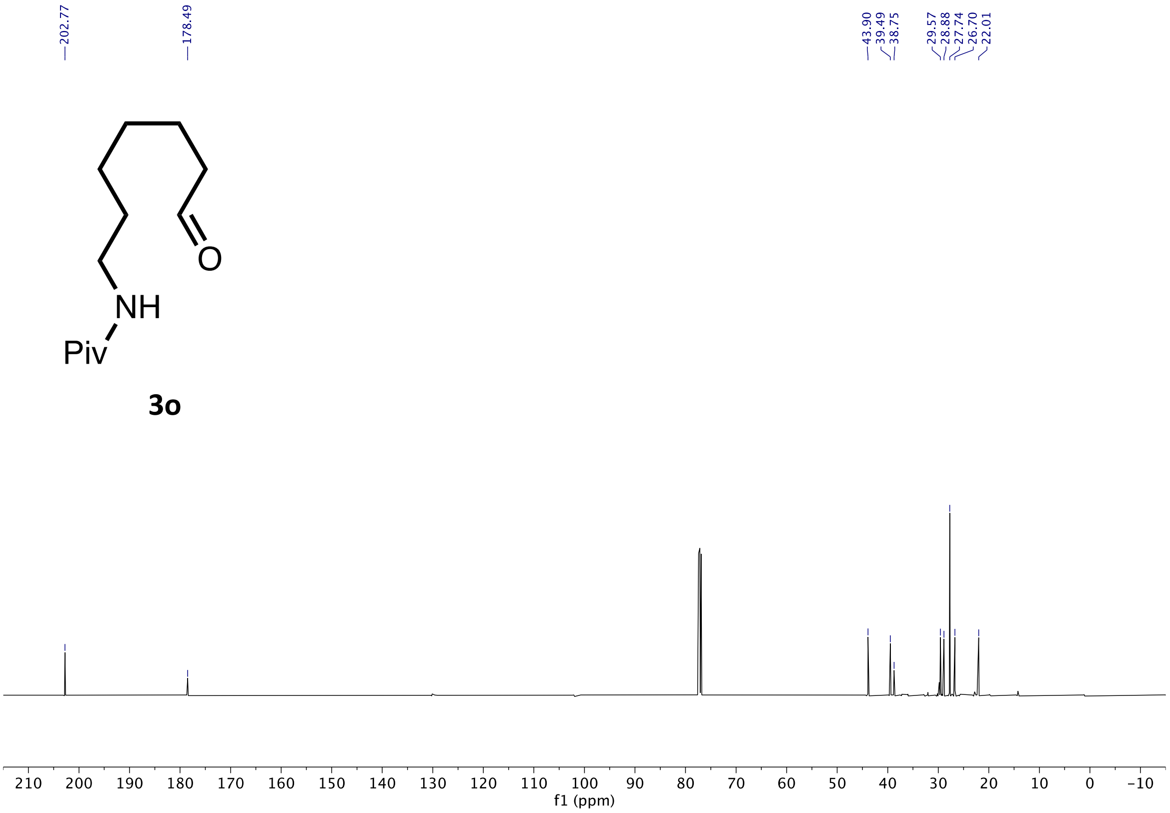

N-((1*S*,2*R*)-2-(3-oxopropyl)cyclohexyl)pivalamide

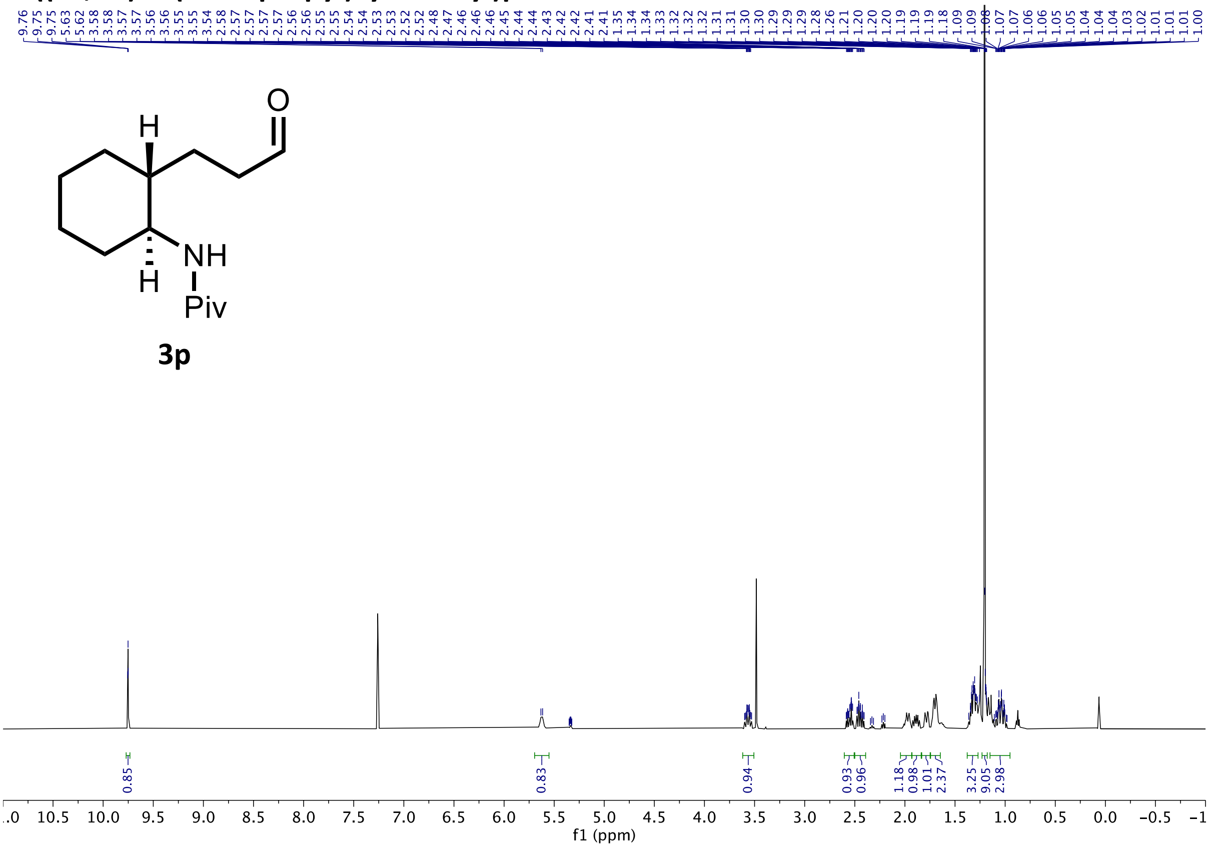

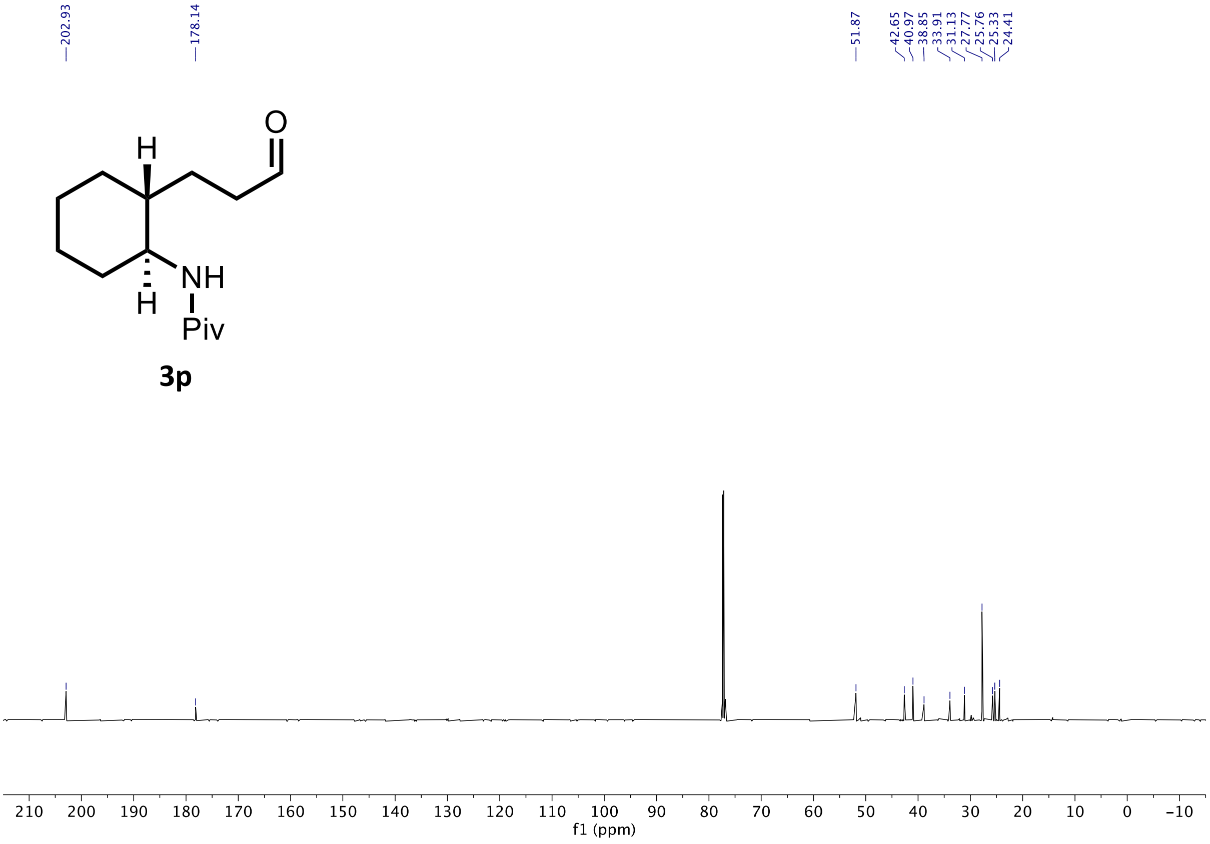

N-(3-(4-chlorophenyl)-5-oxopentyl)pivalamide

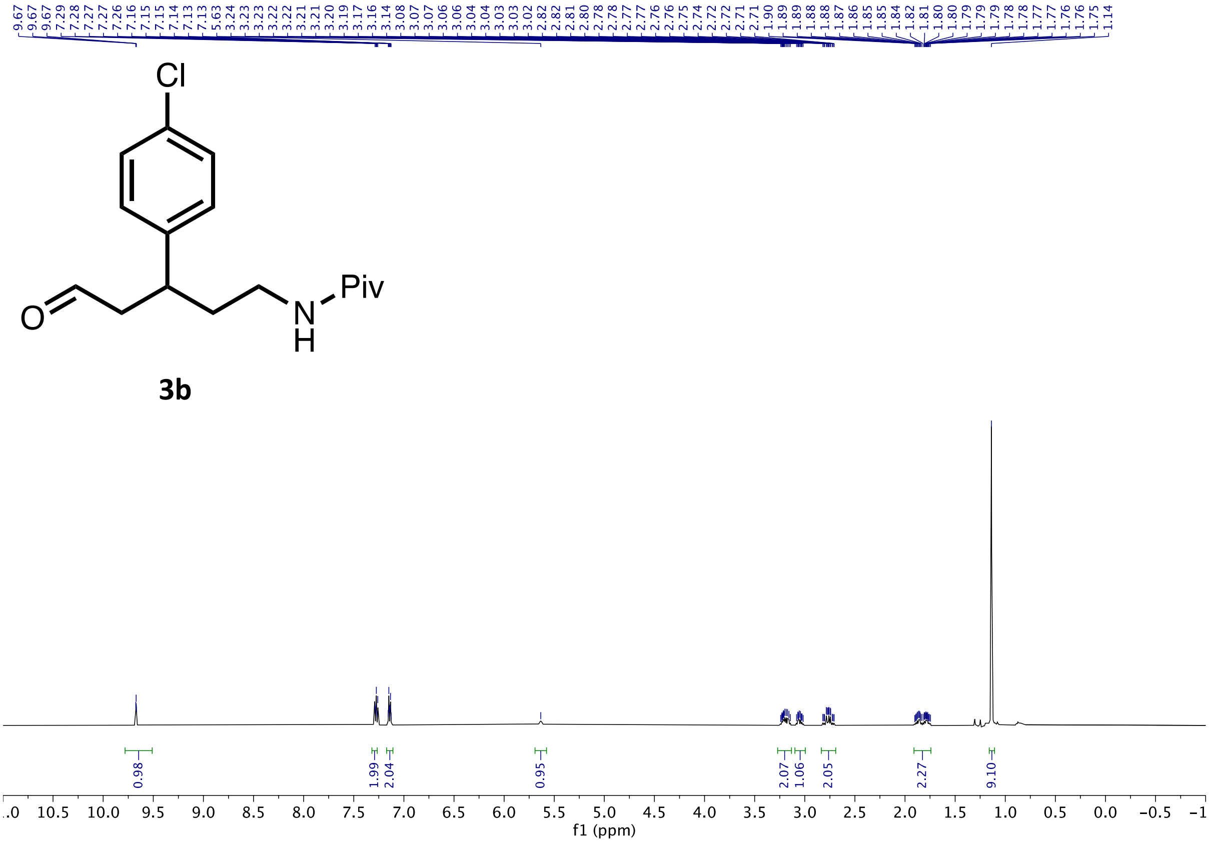

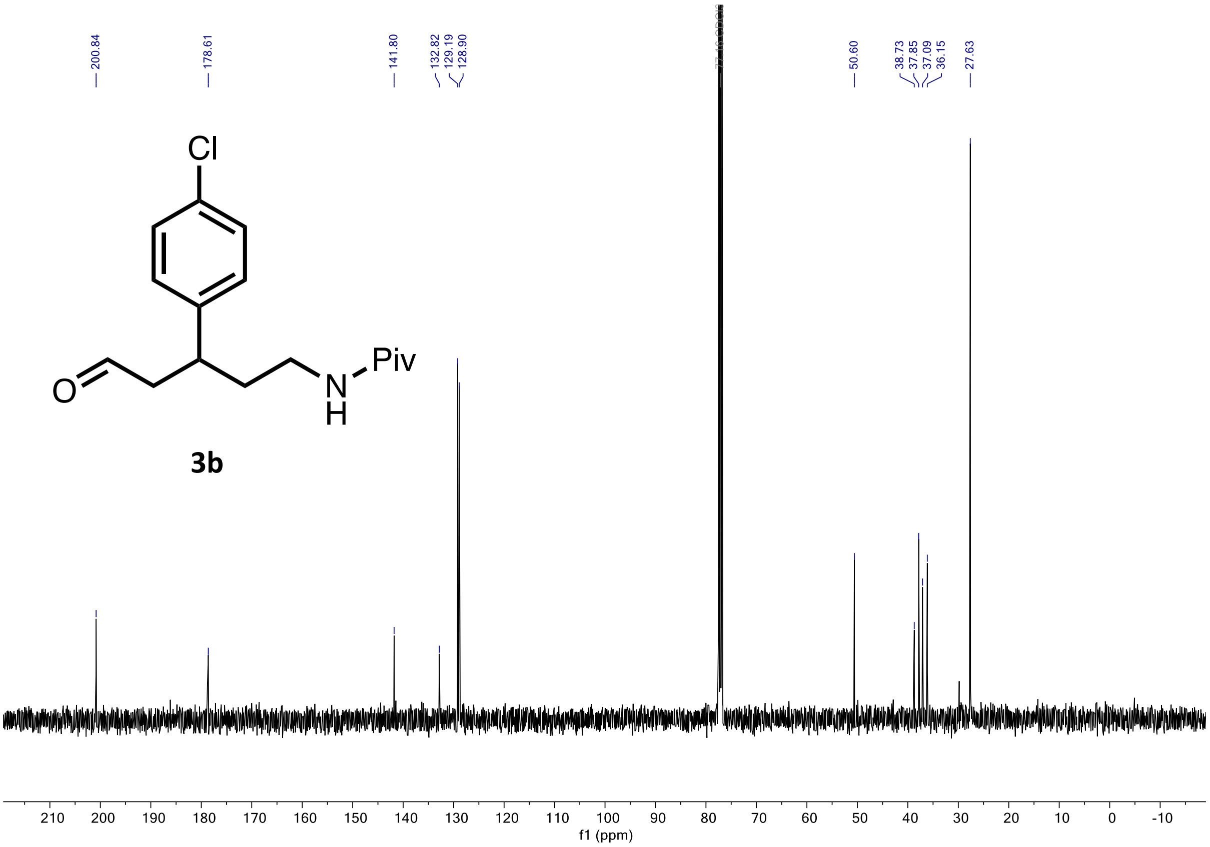

***N*-(5-oxo-3-phenylpentyl)pivalamide**

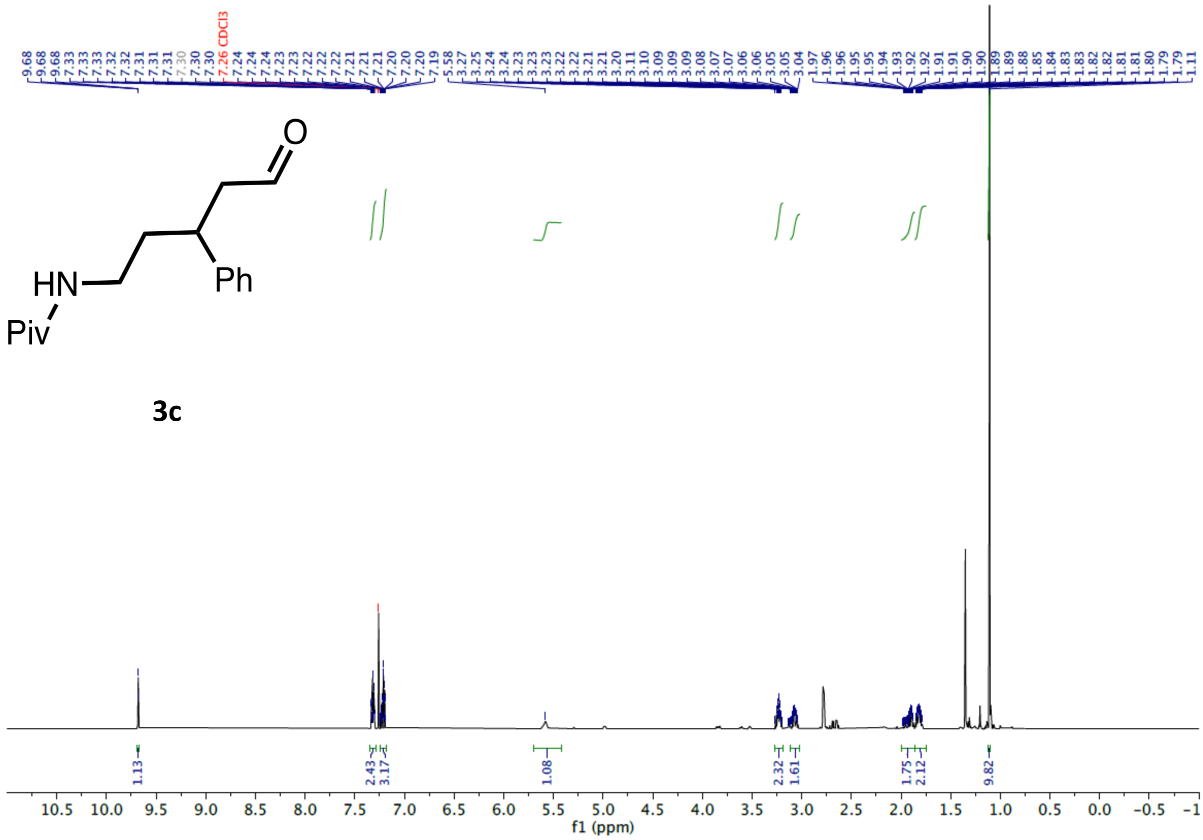

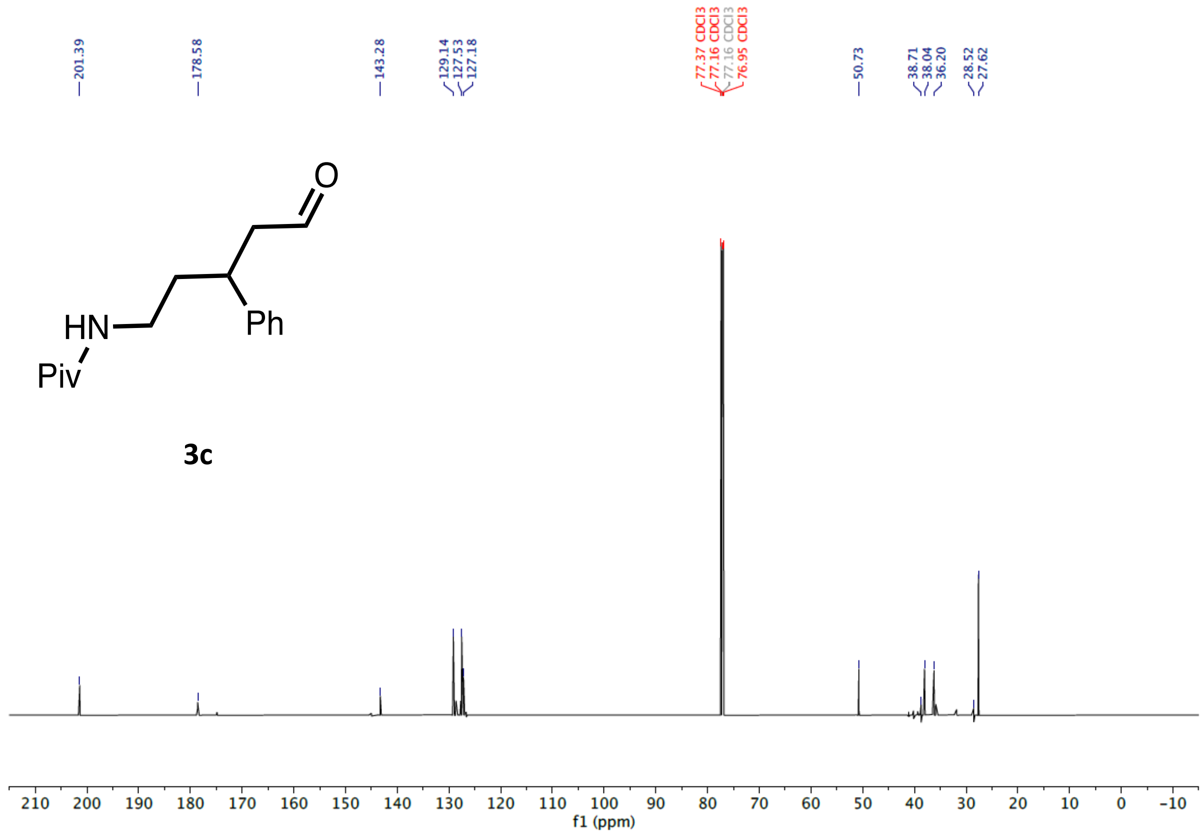

***N*-(3-methyl-5-oxopentyl)pivalamide**

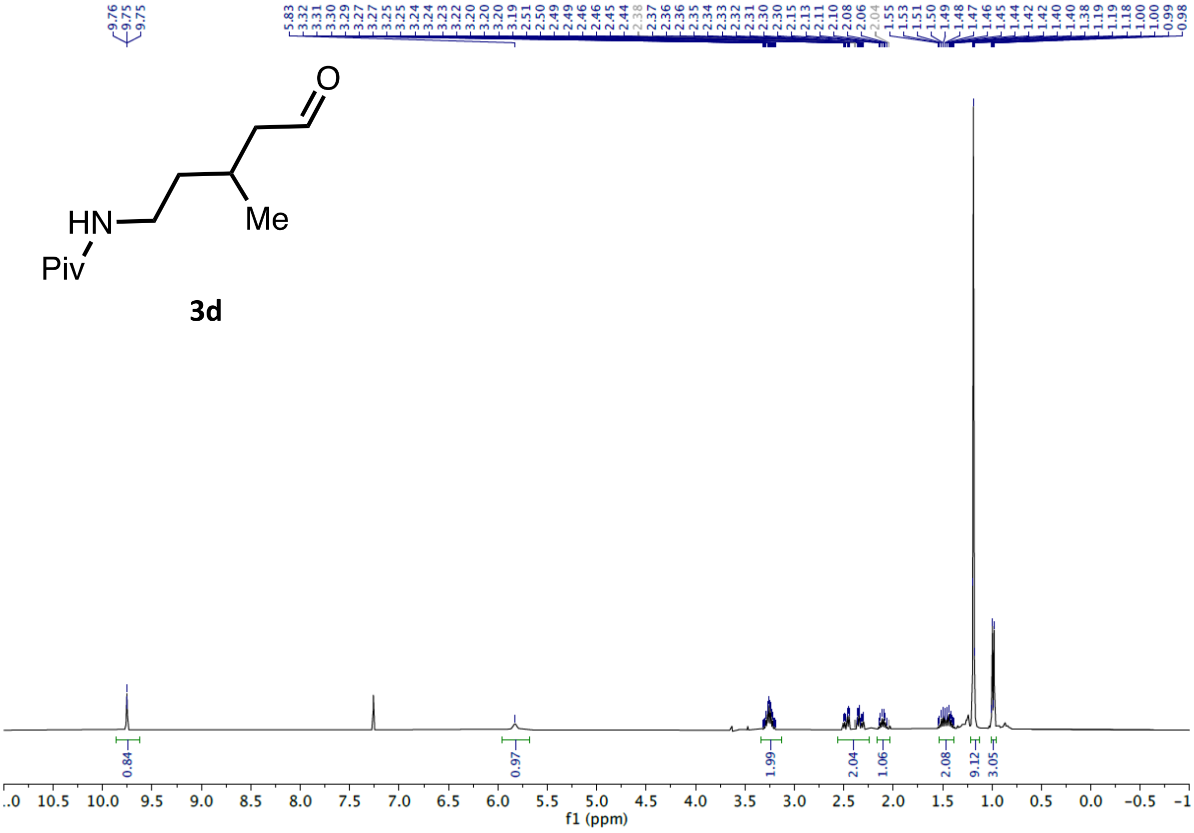

3d

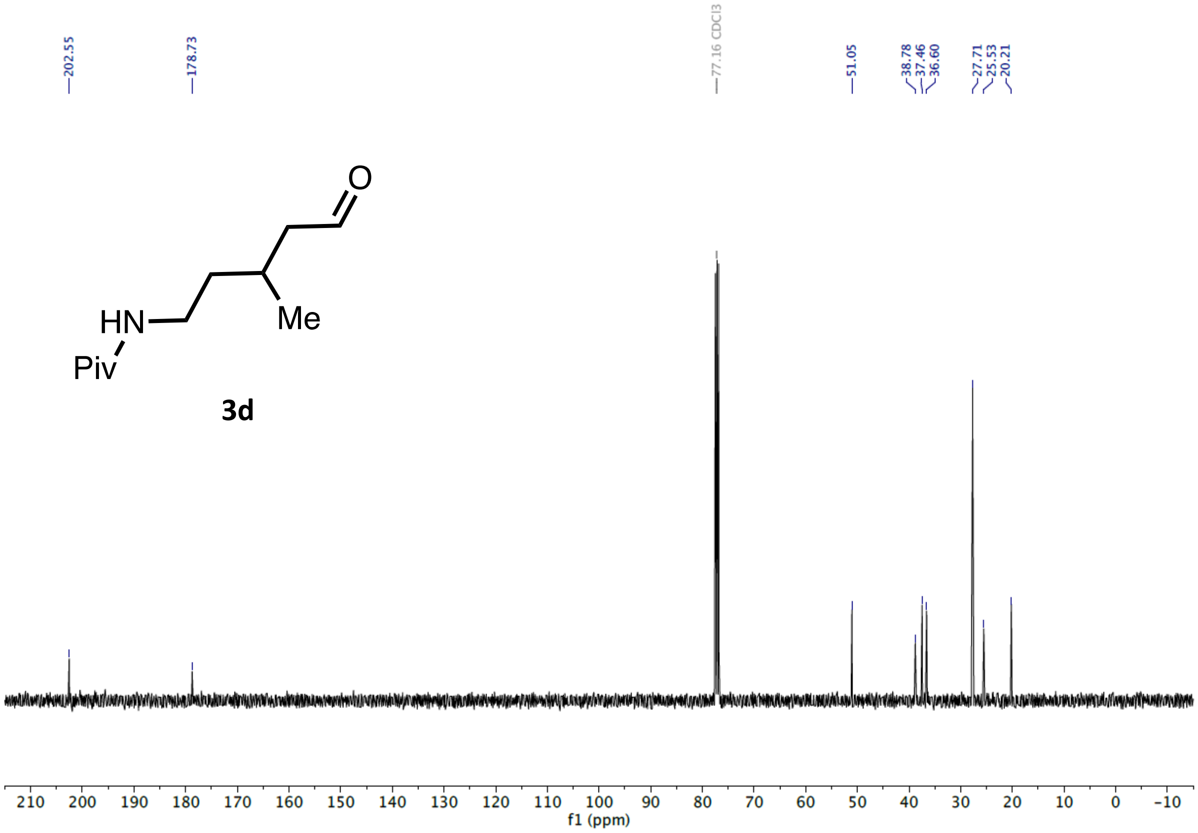

3d

Methyl 5-oxo-3-(2-pivalamidoethyl)pentanoate

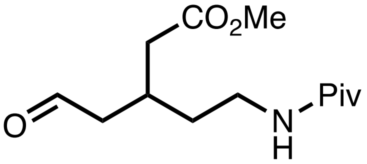

3f

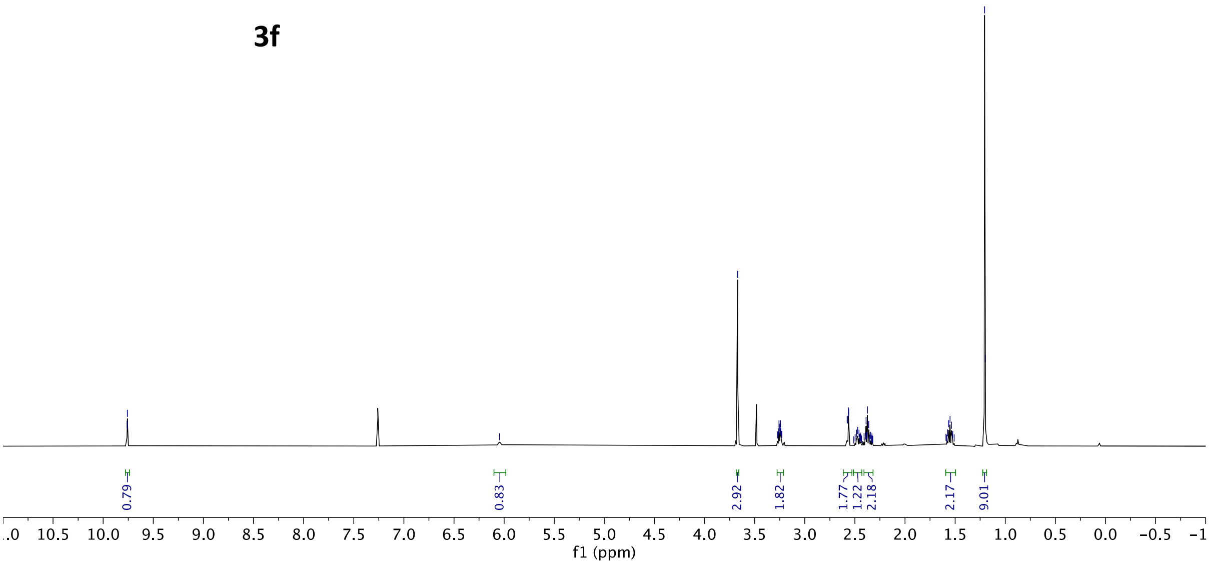

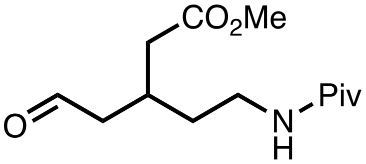

3f

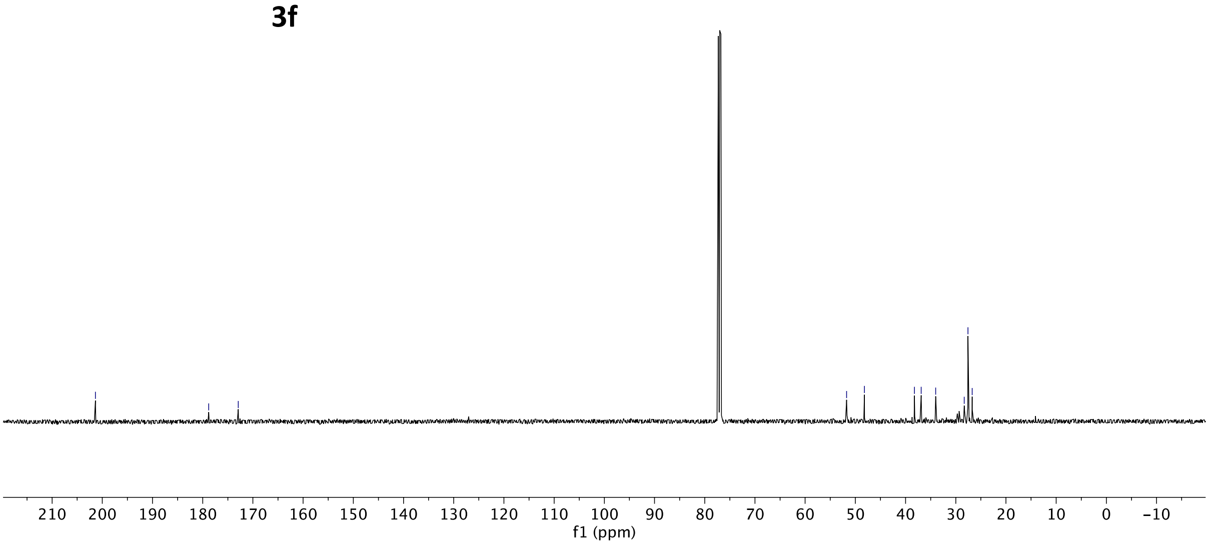

Ethyl 4-oxo-2-(2-pivalamidoethyl)butanoate

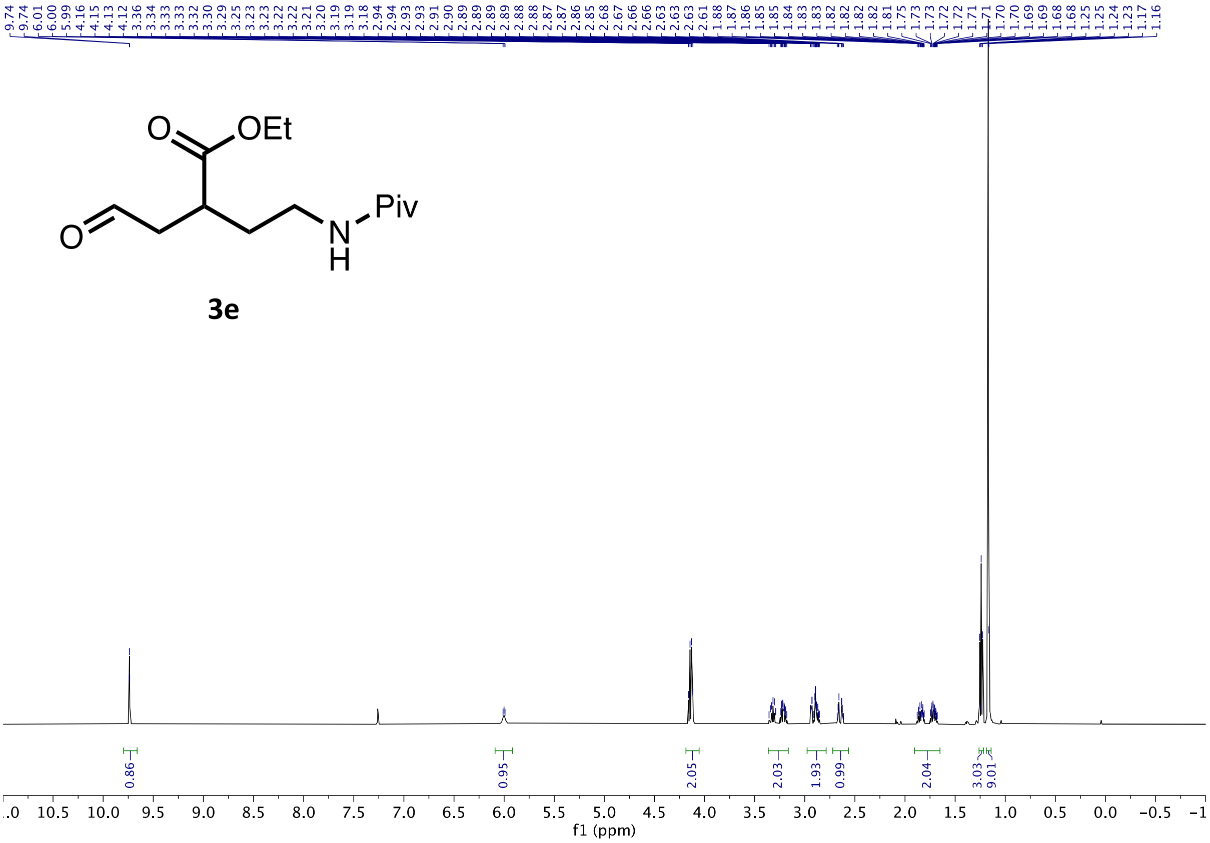

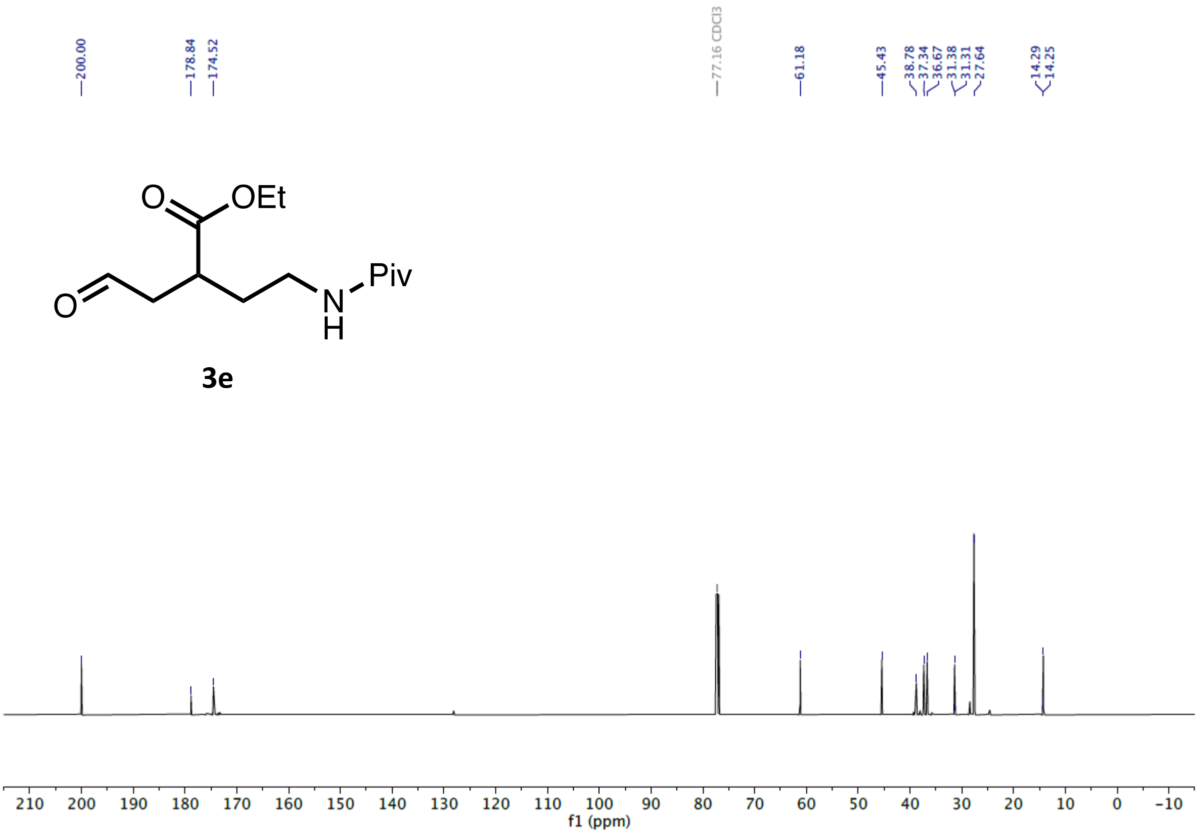

N-(7-oxoheptan-3-yl)pivalamide

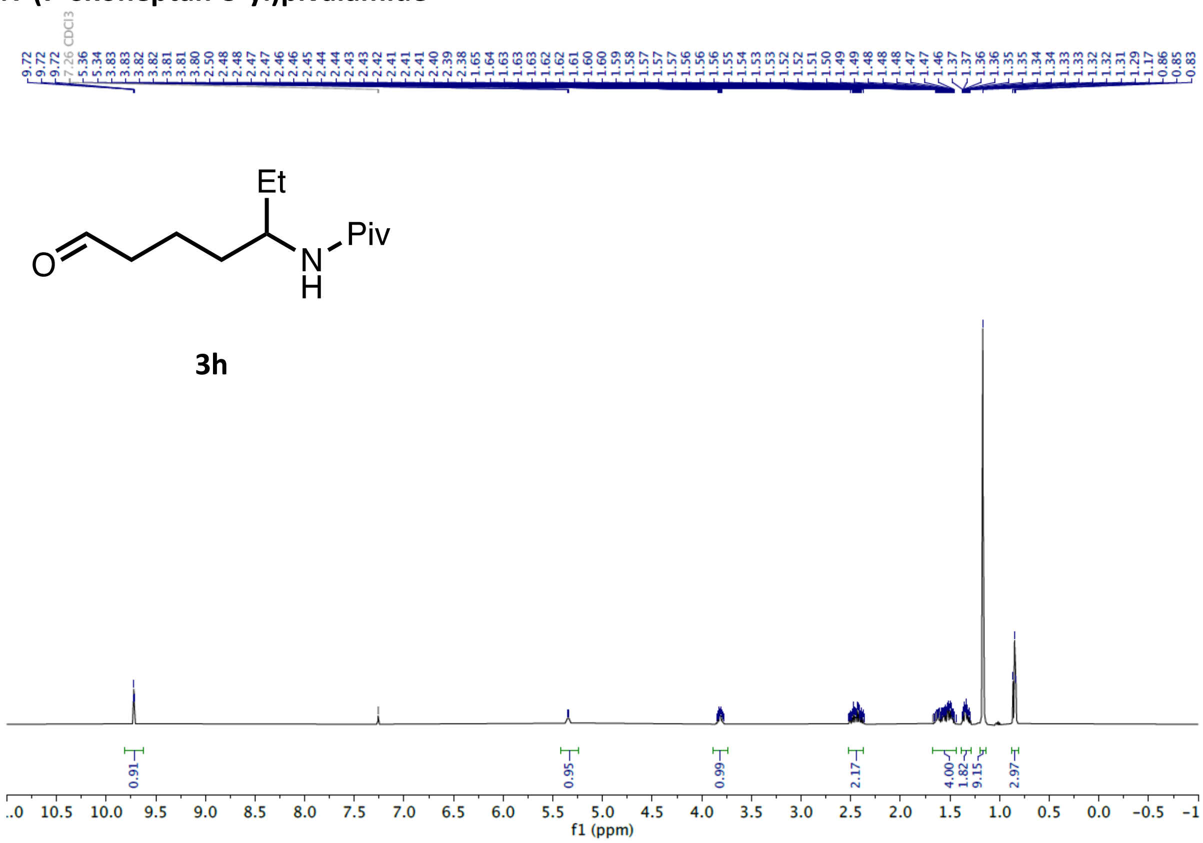

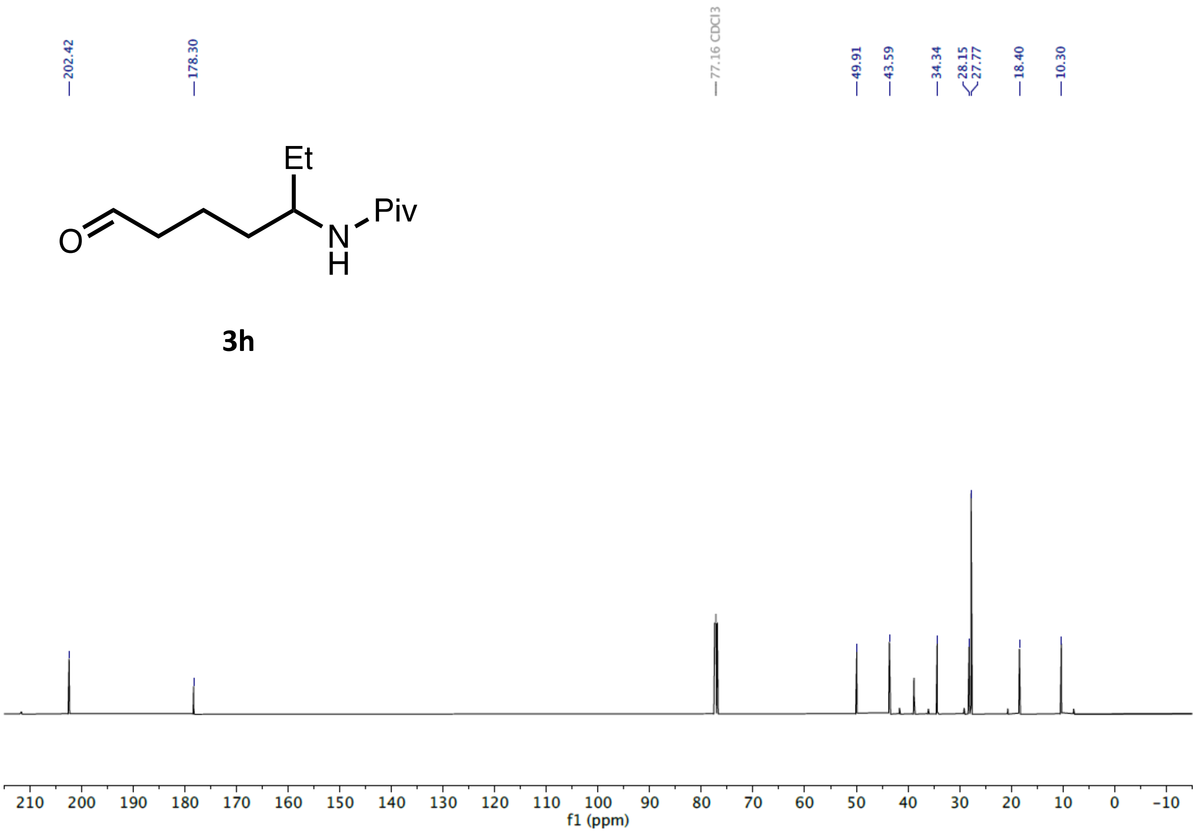

***N*-(6-oxohexan-2-yl)pivalamide**

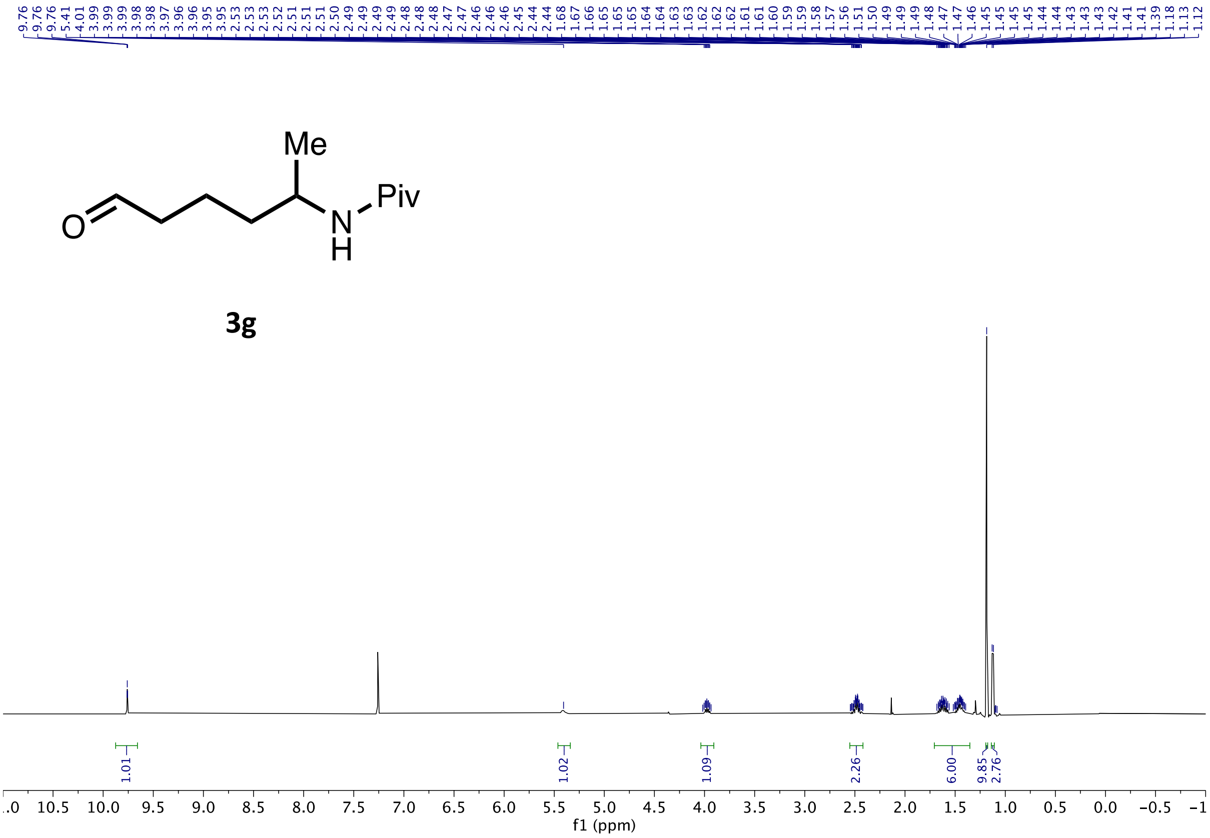

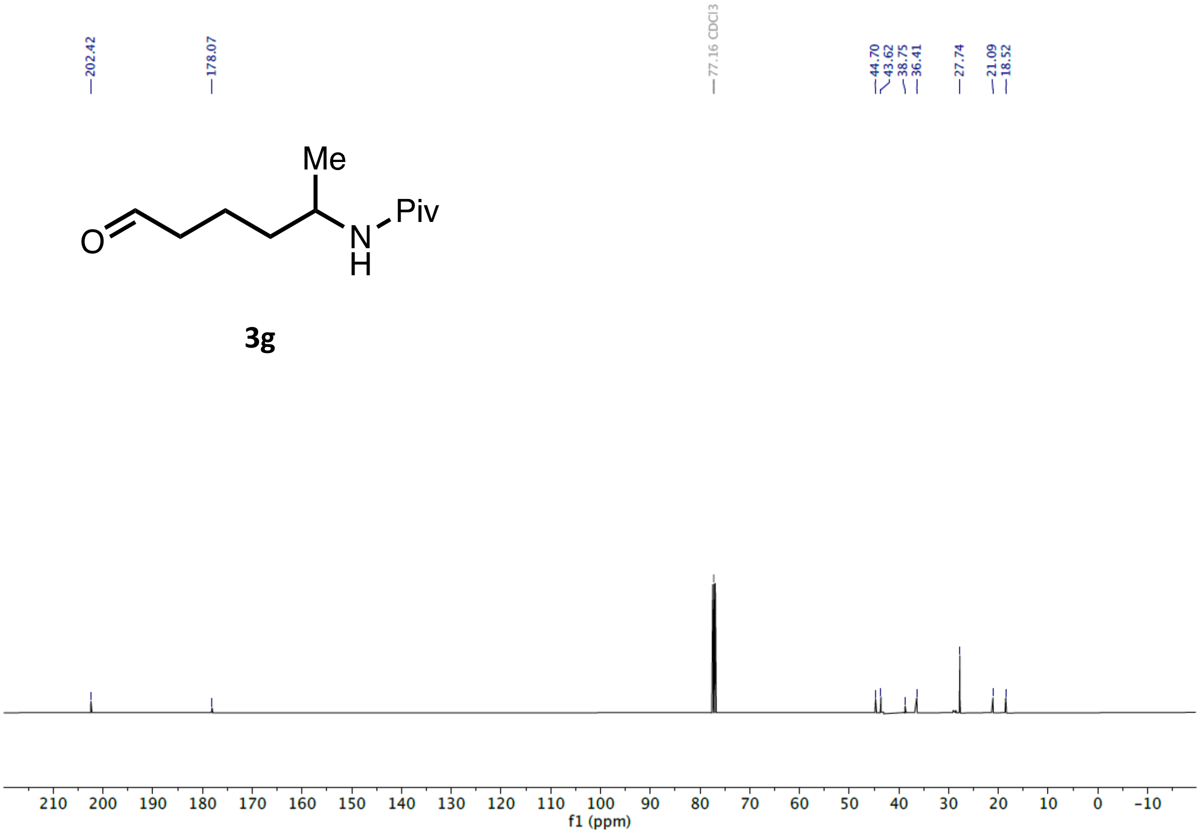

N-(2-methyl-5-oxopentyl)pivalamide (**3i.a**) and **N-(4-methyl-5-oxopentyl)pivalamide** (**3i.b**)

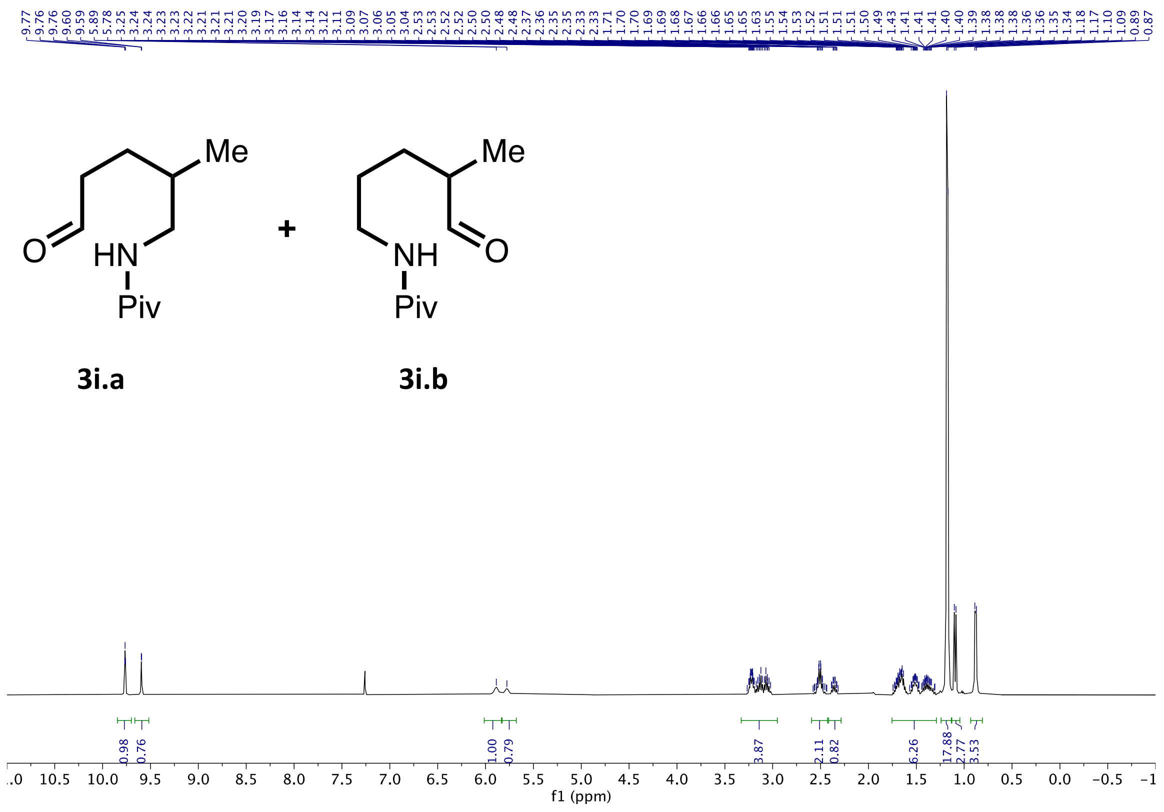

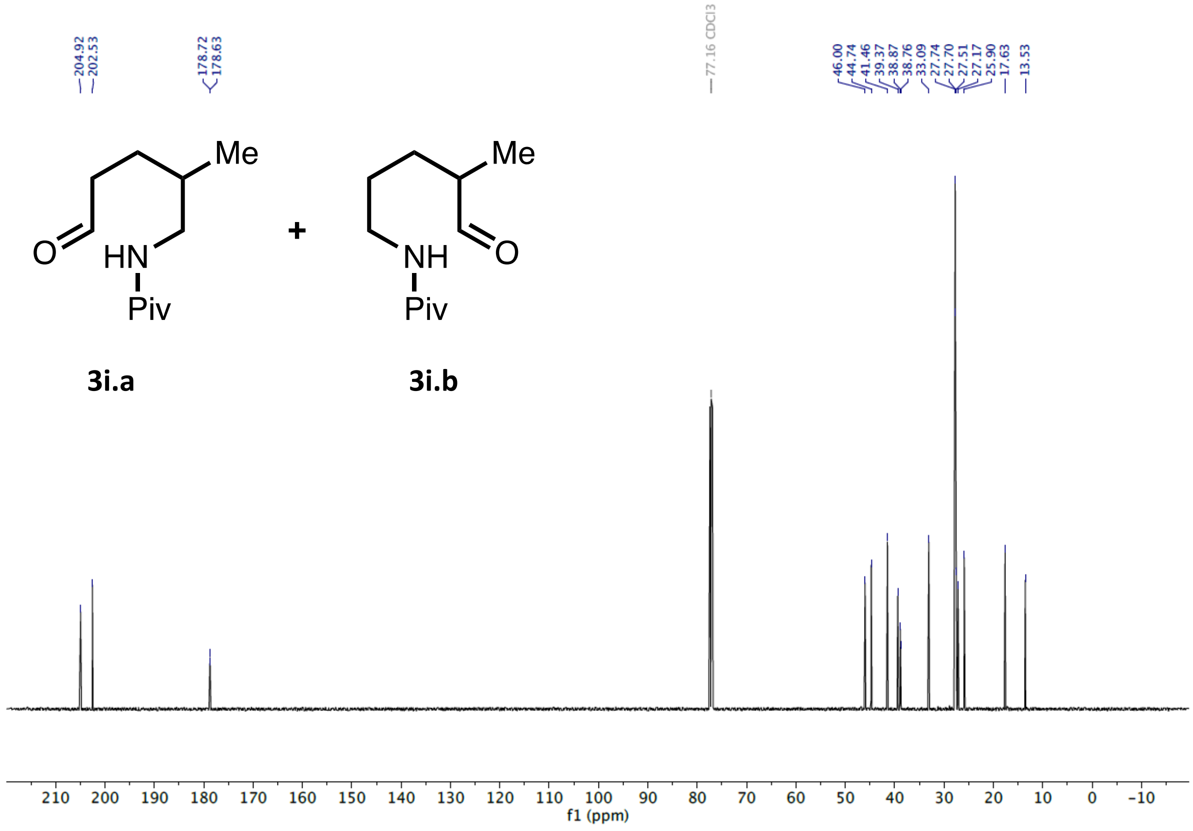

N-(5-oxo-2-phenylpentyl)pivalamide and **N-(5-oxo-4-phenylpentyl)pivalamide**

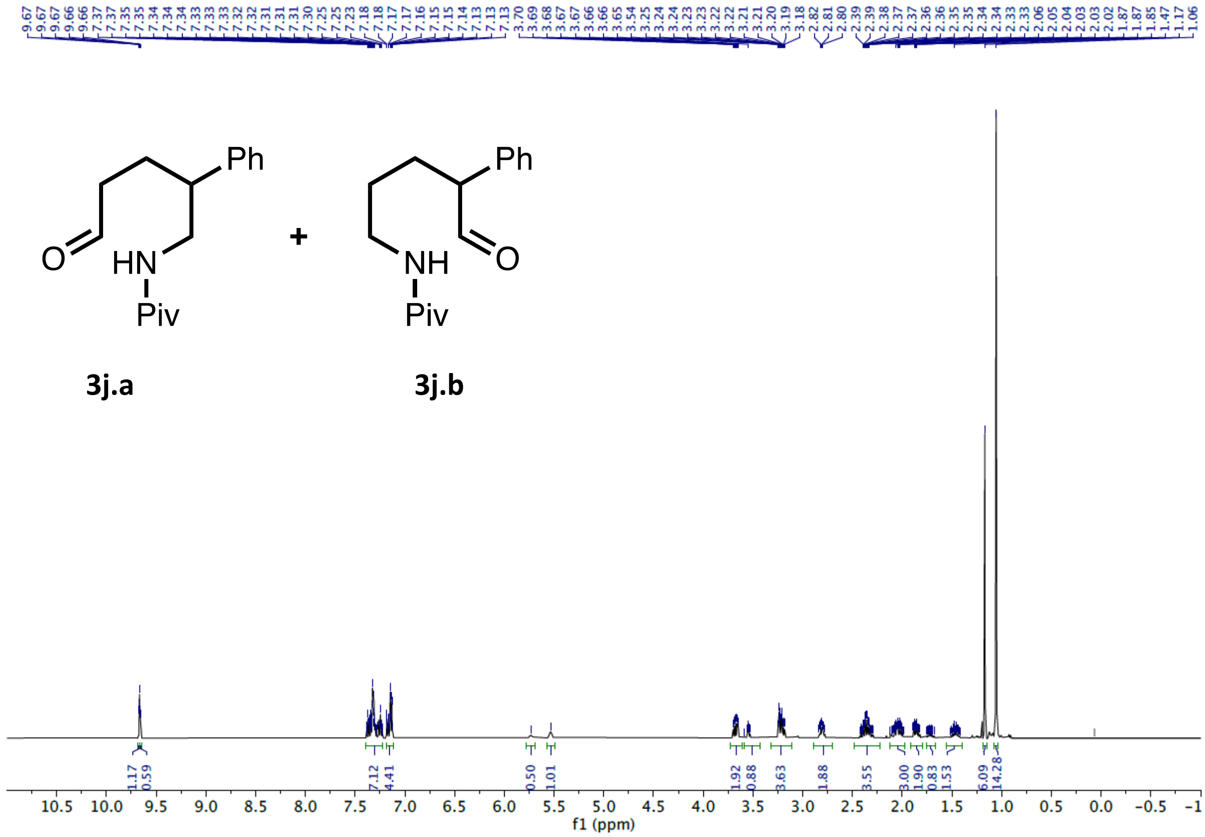

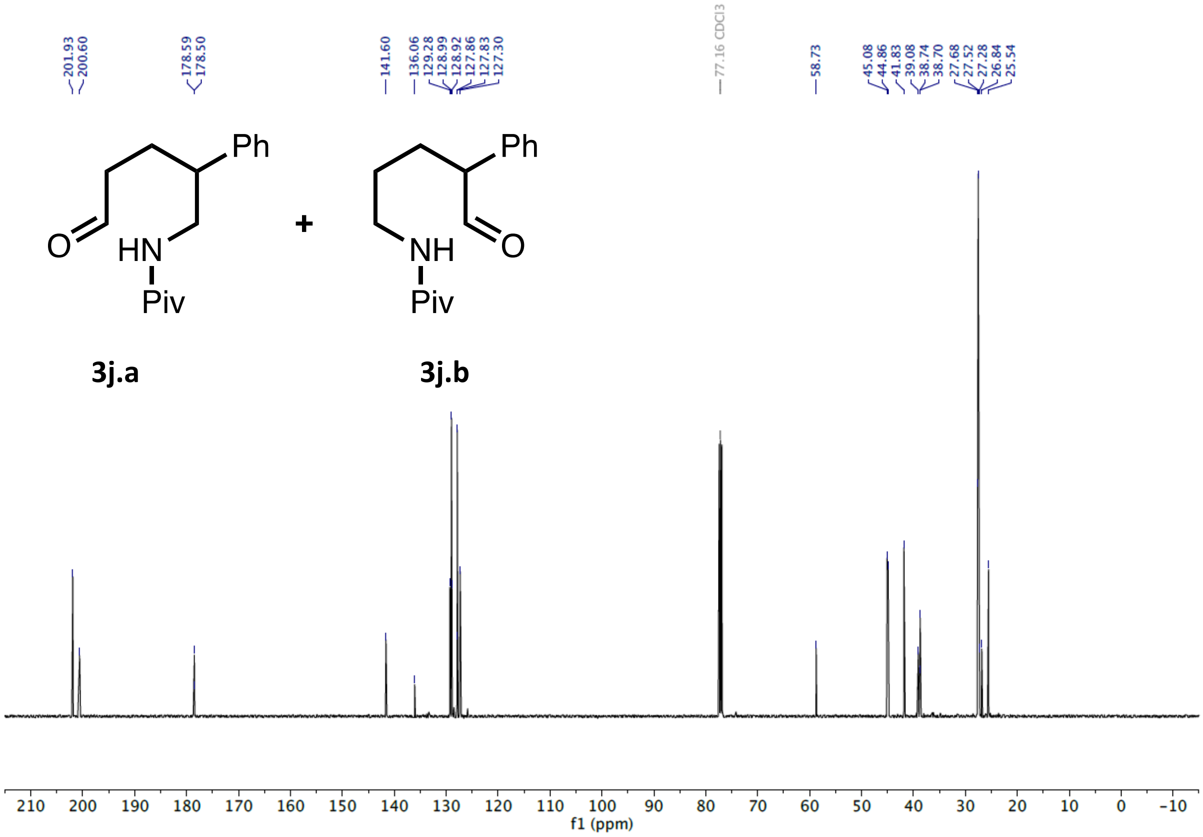

N-(2-formylphenethyl)pivalamide

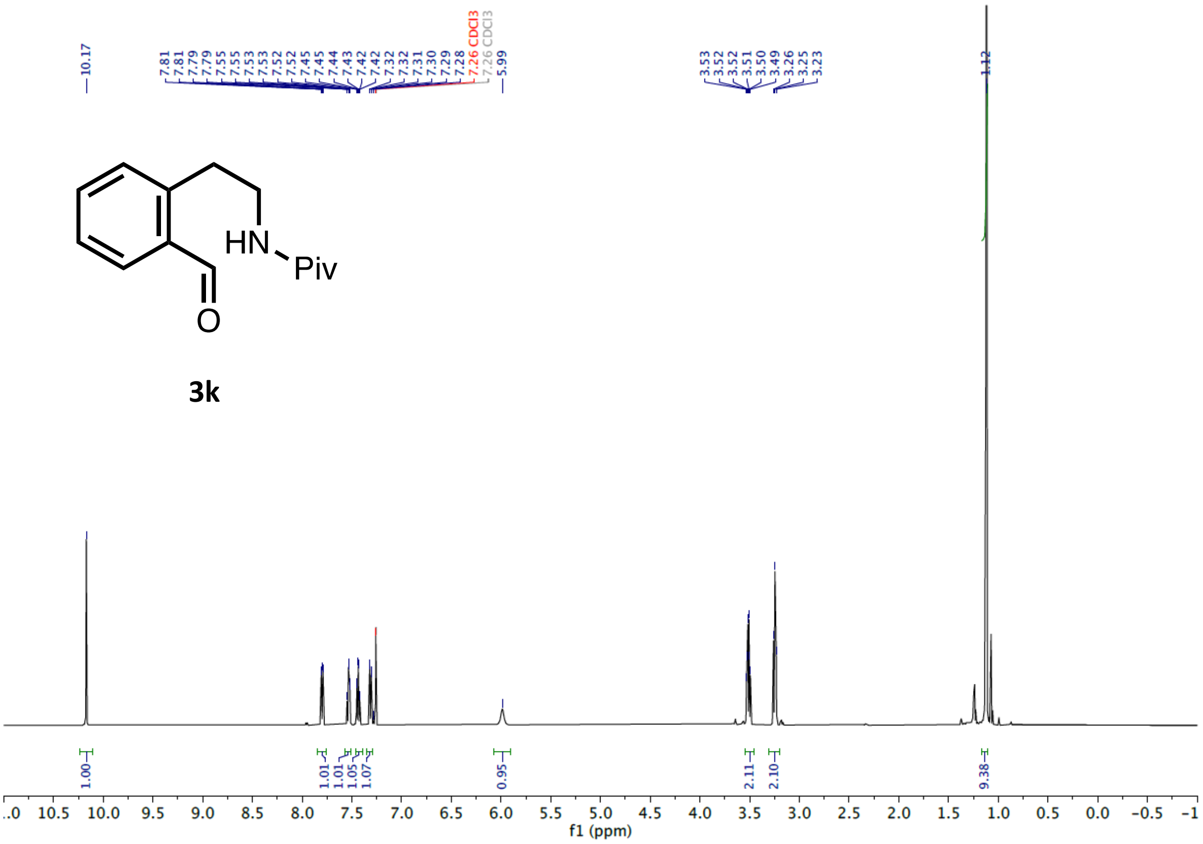

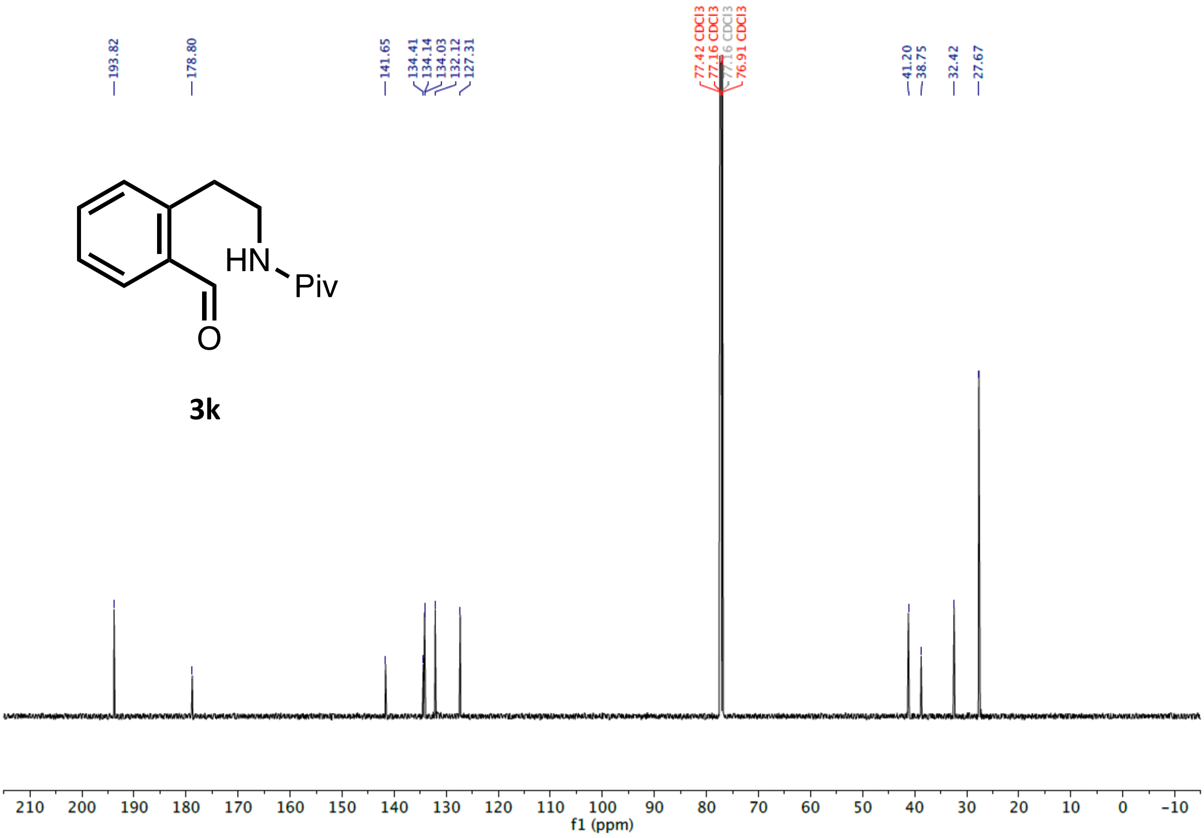

_N_-(2-formyl-4,5-dimethoxyphenethyl)pivalamide

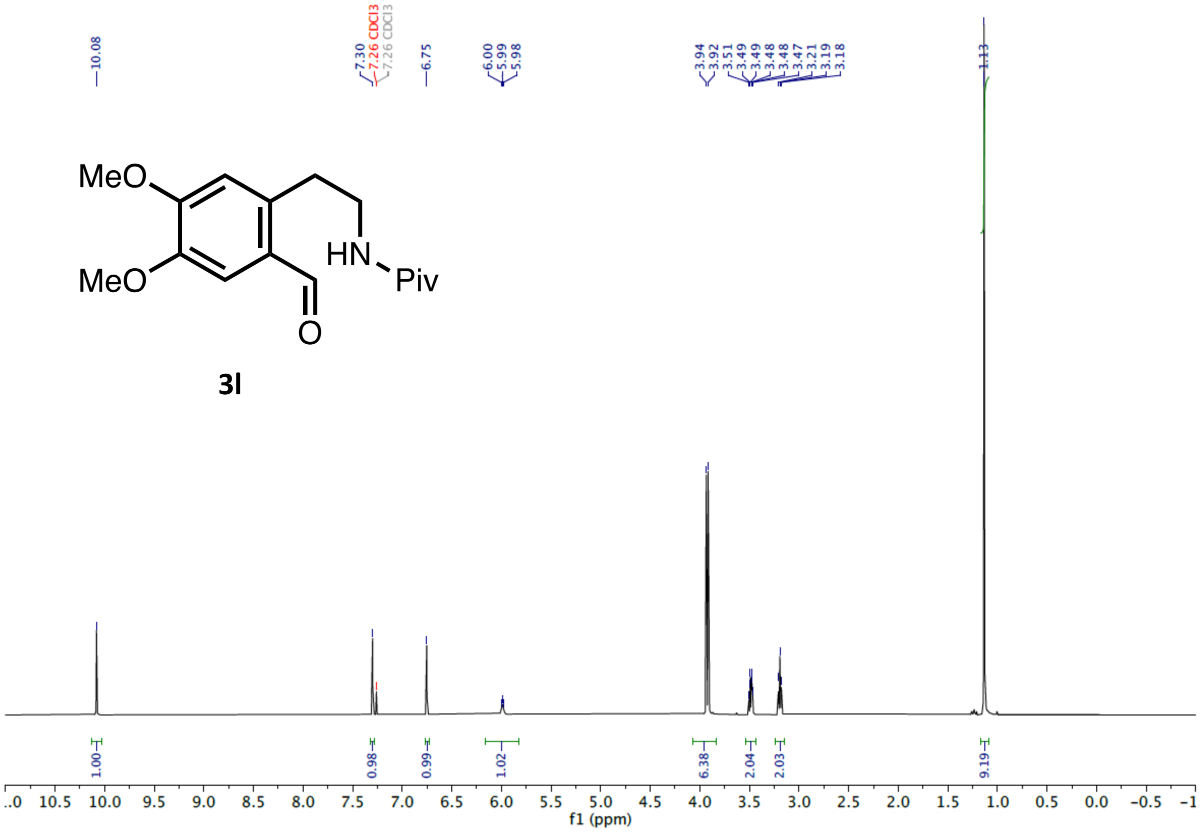

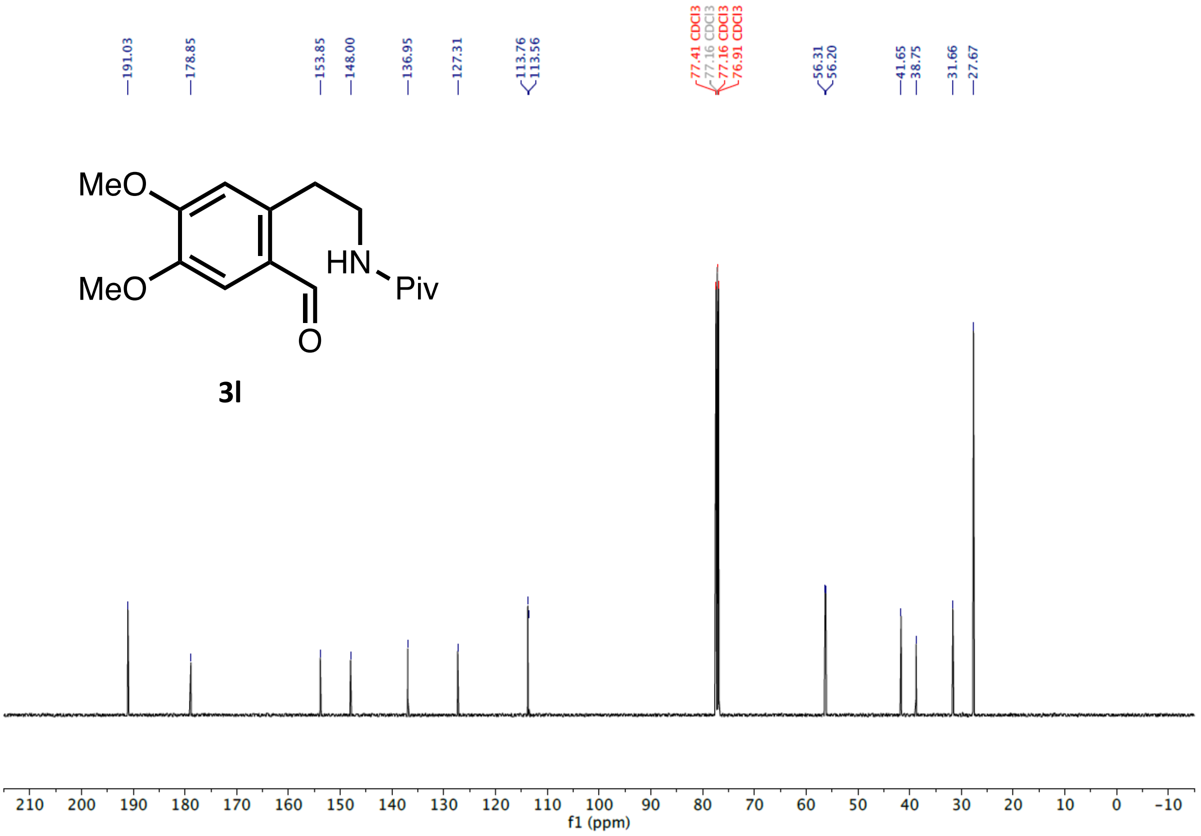

NMR Spectra – Acid Products

7-pivalamidoheptanoic acid

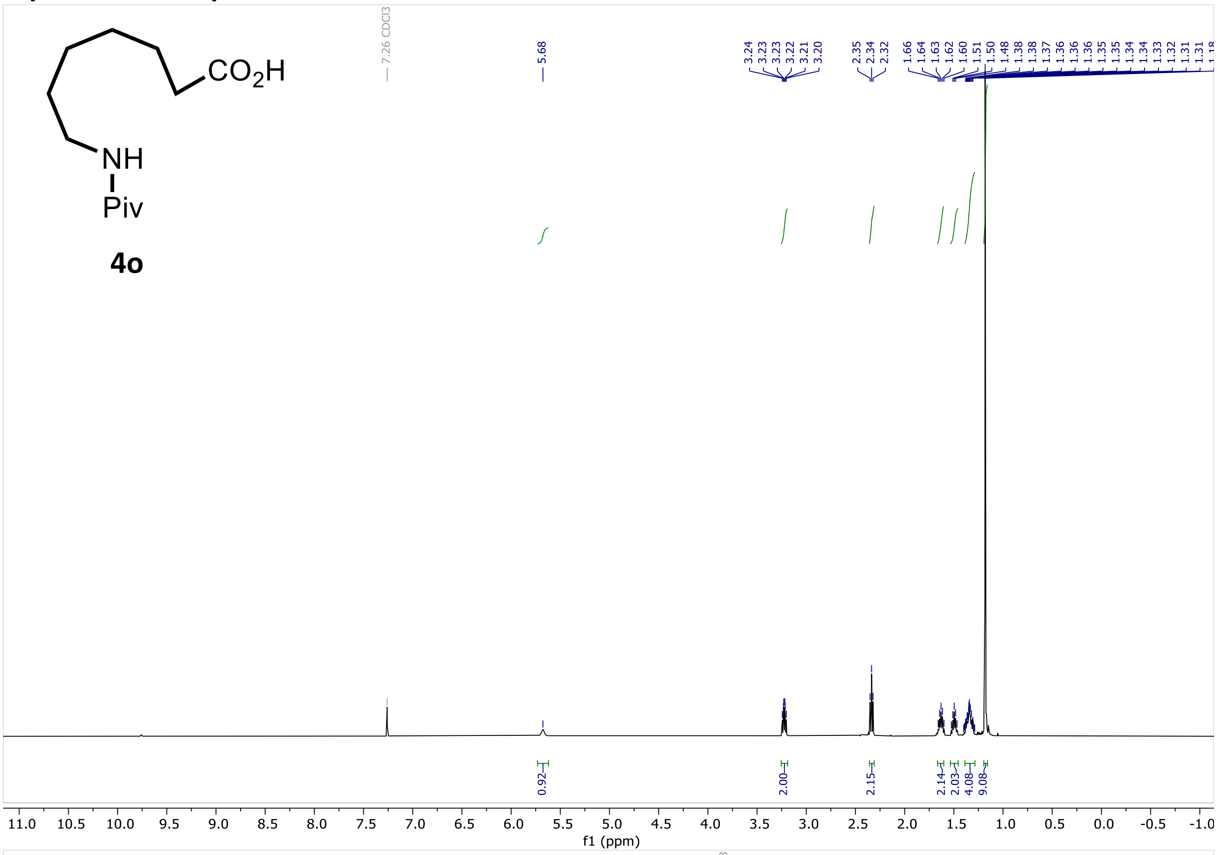

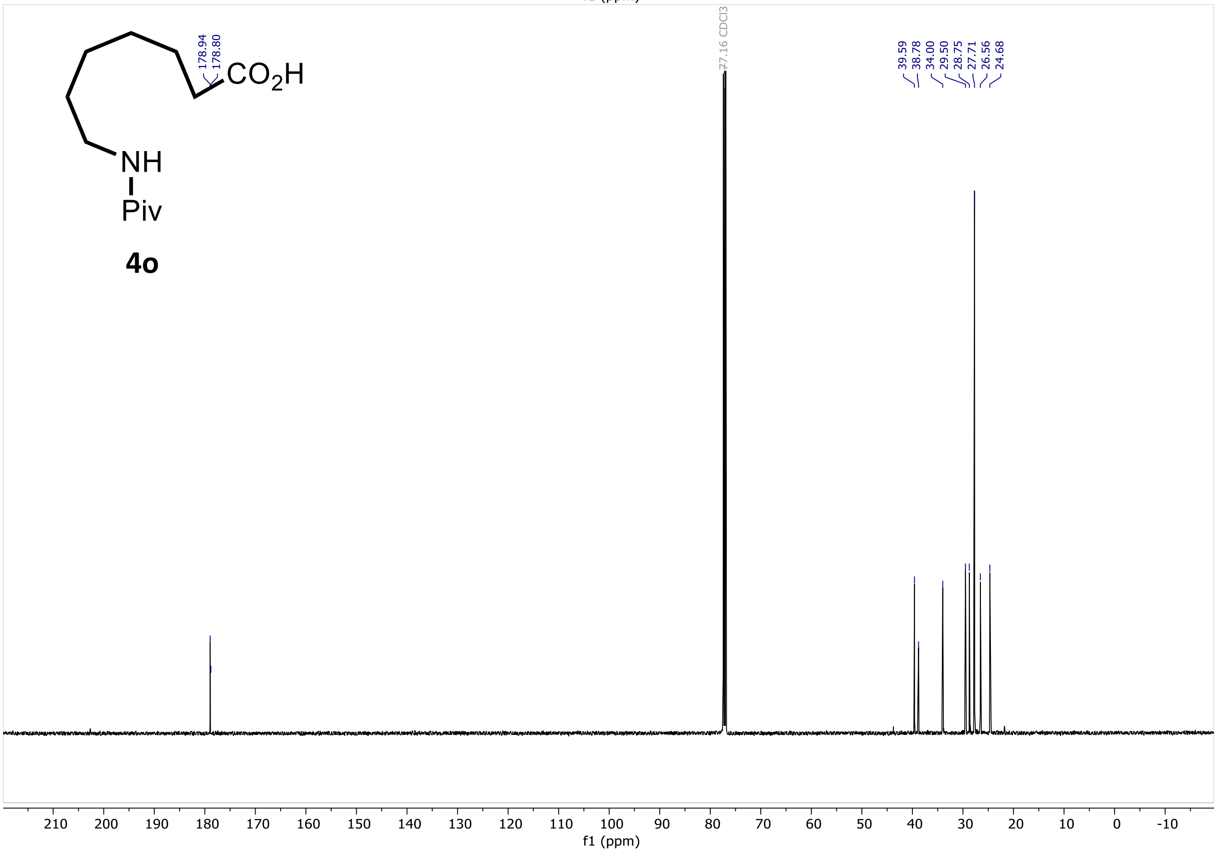

5-pivalamidohexanoic acid

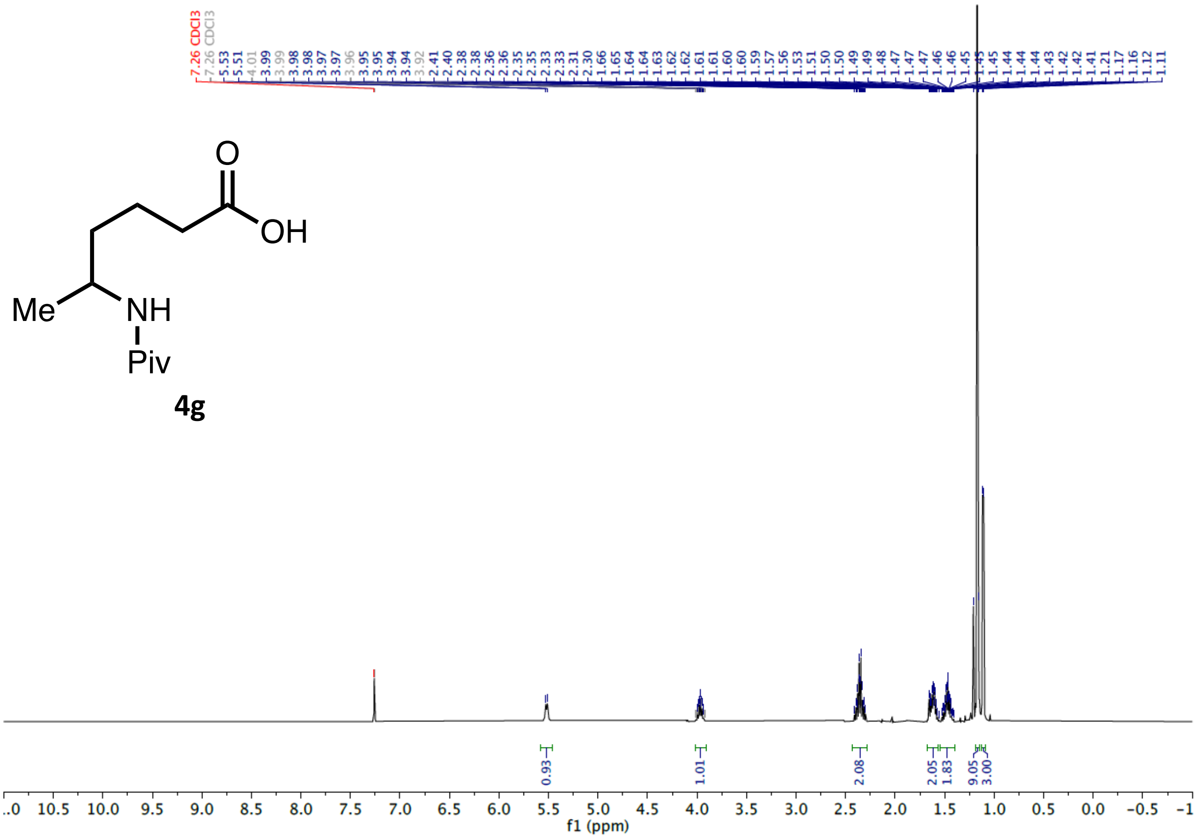

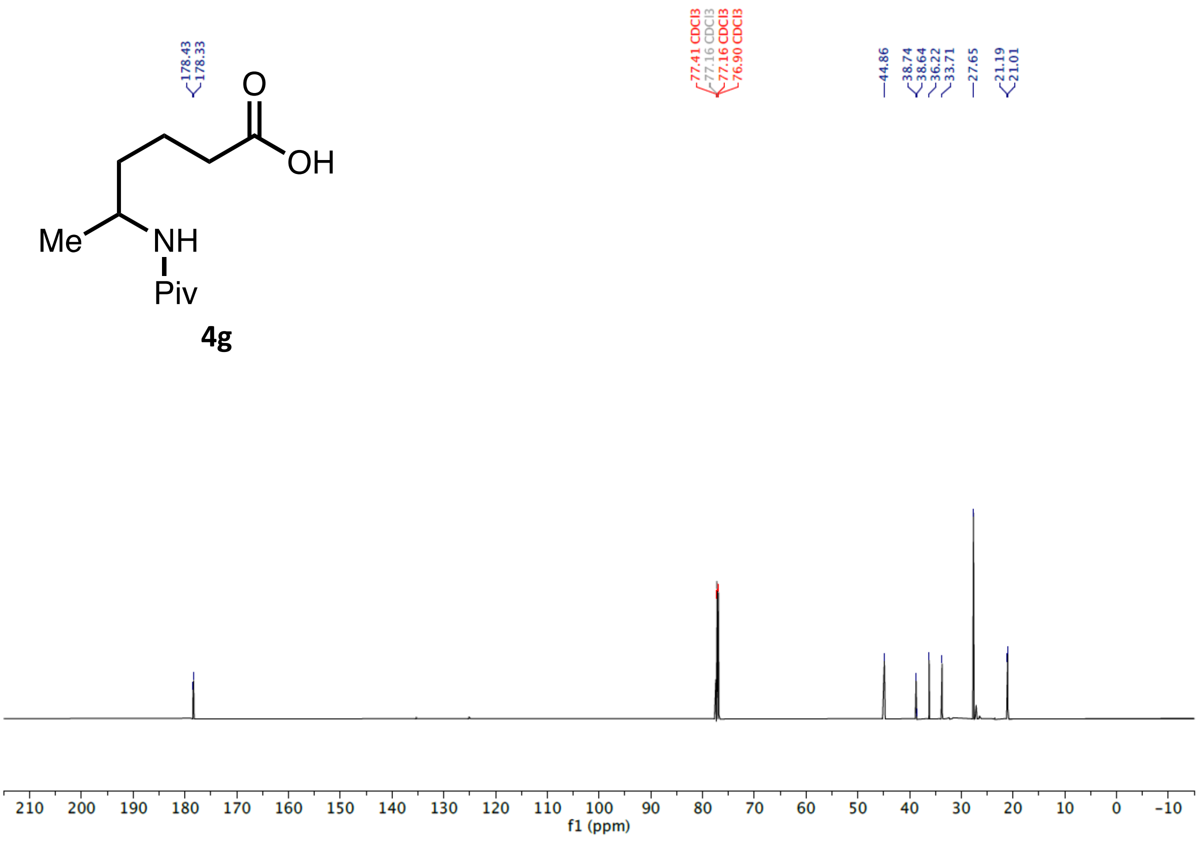

3-phenyl-5-pivalamidopentanoic acid

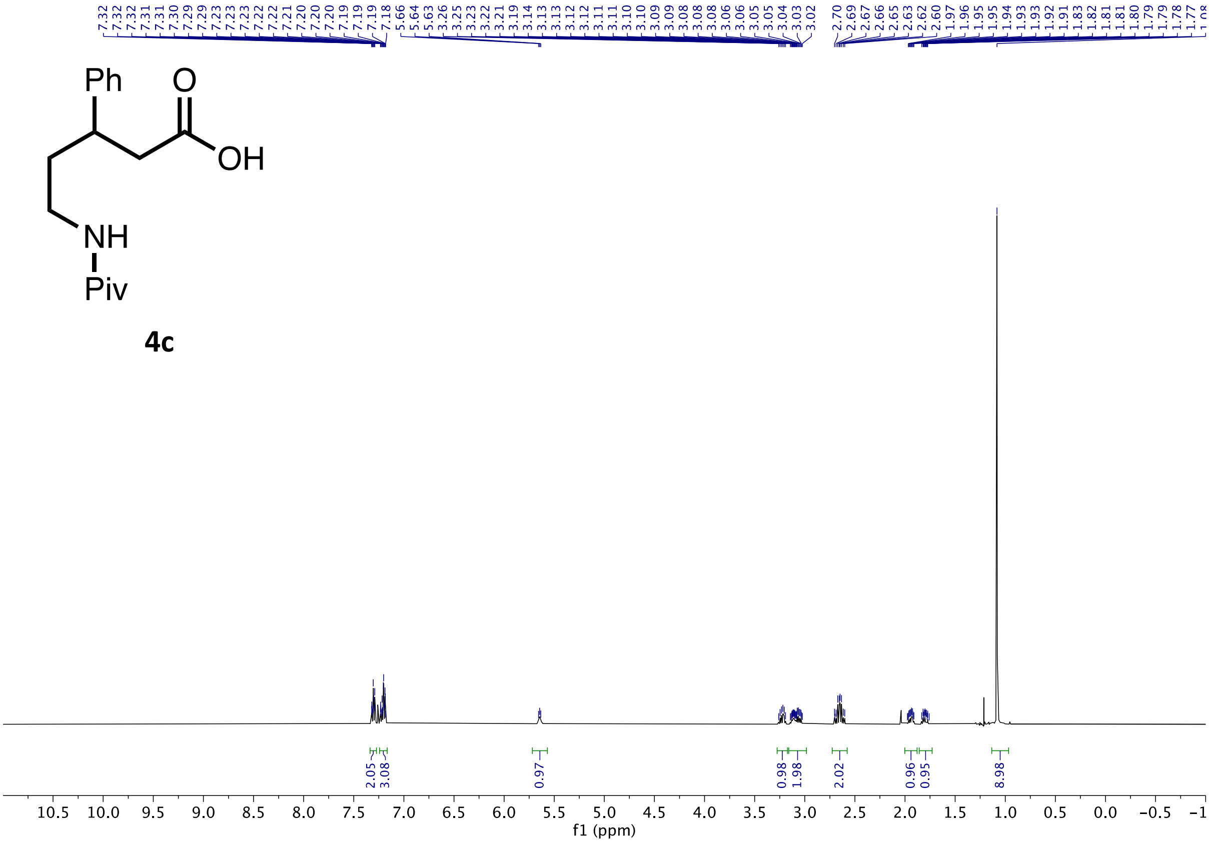

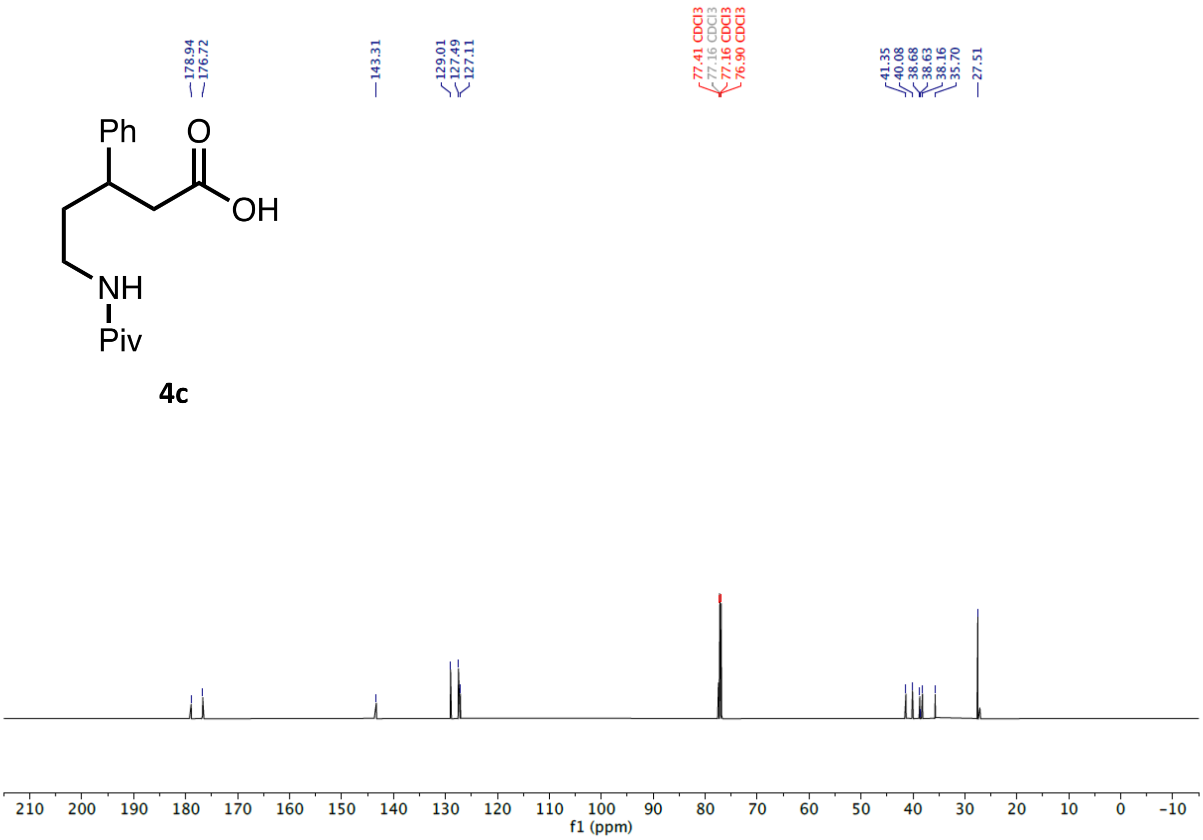

3-methyl-5-pivalamidopentanoic acid

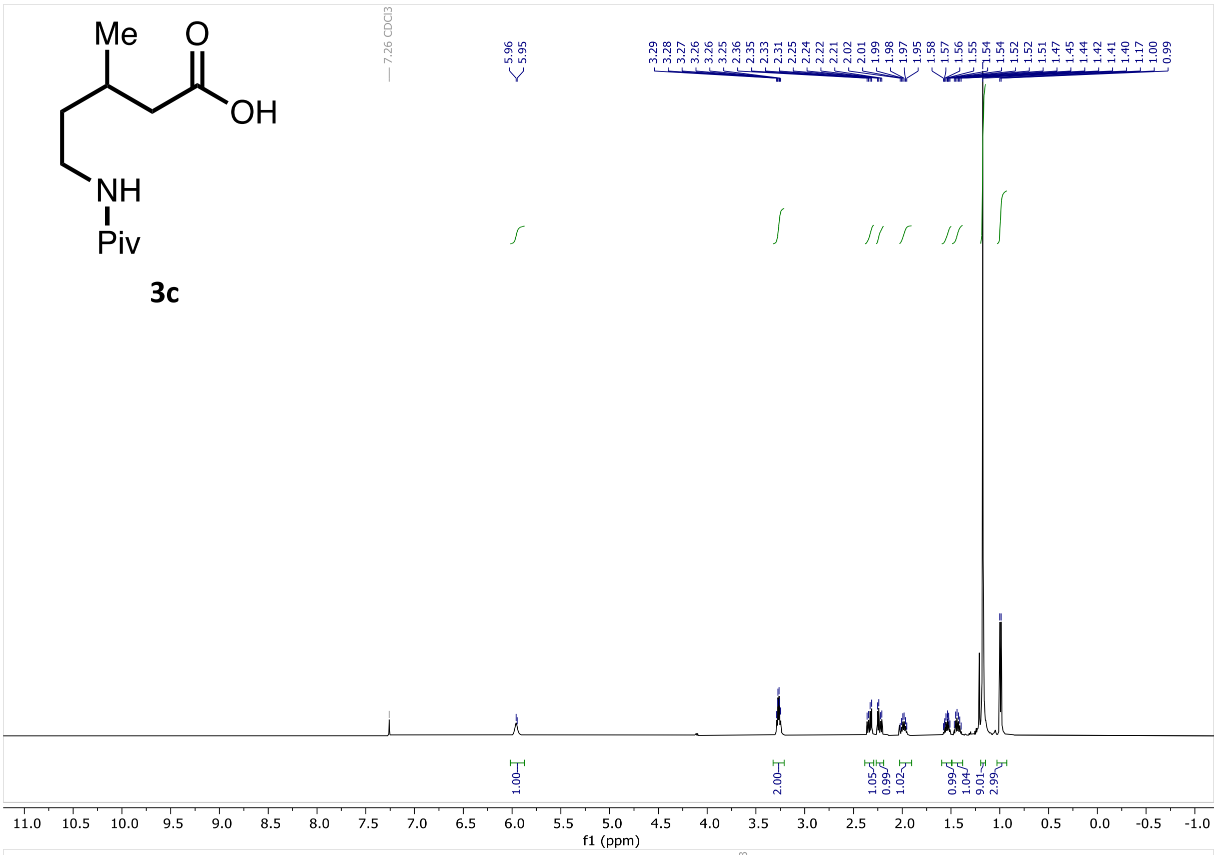

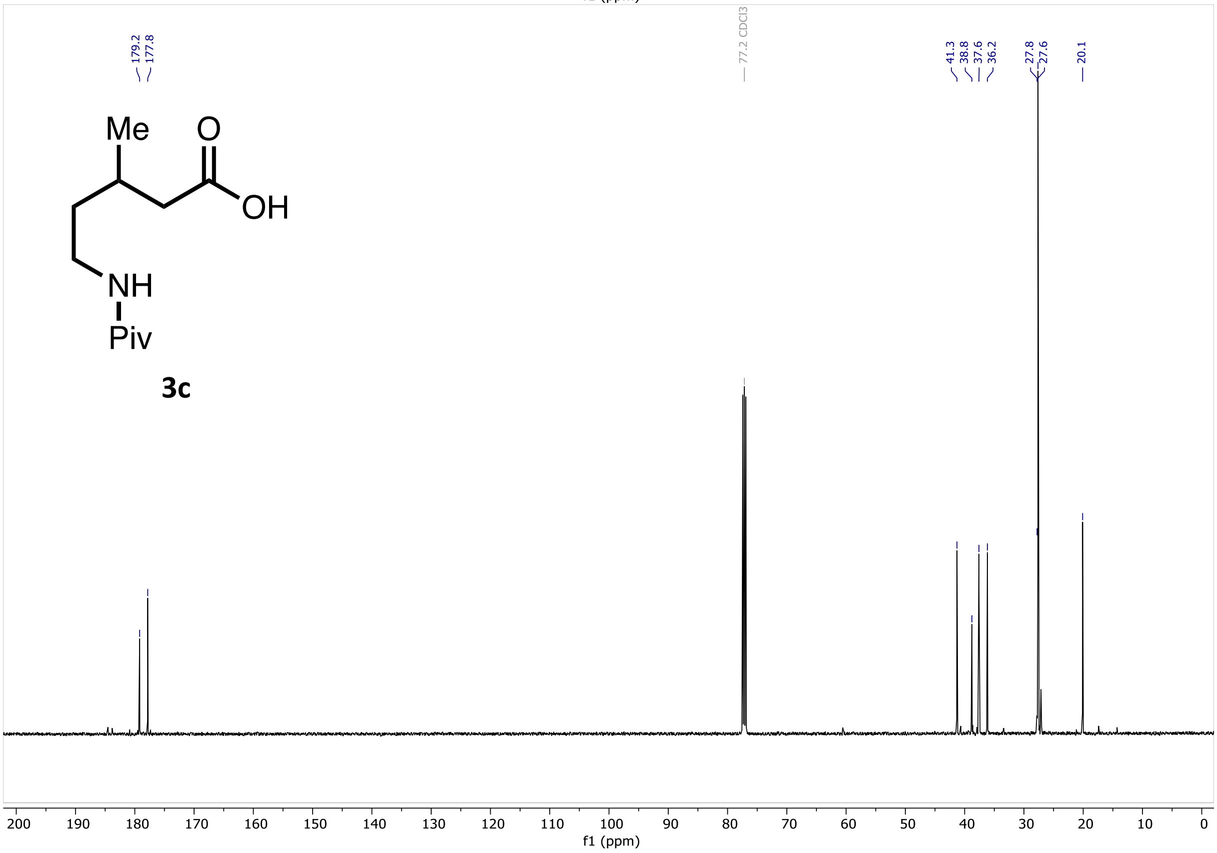

3-(ethoxycarbonyl)-5-pivalamidopentanoic acid

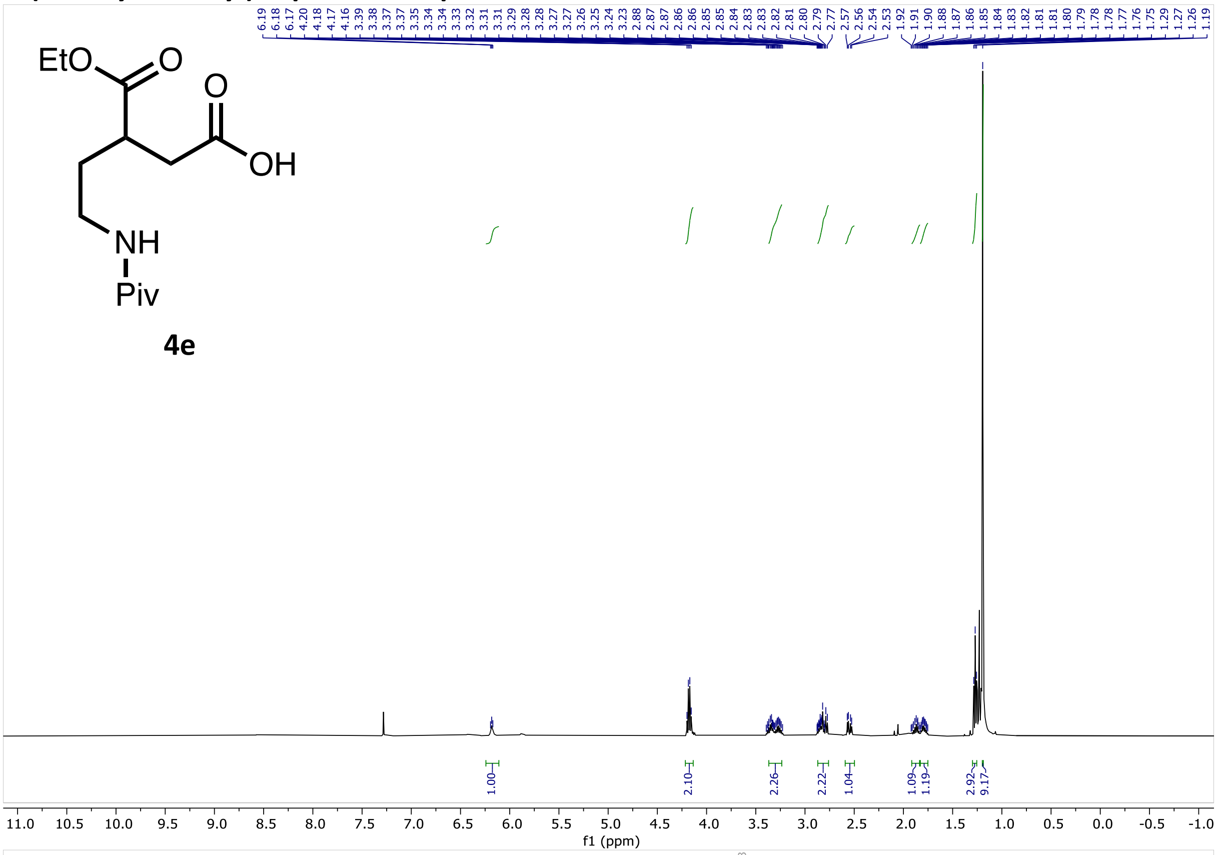

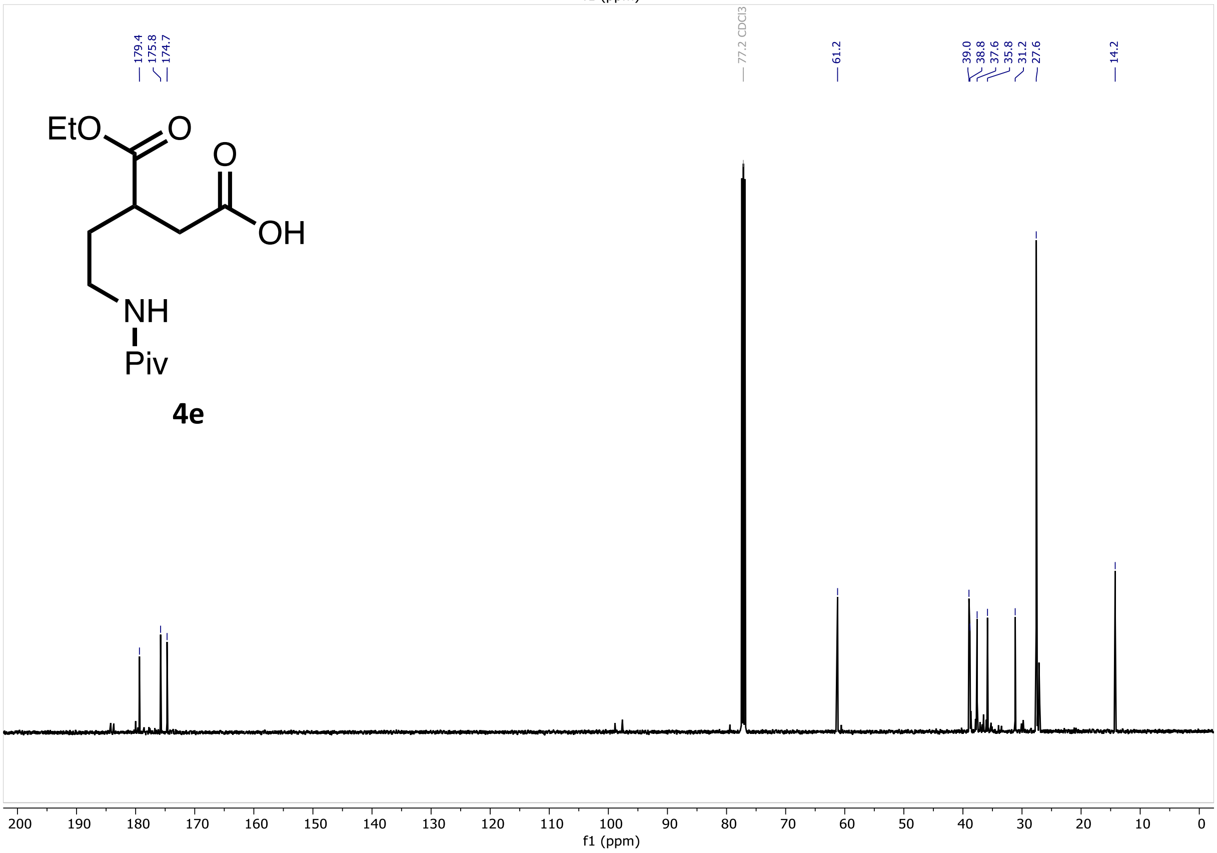

5-methoxy-5-oxo-3-(2-pivalamidoethyl)pentanoic acid

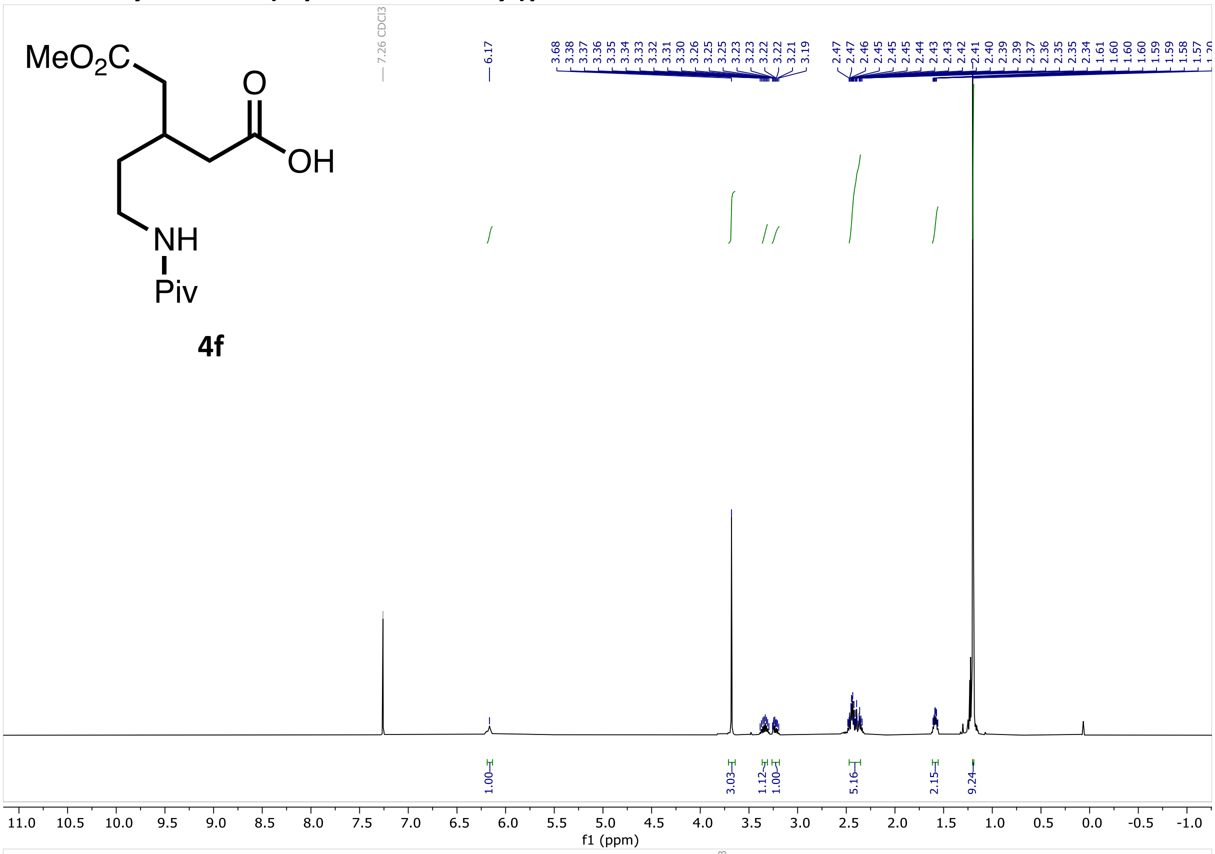

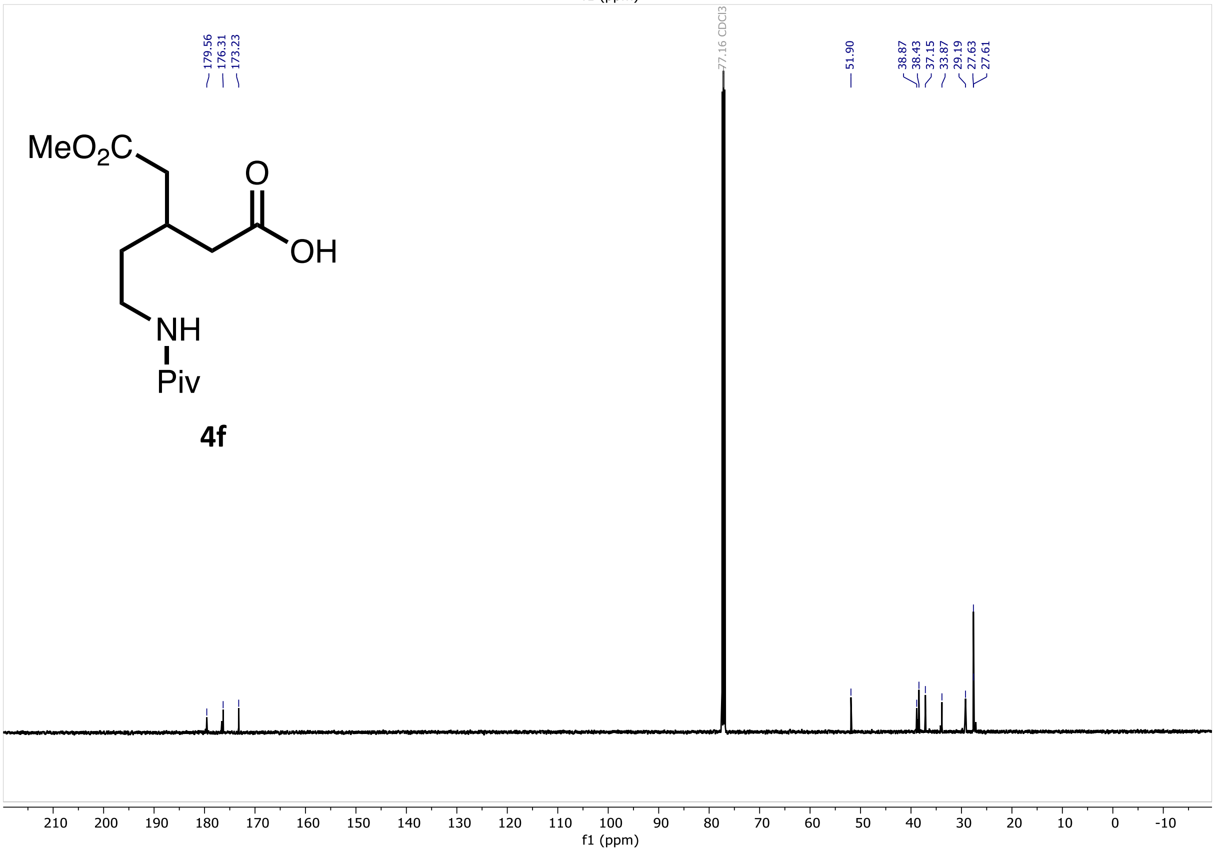

2,4-dimethyl-5-pivalamidopentanoic acid

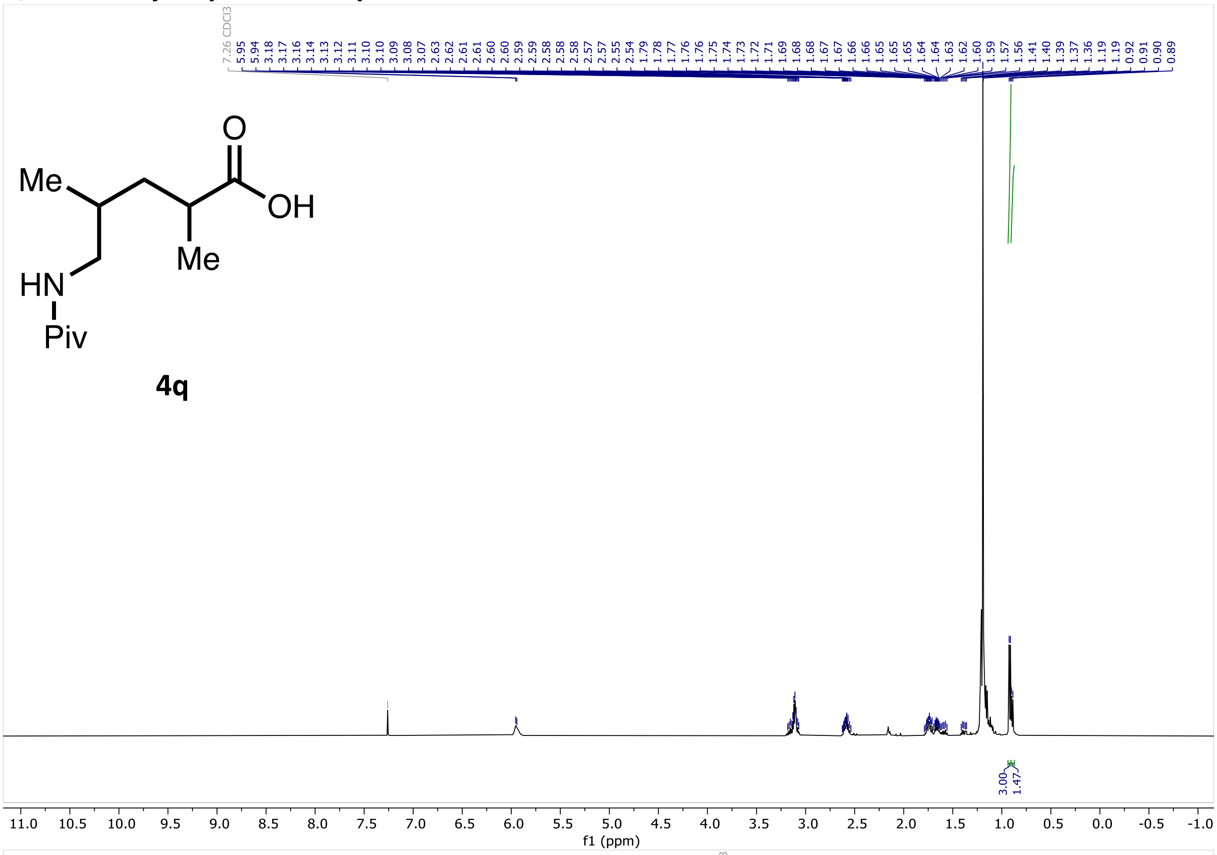

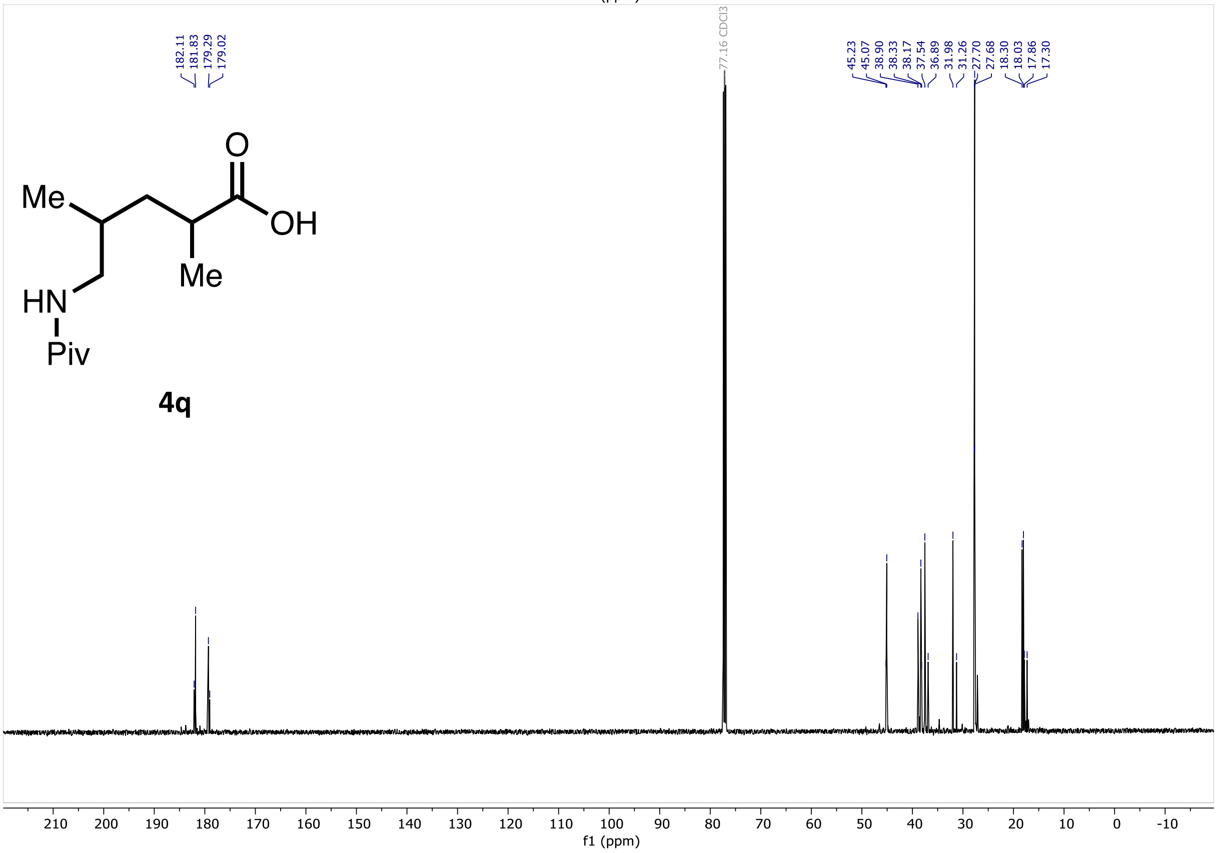

3-(2-pivalamidocyclohexyl)propanoic acid

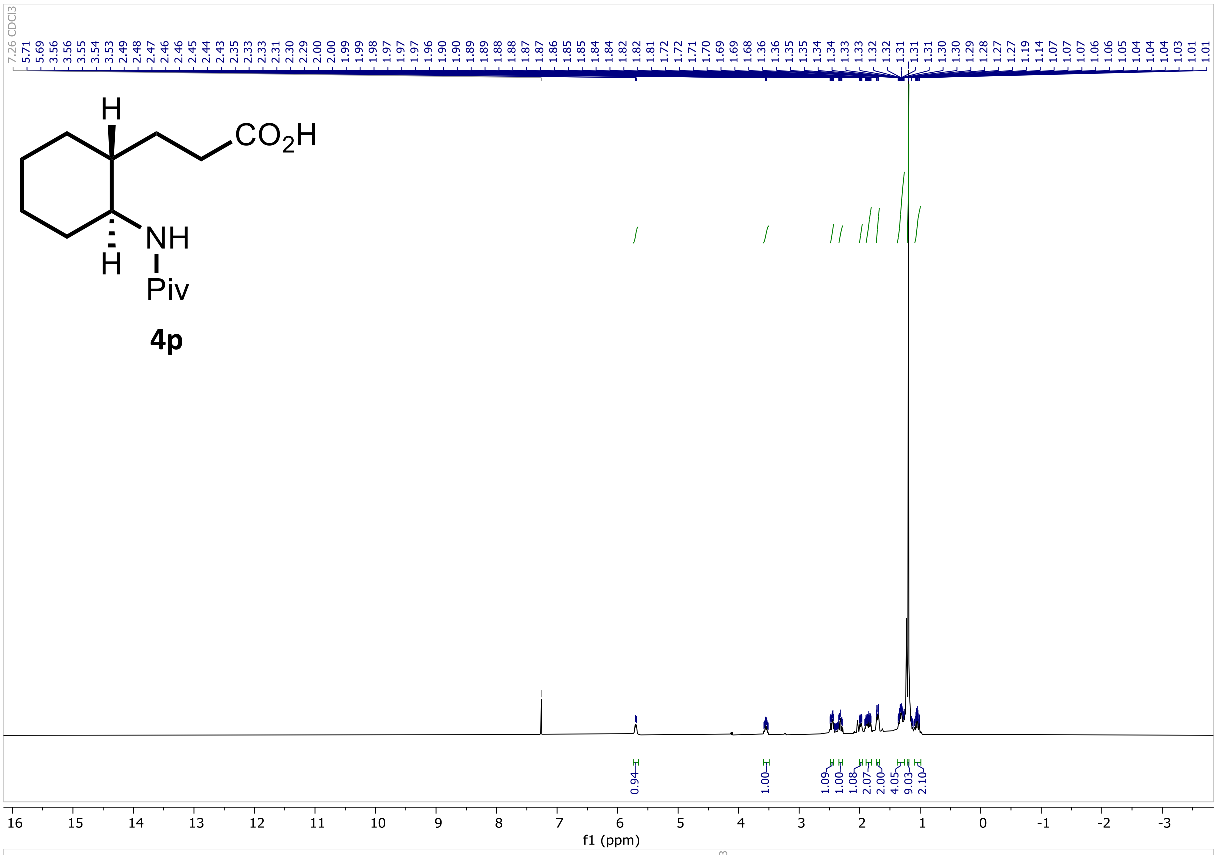

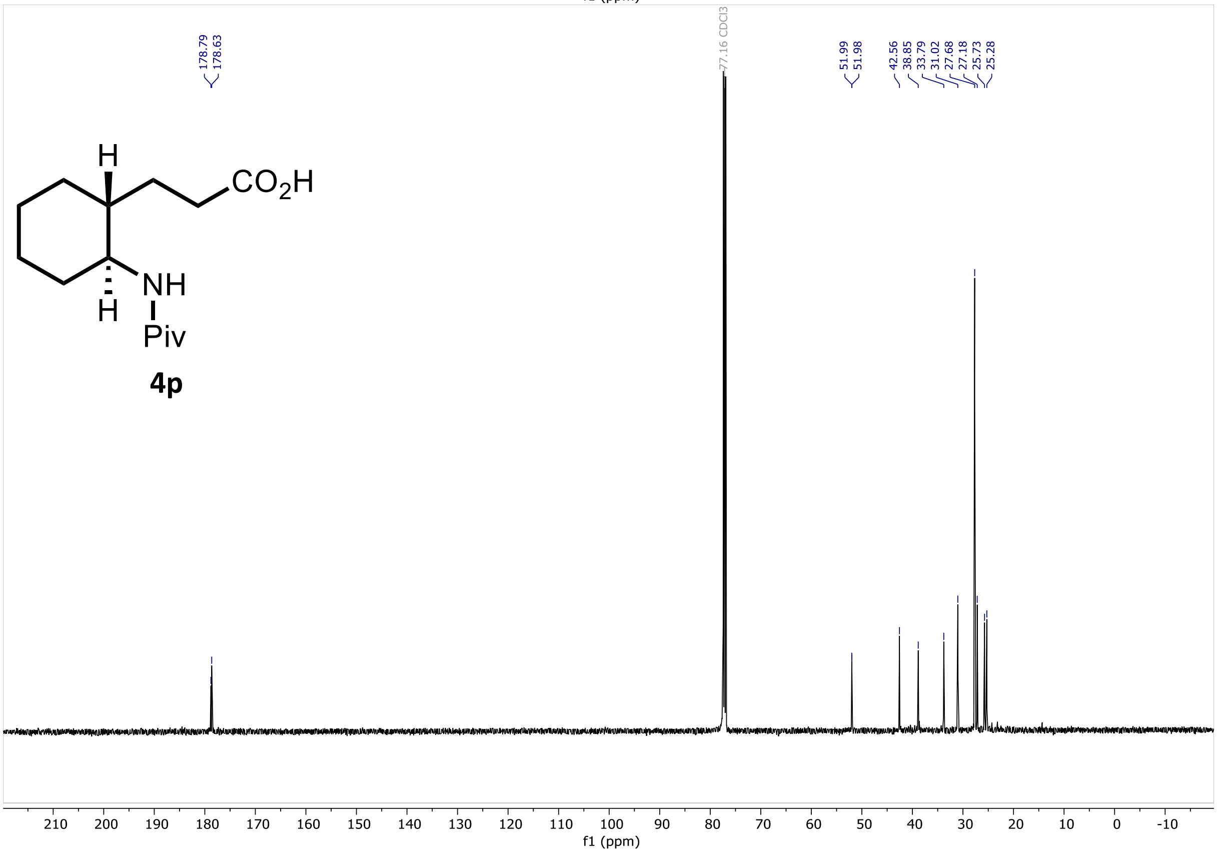

(*S*)-6-methoxy-6-oxo-5-pivalamidohexanoic acid

^{1}H NMR (500 MHz, $CDCl_3$) δ 9.73 (s, 1H), 6.32 (d, *J* = 7.6 Hz, 1H), 4.59 (td, *J* = 7.5, 5.1 Hz, 1H), 3.74 (s, 3H), 2.70 – 2.23 (m, 2H), 1.91 (ddt, *J* = 12.3, 9.5, 5.1 Hz, 1H), 1.78 – 1.52 (m, 3H), 1.21 (s, 9H).

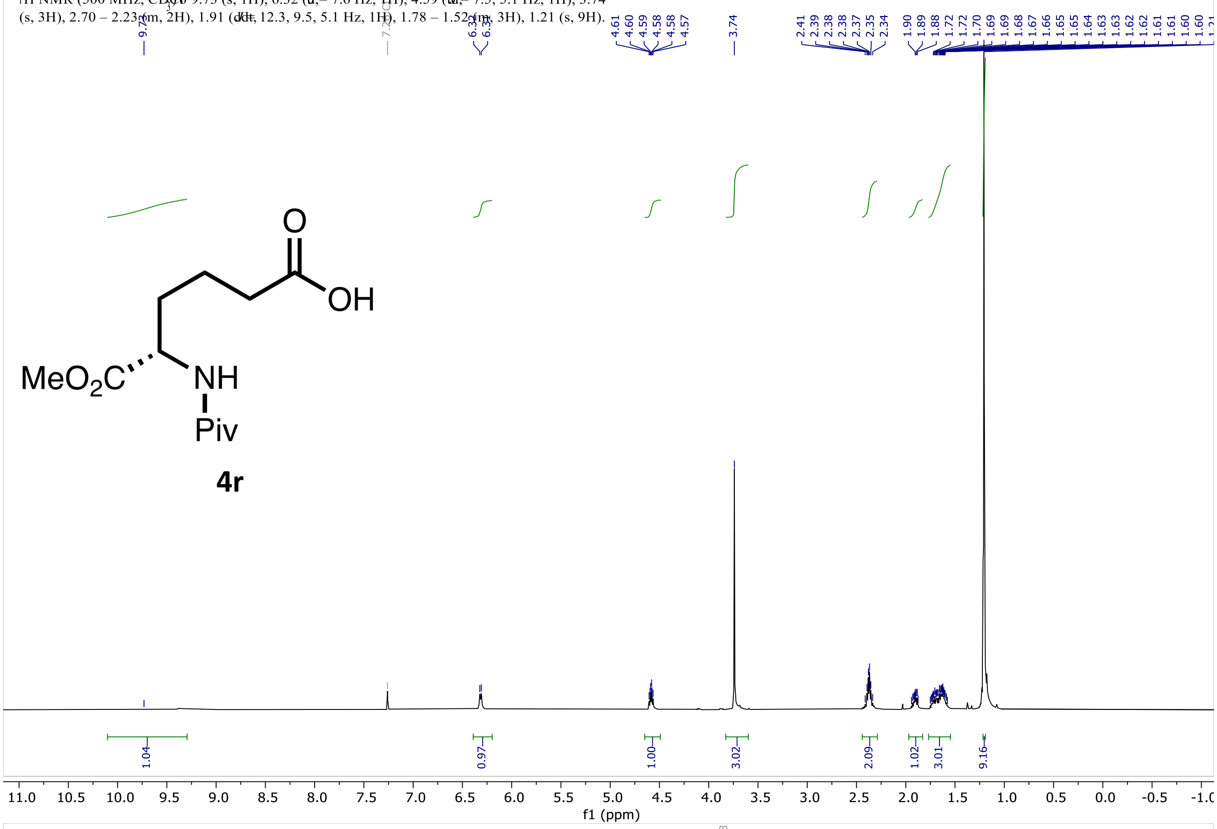

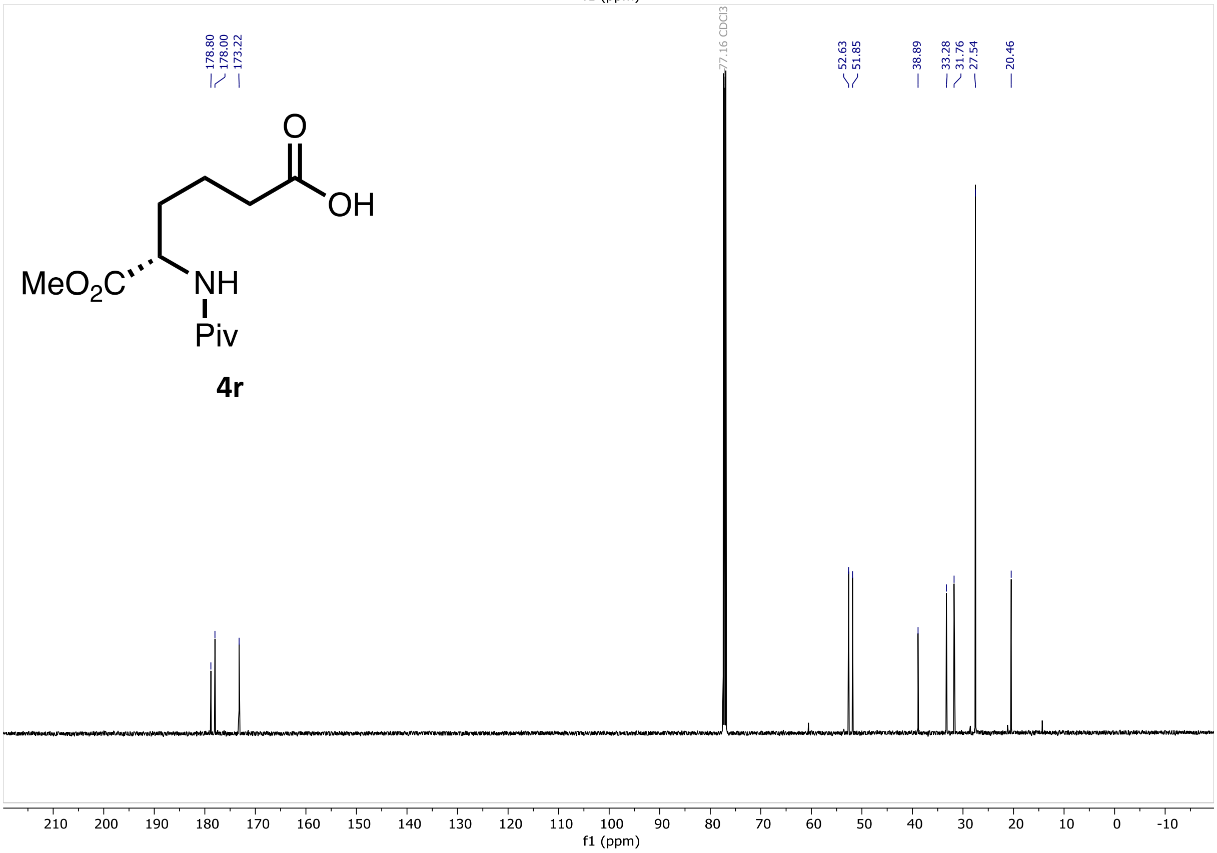

(*S*)-5-methoxy-5-oxo-4-pivalamidopentanoic acid

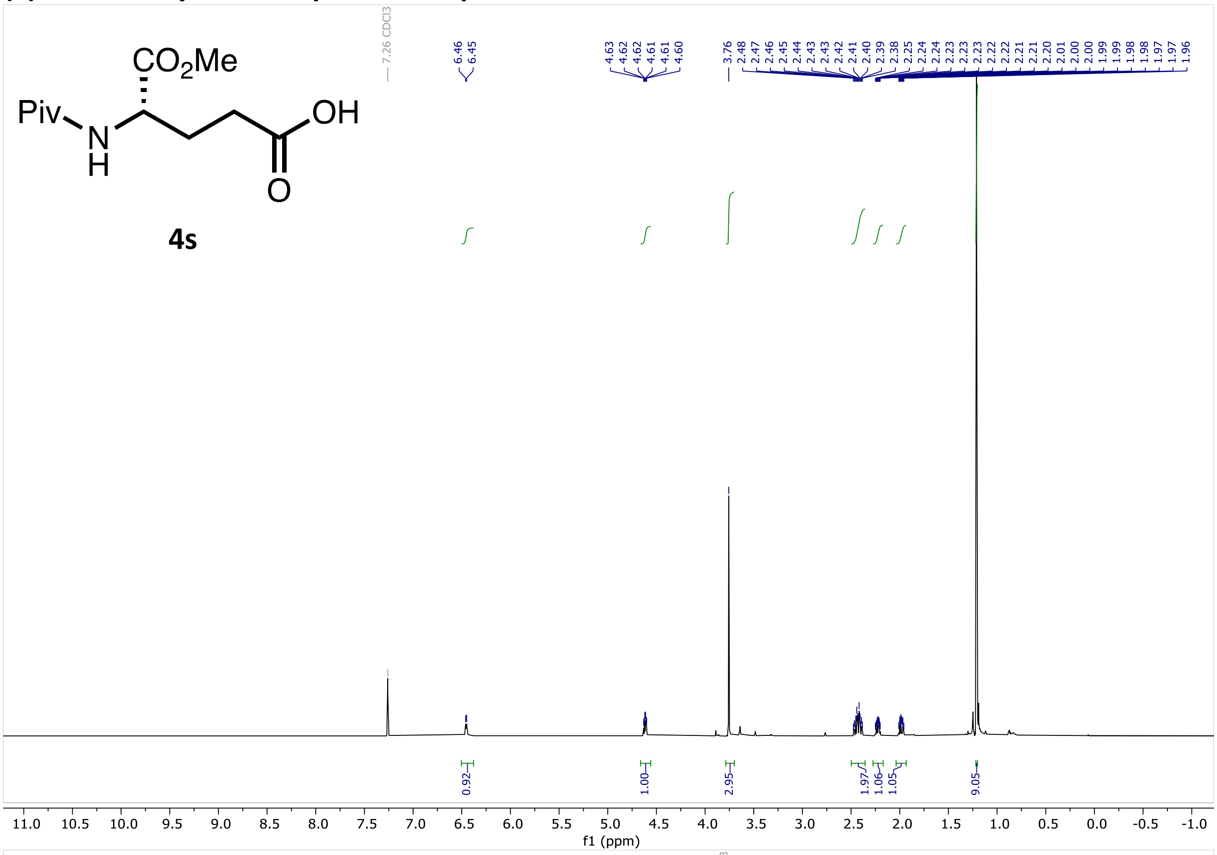

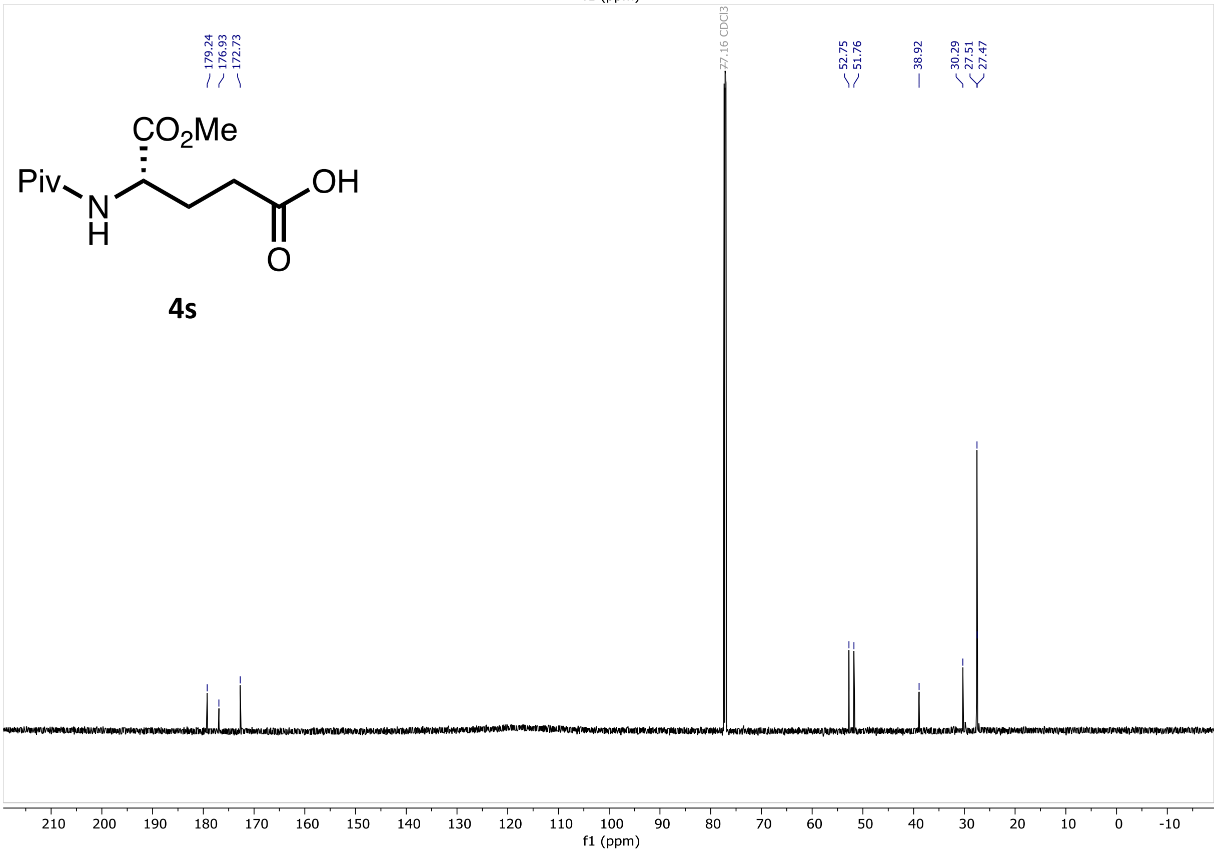

***N*-(5-oxo-5-phenylpentyl)pivalamide**

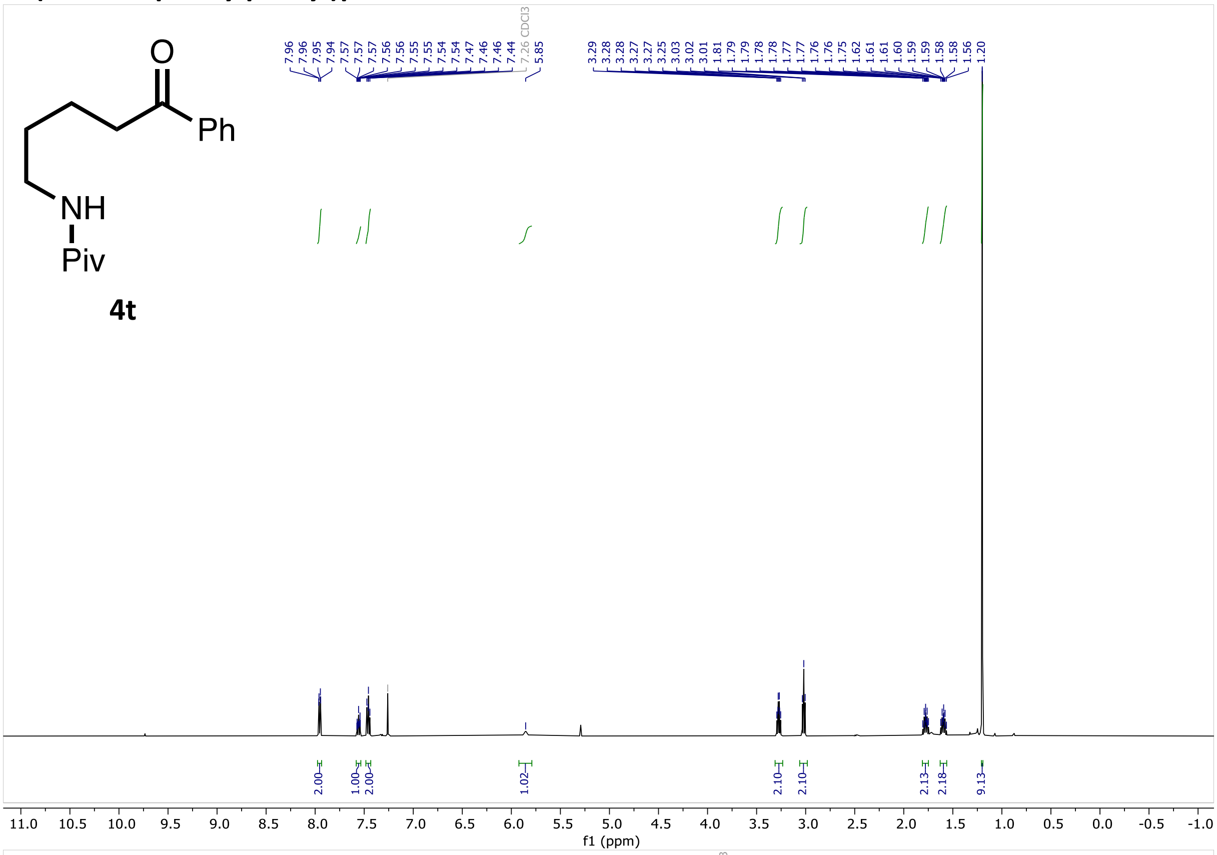

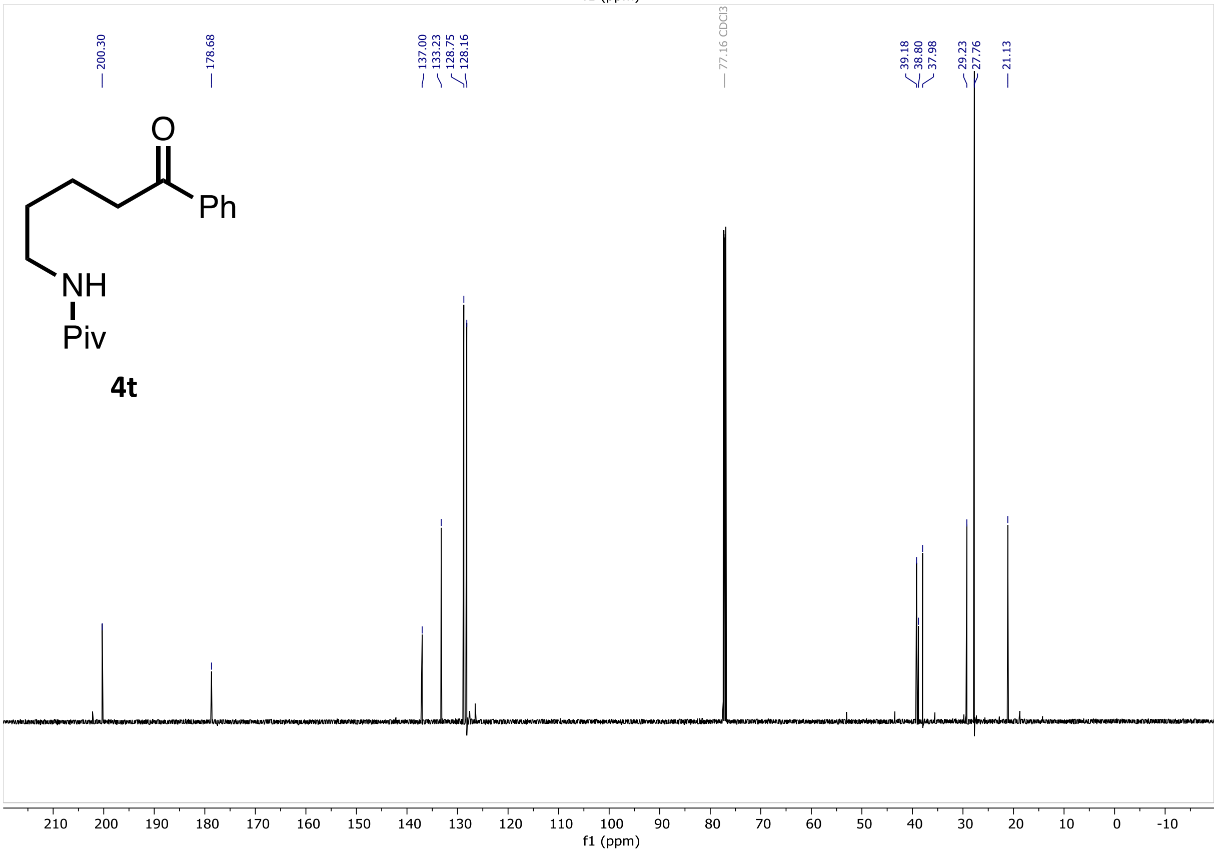

5-oxo-5-phenylpentanoic acid

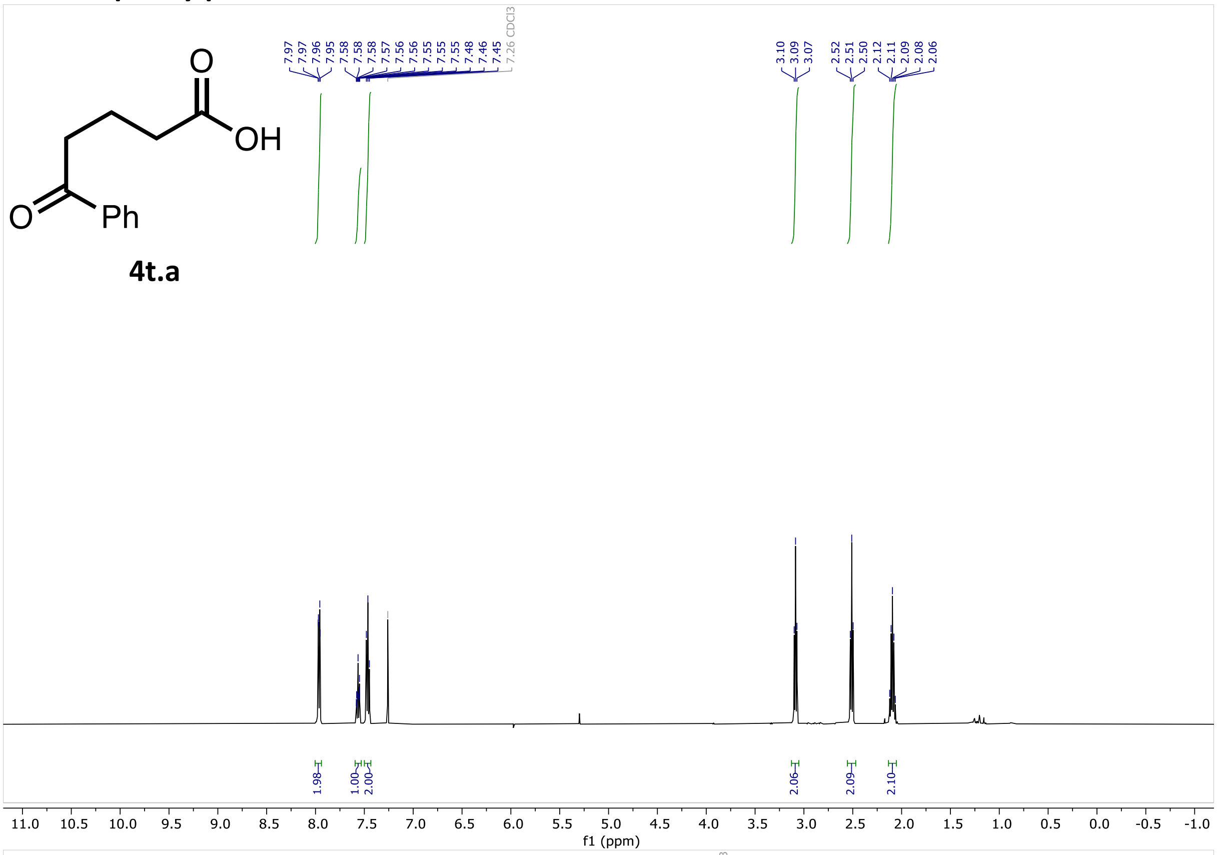

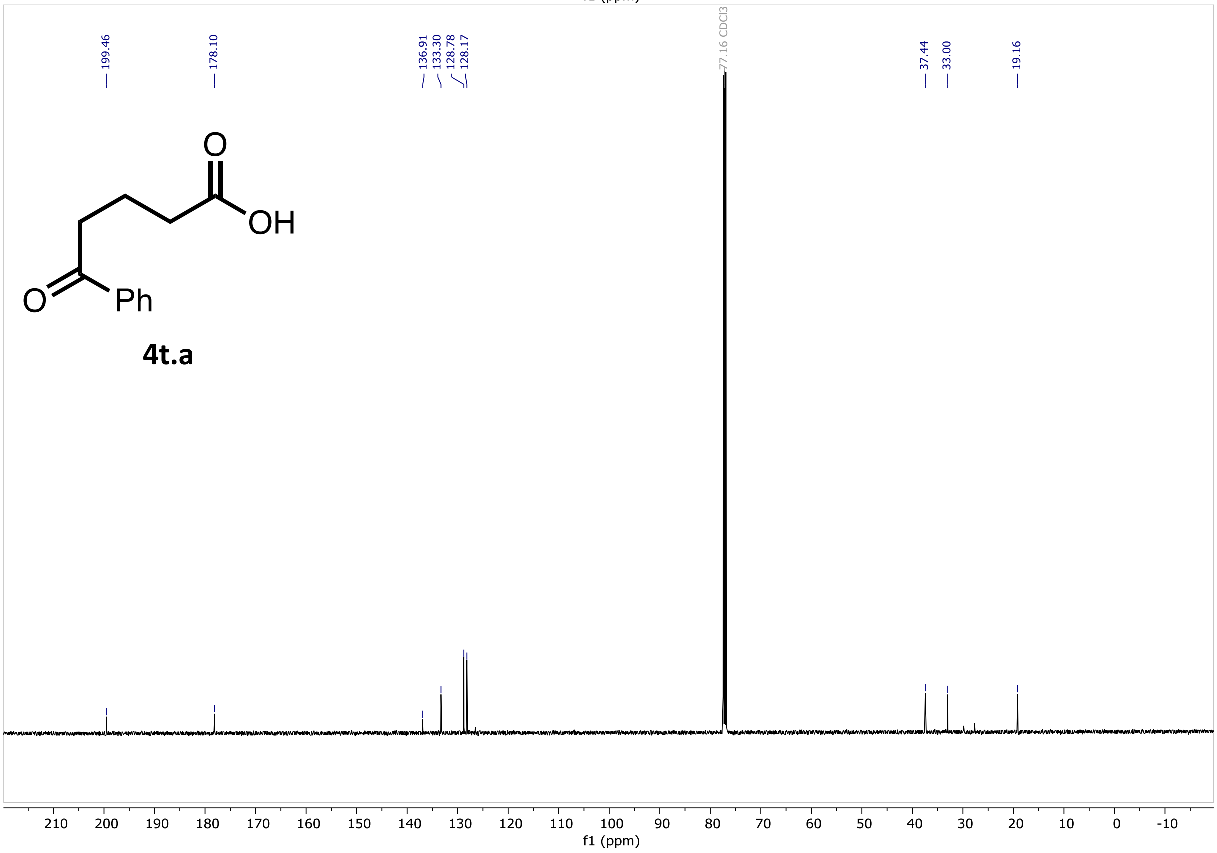

5-oxo-5-phenylpentanoic acid

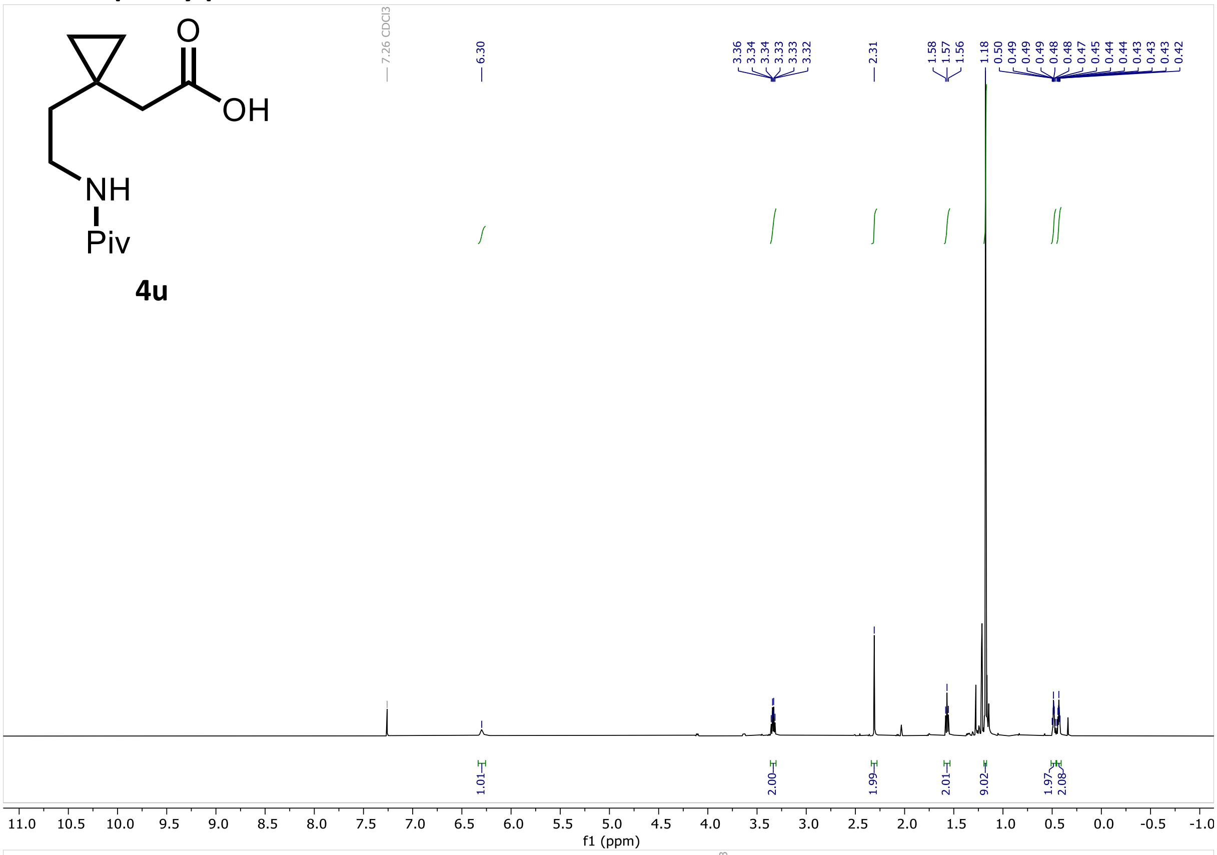

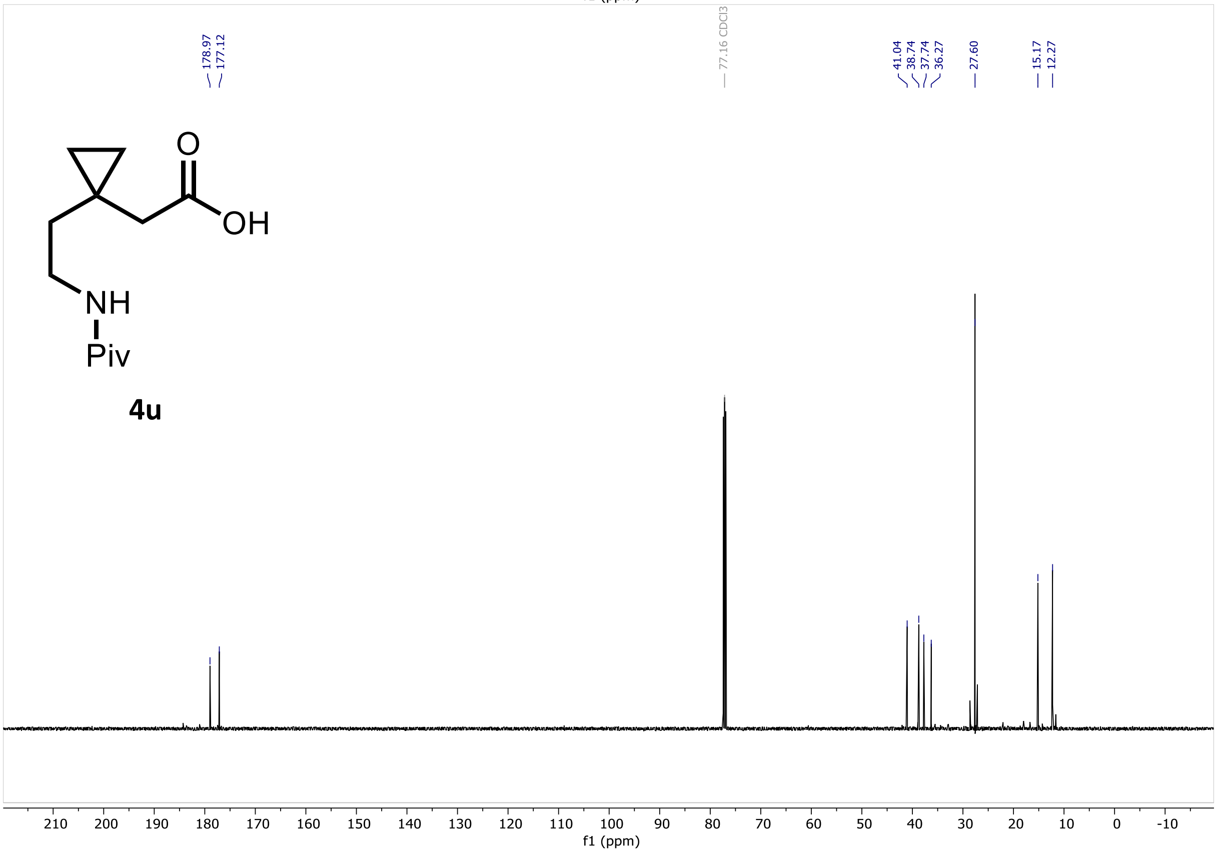

(4*S*)-5-(((3*R*)-3-(*tert*-butoxy)-1-methoxy-1-oxobutan-2-yl)amino)-5-oxo-4-pivalamidopent-anoic acid

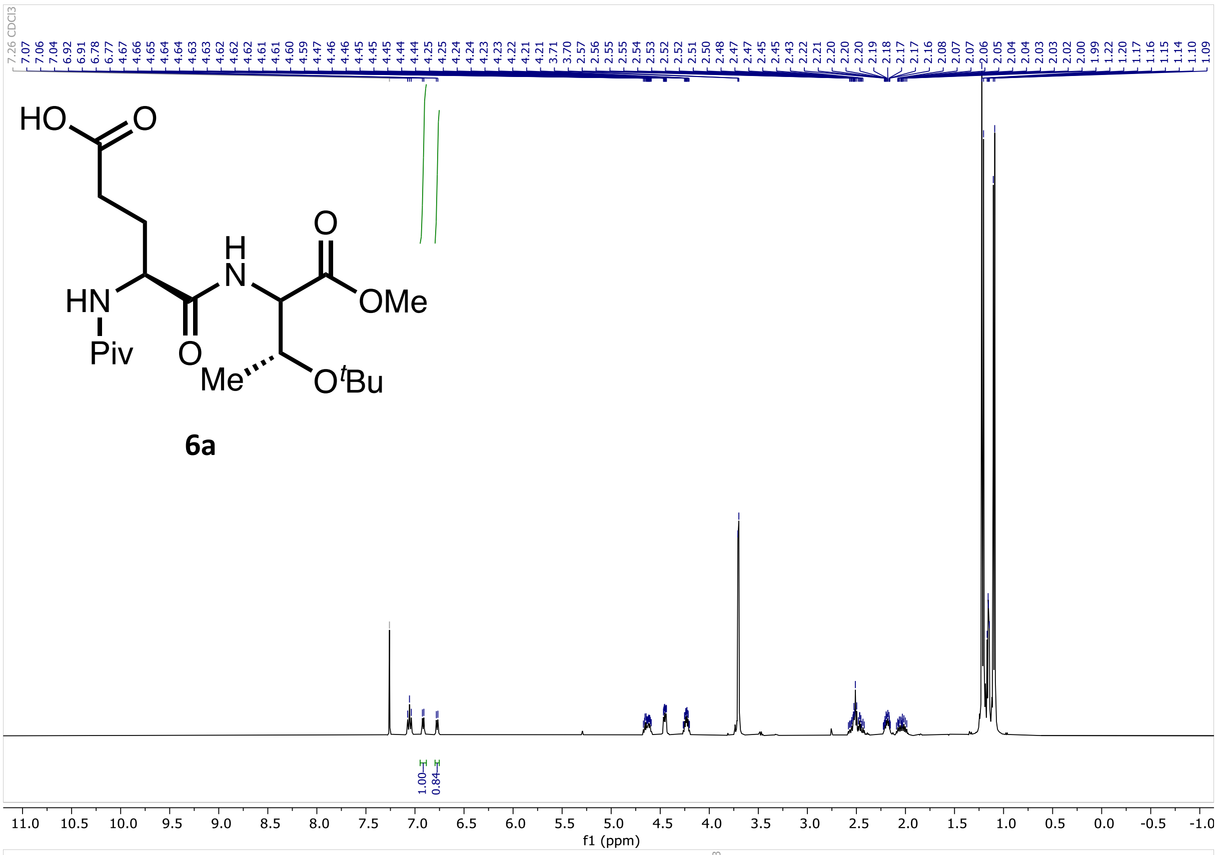

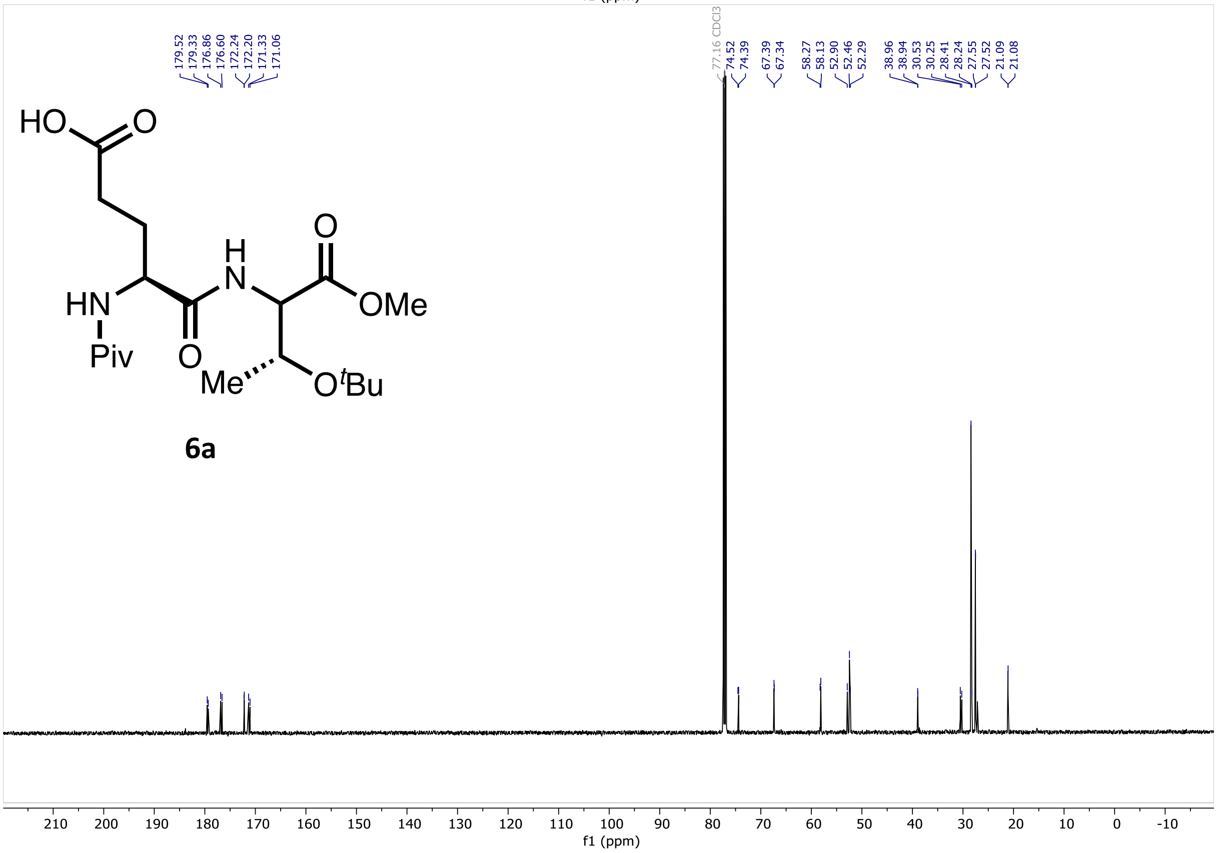

(*S*)-5-(((*S*)-1-methoxy-3-methyl-1-oxobutan-2-yl)amino)-5-oxo-4-pivalamidopentanoic acid

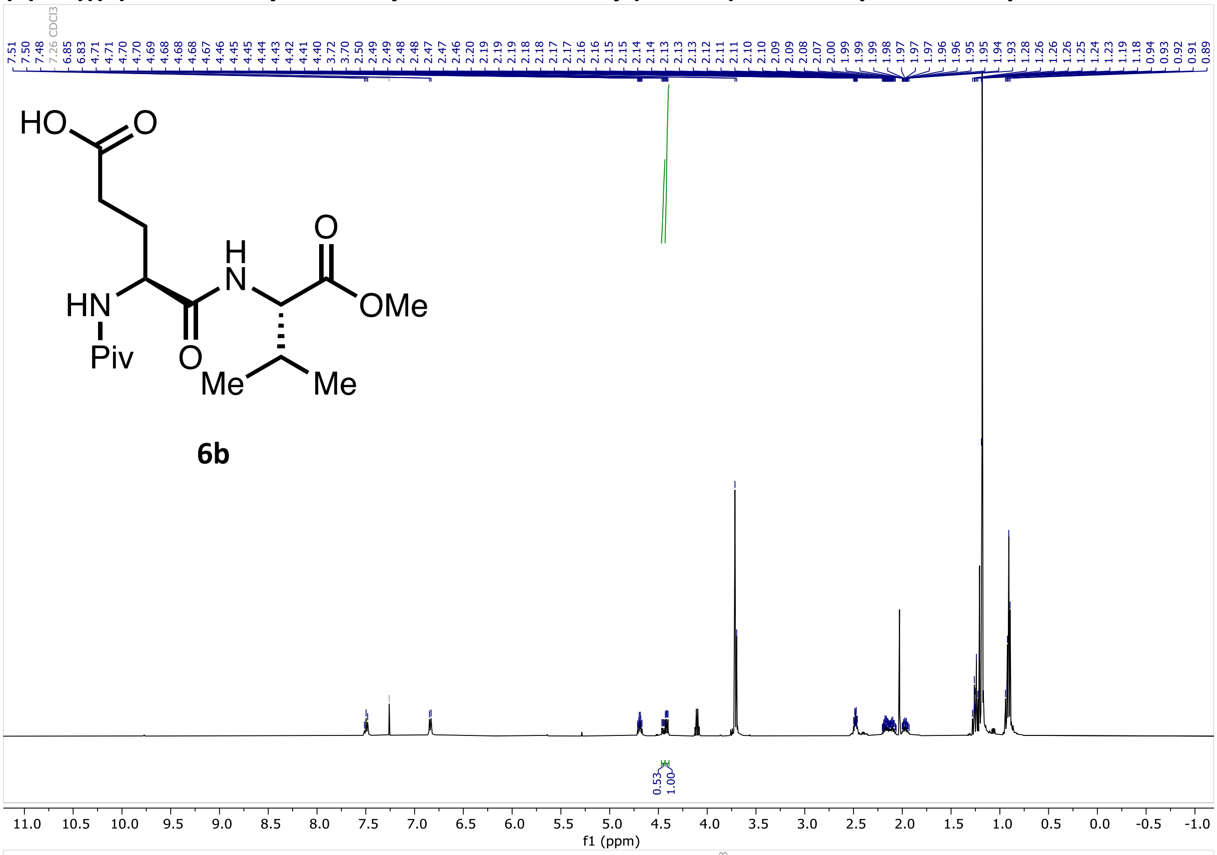

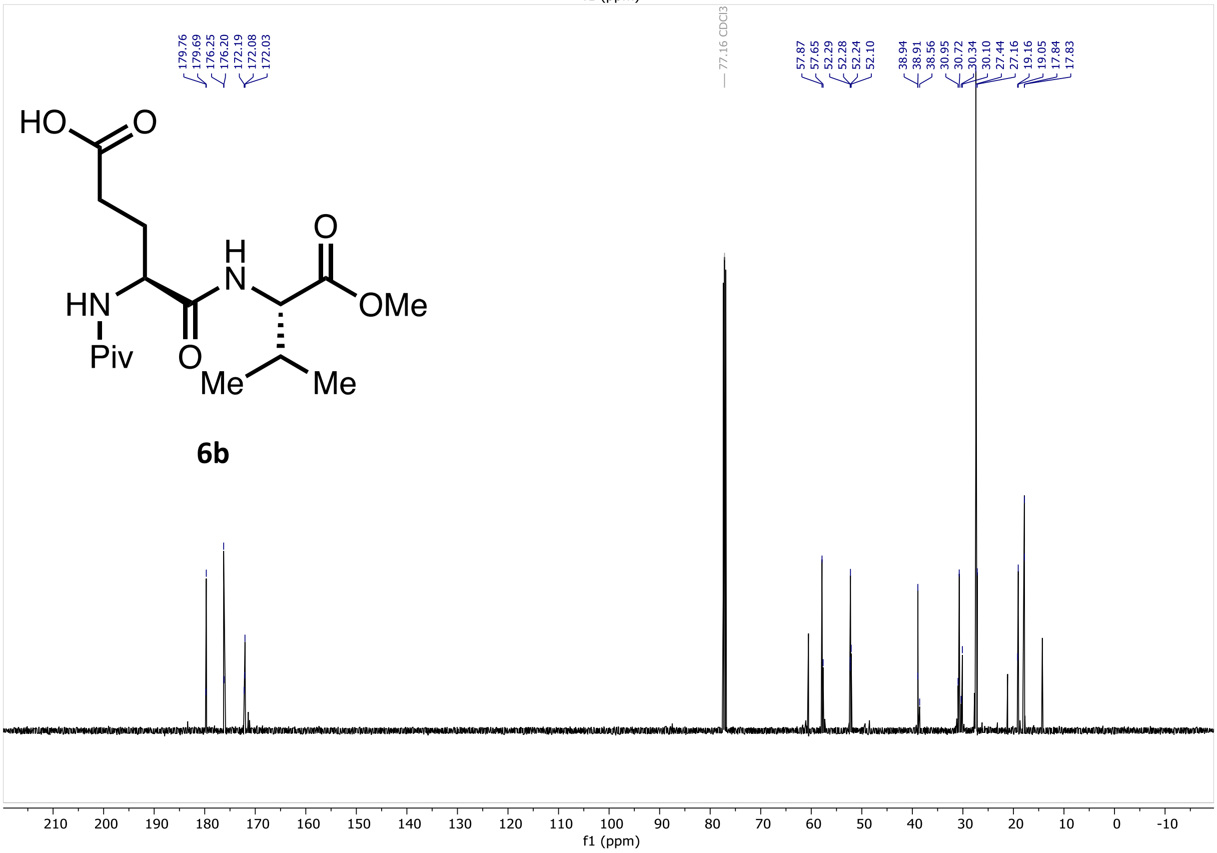

1,3-dioxoisoindolin-2-yl (*S*)-5-(((*S*)-1-methoxy-3-methyl-1-oxobutan-2-yl)amino)-5-oxo-4-pivalamidopentanoate

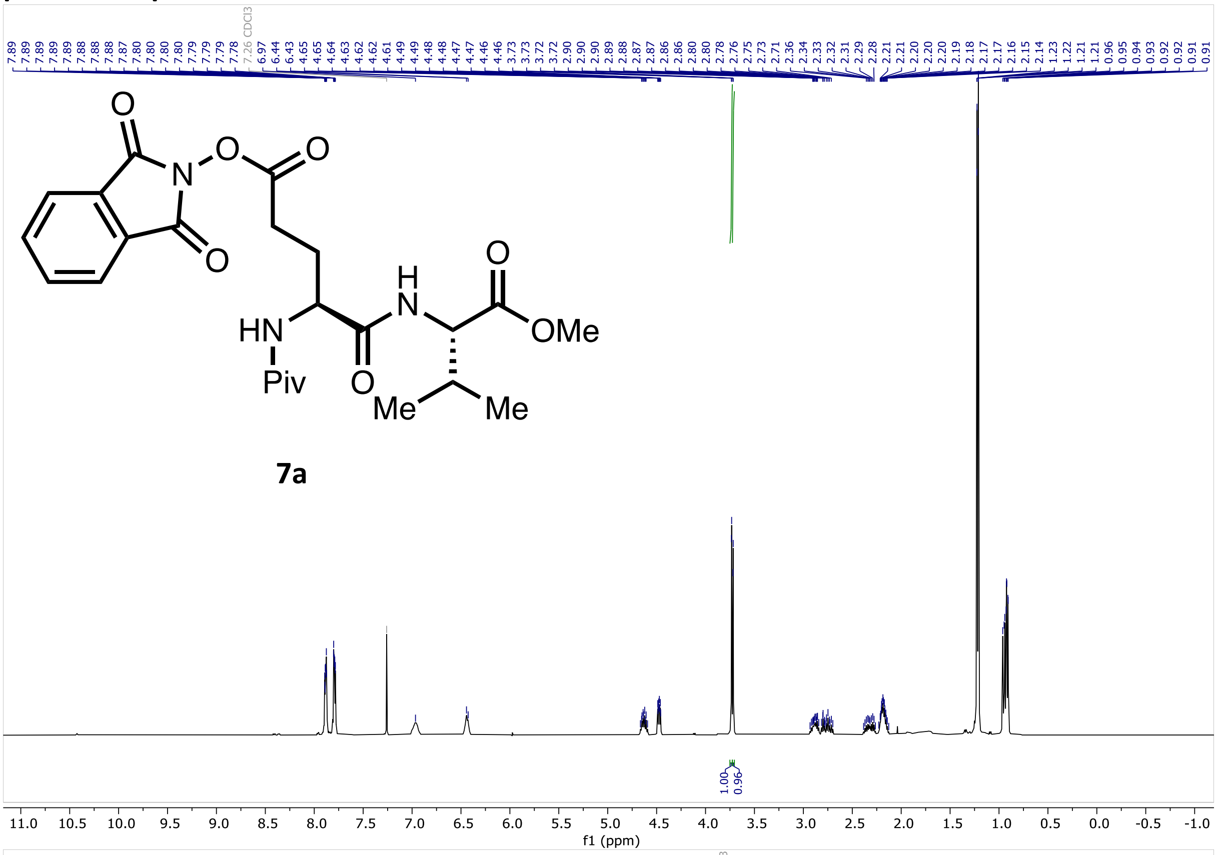

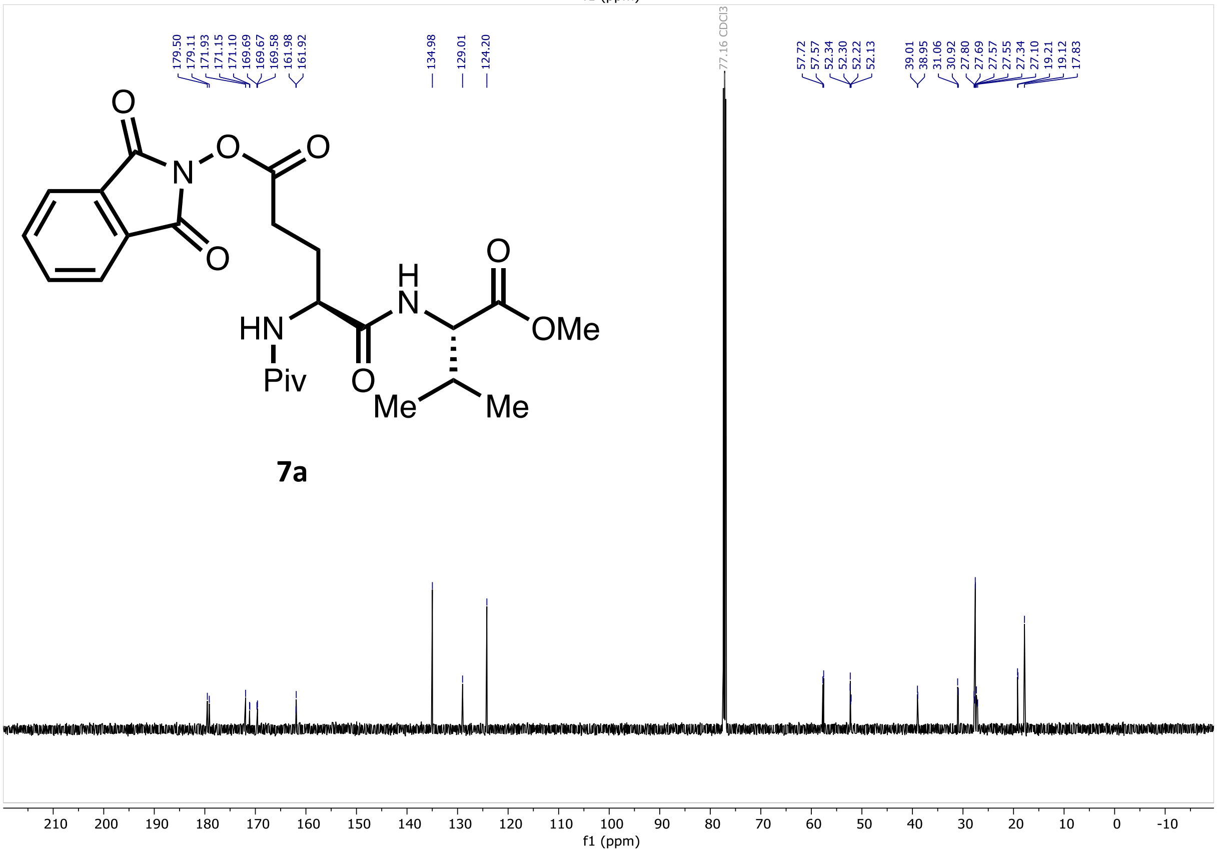

Dimethyl 2,2'-(((*S*)-2-pivalamidopentanedioyl)bis(azanediyl))(2*S*,2'*S*)-bis(3-methylbutanoate)

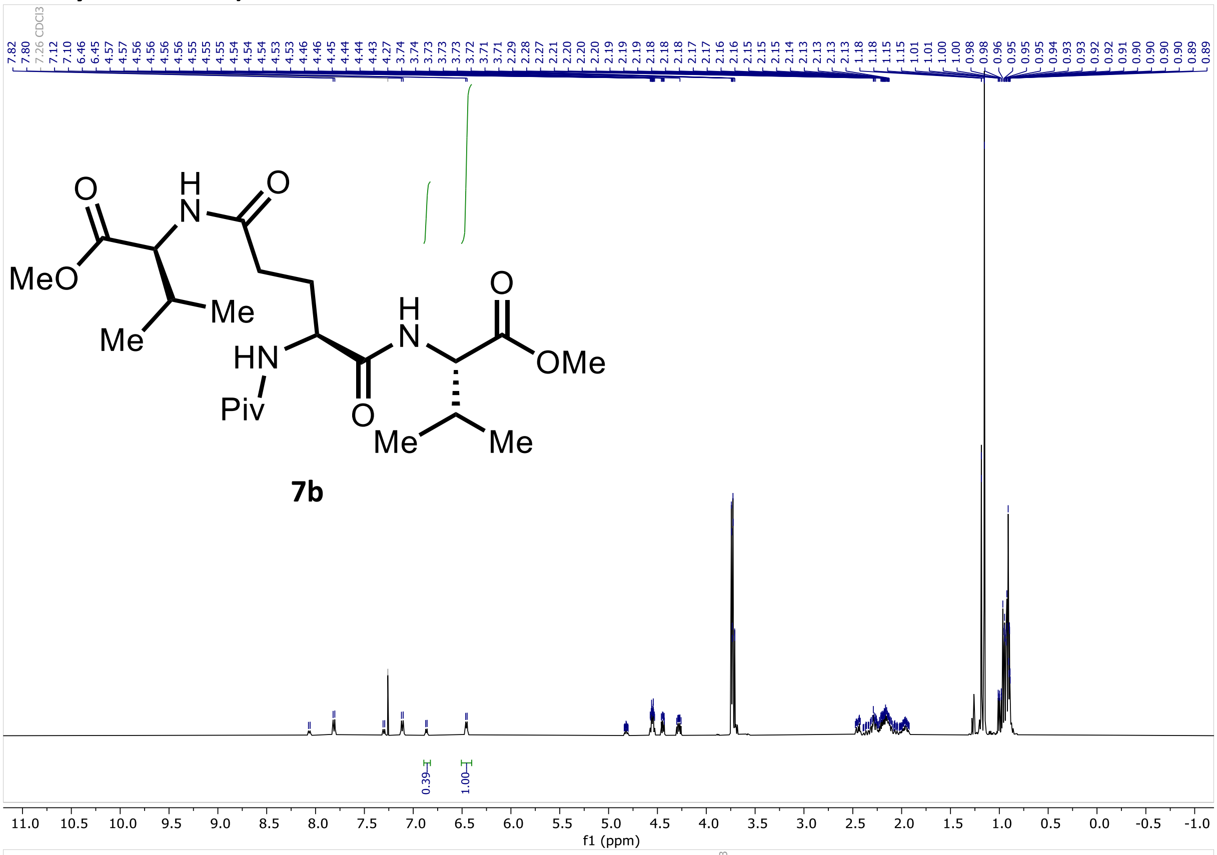

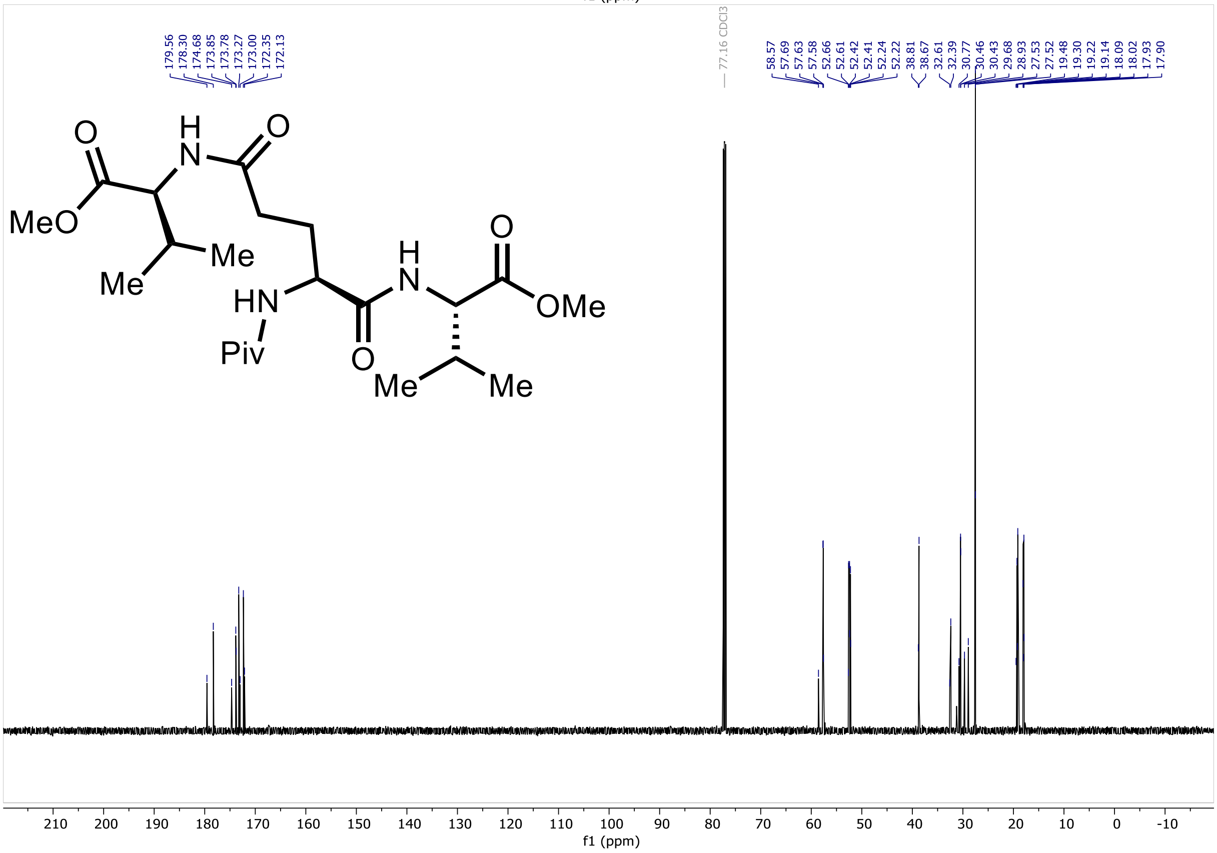

NMR Spectra – Minisci Products

N-(4-(4-(trifluoromethyl)pyridin-2-yl)butyl)pivalamide

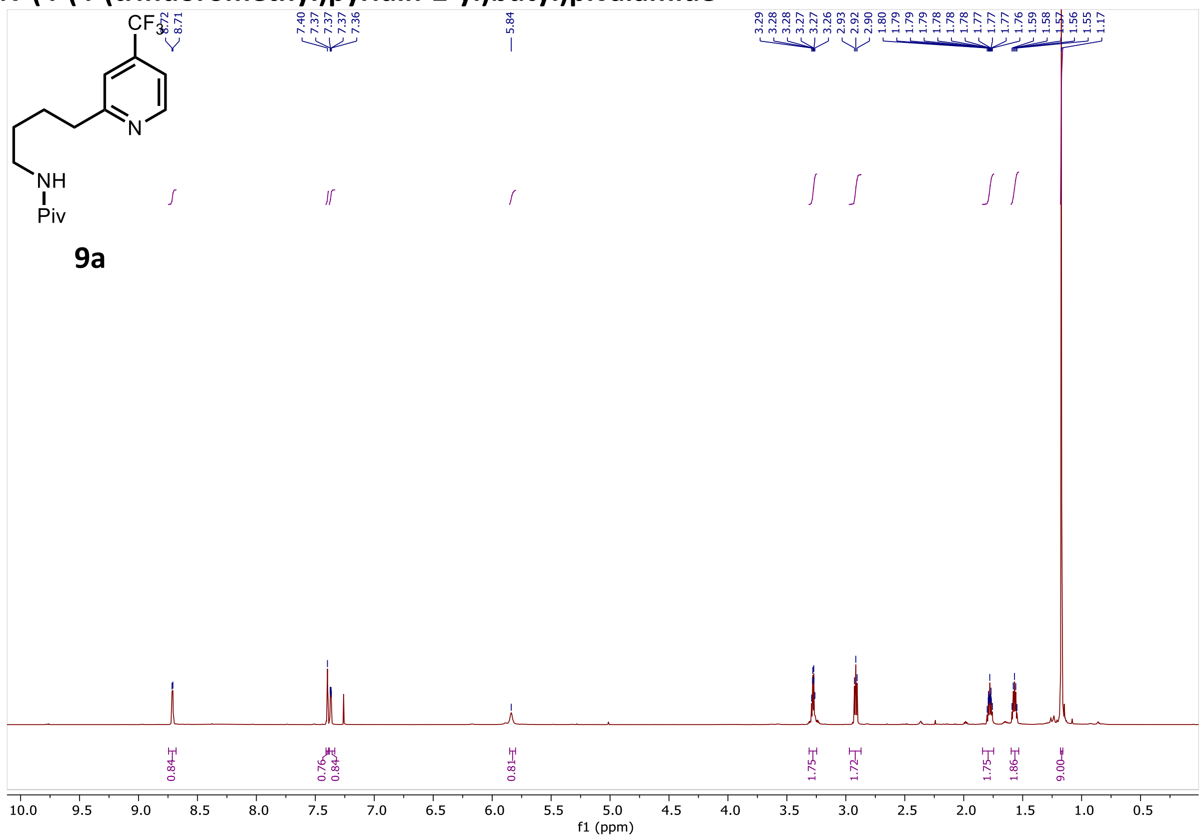

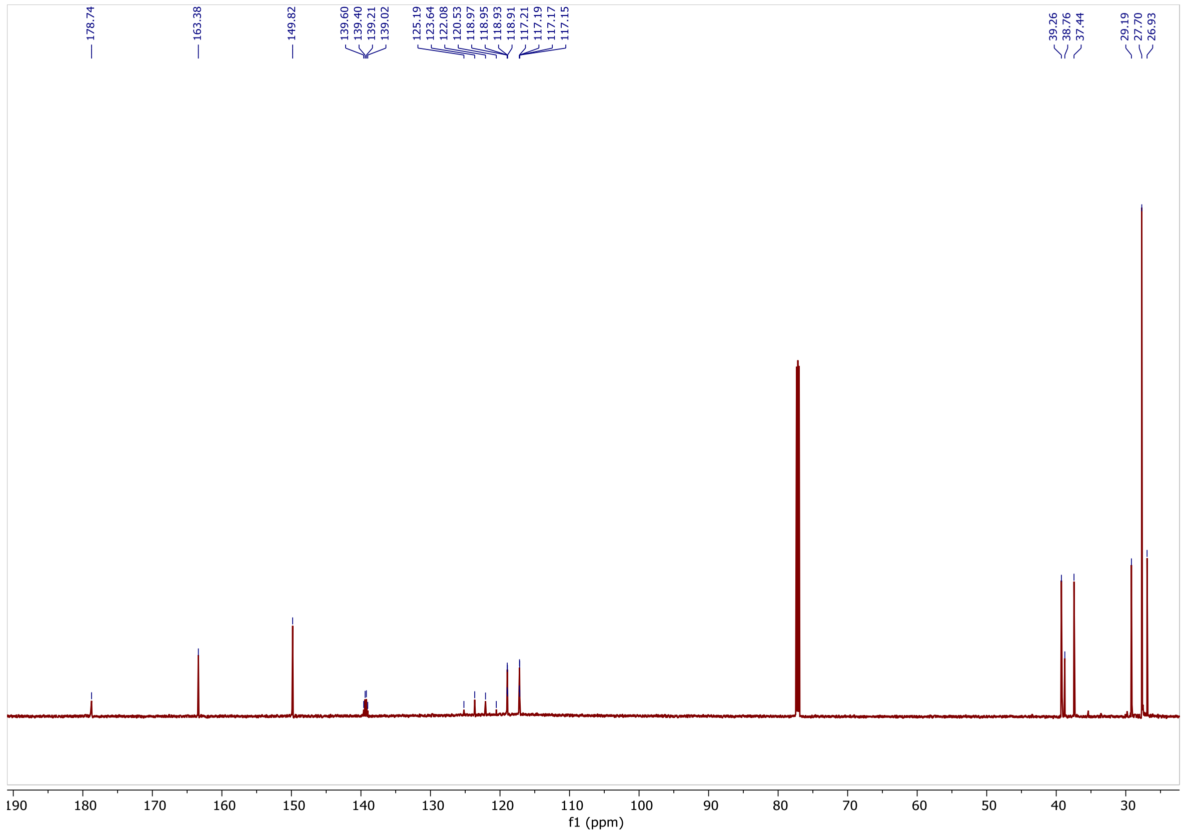

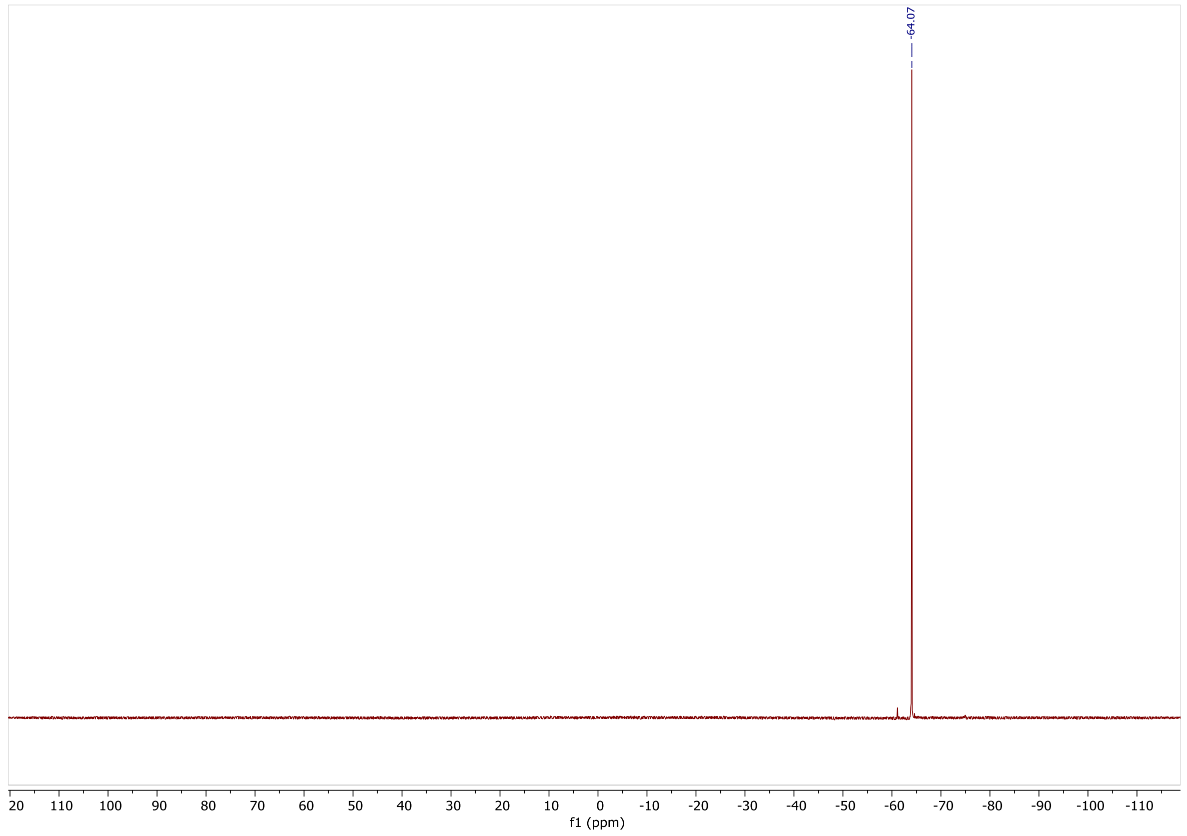
-64.07
20
110
100
90
80
70
60
50
40
30
20
10
0
-10
-20
-30
-40
-50
-60
-70
-80
-90
-100
-110
f1 (ppm)

N-(4-(4-methoxypyridin-2-yl)butyl)pivalamide

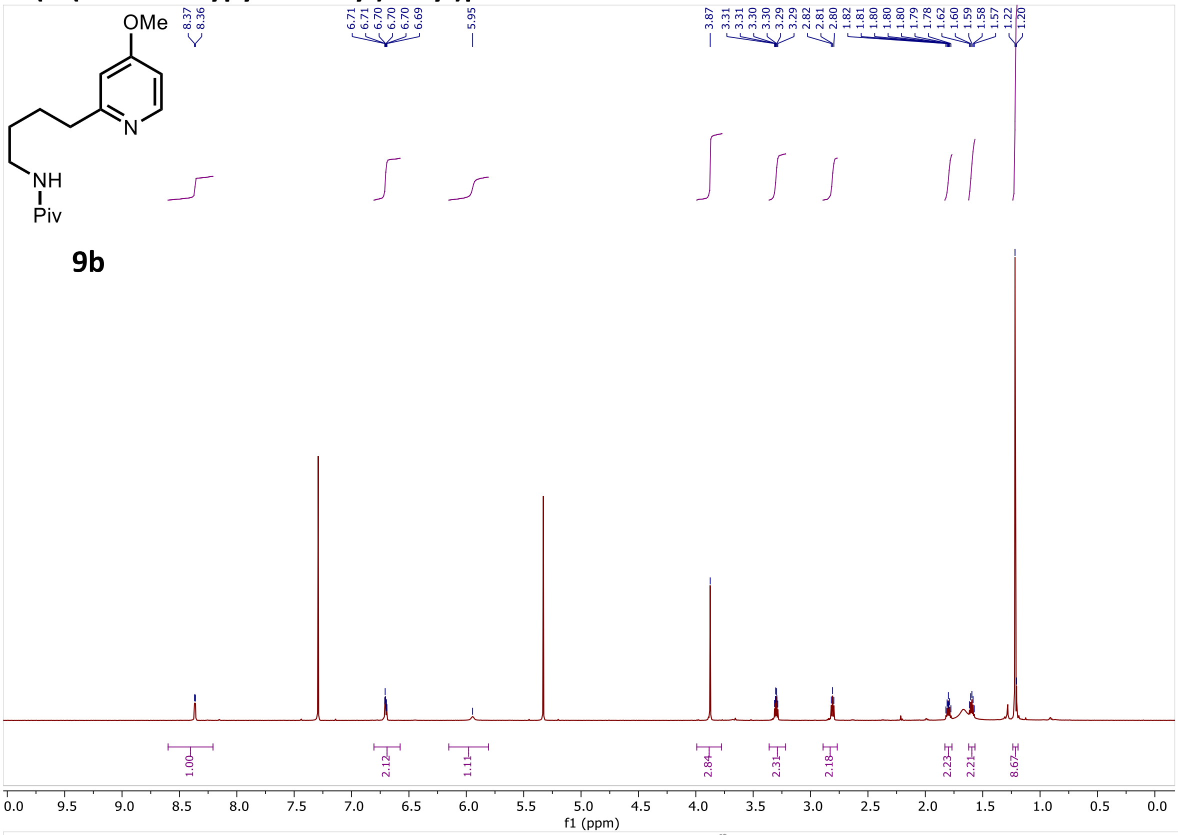

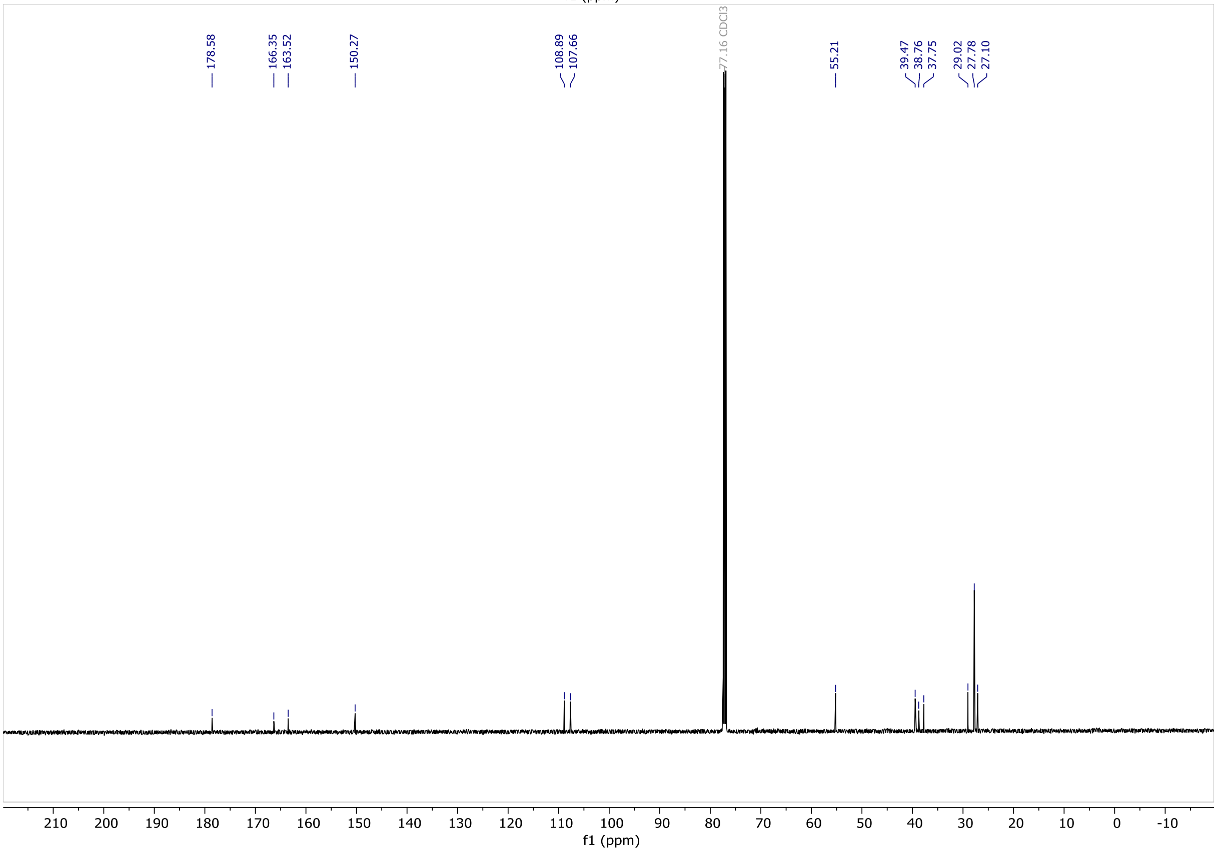

N-(4-(4-cyanopyridin-2-yl)butyl)pivalamide

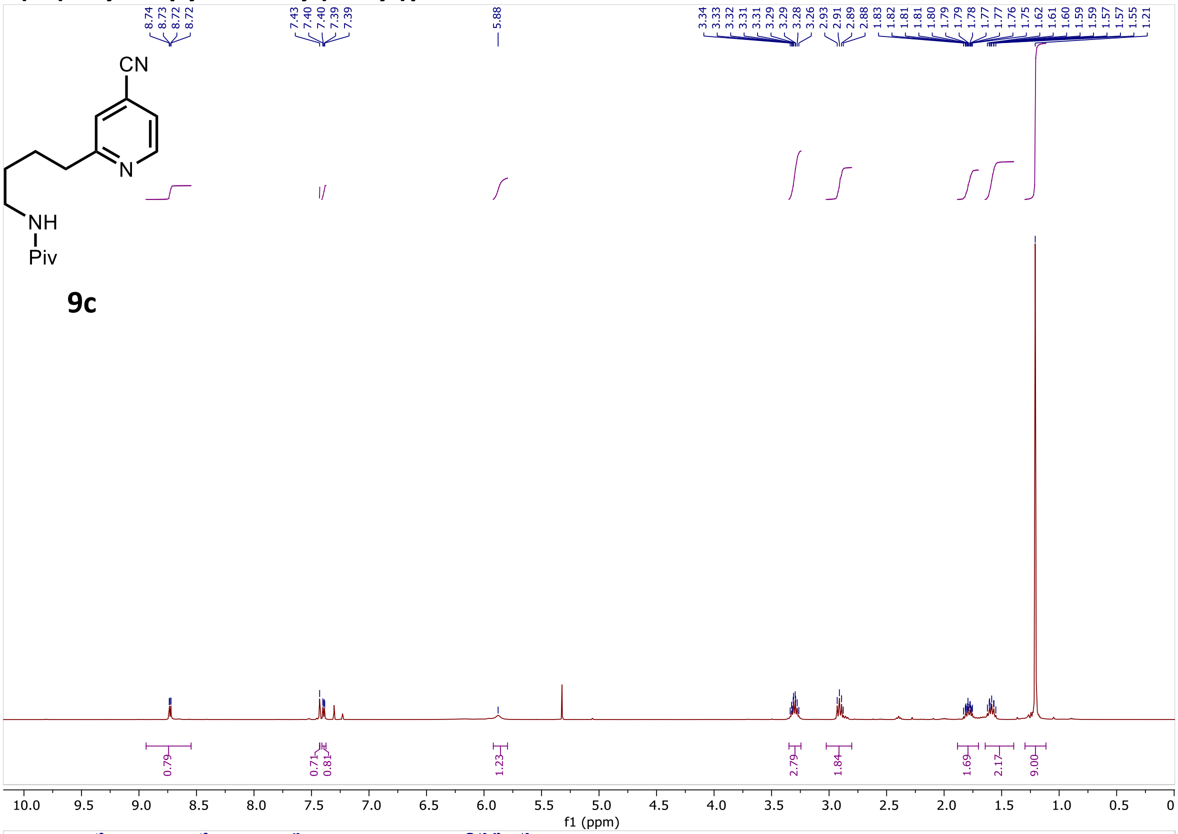

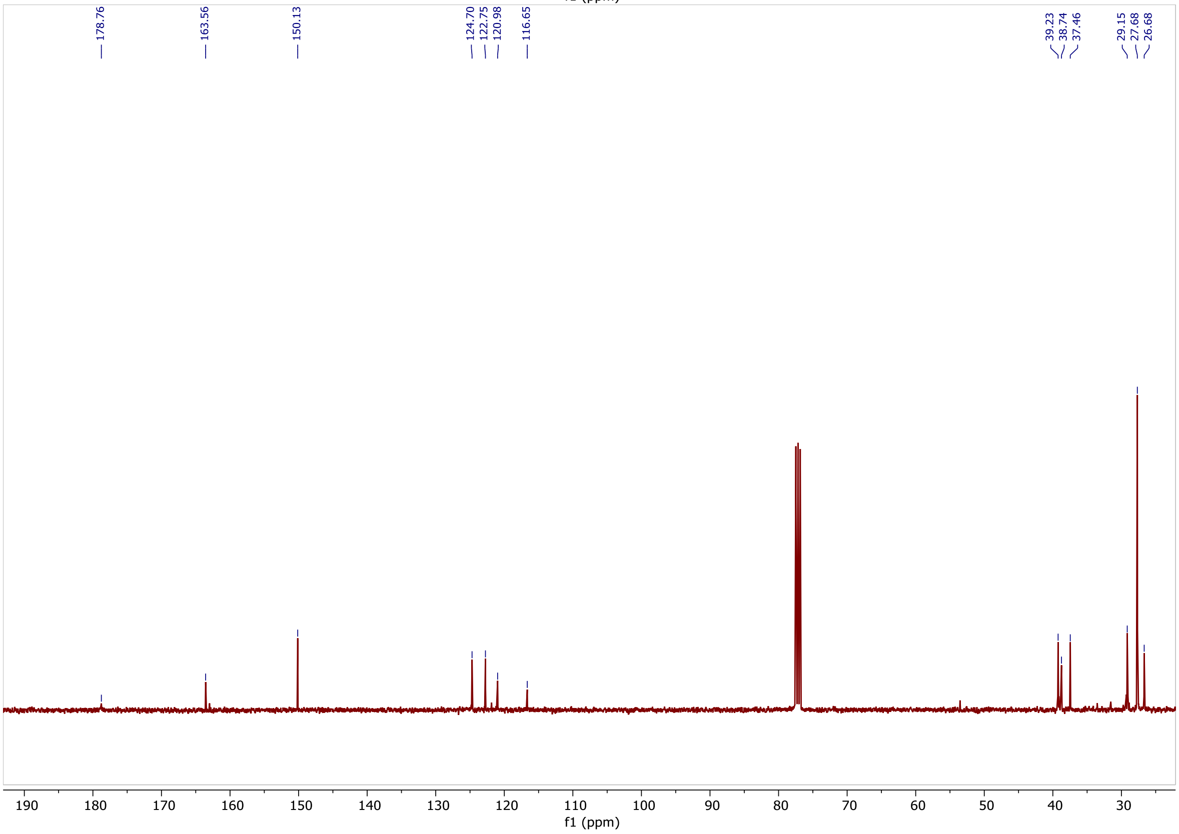

N-(3-(4-(trifluoromethyl)pyridin-2-yl)propyl)pivalamide

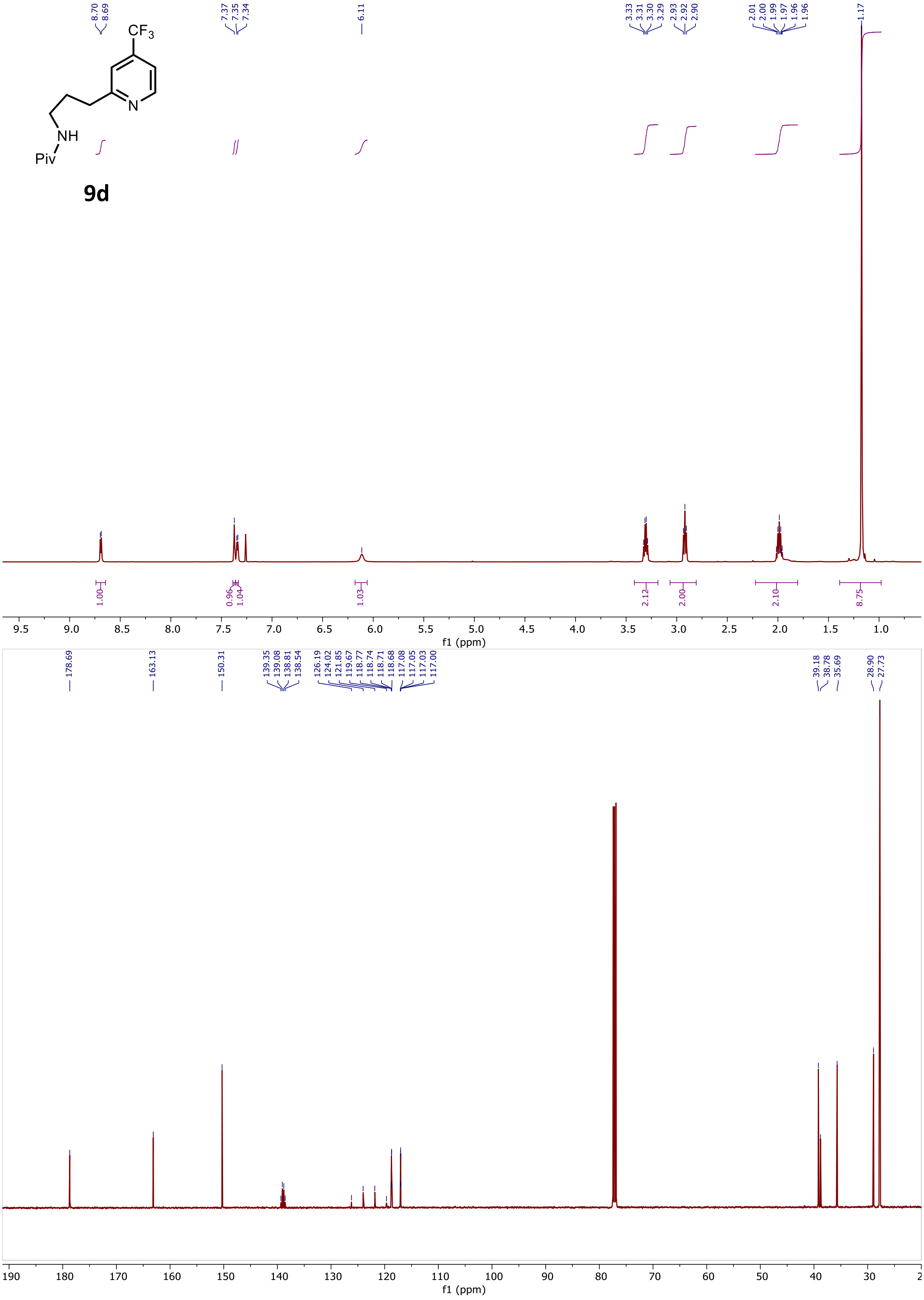

9d

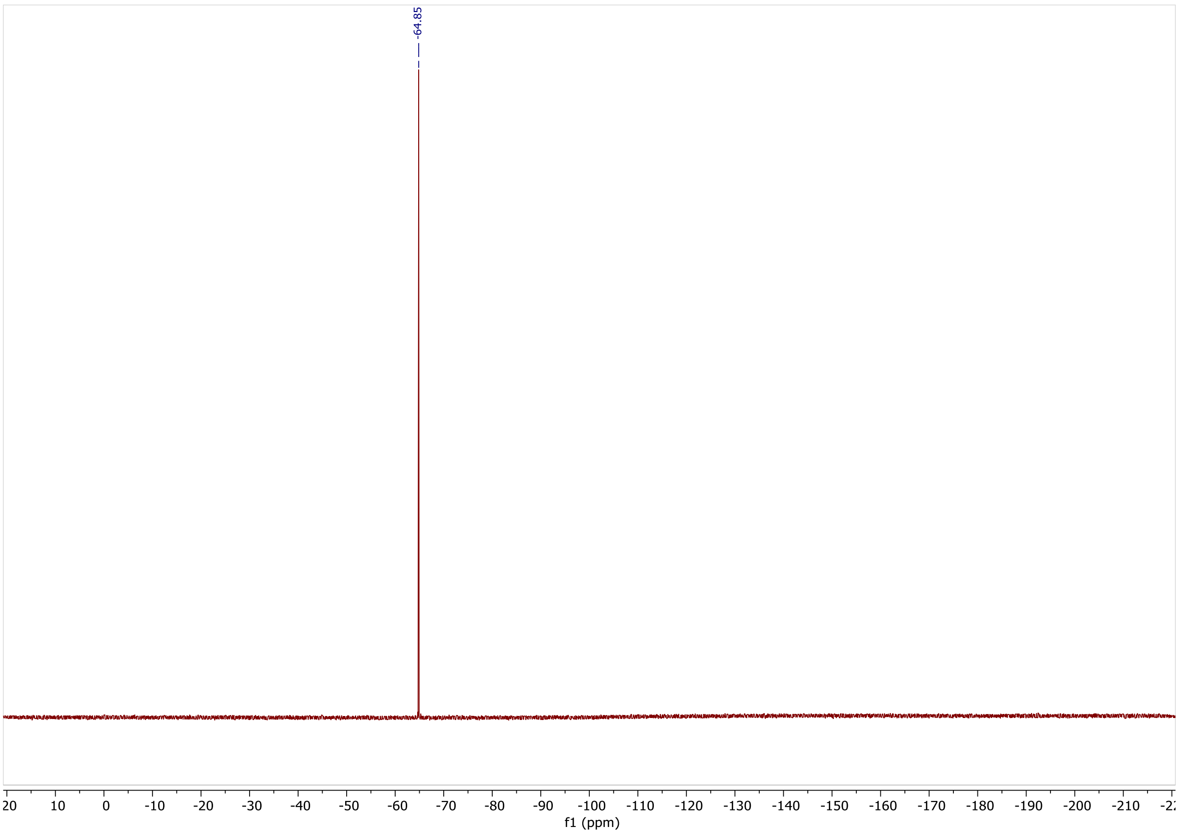
-64.85
20
10
0
-10
-20
-30
-40
-50
-60
-70
-80
-90
-100
-110
-120
-130
-140
-150
-160
-170
-180
-190
-200
-210
f1 (ppm)

***N*-(5-(4-(trifluoromethyl)pyridin-2-yl)pentyl)pivalamide**

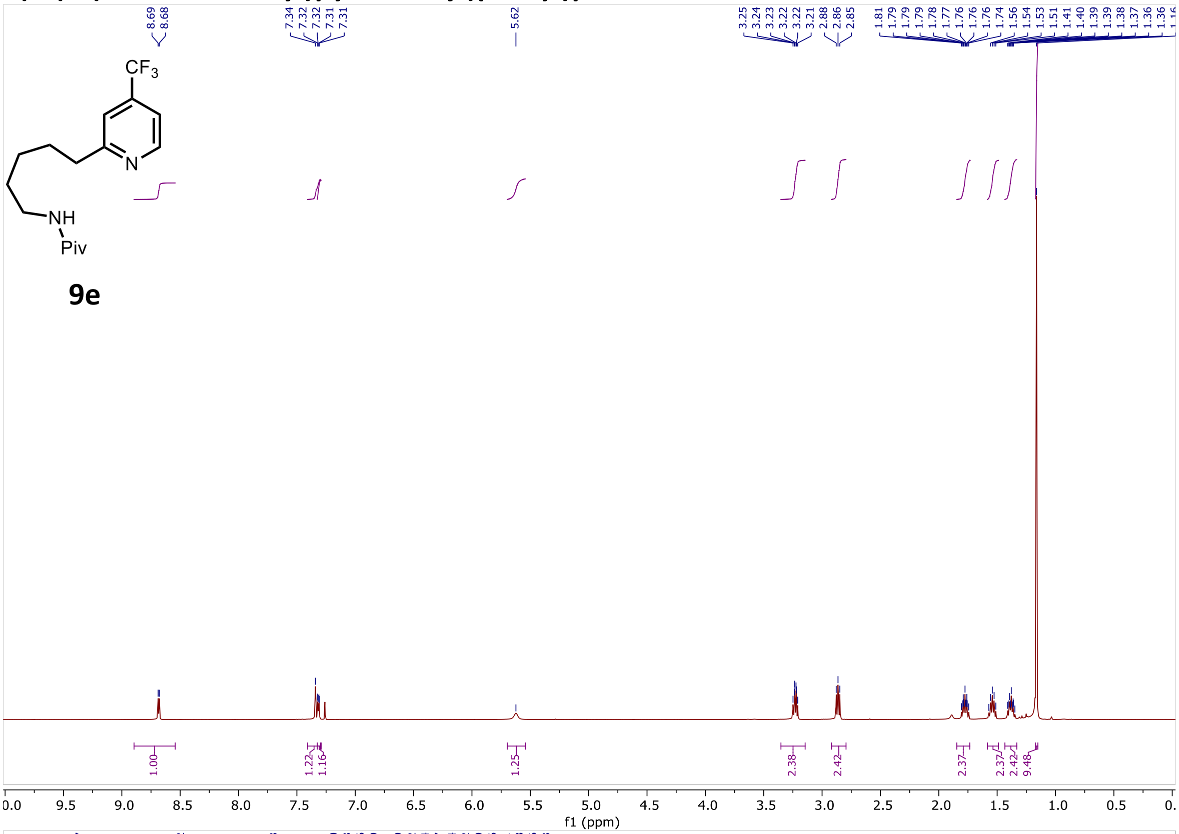

9e

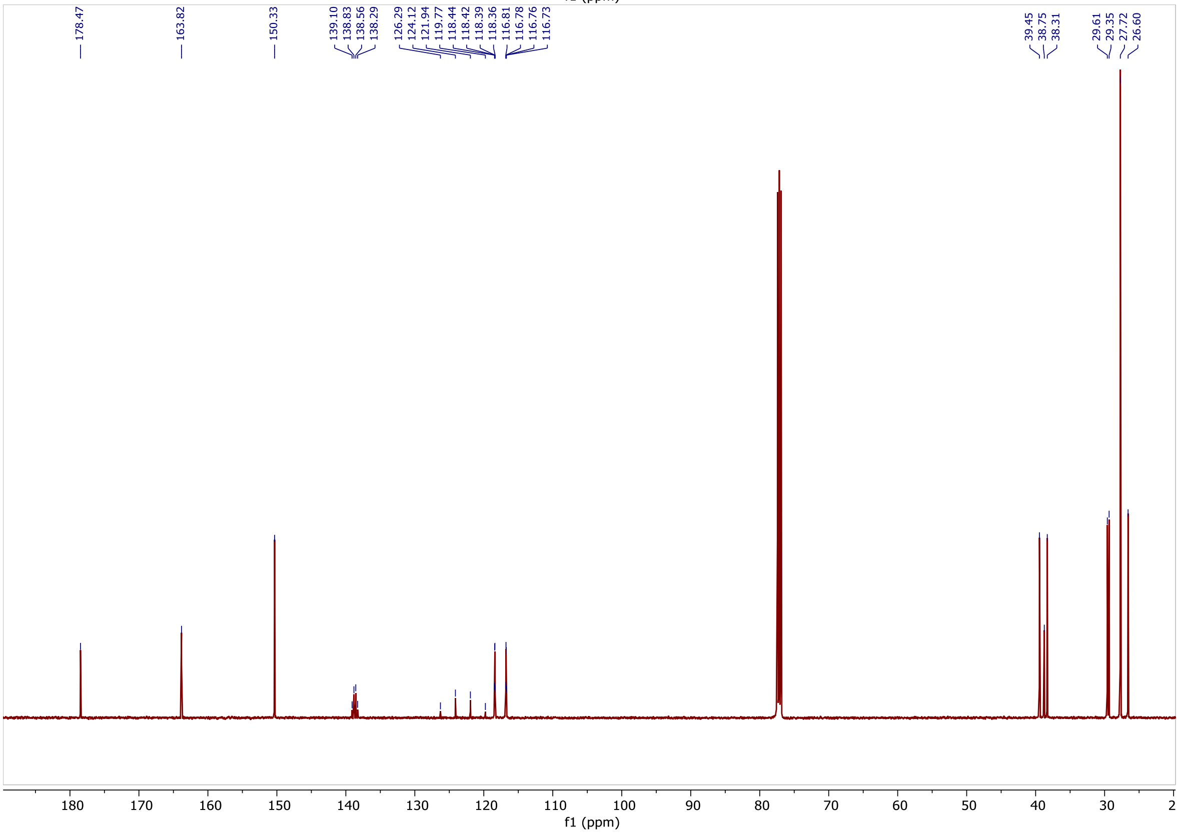

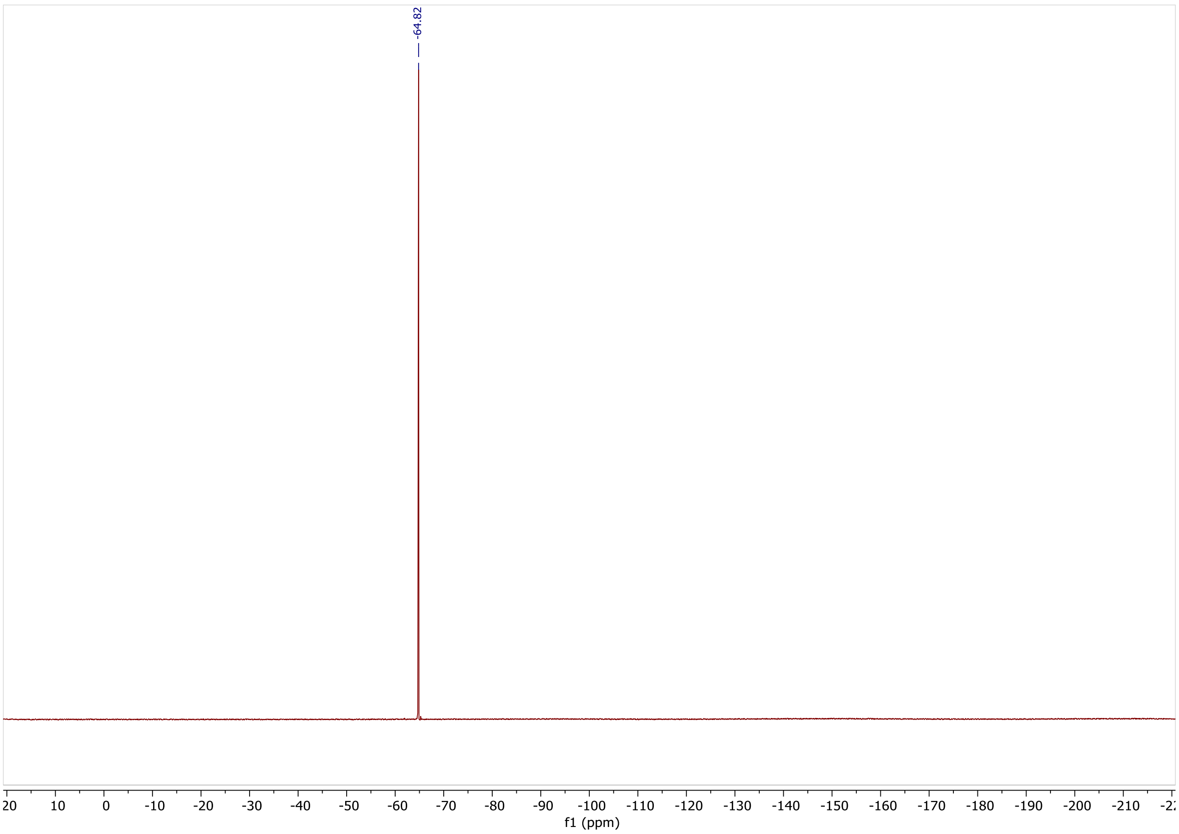
-64.82
20 10 0 -10 -20 -30 -40 -50 -60 -70 -80 -90 -100 -110 -120 -130 -140 -150 -160 -170 -180 -190 -200 -210
f1 (ppm)

N-(6-(4-(trifluoromethyl)pyridin-2-yl)hexyl)pivalamide

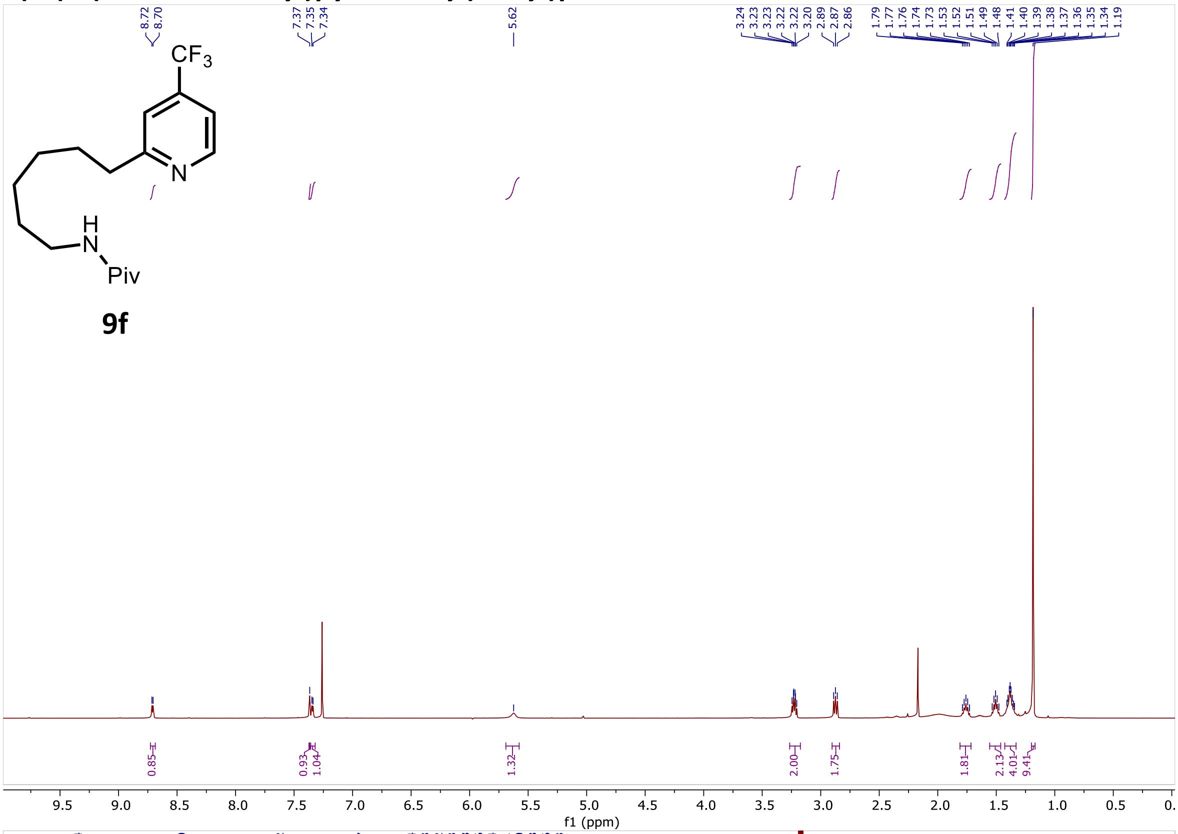

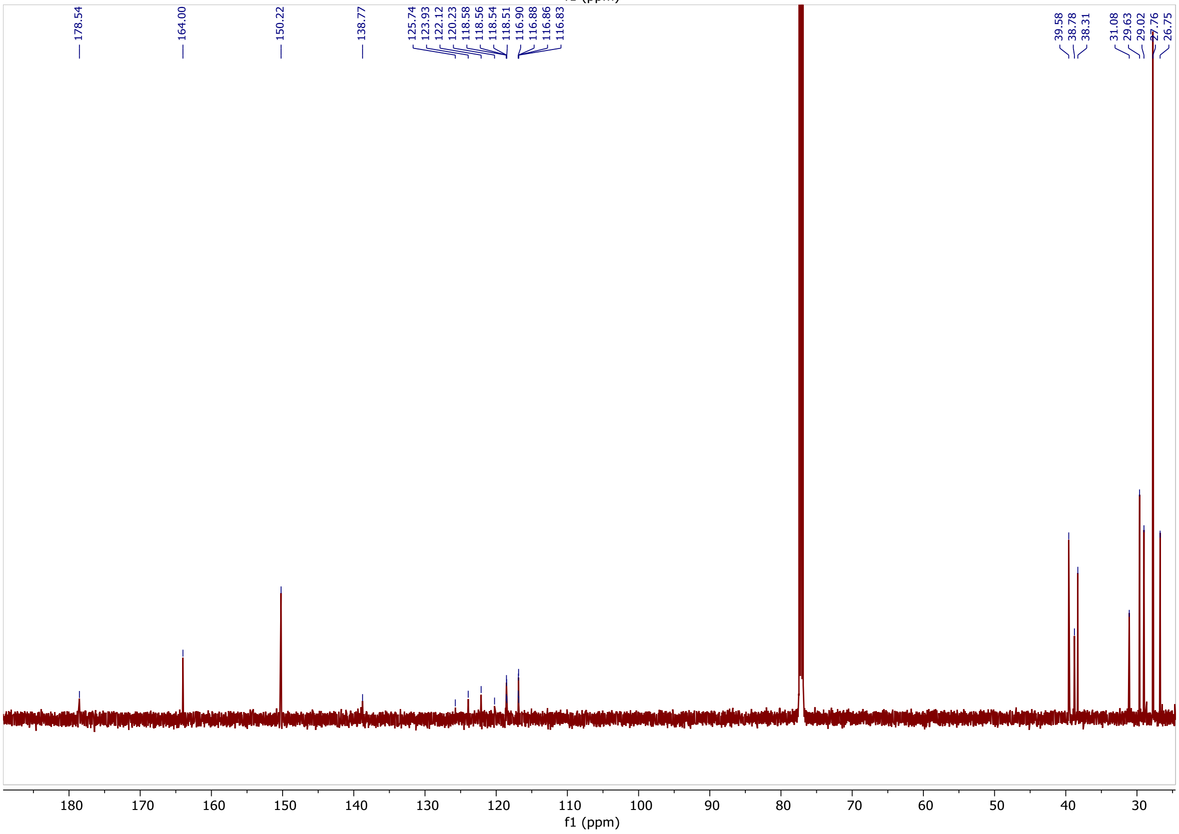

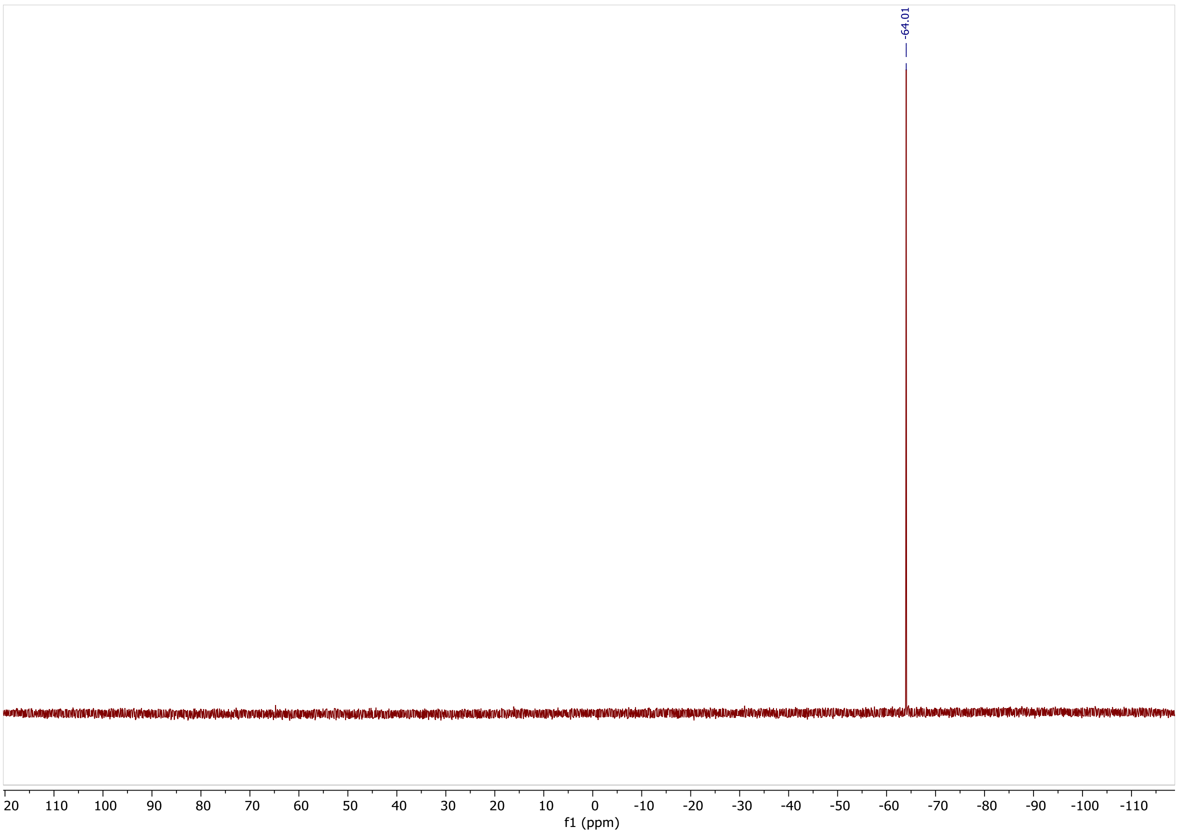
-64.01
20 110 100 90 80 70 60 50 40 30 20 10 0 -10 -20 -30 -40 -50 -60 -70 -80 -90 -100 -110
f1 (ppm)

***N*-(5-(4-(trifluoromethyl)pyridin-2-yl)pentan-2-yl)pivalamide**

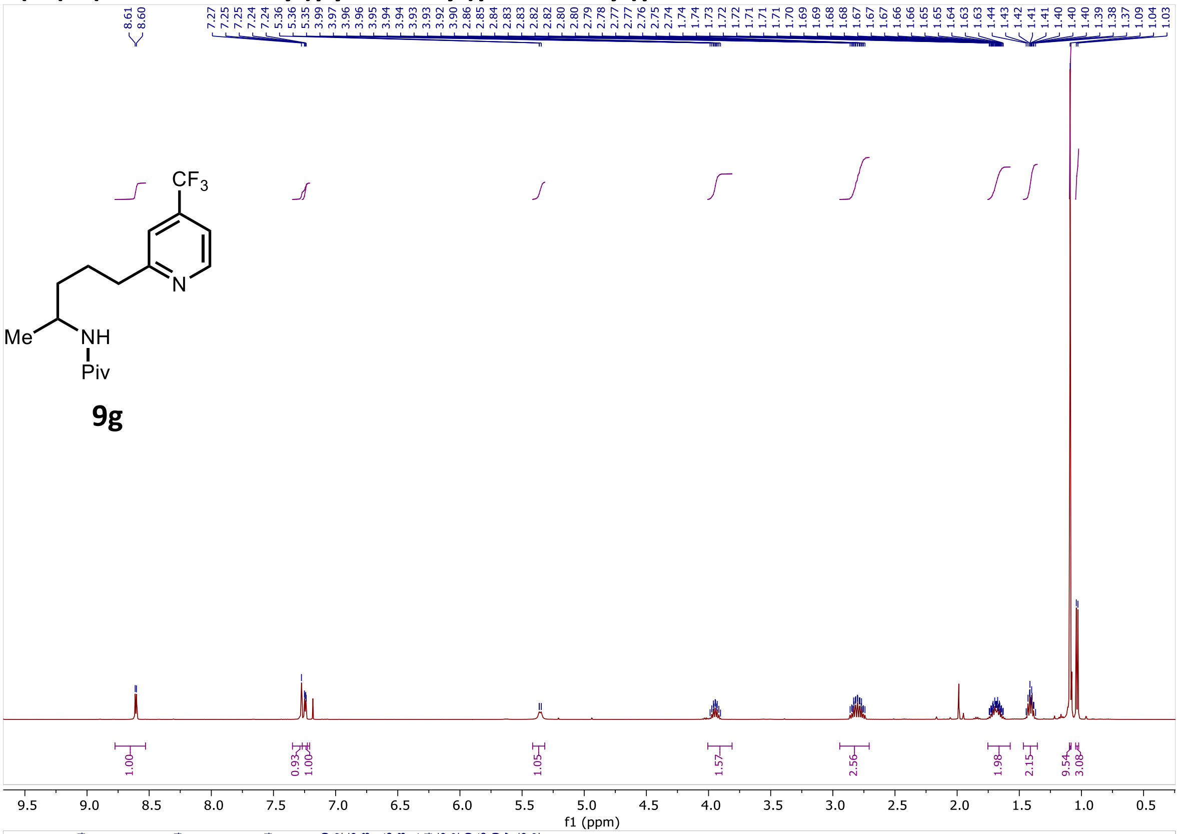

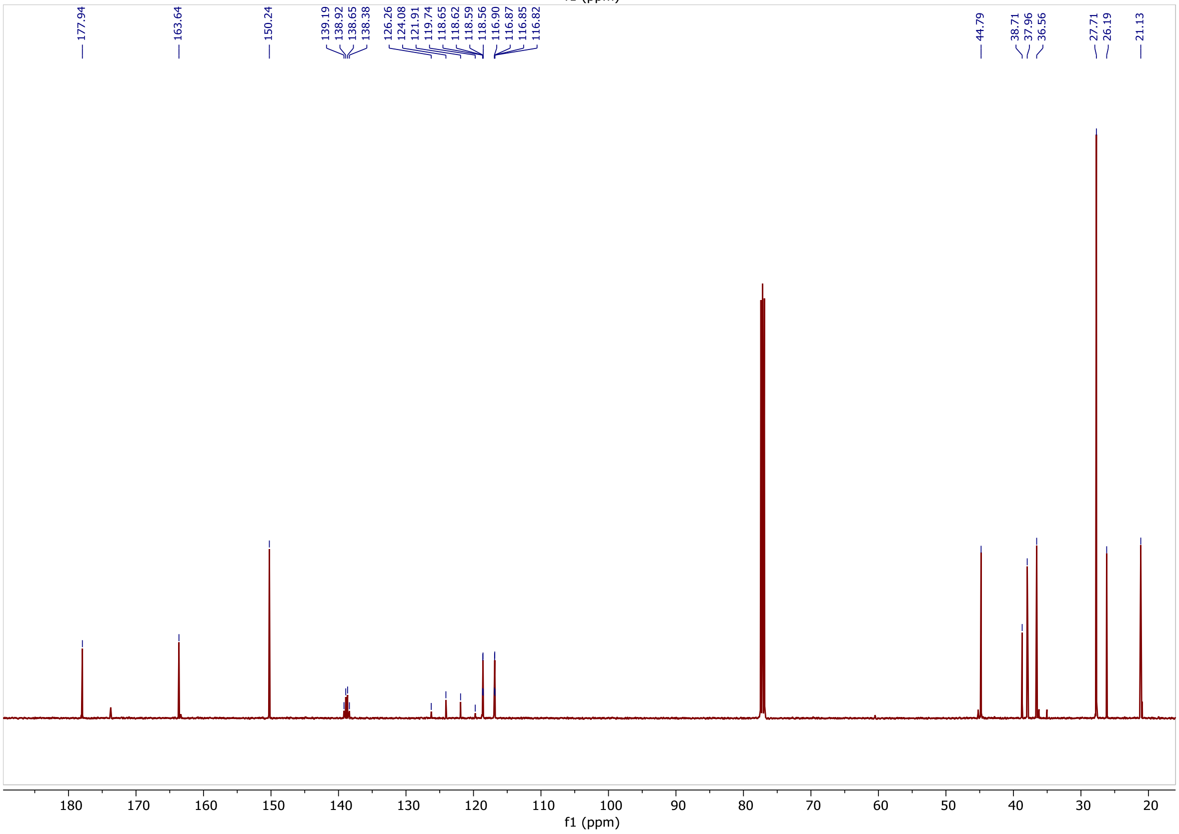

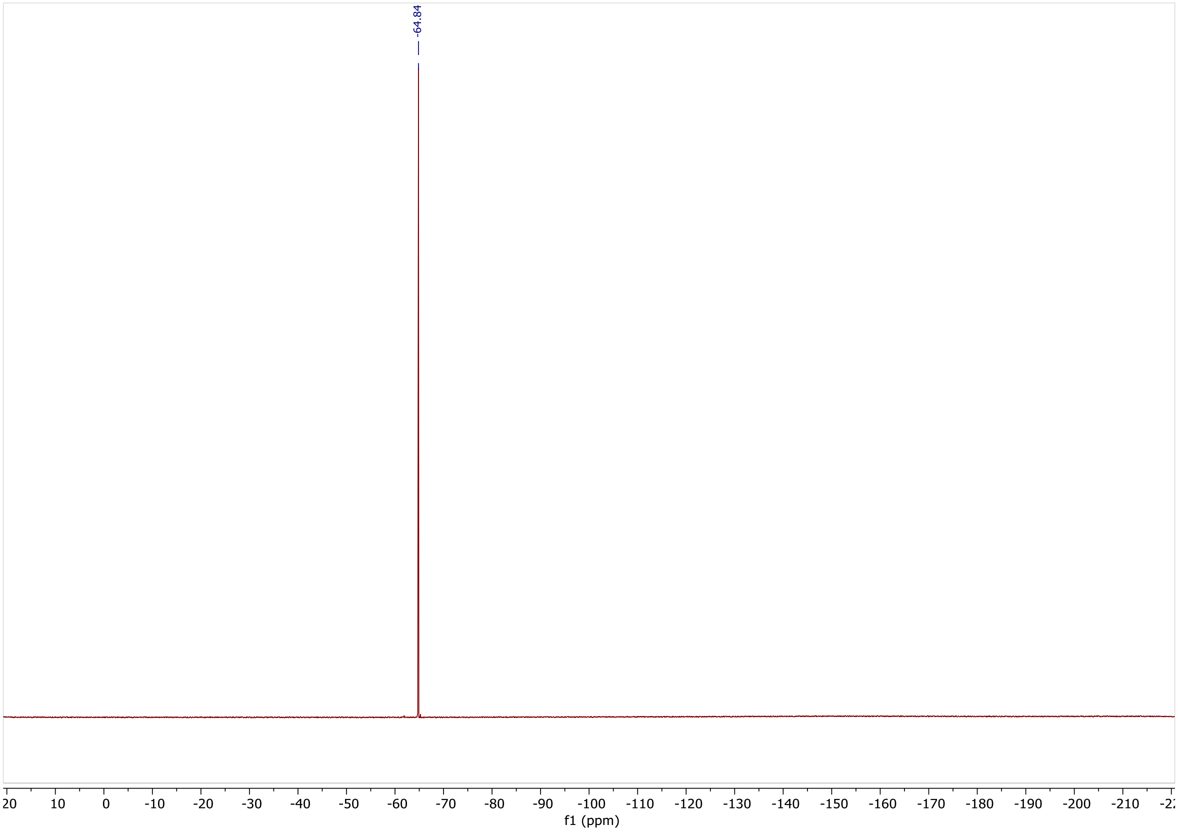
-64.84
20
10
0
-10
-20
-30
-40
-50
-60
-70
-80
-90
-100
-110
-120
-130
-140
-150
-160
-170
-180
-190
-200
-210
f1 (ppm)

NMR Spectra – Autocyclization Products

2-(*tert*-butyl)-5,6-dihydro-4*H*-1,3-oxazine

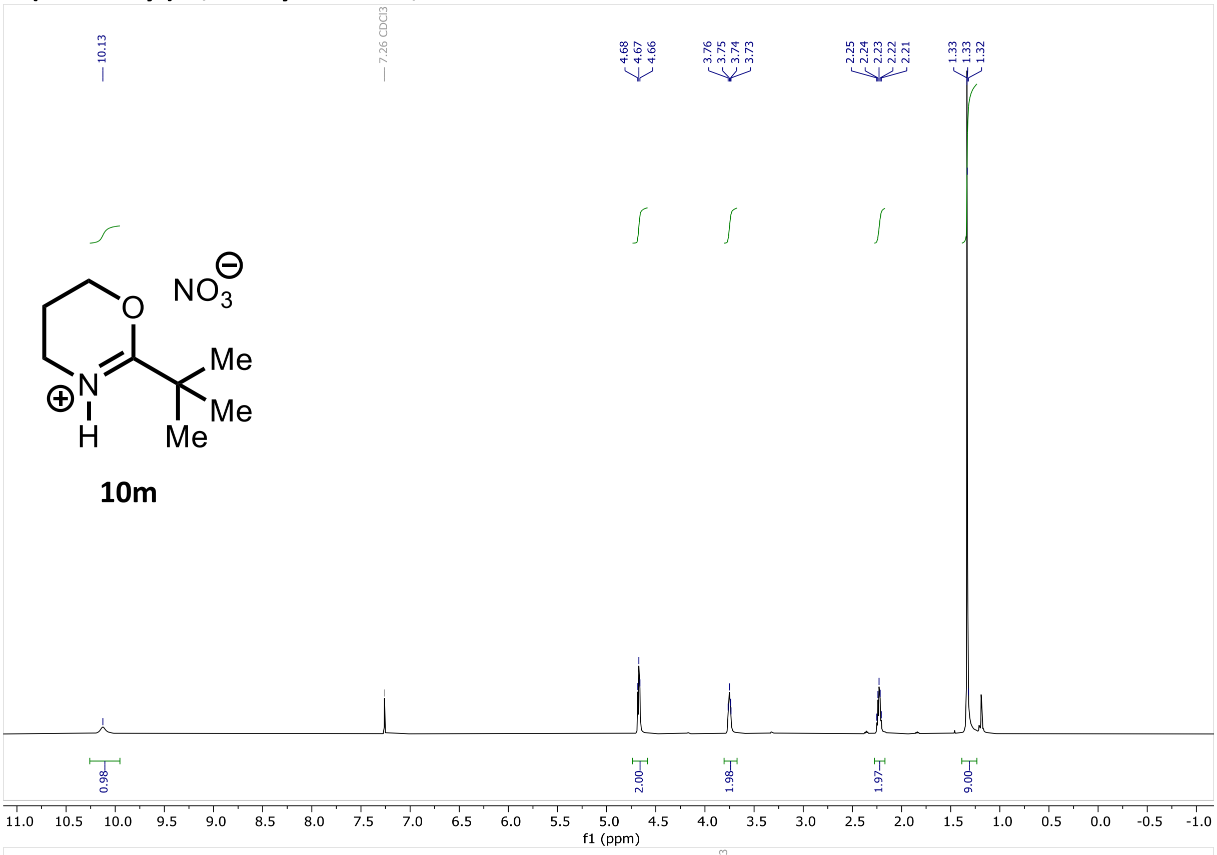

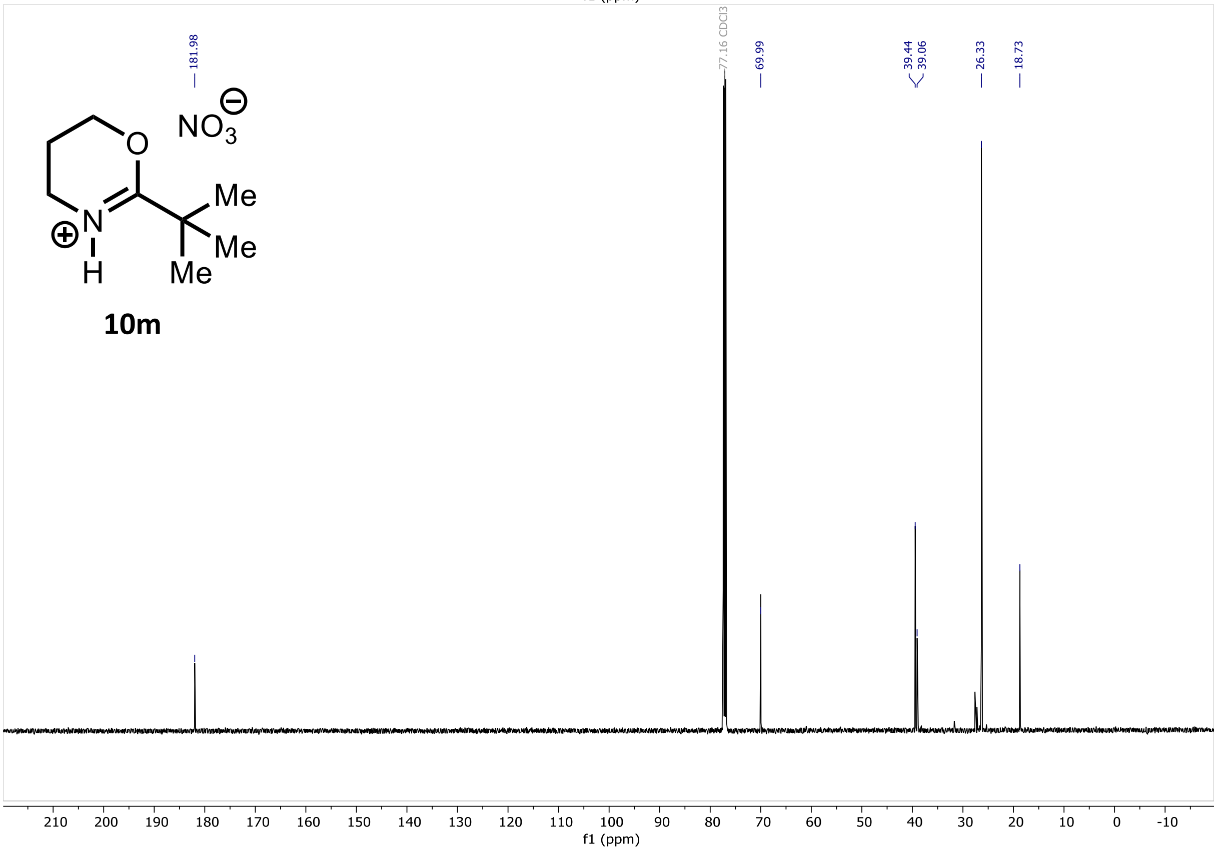

2-phenyl-5,6-dihydro-4*H*-1,3-oxazine

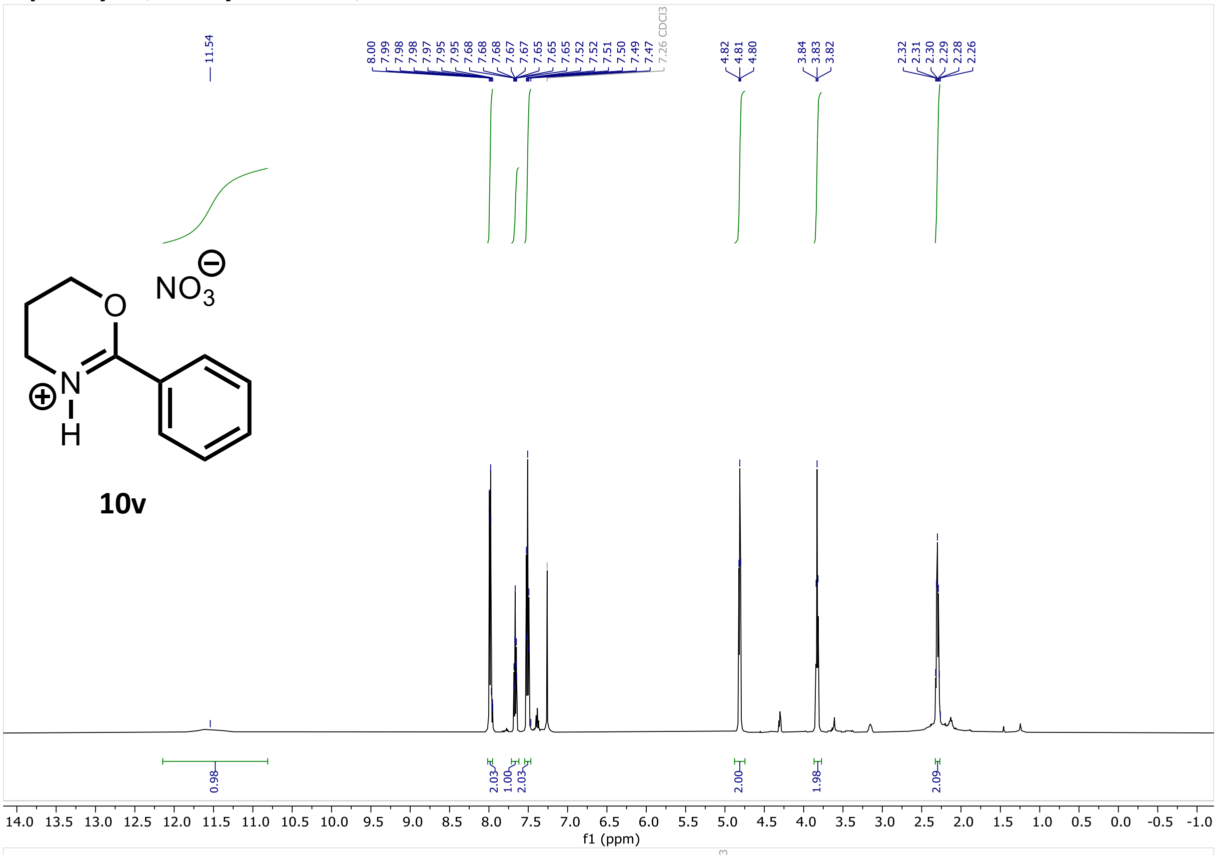

10v

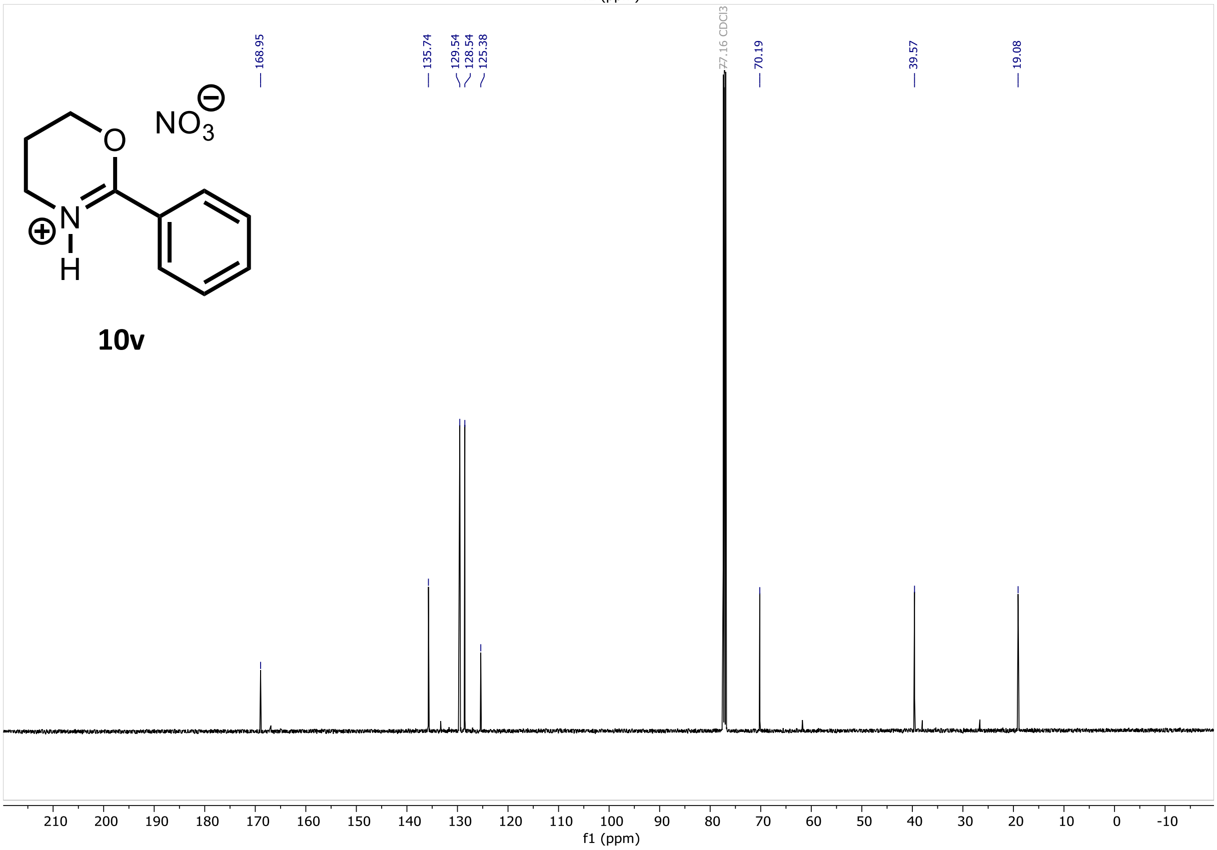

10v

6. Computational Coordinates and Vibrational Frequencies

Table S19. Cartesian coordinates (in Å) of all calculated structures for the riboflavin and copper(I) oxidation reactions.

Na_2SO_4
O -0.83724700 -2.60478100 -1.19689300
Na -4.09655100 -4.68065400 -0.44750400
S -2.02264500 -3.30178700 -1.84032300
O -2.02830200 -4.77595300 -1.47741600
O -3.32385800 -2.69881300 -1.34213100
O -1.90034800 -3.12796100 -3.34366900
Na 0.06257000 -1.92300200 -3.21157100

HSO_4_1min
O -1.56928000 -2.19594600 -0.93216000
S -2.09534900 -3.24272600 -2.12780700
O -2.09854800 -4.58211600 -1.49366100
O -3.43360800 -2.69149400 -2.42267400
O -1.10820900 -3.10375100 -3.22448000
H -0.68275500 -2.49111700 -0.66877800

SO_4_minus
O -1.67668000 -2.32464200 -0.91454800
S -2.02818900 -3.28715100 -2.06255000
O -2.20590400 -4.67250300 -1.57908200
O -3.37715400 -2.61451100 -2.37014800
O -1.08387400 -3.15906300 -3.19227200

$NaHSO_4$
O -0.84364300 -2.71477400 -1.16892100
S -1.93361200 -3.45523600 -1.86965300
O -2.07257200 -4.86392000 -1.46080900
O -3.34513700 -2.70345500 -1.44001300
O -1.89945700 -3.20572300 -3.33515400
Na -0.03018500 -1.71982800 -3.16793500
H -3.52334700 -2.91757700 -0.50889600

1a
N -1.48973100 1.18530800 0.57649100
C -0.50213800 1.37469200 -0.50409900
C -0.42329800 2.84673300 -0.93089900
C -1.82708600 3.45952300 -0.95637200
C -2.42978800 3.47251000 0.46687200
C -1.77629300 2.39468800 1.34908000
H 0.47962400 0.99882600 -0.18708000
H 0.21996300 3.41698200 -0.25056400
H -1.80591100 4.47012400 -1.37486400
H -2.27317800 4.44497900 0.94634000
H -2.39994600 2.16309800 2.20372100
H -3.51114000 3.30834800 0.41857500
H -0.82709100 2.76720400 1.75332600
H -2.45830300 2.85608400 -1.61956500
H 0.04794500 2.89425600 -1.91746500
H -0.82178200 0.75634200 -1.34577200
C -1.95376700 -0.08535900 0.76395100
O -1.49696500 -1.00064400 0.06410400
C -3.04754500 -0.40437800 1.81883600
C -4.31900700 0.44757100 1.61257100
H -5.11084200 0.07763400 2.27190100
H -4.18403000 1.50658100 1.83274500
H -4.67571400 0.36217800 0.58118100
C -3.44869800 -1.87991800 1.63016100
H -2.59087300 -2.54288600 1.75684200
H -4.20983300 -2.14429500 2.37101300
H -3.86050800 -2.05305500 0.63275000
C -2.48566500 -0.25634800 3.25057000
H -1.60178100 -0.88904900 3.37993700
H -2.20378000 0.76591500 3.50835300
H -3.24105000 -0.58240400 3.97294300

1.A
N -1.68274700 1.30592400 0.51590600
C -1.29523400 1.41346600 -0.71435500
C -1.10977000 2.69185200 -1.43211000
C -1.16029700 3.92986800 -0.53429800
C -2.22823400 3.73919000 0.54275100
C -1.93726100 2.49881000 1.37731500
H -0.17319800 2.60954300 -1.99667600
H -0.18289100 4.08017800 -0.06483100
H -2.26292800 4.59497400 1.22112300
H -2.77056900 2.26244300 2.03018800
H -3.21835900 3.64381400 0.08392100
H -1.04347800 2.61971500 1.99584200
H -1.37040700 4.81354700 -1.13982300
H -1.89728900 2.70733900 -2.20212800
H -1.10984100 0.48012000 -1.23902600
C -1.75415900 -0.10105200 1.06809200
O -0.84080000 -0.81771100 0.76562500
C -2.98320400 -0.50919500 1.87242300
C -4.27047200 0.09378800 1.27429800
H -5.12491200 -0.30871600 1.82412900
H -4.32177600 1.18115100 1.34739000
H -4.38350000 -0.18500800 0.22307200
C -3.06290000 -2.04783000 1.79226000
H -2.16960500 -2.51333300 2.21223200
H -3.93301000 -2.38325000 2.36166700
H -3.17479800 -2.38379800 0.75819700
C -2.79842300 -0.11179300 3.35964100
H -1.87121700 -0.52984500 3.75979700
H -2.79116400 0.96567800 3.52835200
H -3.63539000 -0.52958000 3.92533500

1a_OH
N -1.43566600 1.18260000 0.61171800
C -0.44309000 1.38299000 -0.43627200
C -0.48712000 2.82941900 -0.95774700
C -1.92201500 3.36421200 -0.93393200
C -2.42296000 3.44867200 0.52266500
C -1.71673300 2.39061800 1.39206200
H 0.15920500 3.45176200 -0.32765800

H -1.98172600 4.34085800 -1.42234700
H -2.21300000 4.43672700 0.94640300
H -2.29544400 2.15354700 2.27627000
H -3.50733000 3.30575800 0.55727500
H -0.75467800 2.77528600 1.74668700
H -2.56229900 2.68410700 -1.50774400
H -0.06703600 2.85112400 -1.96808900
H -0.69153400 0.68408700 -1.23933200
C -1.97553200 -0.07657400 0.73729900
O -1.58566300 -0.98149900 -0.00709100
O 0.83368300 1.05735700 0.11607400
H 1.49422300 1.22650200 -0.57068700
C -3.06505400 -0.38230100 1.79918500
C -4.30131800 0.53151000 1.65089900
H -5.10474300 0.15118500 2.28957200
H -4.12402000 1.56714300 1.93946100
H -4.66475000 0.52594600 0.61836900
C -2.46706900 -0.30933000 3.22202600
H -2.12506300 0.68781900 3.50378500
H -3.22524300 -0.61661800 3.94942800
H -1.61669600 -0.99236000 3.31344100
C -3.53521900 -1.83106200 1.56700800
H -2.70531800 -2.53529500 1.64832000
H -4.28943400 -2.08867100 2.31698600
H -3.97852200 -1.94869300 0.57502400

$Na_2S_2O_8$

S 1.62960000 2.08771400 -5.89558900
O 0.70032800 3.10867100 -5.35698100
O 0.99701800 1.19925700 -6.89813400
O 2.98644100 2.54504200 -6.21053600
S 0.74807400 -0.96887500 -3.54144200
O 0.92503700 -1.68173200 -4.82869500
O -0.60581200 -1.02156300 -2.97171900
O 1.87546000 -1.11171200 -2.61362200
Na 0.99865200 -1.05055800 -6.98683900
Na -0.53182400 2.63685500 -3.44496800
O 0.71857600 0.70894400 -3.95828500
O 1.99911100 1.09863500 -4.54952800

$K_2S_2O_8$

S 1.64186700 2.07693400 -5.78680500
O 1.48475300 3.36651800 -5.09729700
O 0.35345100 1.45584300 -6.19332100
O 2.72063300 1.96051700 -6.78334300
S 0.78866500 -0.81056400 -3.45713200
O 0.75815500 -1.33886600 -4.83427100
O -0.53809100 -0.54550700 -2.85607300
O 1.78575100 -1.43231100 -2.57526600
K 1.30854200 -0.87143400 -7.37190400
K -1.42993200 1.83622800 -4.10374900
O 1.27506600 0.85089300 -3.58518500
O 2.29180000 1.00242800 -4.61199200

(2.A.m), SINGLET

C -10.49493900 -0.18817900 -0.06030800
C -9.84257500 -0.75029700 1.01751500
C -8.71001000 -0.14349700 1.60353200
C -8.21474500 1.07891900 1.06822100
C -8.88376900 1.65262100 -0.02571700
C -10.00036800 1.04334200 -0.58563600
N -8.13298200 -0.74466000 2.68157400
N -7.10245100 1.65099800 1.66874700
C -6.52211900 1.08036100 2.77675400
C -7.10175100 -0.16840600 3.23345200
C -6.47482900 -0.81446600 4.42076800
O -6.85001400 -1.87087300 4.91094400
N -5.41454000 -0.09456600 4.92288900
C -4.90299300 1.12765600 4.43558000
N -5.50368200 1.69430600 3.33913400
H -10.18548400 -1.68105100 1.45700100
H -8.56250700 2.60045900 -0.43258600
H -4.94211900 -0.47904800 5.73317900
O -3.94593500 1.63285500 5.01240800
C -10.69498500 1.69711400 -1.74820300
H -10.20465300 2.63001600 -2.03172500
H -10.70662400 1.03446000 -2.62065700
H -11.74065900 1.91707500 -1.50596400
C -11.70438000 -0.84945500 -0.66486700
H -12.58184600 -0.19518500 -0.61576100
H -11.54253100 -1.08470600 -1.72265300
H -11.94276300 -1.77795900 -0.14232100
C -6.57600600 2.95405400 1.22762400
H -5.49959700 2.94150700 1.39285400
H -6.76442200 3.06663200 0.16309300
C -7.17870200 4.10004000 2.03320700
H -6.95878900 3.97674500 3.09305000
H -6.77302800 5.04642100 1.67268000
O -8.60525700 4.17908500 1.83667900
C -9.39331700 3.53926400 2.73580100
O -8.96363400 2.92890800 3.69461200
C -10.84315300 3.65912900 2.35484100
H -11.04351900 2.96495200 1.53212100
H -11.46948200 3.39361100 3.20570800
H -11.07344500 4.66720600 2.00537900

(2.A.m), TRIPLET

C -10.50250400 -0.13931500 -0.05930700
C -9.86745100 -0.71114800 1.04071600
C -8.72554000 -0.15434700 1.63562800
C -8.20453900 1.06928500 1.05326100
C -8.85741900 1.64427700 -0.05827500
C -9.98540300 1.07257900 -0.62122500
N -8.18764100 -0.77193700 2.72077700
N -7.08997600 1.63959600 1.63231600
C -6.52812100 1.03479200 2.77780400
C -7.10995000 -0.15880800 3.26847000
C -6.49052800 -0.75868400 4.45064700
O -6.86617900 -1.79154100 5.00265700
N -5.39646600 -0.04619200 4.94233200
C -4.87414700 1.12733400 4.42512200
N -5.47907900 1.65374500 3.28991400
H -10.25503300 -1.62294500 1.48163300
H -8.49470000 2.57161300 -0.47757000
H -4.94458200 -0.42296800 5.76738400
O -3.90915700 1.68171000 4.94813000

C -10.65982100 1.71919400 -1.79890200
H -10.14196600 2.63412900 -2.09290200
H -10.68196500 1.04671200 -2.66394600
H -11.70056700 1.97508300 -1.57015300
C -11.72197300 -0.78274500 -0.64554100
H -12.57798200 -0.09855200 -0.61138200
H -11.56433700 -1.02920300 -1.70205800
H -11.98636600 -1.69636600 -0.11118100
C -6.57509000 2.94964300 1.21621900
H -5.50232300 2.95895000 1.39811700
H -6.74244500 3.06908600 0.14885300
C -7.20805100 4.08920600 2.01479500
H -6.97244700 3.98526600 3.07389700
H -6.83379000 5.04199700 1.63729600
O -8.63635700 4.12336400 1.83605900
C -9.39122800 3.46673800 2.75286700
O -8.92625100 2.88153800 3.71069700
C -10.84893500 3.53644200 2.39193900
H -11.03208100 2.86068300 1.55025000
H -11.45299400 3.22255600 3.24226400
H -11.12322300 4.54462100 2.07535100

(2.A.m)_1plus
C -10.48532200 -0.16990300 -0.07289600
C -9.85009100 -0.72774600 1.03532200
C -8.72488500 -0.13787200 1.60776600
C -8.19537600 1.08423400 1.02907600
C -8.85292900 1.65134100 -0.09292800
C -9.96696500 1.05543400 -0.64036100
N -8.17695300 -0.71864700 2.71281200
N -7.10708800 1.64765900 1.61005700
C -6.53764800 1.07079800 2.74506600
C -7.14462300 -0.14433100 3.25198700
C -6.53323900 -0.75787400 4.47382200
O -6.94809800 -1.77518100 4.99420400
N -5.44974900 -0.04899300 4.95451700
C -4.91133900 1.12061800 4.42228200
N -5.50504200 1.66277900 3.27079900
H -10.22411500 -1.63779300 1.48910500
H -8.49936700 2.57572600 -0.52337700
H -4.99376900 -0.41335600 5.78500100
O -3.95109900 1.66308800 4.93689800
C -10.64878400 1.67668900 -1.82066900
H -10.14937400 2.59602700 -2.12727000
H -10.65827900 0.98448700 -2.66936200
H -11.69328200 1.90772300 -1.58621200
C -11.69091100 -0.82175700 -0.66126400
H -12.55046400 -0.14177500 -0.62604800
H -11.52593900 -1.05669600 -1.71924700
H -11.94675300 -1.73936200 -0.13176900
C -6.57734000 2.96084900 1.18111900
H -5.50339200 2.95121500 1.35371900
H -6.75961600 3.07771600 0.11798100
C -7.20431500 4.08915300 2.00301500
H -6.94495600 3.98268500 3.05630300
H -6.83246300 5.03887100 1.61762700
O -8.63304900 4.12838900 1.85176500
C -9.36914500 3.43614100 2.76156600
O -8.87052400 2.73753700 3.62271700
C -10.83886600 3.62195600 2.52018900
H -11.11666100 3.10588500 1.59581400
H -11.40111900 3.19946700 3.35146300
H -11.07567400 4.68020500 2.39236500

(2.A.m)_1minus
C -10.50883600 -0.17275400 -0.06763700
C -9.84799600 -0.75458200 1.00631100
C -8.71103500 -0.17647100 1.61912400
C -8.23062400 1.05928500 1.08748100
C -8.89915100 1.64485500 0.00830300
C -10.02688000 1.05559800 -0.57638700
N -8.14185600 -0.80945500 2.68138500
N -7.09605100 1.63434600 1.68861100
C -6.51917400 1.03930100 2.80545200
C -7.07120200 -0.18953300 3.25357300
C -6.44583800 -0.80662100 4.40973100
O -6.78235300 -1.87286600 4.94808200
N -5.36933800 -0.07992700 4.91652100
C -4.86959800 1.13718300 4.43616600
N -5.48580900 1.68553400 3.35338000
H -10.20006900 -1.69393100 1.42353600
H -8.57631300 2.60268200 -0.37720100
H -4.90408000 -0.47200500 5.72536200
O -3.90058300 1.65521800 5.01324700
C -10.71775900 1.73961900 -1.72856800
H -10.21979900 2.67782200 -1.98602600
H -10.73090300 1.10893200 -2.62607000
H -11.76460800 1.96875400 -1.49419300
C -11.72195200 -0.83403600 -0.67253700
H -12.60553400 -0.18717500 -0.61322900
H -11.57109700 -1.05949500 -1.73511100
H -11.95553100 -1.77029600 -0.15960400
C -6.57699900 2.92902600 1.25339000
H -5.50113200 2.93044800 1.42705600
H -6.75372000 3.04369700 0.18457800
C -7.17626400 4.09194400 2.03776800
H -6.97287800 3.97138900 3.10119600
H -6.76379900 5.03765700 1.67980600
O -8.60230200 4.17676100 1.82304300
C -9.40901000 3.58806000 2.73547000
O -9.01560900 3.08789200 3.77092800
C -10.84160200 3.61388400 2.27435400
H -10.97400500 2.81824600 1.53350900
H -11.50527100 3.43284300 3.11950100
H -11.08204300 4.56355900 1.79265000

(2.A.t), with two acetates
C -10.88276600 -0.48173900 -0.00614700
C -9.88349300 -0.96896800 0.81092200
C -8.68674500 -0.25326900 1.03385800
C -8.49372500 1.00365600 0.39458000
C -9.51389300 1.50051600 -0.43288900
C -10.68751500 0.78410000 -0.63583600
N -7.75522200 -0.78593500 1.87394500
N -7.30787700 1.68303000 0.63719000
C -6.36737700 1.18205900 1.50650300

C -6.66281700 -0.10989500 2.09585600
C -5.64773700 -0.68035800 3.02546600
O -5.75727100 -1.76132300 3.58770100
N -4.55666000 0.14166500 3.19460300
C -4.33322300 1.40065600 2.59677200
N -5.28836400 1.89562400 1.74325900
H -9.98878900 -1.92249800 1.31754600
H -9.42013400 2.46924200 -0.90167200
H -3.82703100 -0.18670500 3.81703900
O -3.29803600 2.00050400 2.86515500
C -11.76063400 1.35727200 -1.51966800
H -11.46829100 2.32857800 -1.92239500
H -11.97629100 0.68664800 -2.35888200
H -12.69832600 1.48133100 -0.96667000
C -12.15331900 -1.25873700 -0.22396700
H -13.02958100 -0.68785900 0.10270900
H -12.30196200 -1.48939200 -1.28467100
H -12.13457400 -2.19956500 0.32958700
C -7.05629800 3.01695000 0.07386300
H -5.98570600 3.10461300 -0.09827300
H -7.57481000 3.10702100 -0.87800800
C -7.48298900 4.11654700 1.04760600
H -6.95272800 3.98848100 1.99177700
O -8.90353400 4.04483100 1.28517900
C -9.32416300 3.36980700 2.38816700
O -8.56998200 2.83182300 3.17217700
C -10.82503600 3.35481900 2.45979000
H -11.20090800 2.63976400 1.72079600
H -11.14142200 3.04200300 3.45402300
H -11.23567700 4.33596700 2.21427600
C -7.23604300 5.50423700 0.47647600
H -7.58377700 6.26832200 1.17598500
H -7.75427700 5.64277700 -0.47665700
O -5.81581600 5.61152300 0.28632600
C -5.38108900 6.75565800 -0.29192700
O -6.13070900 7.65087300 -0.62367500
C -3.88508000 6.74278200 -0.45229200
H -3.55683800 7.67647500 -0.90673800
H -3.40725600 6.61337000 0.52274900
H -3.58661500 5.89780900 -1.07921300

(2.A.th), with three acetates

C -10.78353500 -0.43055800 -0.54791100
C -9.94942500 -0.82210900 0.47874800
C -8.82364700 -0.05825600 0.85789700
C -8.52954000 1.14780700 0.16126900
C -9.38035600 1.54547300 -0.88280200
C -10.48679900 0.78325200 -1.23795100
N -8.06110900 -0.49471100 1.89982100
N -7.42143900 1.88030900 0.56653600
C -6.65241200 1.47422700 1.63299000
C -7.03769000 0.22638000 2.26349700
C -6.21097700 -0.23590800 3.41376900
O -6.41015900 -1.27065000 4.03503700
N -5.18545500 0.63073600 3.71748400
C -4.86862000 1.84268200 3.06754700
N -5.64783300 2.23560900 2.00700600
H -10.13694200 -1.73328700 1.03691100
H -9.21018700 2.47291000 -1.41003300
H -4.58540000 0.37637500 4.49391500
O -3.91383700 2.49448200 3.47539000
C -11.38068900 1.25113000 -2.35286800
H -11.02756300 2.19193200 -2.77854700
H -11.42919300 0.50601900 -3.15467300
H -12.40635900 1.39736600 -1.99642200
C -11.98340600 -1.25538000 -0.92909000
H -12.91036700 -0.68072100 -0.82423900
H -11.92751000 -1.58001000 -1.97404600
H -12.06103100 -2.14418500 -0.29987300
C -7.09069300 3.18121800 -0.03490700
H -6.00906000 3.28191500 -0.02841900
H -7.44012900 3.20439800 -1.06361400
C -7.70542700 4.30527200 0.80308400
H -7.31031300 4.24447200 1.81724200
O -9.13830200 4.12411200 0.84190400
C -9.68483300 3.55440900 1.94383400
O -9.03646100 3.20225700 2.90955000
C -11.17236200 3.42048400 1.78479000
H -11.38427200 2.73274800 0.96073100
H -11.60719900 3.03787800 2.70679900
H -11.61073200 4.38858500 1.53012300
C -7.48897900 5.69500500 0.19250400
H -8.26469200 5.87725000 -0.55365000
O -6.20279500 5.69443200 -0.45641500
C -6.06414700 6.49118500 -1.55127000
O -6.94928100 7.21061400 -1.96211200
C -4.69205300 6.33744600 -2.14483700
H -4.58199400 7.01043000 -2.99381600
H -3.93314600 6.55749300 -1.38934100
H -4.54515400 5.30259200 -2.46717200
C -7.45967500 6.81216300 1.23832700
H -6.55225700 6.72565300 1.83696100
H -7.49257600 7.78386500 0.74374100
O -8.61412300 6.73258700 2.09214100
C -8.40983500 6.37097500 3.38551900
O -7.31780300 6.11292600 3.84946200
C -9.70515100 6.31380600 4.14909400
H -10.53775800 6.70706000 3.56678700
H -9.90055600 5.26951300 4.40775000
H -9.59682900 6.87444700 5.08019700

(2.A.f), with four acetates

C -10.66493300 -0.19968400 -0.70290900
C -9.92297400 -0.66203900 0.36436200
C -8.80622100 0.04676600 0.85939400
C -8.42338900 1.26927500 0.23905600
C -9.18135500 1.74040600 -0.84488000
C -10.28131000 1.03219100 -1.31371600
N -8.13913900 -0.45625600 1.93625200
N -7.32412200 1.94372900 0.75359300
C -6.65267400 1.47139200 1.85784700
C -7.12515900 0.21503200 2.40646400
C -6.40489800 -0.31805800 3.59696700
O -6.68388900 -1.36760100 4.15987200
N -5.37914800 0.50104200 4.01229200
C -4.97463200 1.72459500 3.43729600

N -5.65569700 2.18257300 2.33592700
H -10.17994700 -1.58877100 0.86642600
H -8.94383000 2.68345400 -1.31534800
H -4.84992500 0.19765200 4.82192500
O -4.03558200 2.32867800 3.94339200
C -11.07488800 1.57810600 -2.46840700
H -10.66200900 2.52385000 -2.82378700
H -11.08641700 0.87064600 -3.30494800
H -12.11927600 1.74383300 -2.18167100
C -11.85667500 -0.96750200 -1.20855200
H -12.77186500 -0.36844200 -1.14515200
H -11.73255000 -1.24746700 -2.26050400
H -12.00692700 -1.88037000 -0.62885900
C -6.90391300 3.25030000 0.22884100
H -5.82271600 3.30701300 0.32359400
H -7.17387700 3.32303200 -0.82172700
C -7.53764500 4.36966900 1.05939800
H -7.21090400 4.26468400 2.09321500
O -8.97351600 4.24589700 0.99080800
C -9.61482900 3.66137700 2.03132000
O -9.04278300 3.23203800 3.01439100
C -11.09485300 3.61843300 1.78328600
H -11.29446200 3.02442000 0.88675200
H -11.59837500 3.17697800 2.64181000
H -11.46986900 4.62913800 1.60232400
C -7.22661700 5.75913000 0.49557000
H -7.87273000 5.93082800 -0.36742800
O -5.85441600 5.78536300 0.05981800
C -5.60934000 5.99229800 -1.26163600
O -6.48027600 6.04821700 -2.10477400
C -4.13600600 6.15389200 -1.50104600
H -3.93338700 6.14775400 -2.57097700
H -3.81174500 7.10665400 -1.07098900
H -3.57820700 5.35869500 -1.00108300
C -7.35500900 6.89609400 1.52985700
H -6.39948200 6.97981100 2.04968200
O -8.38718900 6.62147900 2.49516700
C -7.99359600 6.25953300 3.74940900
O -6.83520400 6.06654700 4.05312300
C -9.17585600 6.10320300 4.66365900
H -10.00828400 6.73306400 4.34902400
H -9.49473700 5.05719000 4.63021700
H -8.87495800 6.34126700 5.68433200
C -7.76408200 8.21948400 0.89516700
H -8.75583900 8.14000900 0.44545500
H -7.77947600 9.01305900 1.64699800
O -6.79171800 8.53394700 -0.11564300
C -7.22060800 9.28419900 -1.16113800
O -8.34694000 9.72861500 -1.24135500
C -6.13292200 9.44947100 -2.18658400
H -6.04752100 8.51680500 -2.75286000
H -6.38891700 10.26231900 -2.86518500
H -5.17139700 9.64117500 -1.70597200

(2.A.m)-[$K_2S_2O_4$], SINGLET
S -0.08250500 -0.95435300 -4.71253200
O -0.60439500 -1.62436400 -5.91212700
O 0.37494200 -1.86710200 -3.64568600
O 0.76060200 0.23392500 -4.93787600
S -2.96104000 -0.61743800 -2.06519700
O -1.76556600 -0.73168600 -1.18729400
O -3.98117700 -1.65218100 -1.85916600
O -3.44209100 0.76787200 -2.23199200
K -0.27682600 -3.01647300 -1.34865600
K -2.42355500 2.47062100 -4.11380600
O -2.44044900 -1.09078500 -3.62312900
O -1.43811400 -0.11473100 -4.04814700
C 5.38628900 0.58780300 -3.19281400
N 4.26202200 1.50620500 -3.44311200
H 6.06648900 0.62647000 -4.03960400
C 6.09265200 0.91951400 -1.88243800
C 4.42844000 2.67032200 -4.18096600
C 3.07157000 1.22994000 -2.81988700
H 5.39766700 0.84423200 -1.04682900
H 6.92881000 0.23357300 -1.73947700
O 6.66621100 2.24215400 -1.92229300
C 3.31653200 3.55084700 -4.30223900
C 5.64055100 3.03050700 -4.79293900
C 2.00024800 2.18306700 -3.02443000
N 2.98135000 0.14354200 -2.08027600
C 5.91966900 3.25912000 -1.42499000
C 3.45647800 4.74822600 -5.03735700
N 2.11537400 3.28052500 -3.71725000
C 5.76019500 4.21508800 -5.50939300
H 6.51690500 2.40751800 -4.68863900
C 0.69070200 1.86133000 -2.40193300
C 1.78406000 -0.16066400 -1.49483600
O 4.81730700 3.10948600 -0.93594800
C 6.62357200 4.57585600 -1.60392600
C 4.64656300 5.09807600 -5.64137700
H 2.58468700 5.39079300 -5.10375900
C 7.07919300 4.56891500 -6.13923200
O -0.31252800 2.56351200 -2.52541900
N 0.69213900 0.70602200 -1.67296800
O 1.62149500 -1.17009400 -0.80279600
H 6.51523000 4.88680800 -2.64823800
H 6.16678600 5.32648400 -0.95992200
H 7.69002000 4.48178000 -1.39162100
C 4.77405900 6.38333900 -6.41427300
H 7.82973500 3.79952500 -5.94990500
H 6.97593700 4.69041600 -7.22321100
H 7.45612000 5.52114400 -5.74972200
H -0.21648700 0.35945400 -1.34909700
H 5.54820900 7.02975200 -5.98614800
H 5.05759100 6.19542000 -7.45575000
H 3.83154300 6.93436700 -6.41229500
H 4.97433100 -0.41779100 -3.12185800

(2.B*.m)-[$K_2S_2O_4$], TRIPLET, T_1'
S -0.27200200 -1.02571700 -4.75667500
O -0.87859100 -1.75342900 -5.88082700
O 0.28618500 -1.88861200 -3.69492200
O 0.52695900 0.16492600 -5.09436200
S -2.97544500 -0.61432700 -1.92989000
O -1.73206600 -0.72647700 -1.11940300
O -3.99783300 -1.62411500 -1.63257400

O -3.44110400 0.77392900 -2.11116400
K -0.32986500 -3.05919000 -1.39646800
K -2.43164300 2.44439100 -4.04321700
O -2.55020800 -1.14494600 -3.49791100
O -1.58334100 -0.17879100 -4.01789000
C 5.43331300 0.60940700 -3.19118500
N 4.31099700 1.50360600 -3.49950300
H 6.13856300 0.63263900 -4.01787300
C 6.10480500 0.97917400 -1.86841000
C 4.47307500 2.67110600 -4.21433400
C 3.06355500 1.24197300 -2.89177100
H 5.39761400 0.88076000 -1.04484900
H 6.96374000 0.32593600 -1.70777700
O 6.62553500 2.32101100 -1.90362400
C 3.33626900 3.56796700 -4.32638800
C 5.70255300 3.02769700 -4.81059100
C 2.01529800 2.17722900 -3.06370400
N 2.98455100 0.11574800 -2.20811200
C 5.82587200 3.30860300 -1.42725800
C 3.52783700 4.75798600 -5.04111700
N 2.11838500 3.32791100 -3.76849200
C 5.86174700 4.21176000 -5.50880000
H 6.56104300 2.38016500 -4.70615800
C 0.73594000 1.83962500 -2.45054200
C 1.78216200 -0.21505700 -1.60140600
O 4.72053100 3.11209800 -0.96254300
C 6.47992800 4.65167900 -1.59477100
C 4.74463900 5.09994900 -5.62999300
H 2.67849800 5.42801100 -5.11779200
C 7.18875600 4.56155700 -6.12232300
O -0.28225900 2.53939300 -2.52745600
N 0.71788000 0.64932200 -1.74408200
O 1.67366500 -1.25970600 -0.94862400
H 6.43915500 4.92953000 -2.65296300
H 5.94493400 5.39640900 -1.00690200
H 7.53077600 4.61123000 -1.30149800
C 4.88373300 6.39051800 -6.37728100
H 7.92507500 3.77768300 -5.93532700
H 7.10297600 4.69740600 -7.20631300
H 7.57983800 5.50071800 -5.71487800
H -0.19476600 0.32001700 -1.41201400
H 5.66730700 7.01544600 -5.93286600
H 5.18402800 6.20980800 -7.41609100
H 3.94926000 6.95330300 -6.37849900
H 5.03969000 -0.40136800 -3.10536200

(2.A.m)–(1a)_1plus,

C -10.53619500 -1.38696200 2.30897800
C -9.24832400 -1.06709300 2.70508200
C -8.42437500 -0.23383800 1.93381000
C -8.93684500 0.33189500 0.71065200
C -10.26264900 0.03375600 0.33136000
C -11.05094500 -0.81108400 1.09109900
N -7.16190300 0.02195800 2.37813500
N -8.11044900 1.13849300 -0.02528700
C -6.80754600 1.38430800 0.39513000
C -6.38783200 0.75039600 1.63419500
C -4.97598200 0.97277200 2.06905000
O -4.48968800 0.46841200 3.06680800
N -4.27277600 1.79848600 1.22164200
C -4.74768300 2.40930100 0.05434300
N -6.06556800 2.16746000 -0.32972900
H -8.83387700 -1.46528700 3.62331200
H -10.69095000 0.47038400 -0.55821200
H -3.30463500 1.98604200 1.46022000
O -4.01398700 3.12775900 -0.60246100
C -12.44578600 -1.13109600 0.64620100
H -12.69413300 -0.61641000 -0.28239800
H -12.56177100 -2.20897900 0.48913400
H -13.17295800 -0.84202100 1.41238900
C -11.38743100 -2.29965000 3.13605300
H -12.29340400 -1.78453800 3.47487500
H -11.71657000 -3.16473800 2.55047700
H -10.84369000 -2.65809700 4.01065700
C -8.60565300 1.92781700 -1.17383400
H -7.78151400 2.03424100 -1.87638400
H -9.40922700 1.38177500 -1.65790300
C -9.05701900 3.31451500 -0.71899100
H -8.22461300 3.86370400 -0.27963500
H -9.44852000 3.85387600 -1.58210900
O -10.14073500 3.22958800 0.22395000
C -9.81644600 3.21915200 1.54259600
O -8.67015400 3.22448500 1.94767300
C -11.05102100 3.16151700 2.39609800
H -11.55128900 2.20118500 2.23746200
H -10.77596300 3.26056300 3.44493700
H -11.75045000 3.94937600 2.10736500
O -5.58028400 -1.30457900 -0.00643300
C -6.20850200 -2.29906300 0.37311500
N -7.42810600 -2.53946800 -0.25055800
C -7.65081100 -1.84595900 -1.53517100
C -8.46801300 -3.46802500 0.17190200
C -8.96518900 -2.26249600 -2.18089000
H -7.59363600 -0.76736200 -1.37542500
H -6.80564900 -2.09339900 -2.18791000
C -8.82979700 -4.47645500 -0.93237300
H -8.16807000 -3.97022200 1.08264800
H -9.35683400 -2.87190800 0.42235000
C -9.05034000 -3.78985800 -2.30249100
H -9.81217100 -1.87690200 -1.60378600
H -9.01845200 -1.78952500 -3.16512300
H -9.73007700 -4.99983800 -0.59922000
H -8.03034500 -5.21901900 -1.00092600
H -10.01792900 -4.07332100 -2.72406200
H -8.28454200 -4.12462300 -3.00966600
C -5.60991500 -3.26319600 1.42130100
C -6.33133600 -3.17953600 2.78518400
H -7.37680100 -3.49110700 2.75187500
H -5.81684500 -3.84132200 3.48801900
H -6.28899500 -2.16185900 3.17864600
C -5.59611100 -4.71143300 0.88086400
H -4.99493700 -5.32805700 1.55479500
H -6.58398900 -5.16900600 0.82243300
H -5.13913900 -4.75554300 -0.11230200
C -4.14819900 -2.82571100 1.64466800
H -4.09369700 -1.81589800 2.05322200

H -3.68212800 -3.51368800 2.35565100
H -3.57819800 -2.85258600 0.71280200

(2.A.m)–(1a), singlet
C -10.78948900 -1.29715400 2.36253700
C -9.47990800 -1.06581100 2.73505900
C -8.62058300 -0.25145500 1.96798500
C -9.10841500 0.36170400 0.78238800
C -10.44430700 0.13732900 0.41195800
C -11.27539300 -0.67745000 1.17379100
N -7.33217900 -0.08844300 2.38534500
N -8.23379300 1.15592400 0.05087200
C -6.92583700 1.31677000 0.44122900
C -6.52903500 0.62496800 1.65308200
C -5.10525900 0.74757400 2.06901700
O -4.62351000 0.19436900 3.04971200
N -4.37045400 1.55502600 1.23535900
C -4.81957400 2.20774100 0.06851300
N -6.13967700 2.07643300 -0.28684500
H -9.06362500 -1.51653200 3.62958400
H -10.86163100 0.61359400 -0.46328400
H -3.38743900 1.66825100 1.45472800
O -4.01504400 2.87002600 -0.57790300
C -12.69575100 -0.90207300 0.73428300
H -12.91828300 -0.35815100 -0.18532600
H -12.88594100 -1.96681500 0.55917900
H -13.40182500 -0.57799400 1.50667900
C -11.68242500 -2.18579600 3.18578400
H -12.56775700 -1.64533400 3.53838900
H -12.04274300 -3.03821400 2.59926200
H -11.15012400 -2.57304100 4.05677600
C -8.68967600 1.92187400 -1.12085200
H -7.85013800 1.99088700 -1.81113500
H -9.49641800 1.37892800 -1.60632700
C -9.11493400 3.33083100 -0.72221100
H -8.28119800 3.86636600 -0.26892300
H -9.47090400 3.86622100 -1.60348400
O -10.22593100 3.29128500 0.19623800
C -9.93926600 3.32980100 1.52127400
O -8.81216600 3.42652800 1.96479200
C -11.19196800 3.19285600 2.34192700
H -11.49380300 2.14017800 2.33918600
H -10.99524300 3.50431700 3.36713400
H -12.00793000 3.77562000 1.91079000
O -5.25243200 -1.43846400 -0.19360300
C -5.94270000 -2.37707400 0.23578700
N -7.16534600 -2.59904900 -0.33070300
C -7.47841300 -1.79986400 -1.53361700
C -8.26453500 -3.42282900 0.17728400
C -8.78930100 -2.25434400 -2.17809700
H -7.50731000 -0.73801700 -1.28569600
H -6.65109800 -1.92994000 -2.23907800
C -8.75788600 -4.42574400 -0.87480100
H -7.95789500 -3.93409700 1.08170100
H -9.08313700 -2.75460200 0.46776900
C -8.82680100 -3.78205600 -2.28140100
H -9.64742000 -1.89067100 -1.60140800
H -8.86049700 -1.79110100 -3.16709800
H -9.74509900 -4.78003700 -0.55943000
H -8.09604300 -5.29746900 -0.89229300
H -9.72402200 -4.11227400 -2.81314400
H -7.96836300 -4.10840600 -2.88026400
C -5.36682400 -3.32346500 1.32415800
C -6.05266800 -3.13339400 2.69523300
H -7.11477300 -3.38578700 2.69377700
H -5.56206200 -3.78330300 3.42792500
H -5.95567400 -2.09900200 3.03016500
C -5.43498200 -4.79528900 0.86097400
H -4.88146100 -5.42051300 1.56889900
H -6.44977600 -5.19052200 0.80416000
H -4.97247700 -4.91388500 -0.12419600
C -3.87991800 -2.95883900 1.50437900
H -3.76659400 -1.93084500 1.85073500
H -3.43635700 -3.62911400 2.24787900
H -3.32815300 -3.06868600 0.56732500

(2.B*.m)–(1a)_triplet
C -10.54261300 -1.32573900 2.32149500
C -9.25602200 -1.00021100 2.72514300
C -8.39972900 -0.18345500 1.96090200
C -8.92823800 0.35950100 0.72424000
C -10.24299000 0.04311200 0.33461200
C -11.05135000 -0.79098200 1.09147000
N -7.14927100 0.05826500 2.41977600
N -8.10793500 1.18293600 -0.02605200
C -6.78242900 1.40363600 0.40093900
C -6.35611800 0.80359600 1.61136400
C -4.95840100 0.98997400 1.98630100
O -4.42053700 0.51333400 2.98687400
N -4.23388900 1.78207700 1.09925700
C -4.71367400 2.37805300 -0.05662400
N -6.04094600 2.15159500 -0.39245700
H -8.86016900 -1.39021200 3.65558200
H -10.65467700 0.46051400 -0.57302800
H -3.25962900 1.94269300 1.32608100
O -3.98407500 3.07649200 -0.75897300
C -12.43958200 -1.12962100 0.62648500
H -12.66746500 -0.63447100 -0.31932700
H -12.55571400 -2.20991200 0.48215500
H -13.19296700 -0.82578600 1.36200000
C -11.39559600 -2.22873000 3.16187100
H -12.31966100 -1.72440700 3.46716500
H -11.69695300 -3.11802500 2.59657000
H -10.86491200 -2.55256500 4.05853500
C -8.61852700 1.98307000 -1.14564000
H -7.80036700 2.13336100 -1.84711000
H -9.40445600 1.42733100 -1.65072800
C -9.11538000 3.34956700 -0.67683200
H -8.29929000 3.91912600 -0.23182100
H -9.53419000 3.89318800 -1.52508700
O -10.18591100 3.21012000 0.27661000
C -9.85095200 3.22128400 1.59092900
O -8.71596300 3.37940000 1.99495100
C -11.05969300 2.97439600 2.45046400
H -11.33482200 1.91770200 2.37070600
H -10.82651700 3.20817700 3.48842200

H -11.90958700 3.56589300 2.10414700
O -5.45428400 -1.44510900 -0.11420300
C -6.16784400 -2.36057600 0.32153200
N -7.38480200 -2.56493800 -0.27731900
C -7.61998600 -1.81598100 -1.52888700
C -8.46316300 -3.45819600 0.14470300
C -8.92436600 -2.24133300 -2.19843200
H -7.60454400 -0.74357700 -1.32693300
H -6.77466100 -2.01311800 -2.19784000
C -8.84509100 -4.45661500 -0.95681900
H -8.17594400 -3.97941300 1.04919600
H -9.33225600 -2.84362700 0.40670400
C -8.98459100 -3.76645800 -2.33792100
H -9.78350200 -1.87581300 -1.62574800
H -8.97525200 -1.75805100 -3.17898500
H -9.78491500 -4.93239400 -0.65856000
H -8.08874900 -5.24618400 -1.00440000
H -9.91590400 -4.06604700 -2.82722300
H -8.16765100 -4.08580100 -2.99495000
C -5.63052300 -3.29507600 1.43861300
C -6.40794800 -3.16739000 2.76656200
H -7.44466200 -3.50216000 2.70489700
H -5.91160900 -3.78223600 3.52482000
H -6.41114800 -2.12805300 3.10132500
C -5.60650000 -4.75806800 0.94127700
H -5.07950000 -5.37673500 1.67454900
H -6.59897600 -5.18983600 0.80543900
H -5.07130600 -4.83671200 -0.01055600
C -4.17586100 -2.87101900 1.72214400
H -4.12882900 -1.84577900 2.09279000
H -3.75364900 -3.53770100 2.48100800
H -3.56038000 -2.93782300 0.82173000

(2.A.m)-[$K_2S_2O_8$]–(1a), Singlet, S_0

S 2.06451200 -0.68474600 -5.86102100
O 0.97567600 -1.33129000 -6.60835200
O 2.31413900 -1.26774100 -4.52214900
O 3.27854900 -0.35611100 -6.63045500
S 0.15733800 1.80695100 -3.57989000
O 1.14613500 1.22174300 -2.64539700
O -1.25416600 1.58013400 -3.23444600
O 0.50475700 3.17375500 -4.02445500
K 1.75973100 -1.30308600 -1.96109500
K 0.90805500 5.46078600 -2.68090400
O 0.19989800 0.85093700 -4.98925200
O 1.53356400 0.92643400 -5.58566100
C 5.22355900 0.14133500 -4.18954600
N 4.54378300 1.42823200 -3.94445200
H 5.13375800 -0.10076900 -5.24271000
C 6.66853500 0.19939800 -3.71226300
C 4.50165600 2.41861500 -4.91486100
C 4.05468600 1.65701500 -2.68875100
H 6.71785700 0.41671900 -2.64530800
H 7.15615500 -0.75374700 -3.92225100
O 7.41414000 1.19563500 -4.44451700
C 3.95129800 3.68446500 -4.56179600
C 4.99478700 2.24274500 -6.21801600
C 3.56060200 2.98566500 -2.41805200
N 4.05763500 0.66962000 -1.80838500
C 7.50204100 2.43313600 -3.90073600
C 3.88920800 4.71274900 -5.52857400
N 3.50413600 3.94265900 -3.30367200
C 4.92613600 3.26398300 -7.15513500
H 5.43276600 1.30289400 -6.51472100
C 3.07943200 3.27100800 -1.04271700
C 3.51980000 0.88343300 -0.57348600
O 7.02067700 2.73427800 -2.82549600
C 8.22758500 3.37237600 -4.82435500
C 4.35782100 4.53000600 -6.81196900
H 3.46463200 5.66181300 -5.21787100
C 5.45721200 3.03063400 -8.54248000
O 2.65079400 4.36504500 -0.68315200
N 3.14769600 2.18922600 -0.20404800
O 3.31929100 -0.03216600 0.23155500
H 7.54172400 3.66616200 -5.62597600
H 8.53715400 4.26131100 -4.27591300
H 9.08807500 2.88261400 -5.28371900
C 4.28235400 5.63694600 -7.82855700
H 5.86100200 2.02226300 -8.64830200
H 4.66908000 3.16650000 -9.29144700
H 6.24983700 3.74699900 -8.78536600
H 2.76775200 2.30350000 0.72879300
H 5.27811900 5.90793500 -8.19640200
H 3.69254400 5.33526500 -8.70114400
H 3.82311200 6.52975100 -7.39930000
H 4.68472800 -0.62019600 -3.63181700
O -0.45661700 4.64002400 -0.59977500
C -0.36774200 4.39887400 0.61918700
N -0.59617900 3.12980700 1.04645600
C 0.02019100 5.53947000 1.59621200
C -0.87306500 2.13365800 -0.01181900
C -0.76293900 2.64872300 2.42021300
C -1.18090600 5.92904900 2.48757800
C 1.26968700 5.17286100 2.42824400
C 0.38935100 6.77594600 0.75330900
C -1.05744300 0.73205500 0.56791200
H -1.74949600 2.44281300 -0.58907200
C 0.13706800 1.43846600 2.69672100
H -0.55036800 3.44238000 3.12506300
H -1.81546200 2.37568300 2.56594900
H -1.50809800 5.13238800 3.15726200
H -0.90772800 6.78969200 3.10647200
H -2.03681500 6.21805500 1.86895800
H 1.59140000 6.05120200 2.99741300
H 1.10774500 4.36622300 3.14408800
H 2.08291900 4.87915300 1.75969900
H -0.43177900 7.07409600 0.09814500
H 0.61924200 7.61004200 1.42360700
H 1.27256800 6.57987300 0.14098200
C 0.06646000 0.39215900 1.55305300
H -2.03214800 0.64338500 1.06176500
H -1.07036600 0.02624300 -0.26849800
H -0.17469400 1.00096100 3.65078900
H 1.16748500 1.78588200 2.83248600
H -0.08221600 -0.61238500 1.96166400
H 1.01961700 0.36822800 1.01630900

H -0.02819100 2.13329100 -0.70263500

(2.B*.m)-[$K_2S_2O_8$]–(1a), triplet, T_1
S -2.21606500 -1.27804600 -4.75422700
O -3.06938700 -2.38223000 -5.21780400
O -0.96339000 -1.70689900 -4.09402000
O -2.09496100 -0.11335900 -5.64889000
S -2.77282400 -0.75817900 -1.01782800
O -1.29949300 -0.83681100 -1.19074700
O -3.32967000 -1.71816600 -0.05602300
O -3.29516600 0.62042500 -0.96053700
K -0.31768000 -3.24115800 -1.98956100
K -2.61071900 2.31034700 -3.03762100
O -3.43242000 -1.41781300 -2.45325700
O -3.14145100 -0.48121800 -3.53164900
C 5.83442000 0.78609900 -3.08366000
N 4.70764500 1.52576600 -3.66618200
H 6.69807700 0.89082500 -3.73569600
C 6.14611300 1.24393100 -1.65897200
C 4.87927400 2.70726700 -4.35395300
C 3.39591400 1.09374600 -3.36567900
H 5.28298200 1.08746500 -1.01198600
H 7.00390400 0.68300100 -1.28541200
O 6.53508300 2.63154800 -1.62273800
C 3.69589300 3.43102100 -4.77925100
C 6.15853700 3.23615000 -4.63845100
C 2.30578800 1.88324900 -3.80661800
N 3.30213200 -0.04196700 -2.69737600
C 5.56249500 3.53832400 -1.36531800
C 3.89092300 4.63805400 -5.45751500
N 2.42096300 3.01649900 -4.53352400
C 6.32212900 4.43447900 -5.31067800
H 7.04382500 2.71619400 -4.30255800
C 0.97361900 1.42349600 -3.43094900
C 2.04195800 -0.52452000 -2.37673300
O 4.40414900 3.23084100 -1.15967300
C 6.09250000 4.94262600 -1.41794700
C 5.15955500 5.15537500 -5.73267700
H 3.00390200 5.18052700 -5.76501100
C 7.69878900 4.97315600 -5.58315000
O -0.07392600 2.01753100 -3.70649100
N 0.94539100 0.24414400 -2.70235500
O 1.90775400 -1.61524100 -1.80665200
H 6.11811100 5.26011700 -2.46561100
H 5.42504600 5.60436900 -0.86676400
H 7.10807600 4.99739200 -1.02344000
C 5.29924800 6.45775600 -6.45710900
H 8.46577900 4.30405700 -5.18883100
H 7.87238400 5.09583600 -6.65811700
H 7.83734700 5.95858100 -5.12444700
H 0.02721200 -0.11929000 -2.43444100
H 5.84437200 7.18597500 -5.84481800
H 5.88077000 6.33147100 -7.37787900
H 4.32628500 6.87845100 -6.71422300
H 5.55634900 -0.26530400 -3.04972700
O -1.31550400 4.12515100 -1.55446600
C -0.21283500 4.28699100 -0.99384500
N 0.24252400 3.31759800 -0.15741000
C 0.57738300 5.59685100 -1.24917500
C -0.66927600 2.17500800 0.05873600
C 1.55464400 3.22859500 0.50079700
C 0.95966000 6.30643100 0.06751500
C 1.81730500 5.32312600 -2.12949100
C -0.34606600 6.54785300 -2.03553600
C -0.22648000 1.32139800 1.24017000
C 2.11197400 1.80723500 0.38207600
H 2.25141000 3.91101000 0.03020600
H 1.46150200 3.51650500 1.55590000
H 1.69850400 5.76498800 0.65776400
H 1.38339400 7.28859000 -0.16566100
H 0.07565700 6.46432200 0.69433700
H 2.28144800 6.27840800 -2.39892100
H 2.58136300 4.71318000 -1.64645900
H 1.53023800 4.81318600 -3.05276500
H -1.25214600 6.78262700 -1.47070200
H 0.18919900 7.48147000 -2.23519600
H -0.64731300 6.10809000 -2.98827500
C 1.18121100 0.74901200 1.01803000
H -0.25421600 1.92384600 2.15583900
H -0.95348500 0.51498300 1.36966400
H 3.10731600 1.78979400 0.83365100
H 2.25979400 1.60643800 -0.68076200
H 1.59027100 0.39976400 1.97194600
H 1.11595400 -0.12445800 0.36182300
H -1.67786700 2.56292600 0.20906700
H -0.69917300 1.55736900 -0.84163100

(2.C.m)-[$K_2S_2O_8$]_1min, doublet
S -0.83257000 -1.26260000 -4.88232000
O -1.43624800 -2.22308300 -5.81903300
O -0.04499700 -1.87658300 -3.79572100
O -0.26175000 -0.03766300 -5.47513700
S -3.09195000 -0.64078600 -1.75320400
O -1.74939300 -0.68733500 -1.11861200
O -4.08012600 -1.55893500 -1.17258300
O -3.55496900 0.72471300 -2.06872600
K -0.29058000 -2.97482300 -1.40560300
K -2.38641200 2.22512700 -4.10571300
O -2.91113700 -1.40697800 -3.27835300
O -2.16744700 -0.48099800 -4.12215700
C 5.52440600 0.66571000 -3.17233900
N 4.39483300 1.54008500 -3.48410700
H 6.25131700 0.71972200 -3.98133200
C 6.16562000 1.00588400 -1.83038800
C 4.57003000 2.73522800 -4.20671200
C 3.15982400 1.23913400 -2.92837800
H 5.43323900 0.91212000 -1.02923300
H 7.01544700 0.34628000 -1.64324300
O 6.70065800 2.34724700 -1.84264900
C 3.43146500 3.57835600 -4.38930400
C 5.80197000 3.12726400 -4.73907500
C 2.09087200 2.14222900 -3.16653900
N 3.08097100 0.11069600 -2.21356000
C 5.91908500 3.33629800 -1.35075600
C 3.61260600 4.77641700 -5.11953400
N 2.19820200 3.29343000 -3.88735000

C 5.95677800 4.32100500 -5.45287000
H 6.68414900 2.52392900 -4.57277200
C 0.81179800 1.79637500 -2.58976300
C 1.88477100 -0.23714700 -1.67851100
O 4.84674800 3.15111400 -0.80942300
C 6.54574600 4.68229400 -1.59687000
C 4.83622900 5.16209000 -5.64955200
H 2.73684700 5.40673600 -5.24668900
C 7.31162000 4.70119000 -5.99298100
O -0.23899400 2.46648900 -2.69748100
N 0.79971300 0.61599300 -1.86833200
O 1.71319400 -1.28583200 -1.01812500
H 6.36433200 4.95562900 -2.64177000
H 6.08889400 5.42697400 -0.94558000
H 7.62586900 4.64664400 -1.44276700
C 4.96939100 6.45641400 -6.41182000
H 8.06097000 3.94485000 -5.74680400
H 7.29466600 4.81560900 -7.08374400
H 7.65454600 5.65965000 -5.58457200
H -0.11116900 0.29052300 -1.54400900
H 5.69611000 7.12854900 -5.94011400
H 5.32045600 6.28625700 -7.43663700
H 4.01196500 6.98025900 -6.46617200
H 5.14994600 -0.35585800 -3.11575600

MSX-1

MSX (T_1)/S_0 (E = -4260.04863529 a.u.)

S	-3.503613	-2.164740	-1.883975
O	-4.702348	-2.916057	-2.285555
O	-2.999836	-2.489400	-0.531766
O	-2.471323	-1.962057	-2.914119
S	-4.606331	0.816891	0.307742
O	-3.481823	0.216266	1.075295
O	-5.862060	0.928147	1.059812
O	-4.216712	1.972651	-0.519837
K	-4.115921	-2.335740	1.892365
K	-2.282209	1.646245	-2.438642
O	-5.085853	-0.388079	-0.810382
O	-4.025175	-0.514631	-1.803437
C	3.369324	-3.196634	1.736088
N	2.716026	-2.578460	0.577618
H	4.321551	-3.609873	1.400860
C	3.559072	-2.296331	2.954783
C	3.436448	-1.888241	-0.436280
C	1.369710	-2.307151	0.608321
H	2.605880	-1.887631	3.283952
H	4.012734	-2.885786	3.753276
O	4.486145	-1.213180	2.712237
C	2.760577	-1.555563	-1.669656
C	4.792314	-1.602848	-0.329800
C	0.866298	-1.766159	-0.638063
N	0.663258	-2.356837	1.701332
C	3.959172	-0.033146	2.318445
C	3.481324	-0.889789	-2.689811
N	1.498287	-2.085584	-1.862546
C	5.483836	-0.931405	-1.347467
H	5.335127	-1.869873	0.566272
C	-0.122376	-0.702420	-0.503164
C	-0.600156	-1.761425	1.662614
O	2.767489	0.143514	2.151718
C	5.022303	1.000451	2.074557
C	4.812503	-0.555868	-2.549123
H	2.941255	-0.642233	-3.597343
C	6.937673	-0.608937	-1.157591
O	-0.418530	0.109285	-1.378790
N	-0.831994	-0.796471	0.687152
O	-1.448253	-2.003907	2.518149
H	5.203683	1.050958	0.996476
H	4.660255	1.977549	2.397359
H	5.955332	0.749067	2.578257
C	5.542300	0.178604	-3.640314
H	7.299290	-0.963547	-0.191079
H	7.549006	-1.065468	-1.944246
H	7.110125	0.471968	-1.215656
H	-1.746420	-0.330862	0.717590
H	5.923833	1.140125	-3.279078
H	6.404754	-0.391354	-4.001613
H	4.881841	0.373591	-4.487298
H	2.742691	-4.034573	2.050659
O	-0.624267	3.375501	-1.278119
C	0.274817	3.237848	-0.423536
N	-0.084241	3.066882	0.874445
C	1.757217	3.287397	-0.878715
C	-1.535911	3.144697	1.152927
C	0.788838	2.824087	2.031566
C	2.573794	4.336003	-0.092693
C	2.393837	1.883715	-0.795924
C	1.773375	3.707293	-2.361744
C	-1.818398	3.198568	2.649424
C	0.224966	1.717483	2.928302
H	1.769099	2.512404	1.692905
H	0.910688	3.755370	2.599224
H	2.763295	4.058564	0.943737
H	3.546161	4.466323	-0.577633
H	2.066588	5.306372	-0.095949
H	3.422466	1.926909	-1.168887
H	2.421347	1.464694	0.210271
H	1.836476	1.191794	-1.429640
H	1.339468	4.700562	-2.503585
H	2.809535	3.728123	-2.713659
H	1.211058	3.001898	-2.976573
C	-1.237213	1.974231	3.366228
H	-1.405081	4.121761	3.072623
H	-2.902891	3.249452	2.789714
H	0.889793	1.623527	3.791815
H	0.304041	0.778924	2.379535
H	-1.300751	2.115690	4.450063
H	-1.849793	1.099978	3.122538
H	-1.935381	4.025807	0.643853
H	-2.036369	2.277886	0.716332

(2.E.m)-[$K_2S_2O_8$]–(1a’), triplet, (T)–1

S -1.50451100 -1.06447400 -4.83061000
O -2.48311500 -1.84530900 -5.60347800
O -0.61987200 -1.87438800 -3.96784500
O -0.85794600 0.05734100 -5.53060600

S -2.88769200 -0.54723000 -1.27011400
O -1.41841300 -0.67263400 -1.10175800
O -3.68330700 -1.56622500 -0.57306900
O -3.38023800 0.84349000 -1.18281500
K -0.26331100 -3.05094700 -1.61805300
K -2.64140200 2.66021900 -3.07031700
O -3.20868500 -1.02233900 -2.87786900
O -2.46779700 -0.11387200 -3.74867900
C 5.73544500 0.75983200 -3.44956100
N 4.57818400 1.56675000 -3.85471200
H 6.52746300 0.88353300 -4.18484300
C 6.21166100 1.11272200 -2.04295900
C 4.74243800 2.79390900 -4.52458500
C 3.33407000 1.16910800 -3.39942200
H 5.41982400 0.92835300 -1.31769100
H 7.09433900 0.52060100 -1.79592600
O 6.62472100 2.49371600 -1.96902500
C 3.60529700 3.58850400 -4.81477300
C 5.99339100 3.28841100 -4.91530500
C 2.22793200 1.99008900 -3.69636000
N 3.23414500 0.04151600 -2.70624000
C 5.70898800 3.39261800 -1.53505000
C 3.74151000 4.81797800 -5.47107300
N 2.37024100 3.13549300 -4.41554500
C 6.13340700 4.51320000 -5.56728300
H 6.89089400 2.73544100 -4.67971900
C 0.92929700 1.66175800 -3.17077700
C 2.00940600 -0.34237400 -2.24200000
O 4.59292900 3.09086700 -1.16000000
C 6.24353700 4.79522800 -1.62425700
C 4.98679600 5.29615000 -5.85389300
H 2.84674800 5.40003800 -5.66982800
C 7.50499700 5.00196000 -5.94995200
O -0.05847300 2.39309900 -3.34941900
N 0.90473200 0.49966400 -2.44208800
O 1.83818700 -1.40494300 -1.62262200
H 6.11262400 5.14587600 -2.65349100
H 5.67977400 5.44527300 -0.95563300
H 7.30873300 4.82851400 -1.39037900
C 5.11374000 6.62428500 -6.55041700
H 8.27602200 4.28678700 -5.65652200
H 7.58184900 5.16218900 -7.03131900
H 7.73070900 5.96193700 -5.47193400
H 0.00841200 0.18901800 -2.05551400
H 5.74461800 7.31346100 -5.97799100
H 5.58031400 6.51258500 -7.53559000
H 4.13672700 7.09209700 -6.68780800
H 5.42414000 -0.28423200 -3.45345600
O -1.53630900 4.02842400 -1.06721500
C -0.36600000 4.14135000 -0.65374100
N 0.11258000 3.18318800 0.21953400
C 0.49725000 5.34291700 -1.11127600
C -0.67457000 2.07913800 0.51587200
C 1.39533100 3.22167500 0.94554200
C 0.54413100 6.39109400 0.02405200
C 1.93208200 4.95828200 -1.56196700
C -0.22177800 5.96798600 -2.32370600
C -0.15424300 1.07321400 1.48036600
C 2.15420600 1.90648800 0.74667700
H 1.98935900 4.05883500 0.60715400
H 1.17521100 3.38142800 2.00750600
H 1.03698400 6.00815400 0.92167300
H 1.09828600 7.27383700 -0.31169100
H -0.46643700 6.70839500 0.30028500
H 2.22918600 5.57789400 -2.41332000
H 2.68606100 5.11221000 -0.78891300
H 2.00074100 3.91665500 -1.87253300
H -1.24404900 6.25923000 -2.07807000
H 0.33091200 6.85407600 -2.65060900
H -0.26107700 5.25584200 -3.15300100
C 1.31420800 0.68021800 1.18171700
H -0.20907000 1.45907100 2.51279400
H -0.79757500 0.19116100 1.44366900
H 3.09152600 1.96636500 1.30767500
H 2.43348400 1.83480300 -0.30718000
H 1.74643600 0.21508800 2.07416600
H 1.32521400 -0.07267100 0.39109700
H -1.60750900 1.98467600 -0.01229200
H 1.53136000 3.68792700 -4.56337100

(2.F.m)-[$K_2S_2O_8$]–(1.A), singlet, (S)–1

S -1.45083900 -1.07829900 -4.80801700
O -2.44883400 -1.86417500 -5.54930600
O -0.61900500 -1.86582000 -3.87605300
O -0.74429000 -0.02862700 -5.56063700
S -2.96935900 -0.32171200 -1.35239900
O -1.57133800 -0.73007900 -1.05580400
O -4.00825400 -1.11217400 -0.68453400
O -3.14841400 1.14847700 -1.34200700
K -0.28013500 -3.03634900 -1.52528000
K -2.59174500 2.74271700 -3.50040000
O -3.26776000 -0.79627600 -2.95803500
O -2.38901200 -0.01032100 -3.81662600
C 5.59469800 0.62523300 -3.58524900
N 4.42492900 1.42726600 -3.93201100
H 6.36620900 0.78477400 -4.33904500
C 6.13662100 0.88407600 -2.17995600
C 4.57804200 2.73673600 -4.48030900
C 3.20144200 1.06967800 -3.36574000
H 5.35930000 0.69720100 -1.44108400
H 6.99901900 0.24046200 -1.99245700
O 6.62380800 2.23848400 -2.03557000
C 3.42655100 3.51114800 -4.74681600
C 5.82368400 3.29293800 -4.77497100
C 2.09122400 1.87150900 -3.62155100
N 3.16871600 -0.03498900 -2.59984000
C 5.78942600 3.14932700 -1.49142800
C 3.56845400 4.80582400 -5.24535400
N 2.16470500 2.94365600 -4.54635600
C 5.96771800 4.58670800 -5.29981700
H 6.72550300 2.73851500 -4.55554400
C 0.90069500 1.62455500 -2.92535600
C 1.99754200 -0.38769500 -2.02102300
O 4.69199700 2.88294600 -1.03927400
C 6.37823100 4.53254900 -1.55003100
C 4.82117100 5.36166400 -5.53173600

H 2.66868900 5.38953600 -5.42492800
C 7.34612000 5.13199800 -5.58117900
O -0.11767700 2.37841100 -2.94719600
N 0.90913800 0.47847600 -2.13893300
O 1.84831400 -1.44093700 -1.35247000
H 6.11639000 4.96653300 -2.52073900
H 5.94866400 5.14840800 -0.75971100
H 7.46611200 4.50672400 -1.47249500
C 4.92474100 6.76553100 -6.07189800
H 8.11735500 4.39625400 -5.33929900
H 7.46639700 5.40966800 -6.63539400
H 7.55097900 6.03594700 -4.99452900
H 0.00721500 0.13660300 -1.80728000
H 5.52032400 7.40752700 -5.41178900
H 5.41544800 6.78545200 -7.05245400
H 3.93715200 7.22069100 -6.18113000
H 5.30184500 -0.42479500 -3.62528300
O -1.49587000 4.26225400 -1.04095400
C -0.30839700 4.28218800 -0.83340000
N 0.20115600 3.23398900 0.09823600
C 0.65240100 5.31464200 -1.42251000
C -0.43259200 2.09782500 0.12169600
C 1.34109000 3.40528100 1.03596700
C 0.67784500 6.53256000 -0.46244500
C 2.07867600 4.78250000 -1.67731600
C 0.02847300 5.75844400 -2.76298300
C -0.04829700 1.04688600 1.08272300
C 2.25118400 2.17964200 0.93486200
H 1.85868800 4.32491200 0.79796000
H 0.90973300 3.50833000 2.03664300
H 1.08461100 6.28620100 0.52147000
H 1.31442100 7.30373500 -0.90491300
H -0.32549800 6.94503800 -0.32797900
H 2.53583100 5.37440700 -2.47406800
H 2.73808000 4.86141100 -0.81387500
H 2.08126600 3.74370000 -2.00266200
H -0.97471200 6.16434600 -2.62184600
H 0.66095700 6.52858700 -3.21071300
H -0.03573000 4.90767400 -3.44449700
C 1.48516200 0.88988800 1.27740100
H -0.50404400 1.36948500 2.03398600
H -0.53410800 0.11488700 0.79934000
H 3.09719300 2.32616100 1.61022400
H 2.65994000 2.13921300 -0.07606100
H 1.66250400 0.59875800 2.31488900
H 1.82119100 0.07006400 0.63992900
H -1.27908000 1.97837800 -0.54917100
H 1.39942800 3.59825200 -4.44289900

MSX-2

MSX (T)-1/S_0 (E = -4260.09988392 a.u.)

S	-3.440243	-2.529157	-1.760539
O	-4.565622	-3.252686	-2.372140
O	-3.233890	-2.816719	-0.326570
O	-2.215750	-2.411485	-2.568632
S	-4.897781	0.500650	0.087166
O	-3.840705	-0.038648	0.978904
O	-6.247067	0.533591	0.663923
O	-4.471686	1.705454	-0.658152
K	-3.965764	-2.426381	2.186491
K	-2.632404	1.546451	-2.660527
O	-5.156168	-0.689169	-1.106934
O	-3.906848	-0.859300	-1.840435
C	3.337024	-2.742865	1.776735
N	2.876492	-2.007677	0.597826
H	4.178774	-3.372157	1.492939
C	3.694831	-1.818511	2.938225
C	3.788327	-1.572111	-0.392834
C	1.552214	-1.593727	0.569709
H	2.820661	-1.248601	3.250821
H	4.081122	-2.405360	3.773544
O	4.754660	-0.910574	2.564710
C	3.313451	-0.804783	-1.486002
C	5.154735	-1.862373	-0.346810
C	1.118412	-0.821967	-0.517950
N	0.754993	-1.958311	1.575692
C	4.396386	0.321804	2.133505
C	4.204933	-0.354879	-2.465217
N	1.971019	-0.510828	-1.555528
C	6.046329	-1.422448	-1.330666
H	5.560455	-2.411245	0.490580
C	-0.219620	-0.337543	-0.551330
C	-0.547215	-1.566831	1.556215
O	3.252712	0.733191	2.131842
C	5.588385	1.084896	1.627435
C	5.564705	-0.650107	-2.408562
H	3.812448	0.240151	-3.284542
C	7.508617	-1.767550	-1.222194
O	-0.659396	0.388102	-1.470115
N	-0.985979	-0.721094	0.530814
O	-1.363952	-1.913866	2.433790
H	5.806429	0.742593	0.610502
H	5.359081	2.149650	1.604446
H	6.469458	0.892268	2.241397
C	6.497590	-0.144615	-3.476953
H	7.706762	-2.366274	-0.330555
H	7.852001	-2.336260	-2.094315
H	8.131060	-0.866702	-1.171151
H	-1.975350	-0.467158	0.527643
H	7.286493	0.486236	-3.051857
H	6.999485	-0.971331	-3.992576
H	5.959597	0.443782	-4.223317
H	2.522008	-3.389605	2.101206
O	-1.301217	3.327247	-1.085654
C	-0.346142	3.277453	-0.311374
N	-0.618126	2.891337	1.028039
C	1.077771	3.627020	-0.784683
C	-1.847871	2.421733	1.325223
C	0.311243	3.013806	2.174015
C	1.413150	5.068957	-0.335335
C	2.164490	2.628754	-0.306572
C	1.041205	3.590066	-2.325997
C	-2.208197	2.144558	2.733034
C	0.304025	1.714257	2.980903
H	1.304309	3.238381	1.812967
H	-0.028135	3.857406	2.785237

H	1.440762	5.173684	0.751975
H	2.397353	5.348412	-0.724031
H	0.678787	5.778217	-0.727816
H	2.918074	2.511691	-1.090160
H	2.692460	2.956779	0.589202
H	1.753996	1.642079	-0.097746
H	0.297250	4.279937	-2.726924
H	2.025681	3.868022	-2.712237
H	0.797226	2.583630	-2.676766
C	-1.104774	1.370421	3.503880
H	-2.378457	3.110553	3.238945
H	-3.155675	1.605940	2.751224
H	1.018466	1.817852	3.801469
H	0.687891	0.921012	2.336903
H	-1.194139	1.612738	4.566832
H	-1.286522	0.299527	3.393623
H	-2.545674	2.316507	0.508464
H	1.606033	0.096829	-2.277883

(2.F.m)-[$K_2S_2O_8$]_1min, singlet, (S)–2

S 0.70859200 -1.38917000 -4.78163700
O 0.27261700 -2.35334700 -5.80296700
O 1.44212600 -1.98182200 -3.64768200
O 1.23909800 -0.10258500 -5.28294400
S -1.71427700 -0.92455600 -1.76857400
O -0.42751100 -1.01699200 -1.03923200
O -2.77346500 -1.81904600 -1.27886100
O -2.11548000 0.45575800 -2.10616600
K 1.32274200 -3.04648000 -1.25030800
K -0.38605100 1.97637200 -3.83769800
O -1.44903100 -1.68227700 -3.29616500
O -0.73756300 -0.72550200 -4.12752200
C 5.77264600 0.63839900 -4.35628900
N 4.66384000 1.55011400 -4.10064500
H 6.08827500 0.74799800 -5.39441900
C 6.95264700 0.81717200 -3.40394000
C 4.59481500 2.82400200 -4.72891900
C 3.80609600 1.26255400 -3.03927000
H 6.62264200 0.69085000 -2.37397300
H 7.73562700 0.09323800 -3.64116500
O 7.56475600 2.11774500 -3.55873500
C 3.50006100 3.67510600 -4.46112600
C 5.57520300 3.27893300 -5.61524600
C 2.75354300 2.13200000 -2.77315400
N 4.04076200 0.13180800 -2.35131900
C 7.17871000 3.09700100 -2.71095600
C 3.44254300 4.92991900 -5.06520200
N 2.45595900 3.24120500 -3.62160500
C 5.51187600 4.53690200 -6.23190700
H 6.44660200 2.66865500 -5.80847600
C 1.84937700 1.82457300 -1.74129200
C 3.17760100 -0.22416500 -1.37168500
O 6.41717700 2.93129800 -1.77801900
C 7.79442200 4.41455800 -3.10051600
C 4.42770800 5.38264400 -5.94982700
H 2.59257800 5.56929500 -4.83742800
C 6.61205000 4.96878600 -7.16974900
O 0.82388800 2.50648100 -1.46023400
N 2.14301300 0.65348100 -1.04973300
O 3.25955900 -1.30383600 -0.73657800
H 7.20010200 4.83780100 -3.91710900
H 7.77279100 5.09758500 -2.25151200
H 8.81563600 4.28108600 -3.46187800
C 4.32055600 6.74974400 -6.57664000
H 7.37602100 4.19297400 -7.26459700
H 6.22733100 5.18536100 -8.17377300
H 7.10545400 5.88382900 -6.82014700
H 1.41261900 0.30217600 -0.44165800
H 5.18178100 7.37881200 -6.32101500
H 4.29145300 6.69059800 -7.67133400
H 3.41703800 7.26702000 -6.24407600
H 5.40425600 -0.37985500 -4.22385300
H 2.01096100 3.99246400 -3.10345600

(2.F.m)-[$K_2S_2O_8$]_1min, triplet, (S)–2 (t)

S -1.03893400 -1.58330500 -4.39262600
O -1.81078500 -2.63327000 -5.07422100
O 0.21126700 -2.04352500 -3.76013400
O -0.95987800 -0.27720600 -5.08119200
S -1.64597800 -1.24921800 -0.59634200
O -0.16613000 -1.18764200 -0.68371800
O -2.16979600 -2.23346200 0.35991900
O -2.29634100 0.07612700 -0.61605500
K 1.13121100 -3.37085000 -1.67207500
K -1.79917100 1.68980400 -2.84684900
O -2.16650300 -2.01670500 -2.04704800
O -2.05538700 -1.03287100 -3.11103300
C 6.40840400 0.84628000 -4.37197800
N 5.17794900 1.63569700 -4.38020200
H 6.97567600 1.05036200 -5.27842100
C 7.24847000 1.11012900 -3.12433500
C 5.13322900 2.88818300 -4.97299500
C 4.11313600 1.14454700 -3.62714900
H 6.67686600 0.86898700 -2.22849600
H 8.16476600 0.51701700 -3.15818900
O 7.66615800 2.48905300 -3.07907800
C 3.93018000 3.68054200 -4.85695500
C 6.20069200 3.46718500 -5.70060800
C 2.92553000 1.95011700 -3.52990500
N 4.19215000 -0.09672500 -3.06743000
C 6.89134400 3.35060900 -2.37408700
C 3.85084900 4.94194600 -5.45806600
N 2.87227700 3.16457600 -4.13612500
C 6.11509700 4.72094300 -6.28486100
H 7.14144800 2.93825900 -5.77911900
C 1.81652700 1.49823000 -2.77146700
C 3.16380300 -0.55389000 -2.35415200
O 5.97804100 3.00681700 -1.64786800
C 7.31936300 4.77052700 -2.62499300
C 4.90543500 5.48949500 -6.17573600
H 2.92534800 5.50349300 -5.34637300
C 7.29672900 5.28011200 -7.03485500
O 0.74040800 2.14850700 -2.64103800
N 2.01193100 0.27298300 -2.15793200
O 3.12535100 -1.69014600 -1.80289600
H 7.00486500 5.05170600 -3.63626800

H 6.85124800 5.43278000 -1.89729600
H 8.40711800 4.86137000 -2.57846100
C 4.79271000 6.85302300 -6.80353900
H 8.13989200 4.58458500 -7.01993000
H 7.04983500 5.48706300 -8.08407300
H 7.63710700 6.23050500 -6.60497900
H 1.20844000 -0.15687800 -1.70669700
H 5.54229400 7.55316100 -6.41042600
H 4.94168200 6.82289400 -7.89118800
H 3.80643300 7.28746800 -6.61748100
H 6.12413600 -0.20625100 -4.36826700
H 2.01528300 3.69353000 -4.01692500

(2.A.m)-[KSO_4_$KHOSO_3$]_1min, singl, (S)–3
S 0.79040800 -0.14383300 -5.48741200
O 1.28892800 -0.68484800 -6.79388200
O 1.74241700 -0.52184400 -4.35831100
O 0.64354400 1.36142300 -5.54249800
S -1.50377000 -0.39589900 -0.49535300
O -1.17770400 1.15474400 -0.13195700
O -2.57707500 -0.76816400 0.43629900
O -1.88697900 -0.41244500 -1.93922800
K 0.10921800 -2.17637600 -2.96121100
K -1.42627900 1.53213700 -3.82115200
O -0.21629400 -1.14104100 -0.28370800
O -0.57574600 -0.74117900 -5.14290600
C 6.10849800 0.52438500 -4.17531400
N 4.88045700 1.32669500 -4.05202900
H 6.52069700 0.66127900 -5.17164300
C 7.11811800 0.88631100 -3.09132700
C 4.72770800 2.52844200 -4.73341600
C 3.93683200 0.90096300 -3.15360100
H 6.69417800 0.71033400 -2.10320800
H 8.02236900 0.29057000 -3.22418700
O 7.52932200 2.26406000 -3.20694100
C 3.56149700 3.30091500 -4.47565700
C 5.67746800 3.02787000 -5.63985900
C 2.77428400 1.75137000 -2.99256800
N 4.15001600 -0.21796400 -2.49432000
C 6.86062900 3.17809100 -2.46164800
C 3.38807500 4.53577100 -5.13619600
N 2.60379800 2.89132900 -3.59550200
C 5.48892700 4.24598300 -6.28232900
H 6.59304300 2.48743500 -5.83202000
C 1.73900700 1.27813500 -2.05164300
C 3.17619500 -0.68287100 -1.65877100
O 5.96679300 2.89503200 -1.68853100
C 7.34886100 4.56918300 -2.75858500
C 4.31909800 5.02229900 -6.03177800
H 2.48565100 5.09396000 -4.90895300
C 6.53466500 4.74849700 -7.23977400
O 0.66408700 1.88306200 -1.87525700
N 2.05571200 0.14199500 -1.38466500
O 3.21297800 -1.78690800 -1.11770600
H 6.90950300 4.89378800 -3.70760000
H 7.02923600 5.24707400 -1.96789500
H 8.43457900 4.58821800 -2.86846500
C 4.11227500 6.34341700 -6.72312000
H 7.36891000 4.04935900 -7.32088000
H 6.11196200 4.89777400 -8.23960400
H 6.92868800 5.71845800 -6.91654700
H 1.32102100 -0.28540600 -0.80221800
H 4.92261100 7.04425900 -6.49335400
H 4.09481800 6.22467700 -7.81221500
H 3.16969900 6.80082200 -6.41562500
H 5.82662500 -0.52059000 -4.05362400
H -0.44400100 1.46107300 -0.74285900

[K_2SO_4]
S 0.91129800 1.02441100 -2.92941400
O 2.25782500 0.32963700 -2.85716800
O 0.20460500 0.92835000 -1.59049700
O 1.10117800 2.49117200 -3.26718700
K 1.86317900 -1.46929600 -4.77790800
K -0.05154700 3.53475000 -1.10768500
O 0.08008900 0.34887800 -4.00380400

$CuBF_4$
Cu 0.33323200 1.23640300 -4.27284500
B -1.35375900 1.16291400 -2.18718400
F -2.62346200 1.68166900 -2.23302700
F -0.38601600 2.18894700 -2.53212700
F -1.03069200 0.60025400 -0.97807100
F -1.17735400 0.18924300 -3.25036900

(LH)– $CuBF_4$
N -1.41176200 1.43913400 0.39947200
C -0.50534400 1.81399000 -0.71681700
C -0.57992900 3.31496900 -1.01029000
C -2.03966300 3.78014000 -0.98710400
C -2.64707200 3.58381800 0.42213700
C -1.83802000 2.56732400 1.23974800
H 0.51308000 1.49291700 -0.46914100
H 0.01276400 3.88646000 -0.28763600
H -2.11796800 4.82580700 -1.29672200
H -2.65225800 4.52511900 0.98073900
H -2.39912900 2.21365000 2.09468300
H -3.68620100 3.24983900 0.34407400
H -0.92770100 3.03142100 1.63206900
H -2.60415400 3.19051500 -1.71872200
H -0.12695000 3.48678500 -1.99097700
H -0.82101900 1.24593700 -1.59330700
C -1.69042900 0.14211200 0.55427500
O -1.15946500 -0.66050200 -0.28104500
Cu 0.02979300 -2.04849700 0.17018000
B 1.30860700 -3.68091000 2.05056500
F 0.87390800 -4.96455200 2.28240300
F 2.58812100 -3.44624600 2.49508100
F 1.29456600 -3.46012300 0.57830400
F 0.39957900 -2.72572800 2.55393400
C -2.64107000 -0.40472800 1.64280700
C -2.01758600 -0.22111000 3.04655000
H -1.85359000 0.82084100 3.32401600
H -2.68935800 -0.66066500 3.79000200
H -1.06085500 -0.74733700 3.10417100
C -2.83623300 -1.91964400 1.42653400

H -3.58309700 -2.27811800 2.14036200
H -3.18652200 -2.14390600 0.41688600
H -1.91512100 -2.47805800 1.61474400
C -4.03931300 0.24735000 1.52899600
H -4.05231100 1.31624100 1.73785900
H -4.45159900 0.09508700 0.52695800
H -4.71057800 -0.23327700 2.24605700

($Na_2S_2O_4$)– $CuBF_4$
Cu 0.71627900 2.20603500 -3.60792300
B 0.82203700 2.78723200 -6.35935300
F 0.79545400 3.94031700 -7.08833100
F 0.41124300 1.66666200 -7.09514400
F 2.04745800 2.55581900 -5.72873900
F -0.16416800 2.90130100 -5.24738900
S 0.83999900 -0.08619900 -1.81495400
O -0.14270900 -0.06186600 -3.22685000
O 1.43255900 1.29239400 -2.05668100
O -0.07307000 -0.15309200 -0.67494200
O 1.81720300 -1.16643200 -1.95942200
O -1.09876100 -1.16593200 -3.19083900
Na -1.54781300 0.94527600 -6.06771200
S -0.85423400 -2.14692000 -4.58948800
O -2.04013400 -2.99853100 -4.42555600
O -0.90489300 -1.19002900 -5.72427500
O 0.45355900 -2.79982000 -4.42315800
Na 2.74651300 -0.67144500 -5.31849900

[(LH)–($NaSO_4$)]–[$NaSO_4$–$CuBF_4$], trip
Cu 1.56805400 1.88684300 -4.03361100
B -0.75175900 1.62220900 -2.44245600
F -1.91794500 2.32075600 -2.15707800
F 0.39502300 2.46610000 -2.32523300
F -0.61397000 0.47797500 -1.67975700
F -0.79171800 1.24241800 -3.83587600
S 2.67922500 -0.45099900 -4.39463900
O 2.60066300 0.78768600 -5.34662500
O 4.06609100 -0.84438700 -4.11993100
O 1.99672600 0.21933700 -3.14027400
O 1.79194400 -1.54653800 -4.86135200
O -2.21904000 -1.27760900 -4.52571700
Na -3.15007800 1.70963400 -4.11608600
S -2.45850100 -0.87705500 -5.93427400
O -3.30818500 -1.90510700 -6.68517000
O -3.02156500 0.49309600 -6.04755000
O -1.21420100 -1.05393800 -6.80598600
Na -0.14655900 -1.17649800 -3.48283200
O 1.21940600 3.51453000 -4.95948100
C 0.12066200 4.01058000 -5.38317600
C -0.31743700 5.34929200 -4.74653600
C 0.73828500 5.74542400 -3.69372500
H 0.81977400 4.98595500 -2.91418600
H 1.72307000 5.88470100 -4.14456600
H 0.43193300 6.68793200 -3.23149700
C -1.66210700 5.19349900 -3.99635600
H -2.50479400 4.93146500 -4.63684400
H -1.58152400 4.43055500 -3.21948800
H -1.90056900 6.14513500 -3.51234500
C -0.36854700 6.47464300 -5.80502400
H -0.54251300 7.42642000 -5.29512300
H 0.58530800 6.54688600 -6.33616400
H -1.15887500 6.35152000 -6.54542900
N -0.55947500 3.39189700 -6.34778900
C -0.05074600 2.14975700 -6.97079900
C -1.81445300 3.83821800 -6.98352200
C 0.21828800 2.37389000 -8.46154700
H -0.81756100 1.38548500 -6.83134200
H 0.85410000 1.82758700 -6.46884800
C -1.59069500 4.11947000 -8.47233200
H -2.53467900 3.02206800 -6.86490300
C -1.03374000 2.88184300 -9.18290200
H 0.56148700 1.42759600 -8.89216900
H 1.03553700 3.09747800 -8.56975300
H -2.54471500 4.42889400 -8.91106300
H -0.89393500 4.95984300 -8.57594200
H -0.80871200 3.11023500 -10.22936400
H -1.79650600 2.09215900 -9.18225700
H -2.21039600 4.70704100 -6.47606800

[(LH)–($NaSO_4$)]–[$NaSO_4$–$CuBF_4$], sing
Cu -0.59056000 2.04520600 -2.02829500
B 2.49659100 2.45591300 -2.46560600
F 3.19257000 3.56783500 -2.03045400
F 3.29934100 1.64069500 -3.29872200
F 1.98159900 1.71365400 -1.39674300
F 1.38265800 2.87121400 -3.29136700
S -1.16277100 -0.27047300 -3.89583200
O 0.26280500 0.20171400 -4.72337800
O -0.71358200 0.18793500 -2.52872900
O -2.24121400 0.52806500 -4.48343100
O -1.25688000 -1.72456000 -3.99792000
O 0.15846500 -0.27881800 -6.10025800
Na 1.98782500 1.90235600 -5.26115100
S 1.63039700 -1.12209600 -6.44312300
O 1.35310300 -1.42464700 -7.85309400
O 2.66941700 -0.08788400 -6.19799900
O 1.68903400 -2.26532500 -5.51899800
Na 2.26297400 -1.52110700 -2.39852500
O -0.80878800 3.79419200 -1.30232100
C -0.13342000 4.82912500 -1.59829500
C -0.55093400 5.64043900 -2.85032700
C -1.61539700 4.82516600 -3.61274500
H -1.19958000 3.88651600 -3.99134000
H -2.47373900 4.58978000 -2.98150500
H -1.96096500 5.41182600 -4.46856000
C 0.61376600 5.89289200 -3.83799700
H 0.19153200 6.23860700 -4.78625900
H 1.32024100 6.65497500 -3.50992100
H 1.16459700 4.96983000 -4.01957800
C -1.20586300 6.96701300 -2.39832500
H -2.05916500 6.77241600 -1.74145100
H -0.51670900 7.62778800 -1.86926400
H -1.57140100 7.50360900 -3.27893200
N 0.85866500 5.19800000 -0.78044700
C 1.22131600 4.35155700 0.37447600
C 1.68669000 6.40891500 -0.85848900

C 1.03629700 5.12234700 1.68206500
H 2.26897700 4.06727700 0.23514400
H 0.61559600 3.45190900 0.34482800
C 1.54350800 7.24017500 0.42172100
H 2.72314700 6.08030900 -0.99717400
H 1.41015300 7.00132600 -1.71988100
C 1.86283400 6.41315900 1.67132600
H 1.32990300 4.47728500 2.51692600
H -0.02770400 5.35769800 1.80998400
H 2.20568100 8.10892600 0.34661700
H 0.51509500 7.61816400 0.47883700
H 1.67460300 7.00102200 2.57550100
H 2.93057000 6.15648600 1.67191400

TS(H-transf), trip
Cu 1.41768100 1.62681600 -3.93997500
B -0.62969000 1.65544800 -1.58385200
F -1.75075400 2.44930000 -1.87655300
F 0.52357700 2.27373800 -2.23400600
F -0.38905900 1.54198100 -0.24595000
F -0.82571000 0.39466900 -2.20038700
S 2.65621500 -0.61332700 -4.49368800
O 2.44087000 0.68484100 -5.34734100
O 4.08000200 -0.90047500 -4.28614900
O 1.96036100 -0.08365800 -3.17756200
O 1.84751400 -1.74136600 -5.01671600
O -1.70512100 -0.61117800 -4.82234700
Na -2.79071100 1.14116700 -3.52355300
S -2.42467100 -0.24937400 -6.09060400
O -3.16820500 -1.38426300 -6.68727200
O -3.23977400 0.98036900 -5.85320700
O -1.34473400 0.07252000 -7.21295900
Na -0.07614100 -1.49513400 -3.48314300
O 0.96135800 3.29269600 -4.84532100
C 0.01674700 3.90562100 -5.40594300
C -0.42197300 5.25288800 -4.78061900
C 0.57332000 5.59052100 -3.64706600
H 0.57956000 4.81694300 -2.87776600
H 1.59134000 5.70663700 -4.02822800
H 0.26892700 6.53597800 -3.18939800
C -1.82492200 5.11474700 -4.13016000
H -2.62720600 4.90695100 -4.83984100
H -1.81923800 4.32324000 -3.37622900
H -2.06503100 6.05822400 -3.63031800
C -0.36327400 6.41189700 -5.80670700
H -0.56138800 7.34929200 -5.27881100
H 0.63326100 6.48420200 -6.25308800
H -1.09042700 6.33788700 -6.61543900
N -0.51713600 3.39943300 -6.55885800
C 0.08674400 2.25728100 -7.18191200
C -1.77624400 3.85092500 -7.20973600
C 0.23780500 2.36806400 -8.70109300
H -0.63796300 1.29682800 -7.03360300
H 1.00994100 1.97715300 -6.68261100
C -1.57965200 4.09602600 -8.70805500
H -2.50552100 3.05045200 -7.04363300
C -1.03972600 2.83987700 -9.39785200
H 0.56527800 1.39520500 -9.07912000
H 1.05702000 3.07750900 -8.88913300
H -2.54603700 4.39051900 -9.12977000
H -0.89122100 4.93719300 -8.85433000
H -0.83959900 3.03577900 -10.45590100
H -1.79237400 2.04359100 -9.35314100
H -2.14394500 4.74028600 -6.71604100

[L-OSO$_3$Na]–[NaHSO$_4$–CuBF$_4$], singlet
Cu 1.45386500 1.78398500 -4.29552300
B -1.29284700 1.80989600 -2.13612300
F -2.66943000 2.06298500 -2.25657600
F -0.61543700 2.83687000 -1.52273700
F -1.07480700 0.57268000 -1.50094900
F -0.81018500 1.66077700 -3.49883200
S 2.80591500 -0.77457000 -3.74333100
O 3.69988400 -0.17265000 -4.74486300
O 3.39994200 -1.61579500 -2.70458300
O 1.86032100 0.27597500 -3.13895400
O 1.77196300 -1.76781500 -4.53281200
O -1.51046300 -0.65265800 -5.17912400
Na -2.81848800 1.34117800 -4.58762500
S -0.84103000 -0.26040700 -6.45171200
O -1.33036900 -0.94753400 -7.65092800
O -1.32601000 1.32440300 -6.55277400
O 0.65044300 -0.19952100 -6.34230700
Na -0.29391700 -0.84829400 -3.14304100
O 1.12250700 3.23541300 -5.49117600
C 0.08520800 3.92142000 -5.68820800
C -0.14580500 5.16774800 -4.80790500
C 0.91657700 5.16310400 -3.68885100
H 0.77927000 4.30931400 -3.02093100
H 1.92964400 5.13733500 -4.09332000
H 0.80091100 6.07598400 -3.09795800
C -1.52682100 5.15046600 -4.11092900
H -2.37330600 5.27857100 -4.78292400
H -1.66452900 4.22289000 -3.55301000
H -1.55462000 5.97591900 -3.39377600
C 0.06579000 6.44726000 -5.65274900
H -0.03729000 7.31932600 -5.00094500
H 1.07287000 6.45948300 -6.07994900
H -0.64665800 6.55968400 -6.47143800
N -0.74427600 3.55770300 -6.70294700
C -0.49437000 2.26194400 -7.31909800
C -2.01643300 4.22374200 -7.06394700
C -0.82455200 2.20439100 -8.80158000
H 0.54155400 1.99643800 -7.14059400
C -2.25646100 4.21719400 -8.57197400
H -2.84151500 3.71171800 -6.55820800
C -2.19357000 2.79582600 -9.12742700
H -0.74363300 1.16148800 -9.11760200
H -0.04193000 2.76682700 -9.32428400
H -3.23335600 4.67515400 -8.75429800
H -1.50242400 4.84456900 -9.06225100
H -2.35735600 2.79046100 -10.20861500
H -2.98265500 2.18280100 -8.67526700
H -1.99059700 5.24402800 -6.70508600
H 1.39588200 -1.26221800 -5.31031400

[(LOH)–CuBF$_4$]–[NaHSO$_4$]$_2$, singlet
Cu 0.91141300 1.31715900 -4.50014300
B -1.91163000 1.58561100 -1.92620300
F -3.23166900 1.87584400 -2.31381200
F -1.30905000 2.65150100 -1.29888500
F -1.86744900 0.41889400 -1.14289600
F -1.19578300 1.27914100 -3.15425100
S 2.48810100 -1.08332800 -3.96937300
O 2.88266000 -0.52383900 -5.27807200
O 3.57555200 -1.46579800 -3.06335500
O 1.46200000 -0.16404100 -3.29234500
O 1.66871900 -2.45792700 -4.23043100
O -2.83768400 -0.51795200 -5.21549400
Na -2.98265500 1.67419200 -4.64634600
S -1.57326000 -1.15090400 -5.67023100
O -1.68472700 -1.92898600 -6.90939300
O -0.52686500 0.07647700 -5.96271400
O -0.87864600 -1.87499600 -4.55232700
Na -0.56111200 -0.95157400 -2.45262100
O 0.88683200 2.90953200 -5.60133900
C 0.00630000 3.79815200 -5.74244000
C -0.07419200 4.93991600 -4.70474800
C 1.09327700 4.76518900 -3.71283600
H 1.02150600 3.81726600 -3.17423000
H 2.06070100 4.80130800 -4.21870100
H 1.05302000 5.57627100 -2.98038800
C -1.37865700 4.89981000 -3.87448300
H -2.29257500 4.88317400 -4.46907800
H -1.38029100 4.02909700 -3.21842400
H -1.41331300 5.79076700 -3.24015800
C 0.11629800 6.30242100 -5.41339200
H 0.21101800 7.08185200 -4.65220000
H 1.03209500 6.30170800 -6.01206200
H -0.71182500 6.58108400 -6.06518900
N -0.75703600 3.77797300 -6.86442700
C -0.44663200 2.78966100 -7.89519800
C -1.99585000 4.54504100 -7.10779400
C -0.45250900 3.41596700 -9.28724200
H 0.53404300 2.37955700 -7.66617400
C -1.97337200 5.22548000 -8.47520300
H -2.82907600 3.83533300 -7.05545800
C -1.73483200 4.19713600 -9.58290200
H -0.27786700 2.62395000 -10.02390500
H 0.41331700 4.08586000 -9.33560400
H -2.92622000 5.74407800 -8.61857000
H -1.18020700 5.98282600 -8.48886400
H -1.65540100 4.68516100 -10.55835500
H -2.58869900 3.51118700 -9.63620800
H -2.13533000 5.27074700 -6.31842100
H 0.73755300 -2.24128600 -4.52365500
O -1.43184200 1.73677200 -7.77267300
H -1.54626400 1.30500000 -8.63227400
H -0.89398900 0.65898600 -6.69775000

[(LNHCO)–CuBF$_4$]–[NaHSO$_4$]$_2$, singlet
Cu 0.79563400 1.46916000 -4.51065500
B -1.49587000 0.48813000 -1.23160300
F -2.83600800 0.86651900 -1.44588000
F -0.95606200 1.11834900 -0.13534100
F -1.39189100 -0.91540300 -1.13496600
F -0.77057300 0.86416700 -2.42638300
S 2.17451500 -0.90822500 -5.54809000
O 1.96137000 0.02830900 -6.67264700
O 3.52912900 -1.44418700 -5.39204400
O 1.63231900 -0.30227300 -4.24706100
O 1.24151100 -2.20926900 -5.79111300
O -3.25998600 -0.33528300 -4.58269700
Na -2.68938800 1.80843000 -3.56497000
S -2.00621000 -0.47369200 -5.35390300
O -2.35952900 -0.82663800 -6.86852500
O -1.24303600 0.81797500 -5.38664000
O -1.17057200 -1.62576600 -4.87832500
Na 0.07139700 -1.38515500 -2.85899600
O 0.46425000 3.36546600 -4.44368300
C -0.26561300 4.13849800 -5.12314200
C -0.39361900 5.59370400 -4.64124500
C 0.61864700 5.82266500 -3.50373800
H 0.45369200 5.13016600 -2.67653500
H 1.64577100 5.69439000 -3.85553400
H 0.50691200 6.84493500 -3.13153500
C -1.82073000 5.76873400 -4.06808700
H -2.59928300 5.64455700 -4.82356700
H -2.00406000 5.04430400 -3.26793400
H -1.91846200 6.77356200 -3.64694200
C -0.10871300 6.63301500 -5.74527400
H -0.07092800 7.62481000 -5.28576700
H 0.85986600 6.44852200 -6.21967100
H -0.87279300 6.66632100 -6.52073800
N -0.95944300 3.64030100 -6.15891400
C -0.47267700 1.29522500 -8.35299600
C -1.77868300 4.29132500 -7.18555400
C 0.22358600 2.46237700 -8.96932800
H 0.17157900 0.51462300 -7.92036400
C -0.99504800 4.70012000 -8.44620700
H -2.56702900 3.58083800 -7.44620300
C -0.68388800 3.59462400 -9.46581500
H 0.81672600 2.03341300 -9.79171900
H 0.97767900 2.80714900 -8.24770500
H -1.58845300 5.46664900 -8.95779600
H -0.06209300 5.18421800 -8.13947100
H -0.19814100 4.06907500 -10.32362100
H -1.61837600 3.16087200 -9.83689400
H -2.26901800 5.16312100 -6.75733300
H 0.29175100 -1.97320000 -5.58864100
O -1.69909400 1.18650300 -8.30654500
H -0.93673900 2.62528800 -6.19581600
H -2.15504900 -0.02105100 -7.46561300

LNHCO
O -0.40862400 6.47387600 -6.50172600
C -0.90856100 5.35570700 -6.33939000
C -0.98632900 4.72595700 -4.93380300
C 0.46656900 4.44847300 -4.49276300
H 0.92915800 3.69318300 -5.13446900
H 1.06016400 5.36510000 -4.54130300
H 0.47877400 4.07970900 -3.46171200

C -1.80216400 3.42461300 -4.87420800
H -2.83665000 3.57900000 -5.20119400
H -1.34808100 2.63033400 -5.47334900
H -1.83984900 3.06938500 -3.83972400
C -1.62164800 5.77372000 -4.00039800
H -1.61202100 5.40856700 -2.96862500
H -1.06840700 6.71384100 -4.04658400
H -2.66172200 5.97266400 -4.28080500
N -1.37668600 4.63191600 -7.39144000
C 0.38910300 2.40153100 -7.96593000
C -1.47045200 5.20857000 -8.73369200
C 1.29959100 3.37545300 -8.66792300
O 0.57604100 1.96641700 -6.84392900

[$(NaHSO_4)_2$–$CuBF_4$]

Cu 2.27429600 0.98275300 -3.78130100
B 0.98186700 1.82372100 -1.39640100
F 1.13831200 2.43666600 -0.18972900
F 2.13449500 2.14780800 -2.25080700
F 0.96644500 0.41883600 -1.28543800
F -0.16069200 2.26000000 -2.08159100
S 0.81917100 -0.74203300 -5.69786900
O -0.16441300 0.58448900 -5.68391300
O 0.84536100 -1.27398300 -7.05413300
O 2.17108400 -0.22391100 -5.25642600
O 0.18435700 -1.59178800 -4.65996200
O -1.96076800 -0.61545400 -2.47650100
Na -1.47862200 1.41674300 -3.72671600
S -3.01970200 -1.06402300 -3.44768000
O -4.24048700 -1.58207100 -2.82101800
O -3.21803000 -0.01417700 -4.48651600
O -2.39371000 -2.36723400 -4.21164900
Na 0.14459200 -1.43931300 -2.28681700
H -1.50937400 -2.11396100 -4.56936600
H 0.09422000 1.18832400 -6.40265200

C -0.12473100 5.41544400 -9.43765500
H -2.10660100 4.54239100 -9.32316500
C 0.65453400 4.15186800 -9.82673400
H 2.11532400 2.75339400 -9.06713100
H 1.75416000 4.03779700 -7.92372100
H -0.33679300 5.98068000 -10.35336600
H 0.49875800 6.05815300 -8.80935900
H 1.45179200 4.45201100 -10.51427900
H 0.00546700 3.47407900 -10.39878700
H -1.97789700 6.17745200 -8.66203100
H -0.46732400 2.03355100 -8.57244500
H -1.90879800 3.79838100 -7.19822700

Table S20. Cartesian coordinates of the optimized geometries for the autocyclization process. The cartesian coordinates of optimized geometries are given below in the standard XYZ format, and units are in Å.

==============================

$Cu^{I}(H_2O)_2$

==============================

Cu	-0.000001	-0.015904	-0.000001
O	-1.916053	-0.041990	0.045146
H	-2.466433	-0.497143	-0.607432
H	-2.459021	0.619412	0.496257
O	1.916053	-0.041999	-0.045135
H	2.459003	0.619319	-0.496388
H	2.466446	-0.497008	0.607532

==============================

H_2O

==============================

O	0.000000	0.000000	0.119237
H	0.000000	0.759322	-0.476819
H	0.000000	-0.759322	-0.476819

==============================

H_3O^+

==============================

O	-0.000003	0.000000	-0.070534
H	-0.479665	-0.817077	0.187333
H	-0.467790	0.823925	0.187333
H	0.947428	-0.006848	0.187344

==============================

CO

==============================

C	0.000000	0.000000	-0.650254
O	0.000000	0.000000	0.487690

==============================

11

==============================

C	-0.643701	2.688618	0.315671
C	-1.731084	1.806837	-0.110986
H	-3.916441	-1.014316	0.481151
N	1.556332	1.664027	-0.133181
C	0.638462	2.718980	-0.543607
C	2.561999	-1.604793	-0.978134
C	2.227635	-0.696826	0.230147
C	1.274533	0.377327	-0.255007
C	3.518959	-0.104202	0.818534
C	1.477039	-1.511548	1.313316
O	0.167627	0.062695	-0.839786
H	-0.429938	2.611425	1.387534
H	-1.126373	3.687038	0.198105
H	-1.942717	1.729989	-1.179628
H	-2.576872	1.676123	0.562794
H	0.391375	2.599735	-1.602309
H	1.151854	3.671224	-0.413376
H	3.092530	-1.053594	-1.759043
H	3.207509	-2.418326	-0.639712
H	1.659031	-2.039289	-1.412205
H	4.172877	-0.919528	1.134983
H	4.081257	0.476324	0.079128
H	3.330995	0.510282	1.706290
H	2.143154	-2.284849	1.703191
H	0.599575	-2.016477	0.897537
H	1.175808	-0.882576	2.158696
O	-3.174503	-0.569526	0.922679
Cu	-1.455840	-0.366317	-0.046435
O	-1.520679	-2.324635	-0.822258
H	-1.869974	-3.162916	-0.487339
H	-0.963454	-2.535235	-1.585888
H	2.446529	1.920672	0.280592
H	-3.227755	-0.781899	1.868409

==============================

11-TS'

==============================

C	2.211283	-0.831218	0.428157
C	2.350518	0.487886	0.004497
H	0.483978	3.976199	0.408292
N	0.165381	-2.077492	-0.115260
C	1.557296	-1.877891	-0.486647
C	-3.071010	-1.284074	-1.187728
C	-2.226441	-1.464650	0.096619
C	-0.785180	-1.150368	-0.276482
C	-2.401565	-2.891425	0.642945
C	-2.666525	-0.438515	1.165127
O	-0.468801	-0.034211	-0.788835
H	2.049384	-0.987997	1.498865
H	3.479207	-1.116543	0.335396
H	2.433203	0.707005	-1.059764
H	2.655517	1.269926	0.697336
H	1.618033	-1.552656	-1.529320
H	2.065544	-2.838362	-0.395598
H	-2.785254	-2.006155	-1.957733
H	-4.124074	-1.447259	-0.946401
H	-2.958704	-0.278007	-1.596854
H	-3.455133	-3.056338	0.878352
H	-2.125222	-3.653774	-0.093724
H	-1.843383	-3.056633	1.571780
H	-3.706482	-0.629786	1.439742
H	-2.600419	0.580318	0.777023
H	-2.061853	-0.518516	2.075225
O	1.139631	3.334334	0.084389
Cu	0.156281	1.637844	-0.196014
O	-1.527462	2.971804	0.507495
H	-1.963245	2.872683	1.367039
H	-2.230851	3.214381	-0.113828
H	-0.110200	-2.974256	0.264988
H	1.662124	3.778281	-0.600528
O	4.663016	-1.846890	0.280270
H	5.124345	-2.002471	1.120927
H	5.329462	-1.607226	-0.384639

==============================

12

==============================

C	-1.281993	1.785136	0.681301
C	-2.608470	1.753735	0.328333
H	-2.697062	-2.320667	-0.704102
N	1.091111	1.602308	-0.057207
C	-0.192546	2.283750	-0.248026
C	3.049685	-0.989119	-1.370055
C	2.708777	-0.278654	-0.038647
C	1.282794	0.277317	-0.175507
C	3.758999	0.807004	0.260289

C	2.684429	-1.308049	1.117832
O	0.333945	-0.515367	-0.400809
H	-0.998648	1.666544	1.725898
H	-2.942685	2.053520	-0.659692
H	-3.376164	1.600921	1.077290
H	-0.515543	2.198858	-1.294582
H	-0.009041	3.340843	-0.044024
H	3.085139	-0.274344	-2.207931
H	4.031290	-1.459453	-1.291861
H	2.303496	-1.759739	-1.601166
H	4.745522	0.341879	0.350133
H	3.833256	1.545950	-0.548629
H	3.567821	1.329513	1.209077
H	3.679515	-1.745482	1.237775
H	1.990796	-2.130379	0.897641
H	2.403710	-0.845626	2.071282
O	-2.874882	-1.455946	-1.118237
Cu	-1.697373	-0.151969	-0.025519
O	-1.354717	-2.087545	1.090559
H	-1.476459	-2.278975	2.029229
H	-0.401951	-2.121285	0.913105
H	1.902608	2.177832	0.095796
H	-2.766378	-1.572837	-2.073976

===============================

olefin

===============================

C	2.946537	0.009109	-0.502506
C	4.116289	0.584329	-0.222507
N	0.707200	0.103518	0.479392
C	2.008584	-0.550683	0.535695
C	-2.789904	-0.414465	0.861194
C	-1.726189	0.271425	-0.024536
C	-0.429307	-0.557392	0.097167
C	-1.575630	1.744428	0.389585
C	-2.160057	0.190793	-1.509472
O	-0.432459	-1.763622	-0.143479
H	2.607965	-0.074213	-1.538700
H	4.470387	0.682687	0.807213
H	4.768835	0.970782	-1.002447
H	1.805193	-1.616405	0.350055
H	2.441413	-0.449790	1.540356
H	-2.518134	-0.342799	1.922135
H	-3.761933	0.063827	0.724903
H	-2.868396	-1.471832	0.597862
H	-2.543039	2.254677	0.297297
H	-1.255499	1.842338	1.434312
H	-0.866034	2.281561	-0.249299
H	-3.122197	0.687055	-1.648305
H	-2.252153	-0.857133	-1.815722
H	-1.424548	0.674692	-2.163712
H	0.679484	1.103636	0.602416

===============================

10m

===============================

C	-2.699482	-0.055645	0.421723
C	-2.008492	-1.238029	-0.228435
N	-0.655543	1.149285	-0.138839
C	-2.129494	1.237346	-0.151444
C	2.072951	-0.790560	-1.188204
C	1.543031	0.006776	0.027662
C	0.032262	0.034120	-0.067526
C	2.134264	1.425327	0.027381
C	1.910454	-0.721244	1.343505
O	-0.550683	-1.130651	-0.070758
H	-2.553213	-0.087610	1.505671
H	-3.773768	-0.109228	0.229696
H	-2.195542	-1.296473	-1.303844
H	-2.256336	-2.190931	0.235797
H	-2.459824	1.407246	-1.180832
H	-2.413313	2.104756	0.447351
H	1.817945	-0.298938	-2.131681
H	3.161715	-0.847335	-1.120476
H	1.675862	-1.806979	-1.201352
H	3.221071	1.351808	0.098354
H	1.915941	1.967583	-0.899500
H	1.800939	2.015757	0.887990
H	2.998829	-0.775047	1.419421
H	1.513509	-1.737597	1.360130
H	1.536293	-0.180712	2.217915
H	-0.120983	2.009325	-0.147260

Vibrational Frequencies of the Optimized Geometries

Table S21. Vibrational Frequencies (in cm^{-1}) of the Optimized Geometries for the autocyclization reaction.

==============================
$Cu^{I}(H_2O)_2$
==============================
114.12 114.21 132.46 183.89 185.02 378.55
509.56 618.25 619.05 1648.19 1649.56 3799.01
3802.21 3894.29 3894.30

==============================
H_2O
==============================
1665.28 3799.59 3912.80

==============================
H_3O^+
==============================
783.27 1668.62 1668.78 3583.76 3702.64 3702.85

==============================
CO
==============================
2208.63

11
==============================
18.13 29.01 55.98
65.38 71.43 101.43 116.88 147.15 163.26
174.39 202.14 209.89 226.45 256.30 259.21
264.10 295.26 303.91 317.65 324.01 329.69
352.86 367.67 377.43 388.66 402.64 437.66
476.54 522.46 535.21 579.93 630.16 686.56
717.96 745.76 790.16 793.10 853.82 907.26
940.62 951.46 975.18 978.01 999.07 1041.18
1047.37 1068.37 1112.31 1157.49 1203.71 1222.35
1236.56 1248.52 1297.61 1333.98 1380.28 1383.68
1409.76 1411.19 1414.43 1451.52 1480.15 1490.11
1493.11 1493.66 1498.51 1515.59 1523.78 1532.91
1542.64 1614.65 1647.59 1652.46 2897.29 3044.02
3047.55 3069.33 3090.21 3101.65 3113.24 3116.47
3118.06 3144.47 3148.34 3148.97 3156.41 3173.35
3228.90 3589.29 3752.80 3785.31 3844.54 3882.39

==============================
11-TS'
==============================
-385.93 19.72 26.83
48.44 58.52 66.12 75.67 86.56 100.66
116.18 117.63 124.22 155.24 183.79 207.77
219.26 241.19 250.99 276.97 286.93 298.89
317.04 325.03 329.88 336.14 373.59 382.70
398.99 417.99 426.88 438.53 444.48 456.86
508.94 535.22 557.06 585.71 647.52 651.80
689.97 726.93 784.28 794.71 867.42 909.04
944.17 948.76 954.09 978.67 1000.67 1028.99
1047.90 1056.79 1069.55 1124.49 1150.66 1228.43
1241.22 1258.20 1284.30 1301.74 1333.50 1391.03
1394.63 1410.16 1414.96 1444.13 1450.81 1487.41
1493.01 1500.36 1502.15 1515.77 1525.31 1537.83
1573.46 1593.19 1611.88 1640.01 1651.03 1657.67
3042.81 3054.25 3067.40 3089.78 3112.47 3115.47
3131.14 3134.33 3143.47 3146.19 3151.94 3157.25
3159.92 3241.20 3616.55 3735.20 3738.19 3775.42
3846.29 3847.08 3869.92
==============================
12
==============================
42.43 50.49 62.40 77.49 82.56 98.25
115.27 154.10 159.29 204.27 233.34 245.74
267.06 274.49 290.77 301.04 311.06 320.50

331.48 338.27 348.67 363.04 370.95 385.27
401.00 408.53 470.12 519.46 532.62 541.43
544.73 609.00 642.61 670.00 776.79 800.04
861.50 899.74 943.80 947.61 953.87 985.23
1000.31 1022.36 1044.21 1051.13 1078.33 1149.13
1231.76 1242.58 1257.77 1282.81 1292.96 1322.66
1363.15 1411.70 1418.33 1448.79 1450.75 1487.18
1495.02 1503.59 1508.93 1517.94 1527.19 1540.45
1576.54 1600.22 1612.37 1641.13 1664.75 3011.05
3016.04 3031.40 3042.44 3080.77 3088.81 3100.37
3117.58 3121.89 3129.08 3148.04 3172.07 3177.46
3273.95 3680.40 3709.25 3765.64 3845.85 3893.26

==============================
olefin
==============================
47.26 65.69 71.56 140.68 222.72 231.00
266.03 282.90 301.80 311.65 337.97 366.71
371.12 392.10 443.49 510.83 520.93 579.90
689.28 771.00 792.14 871.36 934.72 938.41
951.82 964.24 973.17 1014.14 1044.65 1049.53
1050.62 1084.69 1167.89 1215.66 1233.08 1260.38
1278.60 1305.71 1323.90 1378.48 1400.77 1406.54
1438.62 1468.14 1490.65 1500.43 1509.40 1510.63
1513.89 1524.40 1542.62 1547.12 1727.08 1747.89
3015.95 3022.68 3036.66 3040.68 3063.36 3087.88
3099.57 3106.90 3108.41 3117.10 3124.06 3139.57
3150.67 3206.54 3658.62
==============================
10m
==============================
31.59 103.02 168.25
210.50 216.41 263.41 269.85 311.59 313.90
332.52 339.11 387.92 468.20 503.70 516.07
603.90 700.19 755.17 793.35 841.03 878.75
899.97 920.17 942.21 956.11 974.55 979.70
1047.43 1050.63 1074.49 1093.94 1145.74 1224.14
1227.35 1238.21 1250.33 1263.27 1306.28 1326.15
1358.07 1401.90 1410.84 1415.09 1419.19 1454.40
1492.08 1493.12 1499.23 1502.44 1505.32 1516.05
1522.11 1524.91 1538.94 1570.20 1680.90 3041.73
3063.01 3065.20 3080.64 3085.79 3100.84 3111.28
3138.80 3140.50 3141.46 3146.29 3148.03 3158.76
3162.39 3182.76 3609.13

7. References

(1) Xie, W.; Zhao, M.; Cui, C. Cesium Carbonate-Catalyzed Reduction of Amides with Hydrosilanes. *Organometallics* **2013**, *32* (24), 7440–7444. https://doi.org/10.1021/om400951n.

(2) Topczewski, J. J.; Cabrera, P. J.; Saper, N. I.; Sanford, M. S. Palladium-Catalysed Transannular C–H Functionalization of Alicyclic Amines. *Nature* **2016**, *531* (7593), 220–224. https://doi.org/10.1038/nature16957.

(3) Roque, J. B.; Kuroda, Y.; Göttemann, L. T.; Sarpong, R. Deconstructive Diversification of Cyclic Amines. *Nature* **2018**, *564* (7735), 244–248. https://doi.org/10.1038/s41586-018-0700-3.

(4) Seebach, D.; Lohmann, J.-J.; Syfrig, M. A.; Yoshifuji, M. Alkylation of the Isoquinoline Skeleton in the 1-Position: Lithiated 2-Pivaloyl- and 2-Bis(Dimethylamino)-Phosphinoyl-1,2,3,4-Tetrahydroisoquinolines. *Tetrahedron* **1983**, *39* (12), 1963–1974. https://doi.org/10.1016/S0040-4020(01)91914-3.

(5) Rao, Y.; Li, X.; Danishefsky, S. J. Thio FCMA Intermediates as Strong Acyl Donors: A General Solution to the Formation of Complex Amide Bonds. *J. Am. Chem. Soc.* **2009**, *131* (36), 12924–12926. https://doi.org/10.1021/ja906005j.

(6) Metternich, J. B.; Sagebiel, S.; Lückener, A.; Lamping, S.; Ravoo, B. J.; Gilmour, R. Covalent Immobilization of (−)-Riboflavin on Polymer Functionalized Silica Particles: Application in the Photocatalytic E→Z Isomerization of Polarized Alkenes. *Chem. – Eur. J.* **2018**, *24* (17), 4228–4233. https://doi.org/10.1002/chem.201800231.

(7) Vasilopoulos, A.; Krska, S. W.; Stahl, S. S. C(Sp3)–H Methylation Enabled by Peroxide Photosensitization and Ni-Mediated Radical Coupling. *Science* **2021**, *372* (6540), 398–403. https://doi.org/10.1126/science.abh2623.

(8) Kochi, J. K. Mechanisms of Organic Oxidation and Reduction by Metal Complexes. *Science* **1967**, *155* (3761), 415–424. https://doi.org/10.1126/science.155.3761.415.

(9) Frisch, M. J.; Trucks, G. W.; Schlegel, H. B.; Scuseria, G. E.; Robb, M. A.; Cheeseman, J. R.; Scalmani, G.; Barone, V.; Petersson, G. A.; Nakatsuji, H.; Li, X.; Caricato, M.; Marenich, A. V.; Bloino, J.; Janesko, B. G.; Gomperts, R.; Mennucci, B.; Hratchian, H. P.; Ortiz, J. V.; Izmaylov, A. F.; Sonnenberg, J. L.; Williams-Young, D.; Ding, F.; Lipparini, F.; Egidi, F.; Goings, J.; Peng, B.; Petrone, A.; Henderson, T.; Ranasinghe, D.; Zakrzewski, V. G.; Gao, J.; Rega, N.; Zheng, G.; Liang, W.; Hada, M.; Ehara, M.; Toyota, K.; Fukuda, R.; Hasegawa, J.; Ishida, M.; Nakajima, T.; Honda, Y.; Kitao, O.; Nakai, H.; Vreven, T.; Throssell, K.; Montgomery, J. A., Jr.; Peralta, J. E.; Ogliaro, F.; Bearpark, M. J.; Heyd, J. J.; Brothers, E. N.; Kudin, K. N.; Staroverov, V. N.; Keith, T. A.; Kobayashi, R.; Normand, J.; Raghavachari, K.; Rendell, A. P.; Burant, J. C.; Iyengar, S. S.; Tomasi, J.; Cossi, M.; Millam, J. M.; Klene, M.; Adamo, C.; Cammi, R.; Ochterski, J. W.; Martin, R. L.; Morokuma, K.; Farkas, O.; Foresman, J. B.; Fox, D. J. Gaussian 16, Revision C.01, 2016.

(10) Hay, P. J.; Wadt, W. R. Ab Initio Effective Core Potentials for Molecular Calculations. Potentials for the Transition Metal Atoms Sc to Hg. *J. Chem. Phys.* **1985**, *82* (1), 270–283. https://doi.org/10.1063/1.448799.

(11) Hay, P. J.; Wadt, W. R. Ab Initio Effective Core Potentials for Molecular Calculations. Potentials for K to Au Including the Outermost Core Orbitals. *J. Chem. Phys.* **1985**, *82* (1), 299–310. https://doi.org/10.1063/1.448975.

(12) Wadt, W. R.; Hay, P. J. Ab Initio Effective Core Potentials for Molecular Calculations. Potentials for Main Group Elements Na to Bi. *J. Chem. Phys.* **1985**, *82* (1), 284–298. https://doi.org/10.1063/1.448800.

(13) Becke, A. D. Density-Functional Exchange-Energy Approximation with Correct Asymptotic Behavior. *Phys. Rev. A* **1988**, *38* (6), 3098–3100. https://doi.org/10.1103/PhysRevA.38.3098.

(14) Lee, C.; Yang, W.; Parr, R. G. Development of the Colle-Salvetti Correlation-Energy Formula into a Functional of the Electron Density. *Phys. Rev. B* **1988**, *37* (2), 785–789. https://doi.org/10.1103/PhysRevB.37.785.

(15) Becke, A. D. A New Mixing of Hartree–Fock and Local Density-functional Theories. *J. Chem. Phys.* **1993**, *98* (2), 1372–1377. https://doi.org/10.1063/1.464304.

(16) Grimme, S.; Antony, J.; Ehrlich, S.; Krieg, H. A Consistent and Accurate Ab Initio Parametrization of Density Functional Dispersion Correction (DFT-D) for the 94 Elements H-Pu. *J. Chem. Phys.* **2010**, *132* (15), 154104. https://doi.org/10.1063/1.3382344.

(17) Becke, A. D.; Johnson, E. R. A Density-Functional Model of the Dispersion Interaction. *J. Chem. Phys.* **2005**,

123 (15), 154101. https://doi.org/10.1063/1.2065267.

(18) Becke, A. D.; Johnson, E. R. Exchange-Hole Dipole Moment and the Dispersion Interaction. *J. Chem. Phys.* **2005**, *122* (15), 154104. https://doi.org/10.1063/1.1884601.

(19) Johnson, E. R.; Becke, A. D. A Post-Hartree-Fock Model of Intermolecular Interactions: Inclusion of Higher-Order Corrections. *J. Chem. Phys.* **2006**, *124* (17), 174104. https://doi.org/10.1063/1.2190220.

(20) Barone, V.; Cossi, M. Quantum Calculation of Molecular Energies and Energy Gradients in Solution by a Conductor Solvent Model. *J. Phys. Chem. A* **1998**, *102* (11), 1995–2001. https://doi.org/10.1021/jp9716997.

(21) Cossi, M.; Rega, N.; Scalmani, G.; Barone, V. Energies, Structures, and Electronic Properties of Molecules in Solution with the C-PCM Solvation Model. *J. Comput. Chem.* **2003**, *24* (6), 669–681. https://doi.org/10.1002/jcc.10189.

(22) Burke, K.; Werschnik, J.; Gross, E. K. U. Time-Dependent Density Functional Theory: Past, Present, and Future. *J. Chem. Phys.* **2005**, *123* (6), 062206. https://doi.org/10.1063/1.1904586.

(23) Haines, B. E.; Musaev, D. G. Hydrogen-Bonding as a Factor to Determine the Regioselectivity for Pd-Mediated C−H Activation of Pyridine. *ChemCatChem* **2021**, *13* (4), 1201–1206. https://doi.org/10.1002/cctc.202001658.

(24) McLarney, B. D.; Hanna, S.; Musaev, D. G.; France, S. Predictive Model for the [Rh2(Esp)2]-Catalyzed Intermolecular C(Sp3)–H Bond Insertion of β-Carbonyl Ester Carbenes: Interplay between Theory and Experiment. *ACS Catal.* **2019**, *9* (5), 4526–4538. https://doi.org/10.1021/acscatal.9b00889.

(25) Sperger, T.; Sanhueza, I. A.; Kalvet, I.; Schoenebeck, F. Computational Studies of Synthetically Relevant Homogeneous Organometallic Catalysis Involving Ni, Pd, Ir, and Rh: An Overview of Commonly Employed DFT Methods and Mechanistic Insights. *Chem. Rev.* **2015**, *115* (17), 9532–9586. https://doi.org/10.1021/acs.chemrev.5b00163.

(26) Ahn, S.; Hong, M.; Sundararajan, M.; Ess, D. H.; Baik, M.-H. Design and Optimization of Catalysts Based on Mechanistic Insights Derived from Quantum Chemical Reaction Modeling. *Chem. Rev.* **2019**, *119* (11), 6509–6560. https://doi.org/10.1021/acs.chemrev.9b00073.

(27) Parr, R. G.; Weitao, Y. *Density-Functional Theory of Atoms and Molecules*; International Series of Monographs on Chemistry; Oxford University Press: New York, 1995. https://doi.org/10.1093/oso/9780195092769.001.0001.

(28) Frisch, M. J.; Trucks, G. W.; Schlegel, H. B.; Scuseria, G. E.; Robb, M. A.; Cheeseman, J. R.; Scalmani, G.; Barone, V.; Mennucci, B.; Petersson, G. A.; Nakatsuji, H.; Caricato, M.; Li, X.; Hratchian, H. P.; Izmaylov, A. F.; Bloino, J.; Zheng, G.; Sonnenberg, J. L.; Hada, M.; Ehara, M.; Toyota, K.; Fukuda, R.; Hasegawa, J.; Ishida, M.; Nakajima, T.; Honda, Y.; Kitao, O.; Nakai, H.; Vreven, T.; Montgomery, J. A., Jr.; Peralta, J. E.; Ogliaro, F.; Bearpark, M. J.; Heyd, J. J.; Brothers, E. N.; Kudin, K. N.; Staroverov, V. N.; Keith, T. A.; Kobayashi, R.; Normand, J.; Raghavachari, K.; Rendell, A. P.; Burant, J. C.; Iyengar, S. S.; Tomasi, J.; Cossi, M.; Rega, N.; Millam, J. M.; Klene, M.; Knox, J. E.; Cross, J. B.; Bakken, V.; Adamo, C.; Jaramillo, J.; Gomperts, R.; Stratmann, R. E.; Yazyev, O.; Austinm A. J.; Cammi, R.; Pomelli, C.; Ochterski, J. W.; Martin, R. L.; Morokuma, K.; Zakrzewski, V. G.; Voth, G. A.; Salvador, P.; Dannesberg, J. J.; Dapprich, S.; Daniels, A. D.; Farkas, O.; Foresman, J. B.; Ortiz, J. V.; Cioslowski, J.; Fox, D. J. Gaussian 09, Revision D.01, 2013.

(29) Slater, J. C. *Quantum Theory of Molecules and Solids. Vol. 4. Vol. 4.*; McGraw-Hill: New York, NY, 1974.

(30) Vosko, S. H.; Wilk, L.; Nusair, M. Accurate Spin-Dependent Electron Liquid Correlation Energies for Local Spin Density Calculations: A Critical Analysis. *Can. J. Phys.* **1980**, *58* (8), 1200–1211. https://doi.org/10.1139/p80-159.

(31) Becke, A. D. Density-functional Thermochemistry. III. The Role of Exact Exchange. *J. Chem. Phys.* **1993**, *98* (7), 5648–5652. https://doi.org/10.1063/1.464913.

(32) Grimme, S.; Ehrlich, S.; Goerigk, L. Effect of the Damping Function in Dispersion Corrected Density Functional Theory. *J. Comput. Chem.* **2011**, *32* (7), 1456–1465. https://doi.org/10.1002/jcc.21759.

(33) Ditchfield, R.; Hehre, W. J.; Pople, J. A. Self-Consistent Molecular-Orbital Methods. IX. An Extended Gaussian-Type Basis for Molecular-Orbital Studies of Organic Molecules. *J. Chem. Phys.* **1971**, *54* (2), 724–728. https://doi.org/10.1063/1.1674902.

(34) Hehre, W. J.; Pople, J. A. Self-Consistent Molecular Orbital Methods. XIII. An Extended Gaussian-Type Basis for Boron. *J. Chem. Phys.* **1972**, *56* (8), 4233–4234. https://doi.org/10.1063/1.1677844.

(35) Binkley, J. S.; Pople, J. A. Self-consistent Molecular Orbital Methods. XIX. Split-valence Gaussian-type

Basis Sets for Beryllium. *J. Chem. Phys.* **1977**, *66* (2), 879–880. https://doi.org/10.1063/1.433929.

(36) Hariharan, P. C.; Pople, J. A. The Influence of Polarization Functions on Molecular Orbital Hydrogenation Energies. *Theor. Chim. Acta* **1973**, *28* (3), 213–222. https://doi.org/10.1007/BF00533485.

(37) Hehre, W. J.; Ditchfield, R.; Pople, J. A. Self—Consistent Molecular Orbital Methods. XII. Further Extensions of Gaussian—Type Basis Sets for Use in Molecular Orbital Studies of Organic Molecules. *J. Chem. Phys.* **1972**, *56* (5), 2257–2261. https://doi.org/10.1063/1.1677527.

(38) Francl, M. M.; Pietro, W. J.; Hehre, W. J.; Binkley, J. S.; Gordon, M. S.; DeFrees, D. J.; Pople, J. A. Self-consistent Molecular Orbital Methods. XXIII. A Polarization-type Basis Set for Second-row Elements. *J. Chem. Phys.* **1982**, *77* (7), 3654–3665. https://doi.org/10.1063/1.444267.

(39) Clark, T.; Chandrasekhar, J.; Spitznagel, G. W.; Schleyer, P. V. R. Efficient Diffuse Function-Augmented Basis Sets for Anion Calculations. III. The 3-21+G Basis Set for First-Row Elements, Li–F. *J. Comput. Chem.* **1983**, *4* (3), 294–301. https://doi.org/10.1002/jcc.540040303.

(40) Frisch, M. J.; Pople, J. A.; Binkley, J. S. Self-consistent Molecular Orbital Methods 25. Supplementary Functions for Gaussian Basis Sets. *J. Chem. Phys.* **1984**, *80* (7), 3265–3269. https://doi.org/10.1063/1.447079.

(41) Krishnan, R.; Binkley, J. S.; Seeger, R.; Pople, J. A. Self-consistent Molecular Orbital Methods. XX. A Basis Set for Correlated Wave Functions. *J. Chem. Phys.* **1980**, *72* (1), 650–654. https://doi.org/10.1063/1.438955.

(42) McLean, A. D.; Chandler, G. S. Contracted Gaussian Basis Sets for Molecular Calculations. I. Second Row Atoms, Z=11–18. *J. Chem. Phys.* **1980**, *72* (10), 5639–5648. https://doi.org/10.1063/1.438980.

(43) Küchle, W.; Dolg, M.; Stoll, H.; Preuss, H. Energy-adjusted Pseudopotentials for the Actinides. Parameter Sets and Test Calculations for Thorium and Thorium Monoxide. *J. Chem. Phys.* **1994**, *100* (10), 7535–7542. https://doi.org/10.1063/1.466847.

(44) Cancès, E.; Mennucci, B.; Tomasi, J. A New Integral Equation Formalism for the Polarizable Continuum Model: Theoretical Background and Applications to Isotropic and Anisotropic Dielectrics. *J. Chem. Phys.* **1997**, *107* (8), 3032–3041. https://doi.org/10.1063/1.474659.
